# Iron‐Catalyzed Cross‐Coupling of Alkynyl and Styrenyl Chlorides with Alkyl Grignard Reagents in Batch and Flow

**DOI:** 10.1002/chem.201904480

**Published:** 2019-10-22

**Authors:** Yuchao Deng, Xiao‐Jing Wei, Xiao Wang, Yuhan Sun, Timothy Noël

**Affiliations:** ^1^ Department of Chemical Engineering and Chemistry, Micro Flow Chemistry and Synthetic Methodology Eindhoven University of Technology Den Dolech 2 5612 AZ Eindhoven The Netherlands; ^2^ School of Physical Science and Technology ShanghaiTech University Shanghai 201210 P. R. China; ^3^ School of Chemistry and Chemical Engineering Nanjing University Nanjing 210023 P. R. China; ^4^ Shanghai Advanced Research Institute Chinese Academy of Sciences Shanghai 201210 P. R. China

**Keywords:** catalysis, cross-coupling, flow chemistry, Grignard reagents, iron

## Abstract

Transition‐metal‐catalyzed cross‐coupling chemistry can be regarded as one of the most powerful protocols to construct carbon–carbon bonds. While the field is still dominated by palladium catalysis, there is an increasing interest to develop protocols that utilize cheaper and more sustainable metal sources. Herein, we report a selective, practical, and fast iron‐based cross‐coupling reaction that enables the formation of Csp−Csp^3^ and Csp^2^−Csp^3^ bonds. In a telescoped flow process, the reaction can be combined with the Grignard reagent synthesis. Moreover, flow allows the use of a supporting ligand to be avoided without eroding the reaction selectivity.

## Graphical abstract

**A broadly applicable** Fe‐catalyzed cross‐coupling of alkynyl and styrenyl chlorides with alkyl Grignard reagents is reported (see scheme). The reaction displays a broad scope and can be carried out under mild conditions in very short reaction times in batch. The use of flow allows for the process integration of the Grignard reagent synthesis and the Fe‐based cross‐coupling chemistry.

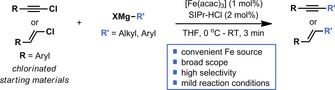

Transition‐metal‐catalyzed cross‐coupling reactions serve as one of the most powerful protocols to construct carbon–carbon and carbon–heteroatom bonds in a variety of biologically active molecules,^1^ natural products,^2^ and functional materials.^3^ To date, the workhorse of cross‐coupling chemistry has been palladium, which in combination with suitable ligands allowed high catalytic efficiency to be enacted for a wide variety of electrophile–nucleophile combinations.^4^ However, due to the scarcity and increasing cost of palladium and the stringent heavy metal regulations in the pharmaceutical industry, alternatives for palladium are currently of high interest.^5^ Amongst potential candidates, earth‐abundant first row transition metals provide arguably the highest likelihood to replace palladium due to their reduced cost and low toxicity.^6^ In this regard, iron has received substantial attention as it is a metal with minimum safety concern and it provides many catalytic options as its oxidation states range from −II to +VI.^7^ However, despite the great potential to cover essentially all relevant catalytic transformations in organic synthesis, reality is different and iron proves to be notorious to tame, hindering its widespread adoption.^8^

While the classical Sonogashira reaction enables the efficient coupling between aryl halides and terminal alkynes,^9^ metal‐catalyzed Csp–Csp^3^ couplings are very rare (Scheme 1). Cahiez et al. found that alkyl–alkynyl cross‐coupling can be achieved using copper catalysis and slow addition of the Grignard coupling partner.^10^ A cobalt‐enabled coupling between bromoalkynes and organozinc halide nucleophiles was described by Gosmini and co‐workers. The groups of Nakamura^11^ and Hu^12^ developed iron‐catalyzed protocols to couple alkyl bromides and iodides with alkynyl Grignard reagents.^13^ In search of synthetically useful Fe‐catalyzed cross‐coupling reactions,^14^ we describe herein our efforts to develop a robust protocol to cross‐couple both styrenyl and alkynyl chlorides with alkyl Grignard reagents by using an Fe catalyst and an NHC ligand.^15^ This method provides a set of conditions that are both practical and widely applicable in Csp−Csp^3^ and Csp^2^−Csp^3^ bond‐forming reactions. Interestingly, the Fe‐based coupling reaction could be translated to flow and was combined with an inline generation of Grignard reagents. Furthermore, the flow strategy allowed the reaction to be carried out under mild conditions whilst avoiding the use of an NHC ligand, thus simplifying the overall process.

**Scheme 1:**
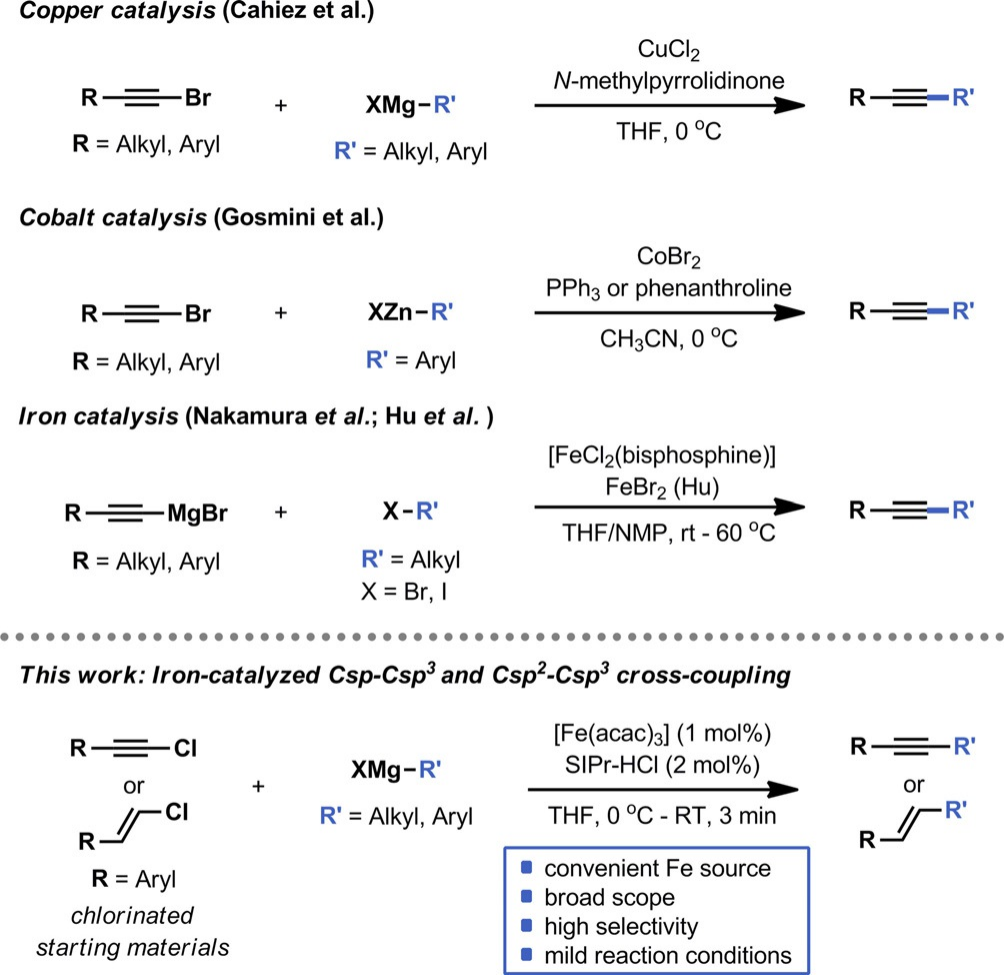
Established metal‐catalyzed Csp–Csp^3^ coupling reactions and reaction design of an Fe‐based protocol to enable the Csp–Csp^3^ and Csp^2^–Csp^3^ coupling.

Initial cross‐coupling experiments started with 1‐chloro‐2‐phenylacetylene as a benchmark substrate and cyclohexyl‐magnesium chloride in ethereal solvents at 0 °C (Table 1). With FeCl_3_ **⋅**6 H_2_O as the iron source, the use of THF as a solvent was preferred over Et_2_O (Table 1, entries 1 and 2). In both cases, substantial amounts of byproducts were observed resulting from homocoupling (**2 a′**) and reduction (**2 a′′**). Switching to an FeCl_2_ **⋅**4 H_2_O catalyst resulted in a diminished reactivity (Table 1, entry 3). A higher selectivity and reactivity for the desired cross‐coupled product (**2 a**) was observed using [Fe(acac)_3_] (Table 1, entry 4). However, the highest selectivities were obtained when catalyst complexes arising from [Fe(acac)_3_] and NHC ligands were used, with the SIPr ligand providing the best results in terms of reaction efficiency and selectivity. (Table 1, entries 5 and 6). A lower selectivity was observed when the reaction temperature was raised to room temperature (Table 1, entry 7). In the absence of an iron catalyst, the reaction produces only a limited amount of product along with unidentified byproducts (Table 1, entry 8).^16^

**Table 1:** Optimization of the reaction conditions for the iron‐catalyzed cross‐coupling between alkynyl chlorides and alkyl Grignard reagents.^[a]^

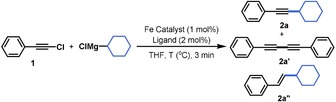							
Entry	Catalyst	Ligand	*T* [°C]	Conv. (**5**)^[a]^	**2 a** [%]^[b]^	**2 a“** [%]^[b]^	**2 a′′** [%]^[b]^
1^[c]^	FeCl_3_ **⋅**6 H_2_O	–	0	88	66	5	18
2	FeCl_3_ **⋅**6 H_2_O	–	0	100	80	12	8
3	FeCl_2_ **⋅**4 H_2_O	–	0	32	27	1	4
4	[Fe(acac)_3_]	–	0	90	77	3	10
5	[Fe(acac)_3_]	L1	0	71	69	–	2
6	[Fe(acac)_3_]	L2	0	100	97	1	2
7	[Fe(acac)_3_]	L2	r.t.	100	91	6	3
8	–	–	0	81	12	–	–
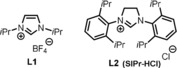							

[a] Standard reaction conditions: **1** (0.5 mmol), cyclohexylmagnesium chloride (1.0 m in THF, 0.6 mmol), THF (1.9 mL, 0.2 m), Fe catalyst (1 mol %) and SIPr‐HCl (2 mol %). [b] Conversion and yields were determined by GCMS. [c] Et_2_O instead of THF.

With optimal conditions in hand, we probed the generality of his protocol for the coupling of alkynyl chlorides with Grignard reagents (Figure 1). Various alkynyl chlorides with electron‐neutral (**2 a**, **2 c**, **2 d**), ‐withdrawing (**2 b**), and ‐donating groups (**2 e**) underwent efficient cross‐coupling with cyclohexylmagnesium chloride (89–96 % yields). 1‐Chloro‐2‐phenylacetylene could be efficiently coupled with a diverse set of aliphatic Grignard reagents, including phenylmagnesium chloride (**2 f**), propylmagnesium chloride (**2 g**), methylmagnesium chloride (**2 h**), (trimethylsilyl)methylmagnesium chloride (**2 i**), and cyclopentyl magnesium chloride (**2 j**) (81–93 % yields). Also Grignard reagents decorated with medicinally important scaffolds, such as *N*‐methylpiperidine (**2 k**), can be tolerated (99 % yield). Finally, also alkylated alkynyl chlorides and aromatic Grignard reagents (**2 l**) can be coupled in this protocol furnishing the targeted product in 90 % isolated yield.

**Figure 1:**
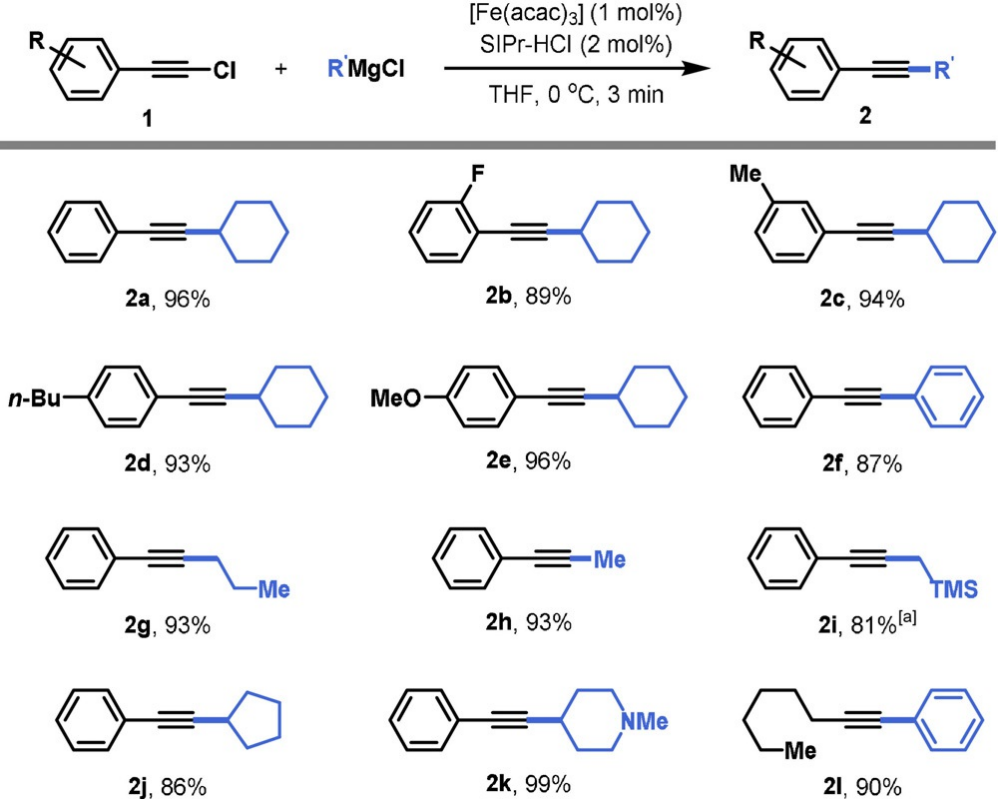
Scope of the iron‐catalyzed cross‐coupling between alkynyl chlorides and alkyl Grignard reagents. Reaction conditions: 0.5 mmol **1**, 0.6 mmol R′MgCl, 1 mol % [Fe(acac)_3_], 2 mol % SIPr‐HCl in 2.5 mL THF at 0 °C. [a] 2 mol % [Fe(acac)_3_] and 4 mol % SIPr‐HCl.

Expanding the substrate scope to involve styrenyl chlorides in this Fe‐catalyzed cross‐coupling protocol permitted us to forge Csp^2^−Csp^3^ bonds as well (Figure 2). Interestingly, for most substrates, the reaction could be completed at room temperature without adding any supporting ligand. β‐Chlorostyrene can be rapidly and efficiently coupled with assorted aliphatic (**4 a**–**g**, 90–96 % yield) and aromatic (**4 h**) (97 % yield) Grignard nucleophiles. The protocol is easily scalable without reduced efficiency (**4 f**, 8 mmol, 90 % yield). β‐Chlorostyrenes bearing electron‐neutral (**4 i**–**l**), ‐donating (**4 m**–**q**), and ‐withdrawing groups (**4 r**) at the *ortho*‐, *meta*‐ and *para*‐positions are readily tolerated (89–97 % yield). The reaction does not display a great sensitivity to steric hindrance, as both naphthyl substrates (**4 s**,**t**, 91–94 % yields) and α‐substituted β‐chlorostyrenes (**4 u**,**v**, 94 % yield) were efficiently coupled with cyclohexylmagnesium chloride. Double functionalization was also possible, albeit at a slightly diminished yield (**4 w**, 57 % yield). The reaction was stereoselective in all cases and even the cross‐coupled product derived from (*Z*)‐ β‐chlorostyrene was obtained in good yield and with retained stereoselectivity (**4 x**, 93 % yield).

**Figure 2:**
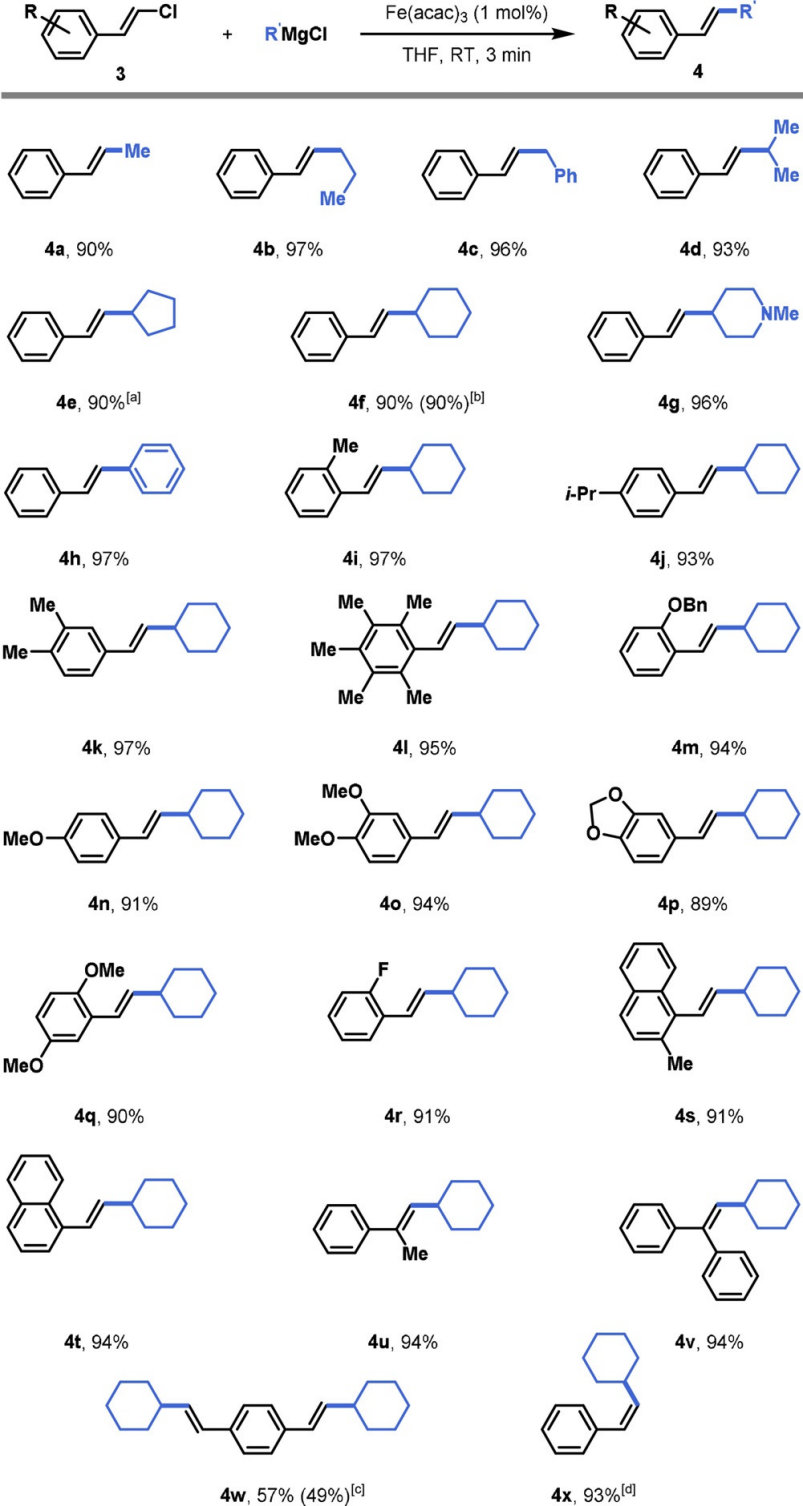
Scope of the iron‐catalyzed cross‐coupling between styrenyl chlorides and alkyl Grignard reagents. Reaction conditions: 0.5 mmol **3**, 0.6 mmol R′MgCl, 1 mol % [Fe(acac)_3_]. in 2.5 mL THF at room temperature. [a] 3 mol % [Fe(acac)_3_] and 6 mol % SIPr‐HCl was added. [b] Scale‐up experiment on an 8 mmol scale. [c] Scale‐up experiment on an 4.7 mmol scale. [d] The *Z*/*E* ratio of starting material **3 x** was 91:9 and of product **4 x** was 88:12.

Next, we investigated the possibility to telescope both the Grignard reagent synthesis and the iron‐catalyzed cross‐coupling transformation in a single, streamlined continuous‐flow process. The combination of these two individual steps allows the safe control of the exotherm of the Grignard reagent synthesis,^16^ to keep the total inventory of potentially hazardous Grignard reagents low and to use cheap organohalides as starting materials.^17^ For the preparation of the Grignard reagent, we filled an open column with magnesium according to the procedure reported by Alcazar et al.^18^ Over this magnesium packed‐bed reactor, a solution of alkyl or aryl halide was directed and the generated Grignard reagent was merged with the reagents required for the Fe‐catalyzed cross‐coupling transformation (Table 2).^19^ The combined reaction mixture was fed to a capillary microreactor (perfluoroalkoxy alkane, PFA; 750 μm ID). The coupling between β‐chlorostyrene and *n*‐pentylmagnesium bromide resulted in the formation of the corresponding cross‐coupled product in 95 % isolated yield, requiring only 30 s residence time (Table 2, entry 1). Next, β‐chlorostyrene and 1‐chloro‐2‐phenylacetylene can be reacted with in situ‐generated 3‐butenylmagnesium bromide (Table 2, entries 2 and 3). Interestingly, the yield and selectivity in flow was systematically higher due a better dissipation of the reaction exotherm, which can be attributed to the increased surface‐to‐volume ratio, and to the enhanced mixing efficiency in the microreactor setup (Table 2, entry 3–11).^20^ Furthermore, this feature allowed the addition of a supporting NHC ligand to be avoided without deterioration of the selectivity of the transformation.

**Table 2:** Telescoped organomagnesium bromide synthesis and iron‐catalyzed cross‐coupling in flow. For detailed reaction conditions, see the Supporting Information.

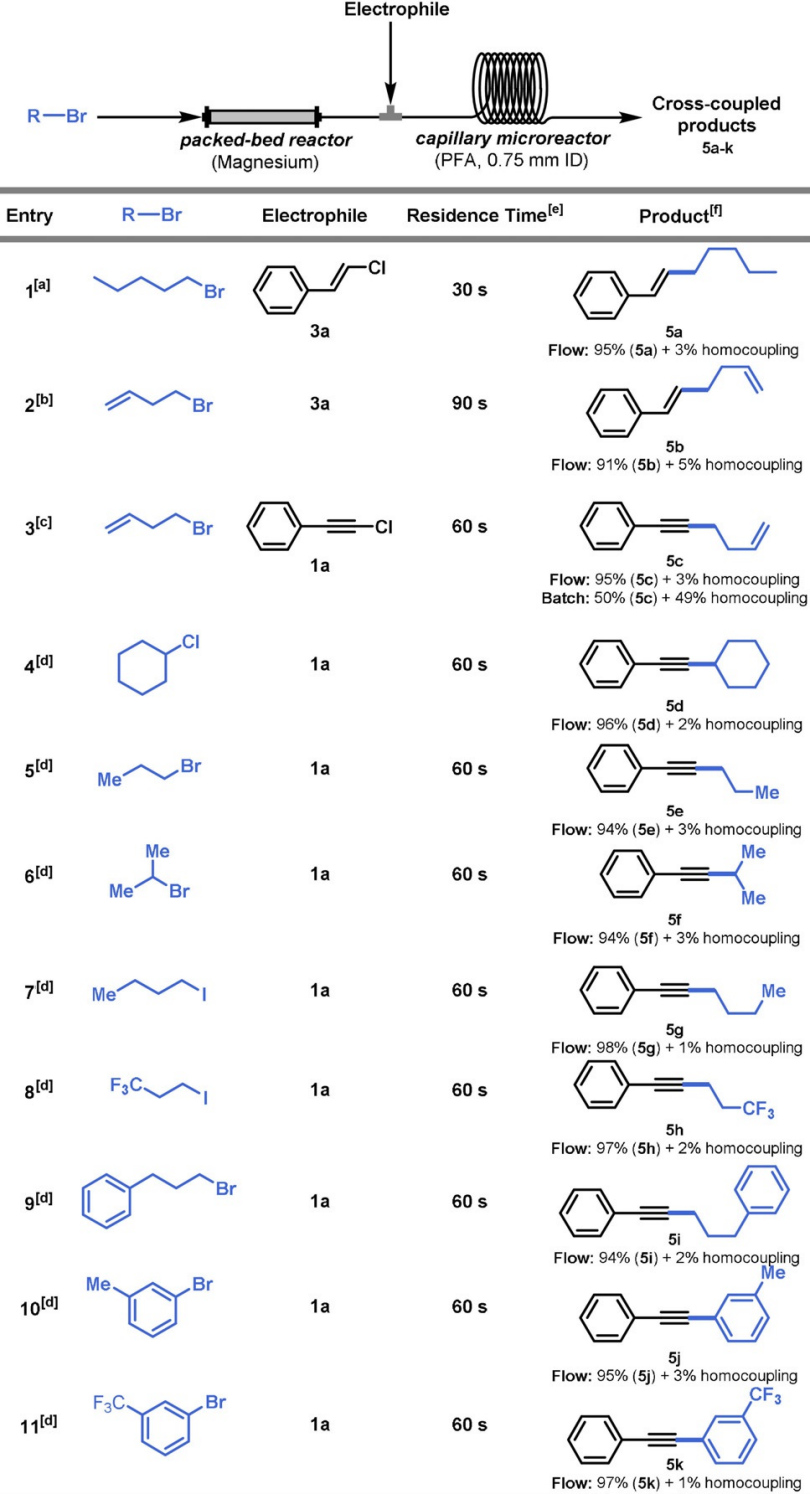

[a] [Fe(acac)_3_] (1 mol %), electrophile (0.31 m), room temperature. [b] [Fe(acac)_3_] (2 mol %), electrophile (0.33 m), room temperature. [c] [Fe(acac)_3_] (2 mol %), electrophile (0.33 m), 0 °C. [d] [Fe(acac)_3_] (1 mol %), electrophile (based on the concentration of the Grignard reagent), 0 °C. [e] Residence time denotes the time spent in the capillary microreactor. [f] Homocoupling compound was determined by GCMS.

In conclusion, we have developed a practical and mild iron‐catalyzed cross‐coupling method to establish Csp–Csp^3^ and Csp^2^–Csp^3^ linkages. The protocol utilizes an NHC ligand to efficiently couple alkynyl chlorides and alkyl Grignard reagents, while no supporting ligand is needed for the functionalization of styrenyl chlorides. Interestingly, the Fe‐based cross‐coupling reaction can be translated to flow and be combined with the synthesis of Grignard reagents in a single, uninterrupted continuous process. A salient feature of the flow protocol is that the use of an NHC ligand can be avoided for the Csp–Csp^3^ coupling without compromising the reaction selectivity, which is attributed to the improved temperature control in a microreactor.

## Conflict of interest

The authors declare no conflict of interest.

## Supporting information

As a service to our authors and readers, this journal provides supporting information supplied by the authors. Such materials are peer reviewed and may be re‐organized for online delivery, but are not copy‐edited or typeset. Technical support issues arising from supporting information (other than missing files) should be addressed to the authors.

Supplementary
