# Supplementary material for: Iron‐Catalyzed Cross‐Coupling of Alkynyl and Styrenyl Chlorides with Alkyl Grignard Reagents in Batch and Flow
Source: Chemistry. 2019 Oct 22;25(64):14532–5. doi: 10.1002/chem.201904480 (PMC6900226; doi:10.1002/chem.201904480)
Supplement: Supplementary file 1 — Supplementary [file CHEM-25-14532-s001.pdf]

CHEMISTRY
A European Journal

Supporting Information

Iron-Catalyzed Cross-Coupling of Alkynyl and Styrenyl Chlorides with Alkyl Grignard Reagents in Batch and Flow

Yuchao Deng+,[a, b] Xiao-Jing Wei+,[a] Xiao Wang,[c] Yuhan Sun,[b, d] and Timothy Noël*[a]

chem_201904480_sm_miscellaneous_information.pdf

Table of Contents

1. General information 3

2. General procedure for the synthesis of α, β-unsaturated aromatic chlorides 4

   2.1 General procedure for the synthesis of 1-chloroalkynes GP1 4

   2.2 General procedure for the synthesis of styrenyl chlorides GP2 4

   2.3 General procedure for the synthesis of *(Z)*-(2-chlorovinyl)benzene GP3 6

3. General procedure for the Fe-catalyzed C–C cross-coupling 7

   3.1 General procedure for the coupling of alkynyl chlorides with Grignard reagent in batch (GP4) 7

   3.2 General procedure the coupling of alkenyl chlorides with Grignard reagent in batch (GP5) ... 7

4. General procedure for the synthesis of organomagnesium reagent in flow (GP6) 7

5. General procedure for the telescope reaction GP7 10

   5.1 General procedure for telescope reactions 10

   5.2 Set up for the telescope reactions 10

6. Preliminary mechanistic study 12

7. Limitations to the reaction scope 14

8. Characterization data 15

8.1 Characterization data of cross-coupling of the products 15

8.2 Characterization of the starting material: 28

9. Reference 33

10. NMR spectra 34

1. General information

All components as well as reagents and solvents were used as received without further purification, unless stated otherwise. Reagents and solvents were bought from Sigma Aldrich and TCI and if applicable, kept under argon atmosphere. Technical solvents were bought from VWR International and used as received. Product isolation was performed using silica (60, F254, Merck™), and TLC analysis was performed using Silica on aluminum foils TLC plates (F254, Supelco Sigma-Aldrich™) with visualization under ultraviolet light (254 nm and 365 nm) or appropriate TLC staining. ^{1}H (400MHz), ^{13}C (101MHz) and ^{19}F (376 MHz) NMR spectra were recorded on ambient temperature using a Bruker-Avance 400 or Mercury 400. ^{1}H NMR spectra are reported in parts per million (ppm) downfield relative to $CDCl_3$ (7.26 ppm) and all ^{13}C NMR spectra are reported in ppm relative to $CDCl_3$ (77.0 ppm). NMR spectra uses the following abbreviations to describe the multiplicity: s = singlet, d = doublet, t = triplet, q = quartet, p = pentet, h = hextet, hept = heptet, m = multiplet, dd = double doublet, td = triple doublet, dt = double triplet, ddd = doublet of doublet of doublets, tt = triplet of triplets. Known products were characterized by comparing to the corresponding ^{1}H NMR and ^{13}C NMR from literature. High-resolution mass spectra (HRMS) were obtained by using electrospray ionization (ESI) on a Bruker Daltonics MicroToF spectrometer. GC analyses were performed on GC-MS combination (Shimadzu GC-2010 Plus coupled to a Mass Spectrometer; Shimadzu GCMS-QP2020 Ultra) with an auto sampler unit (AOC-20s, Shimadzu). Melting point were obtained by using BÜCHI Melting Point B-540. Purification of the crude products was conducted by automatic column chromatography using a Biotage® Isolera Four, with Biotage® SNAP KP-Sil 10 or 25 g flash chromatography cartridges. Flow reactions were carried out in an Omnifit column fixed on a R2/R4 Vapourtec equipment.

2. General procedure for the synthesis of *α*, *β*-unsaturated aromatic chlorides

2.1 General procedure for the synthesis of 1-chloroalkynes GP1

The preparation of the 1-chloroalkynes followed a reported procedure.[1]

In a dry flask, K_2CO_3 (1 mmol), *N*-Chlorosuccinimide (4 mmol) and Ag_2CO_3 (0.2 mmol) were added under argon atmosphere. Then 4 mL n-propanol and 2 mmol terminal alkyne were added to the reaction system, and the reaction mixture was stirred at 50 °C for 5 hours. After that, the mixture was allowed to cool to room temperature, 10 mL of brine was added to the mixture at 0 °C. The resulting mixture was extracted by diethyl ether for three times (20 mL × 3), the combined organic phase was washed with water (150 mL × 3) to remove the n-propanol, then dried over sodium sulfate. The solvent was removed by rotary evaporation and purified by column chromatography on silica gel to give the final product.

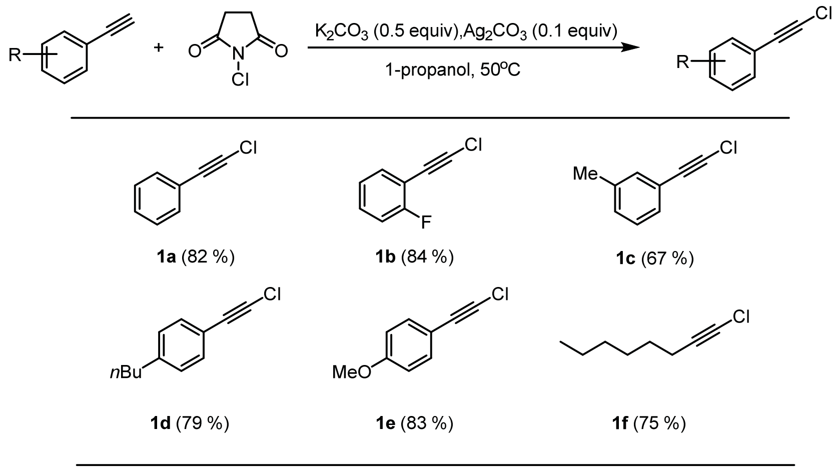

Scheme S1 Synthesis of 1-chloroalkynes.

2.2 General procedure for the synthesis of styrenyl chlorides GP2

Li_2CO_3 (1.25 mmol) was added to a mixture of cinnamic acid (5 mmol) and *N*-Chlorosuccinimide (10 mmol) in 30 mL acetonitrile/water (7:1 v/v) solution under argon. After stirring for 6 h at 60 ^oC, the mixture was cooled down to room temperature, 5 mL of water was added, the aqueous phase was extracted with ethyl acetate (20 mL × 3), and the combined organic layer was washed with brine (50 mL). The organic phase was dried over anhydrous $MgSO_4$. After evaporation, the mixture was subjected to column chromatography (silica gel, ethyl acetate/cyclohexane) to afford the desired product.

In the synthesis of the styryl chlorides, the reaction conditions were optimized with different lithium salt as additive. Compared to LiOAc, LiCl, $LiBF_4$, $LiCO_3$ showed the best yield in $MeCN/H_2O$ solution, when the temperature was increased to 60 ^oC. As such, a 99% yield was observed for the target compound via GCMS.

Then the reaction scope was expanded under the standard reaction conditions, various styryl chlorides were prepared from the corresponding cinnamic acid. The yield gave in the parentheses was isolated yield.

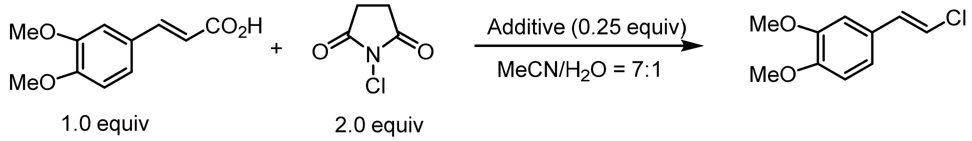

Entry	Additive	Solvent	T/^oC	Yield% [a]
1	LiOAc	MeCN/H_2O	20	53%
2	Li_2CO_3	MeCN/H_2O	20	70%
3	LiCl	MeCN/H_2O	20	60%
4	$LiBF_4$	MeCN/H_2O	20	26%
5	Li_2CO_3 1.2 equiv	MeCN/H_2O	20	20%
6	Li_2CO_3 1.2 equiv	MeCN	20	not observed
7	Li_2CO_3	MeCN	20	not observed
8	Li_2CO_3	MeCN/H_2O	60	99%

[a] Yield determined by GCMS analysis.

Scheme S2 Synthesis of styryl halides.

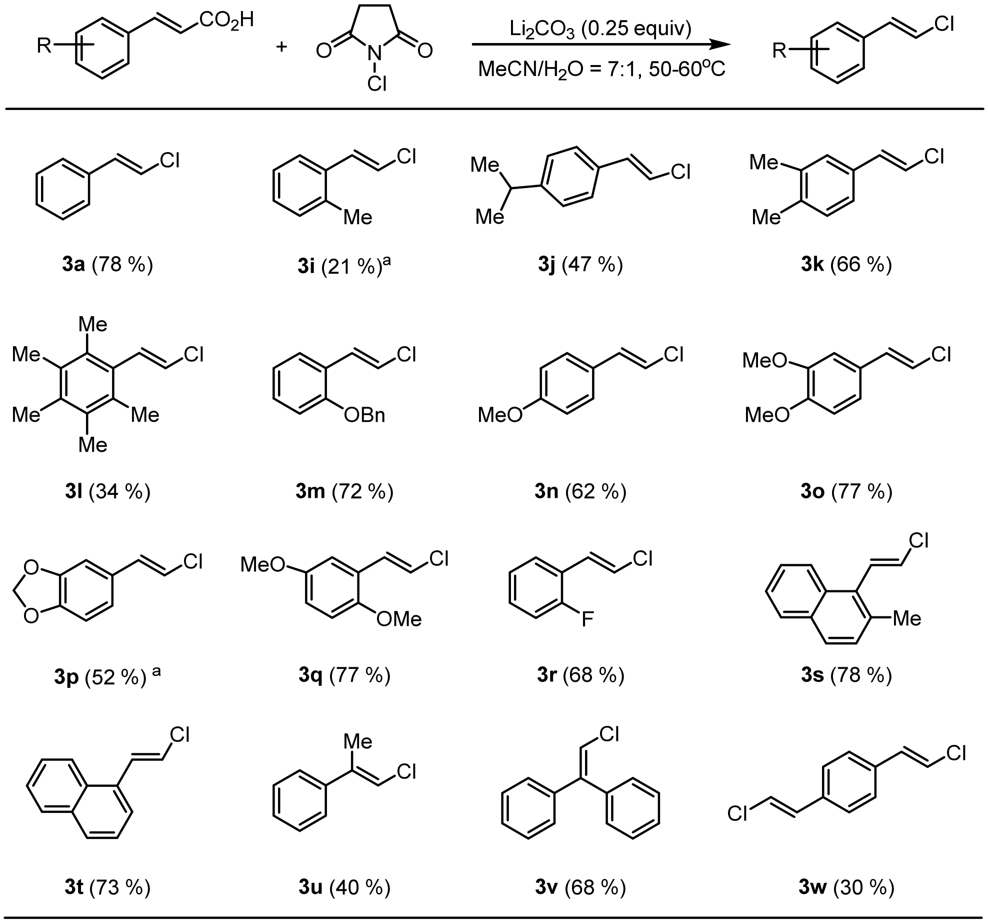

[a] LiOAc was used instead of Li_2CO_3.

Scheme S3 Synthesis of styryl halides.

2.3 General procedure for the synthesis of *(Z)*-(2-chlorovinyl)benzene GP3

The preparation of the *(Z)*-(2-chlorovinyl)benzene followed a literature procedure.[2]

(2, 2, 2-Trichloroethyl)benzene (1.0 mmol) was dissolved in THF (4 mL) and H_2O (1.0 mmol), which was added to a stirring suspension of $CrCl_2$ (1.0 mmol, 1 equiv; Sigma-Aldrich 99.9%) and Fe(0) powder (3.0 mmol, 3 equiv; Sigma-Aldrich 97%, 325 mesh) in THF (15 mL) under argon atmosphere. The reaction mixture was stirred at 50 °C for 18 h, then cooled to room temperature. Then the suspension was filtered through a short pad of silica gel, the filter cake was washed with ethyl ether. The combined filtrates were evaporated in vacuo and the residue was purified by column chromatography on silica gel to give the *(Z)*-(2-chlorovinyl)benzene (*cis:tans* = 91:9, 201 mg, 73 % yield).

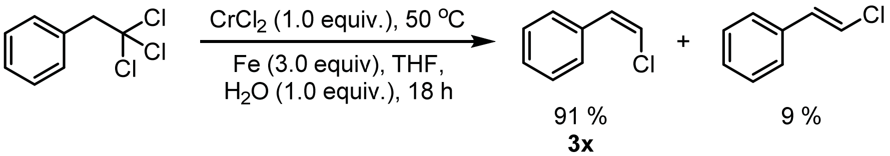

Scheme S4: Preparation of the *(Z)*-(2-chlorovinyl)benzene

3. General procedure for the Fe-catalyzed C–C cross-coupling

3.1 General procedure for the coupling of alkynyl chlorides with Grignard reagent in batch (GP4)

SIPr-HCl (4.3 mg, 2 mol%) was added to vial 1 containing a stirring bar, which is filled with argon and fitted with a septum. 0.6 mL of dry THF was added to the vial. Next, organomagnesium reagent solution (1.2 equiv.) was added to this vial. Vial 1 was left for stirring for 10 minutes. $Fe(acac)_3$ (1.7 mg, 1 mol%) and 1-chloroalkynes or styryl chlorides (0.5 mmol, 1 equiv) were added in vial 2. The vial was filled with N_2 and fitted with septum, afterwards 1.2 mL of THF was added. The solution in vial 2 was transferred to vial 1 at 0 ^{o}C and the mixed solution in vial 1 turned black. After stirring for 3 minutes at 0 ^{o}C, the mixture was quenched with 3.0 mL 1.0 M aqueous HCl solution and extracted with ethyl acetate. The organic layer was separated, washed with water and dried over anhydrous $MgSO_4$. After evaporation of solvent, the mixture was subjected to column chromatography (silica gel, ethyl acetate/cyclohexane) to afford pure product.

3.2 General procedure the coupling of alkenyl chlorides with Grignard reagent in batch (GP5)

$Fe(acac)_3$ (1.7 mg, 1 mol%) and styryl chlorides (0.5 mmol, 1 equiv) were added to a vial containing a stirring bar. The vial was filled with argon and fitted with a septum, afterwards 2.0 mL of THF was added. The organomagnesium reagent solution (1.2 equiv.) was added to this vial and the mixed solution turned black. Notably, the reaction mixture gets warm immediately. After stirring for 3 minutes at room temperature, the mixture was quenched with 3.0 mL 1.0 M aqueous HCl solution and extracted with ethyl acetate. The organic layer was separated, washed with water and dried over anhydrous $MgSO_4$. After evaporation of solvent, the mixture was subjected to column chromatography (silica gel, ethyl acetate/cyclohexane) to afford pure product.

4. General procedure for the synthesis of organomagnesium reagent in flow (GP6)

The preparation of the organomagnesium reagent in flow followed a literature procedure.[3]

Preparation of Mg column: a SolventPlusTM column (bore: 10 mm, length: 100 mm, AF; Omnifit, cat. no.006EZS-10-10-AF) is filled with 4g of magnesium (20-230 mesh, Sigma Aldrich Cat. N.: 254126) using a filter funnel. General flow procedure for magnesium activation and organomagnesium synthesis: 5 mL of DIBAL-H 1M in THF was passed through a 10 mm internal diameter Omni-fit column containing Mg (4 g) at 1 mL/min. After that, 5 mL solution of TMSCl 2.0 M and 1-bromo-2-chloroethane 0.24 M in 10 mL THF was passed through the column at 1 mL/min at room temperature. After the activation, a solution of aliphatic bromide in THF was passed through the column at 0.5 mL/min and at 40 ^{o}C. The solution was collected in a sealed vial under argon.

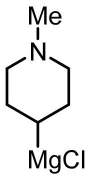

(1-methylpiperidin-4-yl)magnesium chloride: Prepared according to GP6 starting from 1.0 mL (8 mmol) of 4-chloro-1-methylpiperidine in 10 mL THF (0.73 M). Titration: An accurately weighed sample of 2-Hydroxybenzaldehyde phenylhydrazone (around 25 mg) is dissolved in 0.5 mL of THF under nitrogen, then the Grignard reagent is added drop by drop until the color of the solution turned dark red from pale yellow. The calculated concentration of the organomagnesium reagent was 0.50 M.

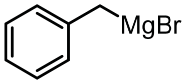

Benzylmagnesium bromide: Prepared according to GP6 starting from 0.6 mL (5 mmol) of benzyl bromide in 10 mL THF (0.47 M). Titration: An accurately weighed sample of 2-Hydroxybenzaldehyde phenylhydrazone (around 25 mg) is dissolved in 0.5 mL of THF under nitrogen, then the Grignard reagent is added drop by drop until the color of the solution turned dark red from pale yellow. The calculated concentration of the organomagnesium reagent was 0.40 M.

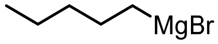

Pentylmagnesium bromide: Prepared according to GP6 starting from 0.93 mL (7.5 mmol) of 1-bromopentane in 15 mL THF (0.47 M). Titration: An accurately weighed sample of 2-Hydroxybenzaldehyde phenylhydrazone (around 25 mg) is dissolved in 0.5 mL of THF under nitrogen, then the Grignard reagent is added drop by drop until the color of the solution turned dark red from pale yellow. The calculated concentration of the organomagnesium reagent was 0.37 M.

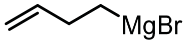

(but-3-en-1-yl)magnesium bromide: Prepared according to GP6 starting from 0.7 mL (7 mmol) of 4-bromobut-1-ene in 10 mL THF (0.65 M). Titration: An accurately weighed sample of 2-Hydroxybenzaldehyde phenylhydrazone (around 25 mg) is dissolved in 0.5 mL of THF under nitrogen, then the Grignard reagent is added drop by drop until the color of the solution turned dark red from pale yellow. The calculated concentration of the organomagnesium reagent was 0.42 M.

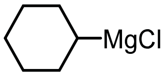

Cyclohexylmagnesium chloride: Prepared according to GP6 starting from 0.9 mL (7.5 mmol) of chlorocyclohexane in 15 mL THF (0.47 M). Titration: An accurately weighed sample of 2-Hydroxybenzaldehyde phenylhydrazone (around 25 mg) is dissolved in 0.5 mL of THF under nitrogen, then the Grignard reagent is added drop by drop until the color of the solution turned dark red from pale yellow. The calculated concentration of the organomagnesium reagent was 0.45 M.

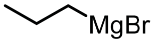

Propylmagnesium bromide: Prepared according to GP6 starting from 0.68 mL (7.5 mmol) of 1-bromopropane in 15 mL THF (0.48 M). Titration: An accurately weighed sample of 2-Hydroxybenzaldehyde phenylhydrazone (around 25 mg) is dissolved in 0.5 mL of THF under nitrogen, then the Grignard reagent is added drop by drop until the color of the solution

turned dark red from pale yellow. The calculated concentration of the organomagnesium reagent was 0.39 M.

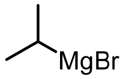

Isopropylmagnesium bromide: Prepared according to GP6 starting from 0.70 mL (7.5 mmol) of 2-bromopropane in 15 mL THF (0.48 M). Titration: An accurately weighed sample of 2-Hydroxybenzaldehyde phenylhydrazone (around 25 mg) is dissolved in 0.5 mL of THF under nitrogen, then the Grignard reagent is added drop by drop until the color of the solution turned dark red from pale yellow. The calculated concentration of the organomagnesium reagent was 0.36 M.

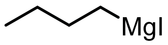

Butylmagnesium iodide: Prepared according to GP6 starting from 0.85 mL (7.5 mmol) of 1-iodobutane in 15 mL THF (0.47 M). Titration: An accurately weighed sample of 2-Hydroxybenzaldehyde phenylhydrazone (around 25 mg) is dissolved in 0.5 mL of THF under nitrogen, then the Grignard reagent is added drop by drop until the color of the solution turned dark red from pale yellow. The calculated concentration of the organomagnesium reagent was 0.46 M.

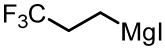

(3,3,3-trifluoropropyl)magnesium iodide: Prepared according to GP6 starting from 0.85 mL (7.5 mmol) of 1,1,1-trifluoro-3-iodopropane in 15 mL THF (0.47 M). Titration: An accurately weighed sample of 2-Hydroxybenzaldehyde phenylhydrazone (around 25 mg) is dissolved in 0.5 mL of THF under nitrogen, then the Grignard reagent is added drop by drop until the color of the solution turned dark red from pale yellow. The calculated concentration of the organomagnesium reagent was 0.44 M.

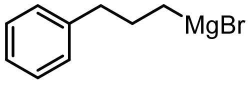

(3-phenylpropyl)magnesium bromide: Prepared according to GP6 starting from 1.1 mL (7.5 mmol) of (3-bromopropyl)benzene in 15 mL THF (0.47 M). Titration: An accurately weighed sample of 2-Hydroxybenzaldehyde phenylhydrazone (around 25 mg) is dissolved in 0.5 mL of THF under nitrogen, then the Grignard reagent is added drop by drop until the color of the solution turned dark red from pale yellow. The calculated concentration of the organomagnesium reagent was 0.29 M.

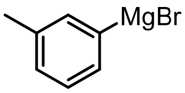

***m*-tolylmagnesium bromide**: Prepared according to GP6 starting from 0.91 mL (7.5 mmol) of 1-bromo-3-methylbenzene in 15 mL THF (0.47 M). Titration: An accurately weighed sample of 2-Hydroxybenzaldehyde phenylhydrazone (around 25 mg) is dissolved in 0.5 mL of THF under nitrogen, then the Grignard reagent is added drop by drop until the color of the solution turned dark red from pale yellow. The calculated concentration of the organomagnesium reagent was 0.39 M.

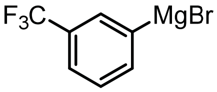

(3-(trifluoromethyl)phenyl)magnesium bromide: Prepared according to GP6 starting from 1.0 mL (7.5 mmol) of 1-bromo-3-(trifluoromethyl)benzene in 15 mL THF (0.47 M). Titration: An accurately weighed sample of 2-Hydroxybenzaldehyde phenylhydrazone (around 25 mg) is dissolved in 0.5 mL of THF under nitrogen, then the Grignard reagent is added

drop by drop until the color of the solution turned dark red from pale yellow. The calculated concentration of the organomagnesium reagent was 0.41 M.

5. General procedure for the telescope reaction GP7

5.1 General procedure for telescope reactions

The Grignard reagent was prepared following **GP6**, 5 mL of DIBAL-H 1M in THF was passed through a 10 mm internal diameter Omni-fit column containing Mg (4 g) at 1 mL/min at 40 °C. After that, 5 mL solution of TMSCl 2.0 M and 1-bromo-2-chloroethane 0.24 M in THF was passed through the column at 1 mL/min. After the activation step, a solution of the alkyl bromide/chloride (7.5 mmol) in 15 mL THF (0.5 M) was passed through the column at 40 °C with a flowrate of 0.5 mL/min. The first 3 mL of Grignard reagent was collected and titrate with 2-Hydroxybenzaldehyde phenylhydrazone. Based on the concentration of the Grignard reagent, THF was added to the vial of vinyl or alkynl chloride ($C_{Grignard\ reagent} = 1.2\ C_{Sub}$) with $Fe(acac)_3$ (1.7 mg, 1 mol%). The solution of the substrate and $Fe(acac)_3$ was charged to a syringe pump and mixed with Grignard reagent in a T-mixer, the combined solution was passed through a PFA (I.D. 0.75 mm) reactor at room temperature or ice-water bath. The effluent was quenched with 3.0 mL 1.0 M HCl solution and extracted with ethyl acetate (10 mL × 3). The organic layer was combined, washed with brine (30 mL) and dried over anhydrous $MgSO_4$. After evaporation, the crude was purified with chromatography (silica gel, 100 % cyclohexane) to afford the desired product.

5.2 Set up for the telescope reactions

Flow reactions were carried out in an Omnifit column fixed on a R2/R4 Vapourtec equipment. All microfluidic fittings were purchased from IDEX Health and Science. The syringes were connected to the capillary using 1/16 flat-bottom flangeless fittings. Syringe pump (Fusion 200 Classic) equipped with a 10 or 5 mL syringe was used to infuse the liquid reagents into a reactor coil fabricated from a high purity perfluoroalkoxyalkane (PFA) capillary tubing (1.0 mL, ID = 750 μm). The outlet of the microreactor led to the collection vial under argon. The detail of the assembling the reactor is shown in **Figure S1-2**.

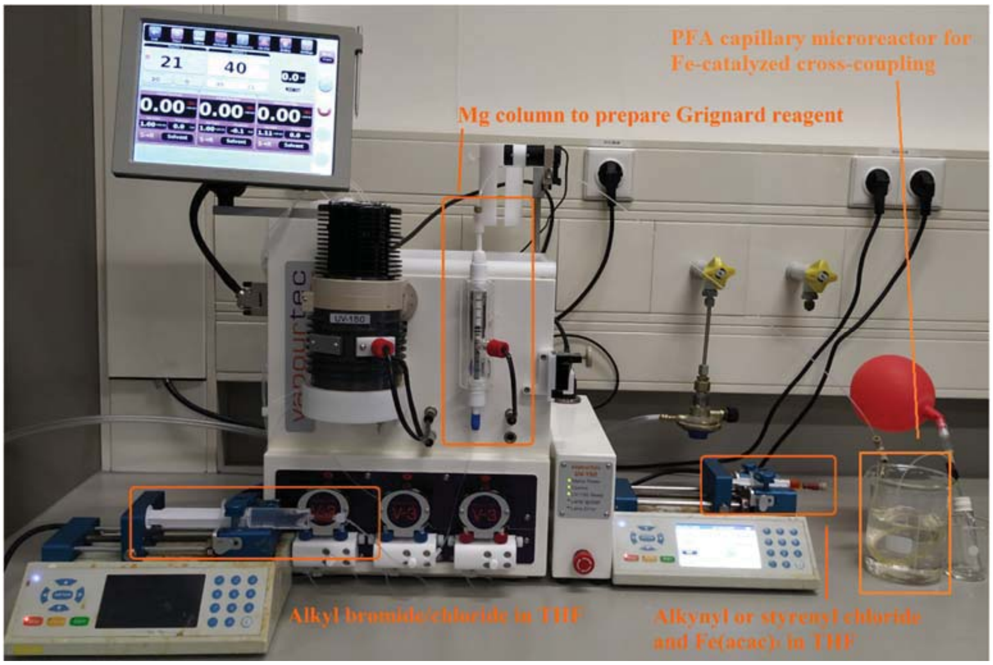

Figure S1: The overview of the flow set-up for telescoped Grignard synthesis and Fe-based cross-coupling.

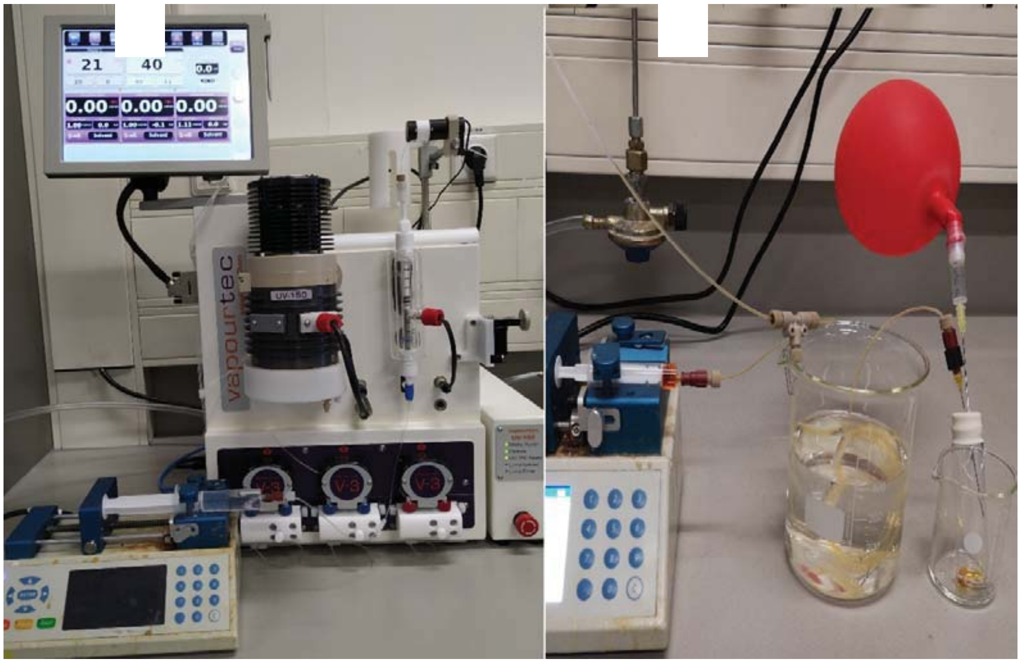

Figure S2: Detailed pictures of the telescoped flow setup: (A) A vapourtec reactor system was used for the Grignard reagent synthesis. (B) A capillary microreactor for the Fe-catalyzed cross-coupling reaction in flow.

6. Preliminary mechanistic study

To test whether this iron-catalyzed cross-coupling reaction is proceeding through a radical pathway, some preliminary experiments were carried out and all the data was collected by GC-MS (Scheme S4). Firstly, 1 equivalent of TEMPO was added to the reaction mixture of (2-chlorovinyl)benzene and cyclohexylmagnisum chloride under standard reaction condition, the product was observed with 18% of yield from GC. When the amount of TEMPO was decreased to 0.2 equivalent, the product was detected with 71% yield. In both cases, the adduct of TEMPO-Cyhexyl was detected with GC-MS. However, when we tried the reaction of TEMPO with the (2-chlorovinyl)benzene and cyclohexylmagnisum chloride separately. It shows that the substrate cannot react with TEMPO, but Grignard reagent and TEMPO could be coupled directly without iron catalyst. These results did not necessarily indicate that a radical pathway is involved in the reaction. The addition of TEMPO to the reaction of (chloroethynyl)benzene shows the same result.

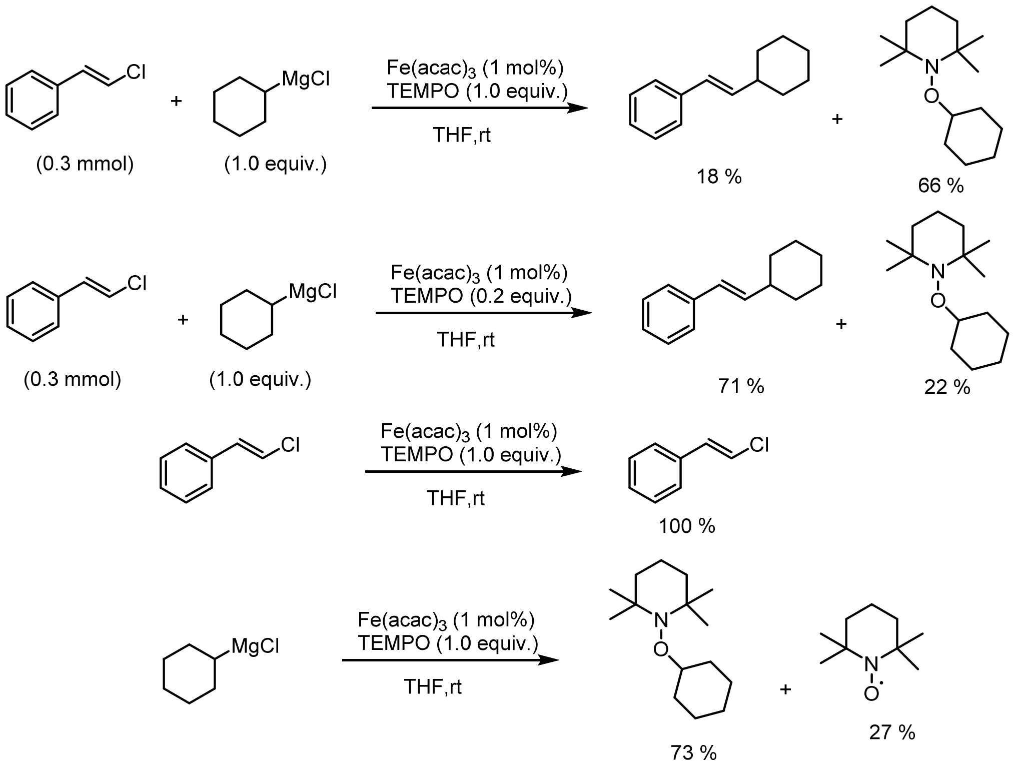

Scheme S5: Control experiments with TEMPO.

We observed that the *Z*-configuration substrate *(Z)*-(2-chlorovinyl)benzene (*cis:trans* = 91:9) could remain the configuration when conducted under the standard reaction condition, the ratio of the isomer is *cis:trans* = 88:12 compared with 91:9 (Scheme S5). This evidence demonstrated that radical pathways were not likely involved in the main pathway of the reaction.

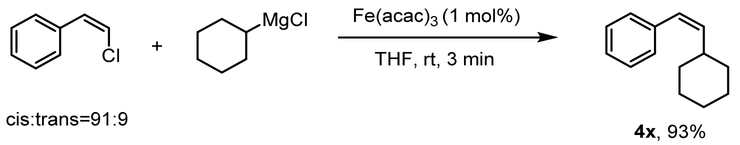

4x, 93%
cis:trans=88:12

Scheme S6 Reaction of *Z*-configuration substrate.

7. Limitations to the reaction scope

Some substrates and Grignard reagents proved much more challenging and provided either no product or only limited product as difficult to separate reaction mixture. An overview is given in Scheme S7.

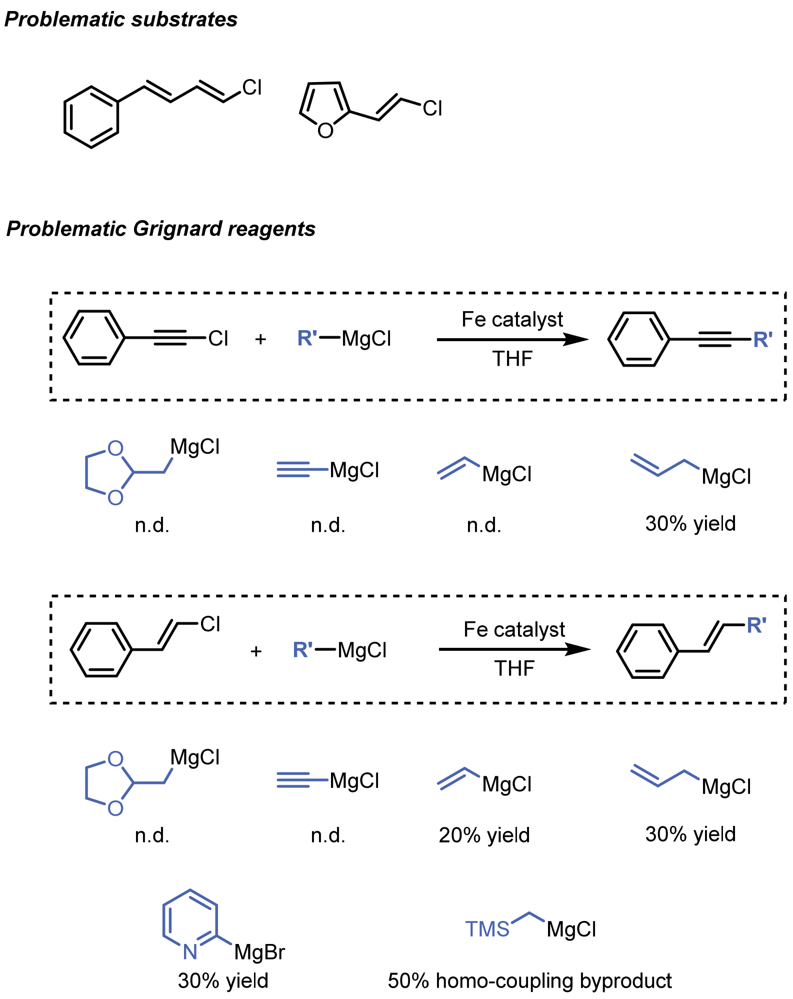

Scheme S7. Limitations in the Fe-catalyzed cross coupling of alkynyl and styrenly chlorides with alkyl Grignard reagents.

8. Characterization data

8.1 Characterization data of cross-coupling of the products

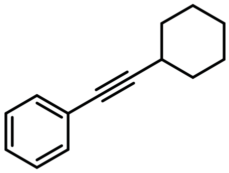

(cyclohexylethynyl)benzene (2a): 0.5 mmol (68 mg) **1a** was employed with general procedure GP4 and 88 mg (96 % yield) **2a** was obtained as pale yellow oil using column chromatography (eluent: 100% cyclohexane). **^{1}H NMR** (400 MHz, Chloroform-*d*) δ 7.42 – 7.39 (m, 2H), 7.31 – 7.27 (m, 3H), 2.63 – 2.57 (m, 1H), 1.91 – 1.88 (m, 2H), 1.79 – 1.74 (m, 2H), 1.59 – 1.51 (m, 3H), 1.39 –1.39 (m, 3H). **^{13}C NMR** (101 MHz, $CDCl_3$) δ 131.55, 128.12, 127.37, 124.12, 94.45, 80.48, 32.71, 29.66, 25.93, 24.91.

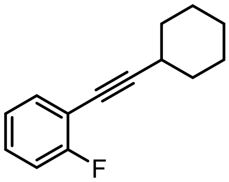

1-(cyclohexylethynyl)-2-fluorobenzene (2b): 0.5 mmol (77 mg) **1b** was employed with general procedure GP4 and 90 mg (89 % yield) **2b** was obtained as pale yellow oil using column chromatography (eluent: 100% cyclohexane). **^{1}H NMR** (400 MHz, Chloroform-*d*) δ 7.39 (td, J = 7.6, 1.8 Hz, 1H), 7.24 – 7.24 (m, 1H), 7.08 – 7.00 (m, 2H), 2.68 – 2.61 (m, 1H), 1.91 – 1.86 (m, 2H), 1.82 – 1.71 (m, 2H), 1.61 – 1.54 (m, 3H), 1.38 – 1.32 (m, 3H). **^{13}C NMR** (101 MHz, $CDCl_3$) δ 162.74 (d, J_{C-F} = 251.0 Hz), 133.52 (d, J_{C-F} = 1.5 Hz), 129.97 (d, J_{C-F} = 7.9 Hz), 123.69 (d, J_{C-F} = 3.8 Hz), 115.28 (d, J_{C-F} = 21.3 Hz), 112.57 (d, J_{C-F} = 15.9 Hz), 99.90 (d, J_{C-F} = 3.2 Hz), 73.82, 32.52, 29.79, 25.91, 24.78. **^{19}F NMR** (376 MHz, $CDCl_3$) δ **-**111.78 (q, J = 7.7, 7.2 Hz).

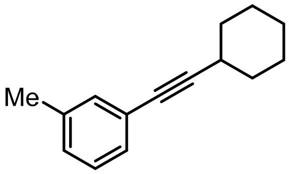

1-(cyclohexylethynyl)-3-methylbenzene (2c): 0.5 mmol (75 mg) **1c** was employed with general procedure GP4 and 93 mg (94 % yield) **2c** was obtained as pale yellow oil using column chromatography (eluent: 100% cyclohexane). **^{1}H NMR** (400 MHz, Chloroform-*d*) δ 7.23 – 7.14 (m, 3H), 7.07 (d, J = 7.4 Hz, 1H), 2.58 (tt, J = 9.0, 3.6 Hz, 1H), 2.31 (s, 3H), 1.88 – 1.84 (m, 2H), 1.78 – 1.73 (m, 2H), 1.58 – 1.49 (m, 3H), 1.38 – 1.32 (m, 3H). **^{13}C NMR** (101 MHz, $CDCl_3$) δ 137.74, 132.18, 128.58, 128.26, 128.02, 123.89, 94.06, 80.60, 32.74, 30.92, 29.64, 25.94, 24.90, 21.18.

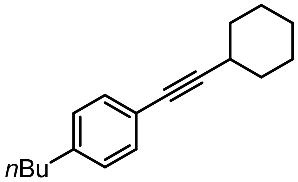

1-butyl-4-(cyclohexylethynyl)benzene (2d): 0.5 mmol (96 mg) **1d** was employed with general procedure GP4 and 112 mg (93 % yield) **2d** was obtained as pale yellow oil using column chromatography (eluent: 100% cyclohexane). **^{1}H NMR** (400 MHz, Chloroform-*d*) δ 7.30 (d, J = 8.1 Hz, 2H), 7.08 (d, J = 8.2 Hz, 2H), 2.64 – 2.55 (m, 3H), 1.89 – 1.85 (m, 2H), 1.77 – 1.72 (m, 2H), 1.61 – 1.51 (m, 5H), 1.36 – 1.30 (m, 5H), 0.91 (t, J = 7.3 Hz, 3H). **^{13}C NMR** (101 MHz, $CDCl_3$) δ 142.34, 131.41, 128.24, 121.20, 93.64, 80.52, 35.48, 33.43, 32.77, 30.92, 29.66, 25.94, 24.90, 22.25, 13.91.

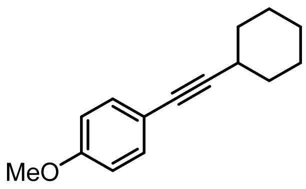

1-(cyclohexylethynyl)-4-methoxybenzene (2e): 0.5 mmol (83 mg) **1e** was employed with general procedure GP4 and 103 mg (96 % yield) **2e** was obtained as pale yellow oil using column chromatography (eluent: 100% cyclohexane). **^{1}H NMR** (400 MHz, Chloroform-*d*) δ 7.35 – 7.30 (m, 2H), 6.83 – 6.78 (m, 2H), 3.80 (s, 3H), 2.59 – 2.53 (m, 1H), 1.92 – 1.83 (m, 2H), 1.78 – 1.73 (m, 2H), 1.58 – 1.49 (m, 3H), 1.37 – 1.31 (m, 3H). **^{13}C NMR** (101 MHz, $CDCl_3$) δ 158.91, 132.85, 116.28, 113.73, 92.83, 80.12, 55.23, 32.83, 30.91, 29.68, 25.94, 24.95.

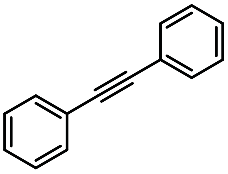

1,2-diphenylethyne (2f): 0.5 mmol (68 mg) **1a** was employed with general procedure GP4 and 77 mg (87 % yield) **2l** was obtained as white solid using column chromatography (eluent: 100% cyclohexane). Melting point: 53.9 ^{o}C. **^{1}H NMR** (400 MHz, Chloroform-*d*) δ 7.57 – 7.51 (m, 4H), 7.38 – 7.31 (m, 6H). **^{13}C NMR** (101 MHz, $CDCl_3$) δ 131.59, 128.32, 128.23, 123.26, 89.35.

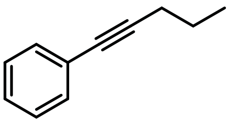

pent-1-yn-1-ylbenzene (2g): 0.5 mmol (68 mg) **1a** was employed with general procedure GP4 and 67 mg (93 % yield) **2g** was obtained as colorless oil using column chromatography (eluent: 100% cyclohexane). **^{1}H NMR** (400 MHz, Chloroform-*d*) δ 7.43 – 7.38 (m, 2H), 7.30 – 7.25 (m, 3H), 2.40 (t, *J* = 7.0 Hz, 2H), 1.65 (h, *J* = 7.3 Hz, 3H), 1.06 (t, *J* = 7.4 Hz, 3H). **^{13}C NMR** (101 MHz, $CDCl_3$) δ 131.52, 128.15, 127.44, 124.07, 90.24, 80.68, 22.22, 21.39, 13.54.

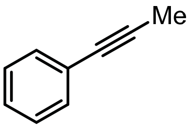

prop-1-yn-1-ylbenzene (2h): 0.5 mmol (68 mg) **1a** was employed with general procedure GP4 and 54 mg (93 % yield) **2h** was obtained as colorless oil using column chromatography (eluent: 100% cyclohexane). **^{1}H NMR** (400 MHz, Chloroform-*d*) δ 7.42 – 7.39 (m, 2H), 7.31 – 7.26 (m, 3H), 2.06 (s, 3H). **^{13}C NMR** (101 MHz, $CDCl_3$) δ 131.46, 128.17, 127.48, 124.00, 85.77, 79.70, 4.29.

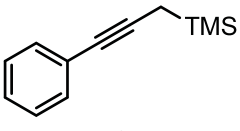

trimethyl(3-phenylprop-2-yn-1-yl)silane (2i): 0.5 mmol (68 mg) **1a** was employed with general procedure GP4 and 76 mg (81 % yield) **2i** was obtained as colorless oil using column chromatography (eluent: 100% cyclohexane). **^{1}H NMR** (400 MHz, Chloroform-*d*) δ 7.37 – 7.03 (m, 5H), 1.61 (s, 2H), 0.08 (s, 9H). **^{13}C NMR** (101 MHz, $CDCl_3$) δ 131.40, 128.12, 126.99, 124.83, 88.44, 79.58, 7.96, -1.96.

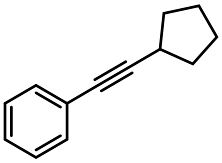

(cyclopentylethynyl)benzene (2j): 0.5 mmol (68 mg) **1a** was employed with general procedure GP4 and 73 mg (86 % yield) **2j** was obtained as colorless oil using column chromatography (eluent: 100% cyclohexane). **^{1}H NMR** (400 MHz, Chloroform-*d*) δ 7.40 – 7.37 (m, 2H), 7.27 – 7.24 (m, 3H), 2.82 (p, *J* = 7.5 Hz, 1H), 2.04 – 1.96 (m, 2H), 1.80 – 1.67 (m, 4H), 1.62 – 1.57 (m, 2H). **^{13}C NMR** (101 MHz, $CDCl_3$) δ 131.51, 128.12, 127.35, 124.14, 94.59, 80.03, 33.92, 30.78, 25.05.

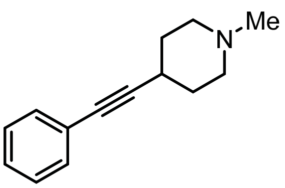

1-methyl-4-(phenylethynyl)piperidine (2k): 0.5 mmol (69 mg) **1a** was employed with general procedure GP4. The reaction mixture was quenched with 3.0 mL 1.0 M aqueous HCl solution. The acidic aqueous layer was basified with saturated K_2CO_3 aqueous solution and extracted with diethyl ether, and the combined organic layers were dried (anhydrous $MgSO_4$). After filtration, the solvent was evaporated in vacuo and 99 mg (99 % yield) **2k** was obtained as orange oil. **^{1}H NMR** (400 MHz, Chloroform-*d*) δ 7.43 – 7.38 (m, 2H), 7.31 – 7.24 (m, 3H), 2.79 – 2.66 (m, 2H), 2.66 – 2.54 (m, 1H), 2.29 (s, 3H), 2.25 – 2.14 (m, 2H), 1.99 – 1.89 (m, 2H), 1.83 – 1.72 (m, 2H). **^{13}C NMR** (101 MHz, $CDCl_3$) δ 131.55, 128.16, 127.59, 123.78, 92.74, 81.42, 81.35, 54.21, 46.52, 31.81, 27.03. **HRMS** (ESI) calcd for $C_{14}H_{17}NH$ [M]+: 200.1434; found: 200.1447. **MS (EI, 70 ev)**: *m/z* (relative intensity) = 199 ($M^{+\bullet}$, 84), 198 (100), 170 (16), 142 (61), 115 (29), 108 (39), 96 (47), 82 (35), 70 (46), 57 (35), 42 (82).

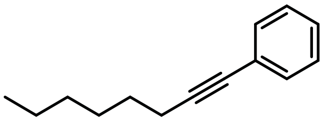

oct-1-yn-1-ylbenzene (2l): 0.5 mmol (72 mg) **1f** was employed with general procedure GP4 and 84 mg (90 % yield) **2f** was obtained as pale yellow oil using column chromatography (eluent: 100% cyclohexane). **^{1}H NMR** (400 MHz, Chloroform-*d*) δ 7.42 – 7.36 (m, 2H), 7.31 – 7.26 (m, 3H), 2.40 (t, *J* = 7.1 Hz, 2H), 1.64 – 1.57 (m, 2H), 1.49 – 1.42 (m, 2H), 1.34 – 1.30 (m, 4H), 0.91 (t, *J* = 6.9 Hz, 3H). **^{13}C NMR** (101 MHz, $CDCl_3$) δ 131.52, 128.15, 127.42, 124.09, 90.47, 80.52, 31.37, 28.73, 28.60, 22.57, 19.41, 14.06, 1.02.

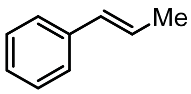

***(E)*-prop-1-en-1-ylbenzene (4a)**: 0.5 mmol (69 mg) **3a** was employed with general procedure GP5 and 53 mg (90 % yield) **4a** was obtained as colorless oil using column chromatography (eluent: 100% cyclohexane). **^{1}H NMR** (400 MHz, Chloroform-*d*) δ 7.34 – 7.27 (m, 4H), 7.22 – 7.16 (m, 1H), 6.42 (dd, *J* = 15.7, 1.4 Hz, 1H), 6.24 (dq, *J* = 15.7, 6.5 Hz, 1H), 1.89 (dd, *J* = 6.5, 1.5 Hz, 3H). **^{13}C NMR** (101 MHz, $CDCl_3$) δ 137.92, 131.00, 128.45, 126.71, 125.79, 125.68, 18.48.

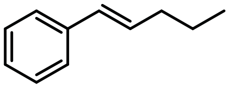

***(E)*-pent-1-en-1-ylbenzene (4b)**: 0.5 mmol (69 mg) **3a** was employed with general procedure GP5 and 71 mg (97 % yield) **4b** was obtained as colorless oil using column chromatography (eluent: 100% cyclohexane). **^{1}H NMR** (400 MHz, Chloroform-*d*) δ 7.39 – 7.30 (m, 4H), 7.24 – 7.20 (m, 1H), 6.42 (d, *J* = 15.8 Hz, 1H), 6.26 (dt, *J* = 15.8, 6.9 Hz, 1H), 2.25 – 2.20 (m, 2H), 1.58 – 1.47 (m, 2H), 0.99 (t, *J* = 7.4 Hz, 3H). **^{13}C NMR** (101 MHz, $CDCl_3$) δ 137.92, 130.94, 129.87, 128.43, 126.72, 125.89, 35.11, 22.54, 13.73.

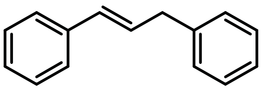

***(E)*-prop-1-ene-1,3-diyldibenzene (4c)**: 0.5 mmol (69 mg) **3a** was employed with general procedure GP5 and 93 mg (96 % yield) **4c** was obtained as colorless oil using column chromatography (eluent: 100% cyclohexane). **^{1}H NMR** (400 MHz, Chloroform-*d*) δ 7.38 – 7.26 (m, 7H), 7.25 – 7.19 (m, 3H), 6.47 (d, *J* = 15.8 Hz, 1H),

6.37 (dt, *J* = 15.7, 6.6 Hz, 1H), 3.57 (d, *J* = 6.6 Hz, 2H). **^{13}C NMR** (101 MHz, $CDCl_3$) δ 140.15, 137.46, 131.05, 129.21, 128.65, 128.48, 127.08, 126.16, 126.10, 39.34.

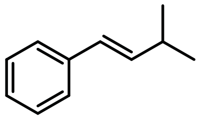

***(E)*-(3-methylbut-1-en-1-yl)benzene (4d)**: 0.5 mmol (69 mg) **3a** was employed with general procedure GP5 and 68 mg (b:l = 94:6, 93 % yield) **4d** was obtained as colorless oil using column chromatography (eluent: 100% cyclohexane). **^{1}H NMR** (400 MHz, Chloroform-*d*) δ 7.36 (d, *J* = 7.3 Hz, 2H), 7.29 (t, *J* = 7.6 Hz, 2H), 7.19 (t, *J* = 7.2 Hz, 1H), 6.35 (d, *J* = 16.1 Hz, 1H), 6.20 (dd, *J* = 15.9, 6.7 Hz, 1H), 2.52 – 2.43 (m, 1H), 1.10 (d, *J* = 6.7 Hz, 6H). **^{13}C NMR** (101 MHz, $CDCl_3$) δ 138.00, 137.94, 128.44, 126.80, 126.73, 125.94, 31.52, 22.44.

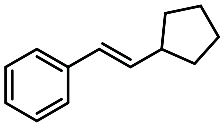

***(E)*-(2-cyclopentylvinyl)benzene (4e)**: 0.5 mmol (69 mg) **3a** was employed with general procedure GP4 and 77 mg (90 % yield) **4e** was obtained as colorless oil using column chromatography (eluent: 100% cyclohexane). **^{1}H NMR** (400 MHz, Chloroform-*d*) δ 7.36 (d, *J* = 7.3 Hz, 2H), 7.29 (t, *J* = 7.6 Hz, 2H), 7.19 (t, *J* = 7.2 Hz, 1H), 6.38 (d, *J* = 15.8 Hz, 1H), 6.22 (dd, *J* = 15.8, 7.7 Hz, 1H), 2.56 – 2.66 (m, 1H), 1.91 – 1.84 (m, 2H), 1.76 – 1.61 (m, 4H), 1.45 – 1.37 (m, 2H). **^{13}C NMR** (101 MHz, $CDCl_3$) δ 137.92, 135.69, 128.43, 127.82, 126.68, 125.89, 43.80, 33.21, 25.23.

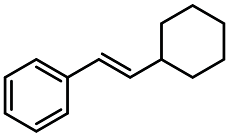

***(E)*-(2-cyclohexylvinyl)benzene (4f)**: 0.5 mmol (69 mg) **3a** was employed with general procedure GP5 and 84 mg (90 % yield) **4f** was obtained as colorless oil using column chromatography (eluent: 100% cyclohexane). **^{1}H NMR** (400 MHz, Chloroform-*d*) δ 7.34 (d, *J* = 7.2 Hz, 2H), 7.29 (d, *J* = 7.4 Hz, 2H), 7.18 (t, *J* = 7.2, 1H), 6.35 (d, *J* = 16.0 Hz, 1H), 6.18 (dd, *J* = 16.0, 6.9 Hz, 1H), 2.16 – 2.10 (m, 1H), 1.83 – 1.67 (m, 5H), 1.37 – 1.14 (m, 5H). **^{13}C NMR** (101 MHz, $CDCl_3$) δ 138.11, 136.87, 128.49, 127.29, 126.77, 125.99, 41.23, 33.03, 26.25, 26.13.

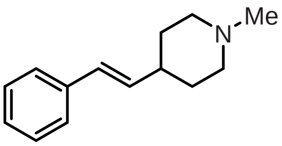

***(E)*-1-methyl-4-styrylpiperidine (4g)**: 0.5 mmol (69 mg) **3a** was employed with general procedure GP5. The reaction mixture was quenched with 3.0 mL 1.0 M aqueous HCl solution. The acidic aqueous layer was basified with saturated K_2CO_3 aqueous solution and extracted with diethyl ether, and the combined organic layers were dried (anhydrous $MgSO_4$). After filtration, the solvent was evaporated in vacuo and 97 mg (96 % yield) **4g** was obtained as orange oil. **^{1}H NMR** (400 MHz, Chloroform-d) δ 7.39 – 7.32 (m, 2H), 7.31 – 7.26 (m, 2H), 7.16 – 7.22 (m, 1H), 6.38 (d, *J* = 16.0 Hz, 1H), 6.17 (dd, *J* = 16.0, 6.9 Hz, 1H), 2.95 – 2.86 (m, 1H), 2.31 (s, 3H), 2.14 – 2.07 (m, 1H), 2.07 – 1.97 (m, 2H), 1.82 – 1.74 (m, 2H), 1.57 (qd, *J* = 12.9, 12.4, 3.7 Hz, 2H). **^{13}C NMR** (101 MHz, CDCl3) δ 137.64, 134.95, 128.47, 128.23, 126.96, 125.98, 55.57, 46.40, 38.66, 32.02.

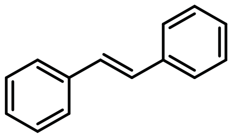

***(E)*-1,2-diphenylethene (4h)**: 0.5 mmol (69 mg) **3a** was employed with general procedure GP5 and 87 mg (97 % yield) **4h** was obtained as colorless oil using column chromatography (eluent: 100% cyclohexane). **^{1}H NMR** (400 MHz, Chloroform-*d*) δ 7.57 – 7.55 (m, 4H), 7.42 – 7.39 (m, 4H), 7.34 – 7.27 (m, 2H), 7.16 (s, 2H). **^{13}C NMR** (101 MHz, $CDCl_3$) δ 137.29, 128.66, 128.64, 127.58, 126.48.

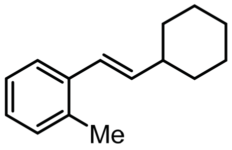

***(E)*-1-(2-cyclohexylvinyl)-2-methylbenzene (4i)**: 0.5 mmol (76 mg) **3i** was employed with general procedure GP5 and 97 mg (97 % yield) **4i** was obtained as colorless oil using column chromatography (eluent: 100% cyclohexane). **^{1}H NMR** (400 MHz, Chloroform-*d*) δ 7.46 (d, *J* = 7.0 Hz, 1H), 7.21 – 7.13 (m, 3H), 6.59 (d, *J* = 15.8 Hz, 1H), 6.09 (dd, *J* = 15.8, 7.0 Hz, 1H), 2.38 (s, 3H), 2.24 – 2.17 (m, 1H), 1.89 – 1.72 (m, 5H), 1.43 – 1.20 (m, 5H). **^{13}C NMR** (101 MHz, $CDCl_3$) δ 138.22, 137.12, 134.91, 130.09, 126.65, 125.95, 125.31, 125.01, 41.45, 33.09, 26.18, 26.05, 19.80.

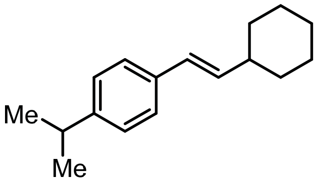

***(E)*-1-(2-cyclohexylvinyl)-4-isopropylbenzene (4j)**: 0.5 mmol (90 mg) **3j** was employed with general procedure GP5 and 106 mg (93 % yield) **4j** was obtained as colorless oil using column chromatography (eluent: 100% cyclohexane). **^{1}H NMR** (400 MHz, Chloroform-*d*) δ 7.34 (d, *J* = 8.1 Hz, 2H), 7.21 (d, *J* = 8.1 Hz, 2H), 6.38 (d, *J* = 16.0 Hz, 1H), 6.19 (dd, *J* = 16.0, 7.0 Hz, 1H), 2.97 – 2.90 (m, 1H), 2.21 – 2.14 (m, 1H), 1.87 – 1.72 (m, 5H), 1.36 – 1.24 (m, 11H). **^{13}C NMR** (101 MHz, $CDCl_3$) δ 147.49, 135.99, 135.69, 127.00, 126.49, 125.86, 41.16, 33.81, 33.03, 26.19, 26.06, 23.98.

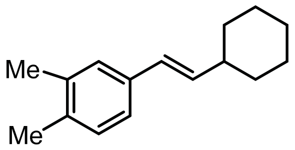

***(E)*-4-(2-cyclohexylvinyl)-1,2-dimethylbenzene (4k)**: 0.5 mmol (83 mg) **3k** was employed with general procedure GP5 and 104 mg (97 % yield) **4k** was obtained as colorless oil using column chromatography (eluent: 100% cyclohexane). **^{1}H NMR** (400 MHz, Chloroform-*d*) δ 7.20 (s, 1H), 7.13 (q, *J* = 7.8 Hz, 2H), 6.36 (d, *J* = 16.0 Hz, 1H), 6.18 (dd, *J* = 16.0, 6.9 Hz, 1H), 2.31 (s, 3H), 2.30 (s, 3H), 2.21 – 2.14 (m, 1H), 1.88 – 1.73 (m, 5H), 1.44 – 1.20 (m, 5H). **^{13}C NMR** (101 MHz, $CDCl_3$) δ 136.39, 135.70, 135.62, 135.01, 129.69, 127.15, 127.08, 123.39, 41.13, 33.03, 26.20, 26.07, 19.74, 19.40. **MS (EI, 70 ev)**: *m/z* (relative intensity) = 214 ($M^{+\bullet}$, 81), 199 (28), 171 (14), 157 (35), 143 (24), 132 (100), 119 (38), 108 (13), 91 (12), 41 (6).

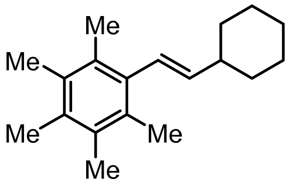

***(E)*-1-(2-cyclohexylvinyl)-2,3,4,5,6-pentamethylbenzene (4l)**: 0.5 mmol (104 mg) **3l** was employed with general procedure GP5 and 122 mg (95 % yield) **4l** was obtained as white solid using column chromatography (eluent: 100% cyclohexane). Melting point: 89.6 °C. **^{1}H NMR** (400 MHz, Chloroform-*d*) δ 6.31 (d, *J* = 16.3 Hz, 1H), 5.43 (dd, *J* = 16.3, 6.9

Hz, 1H), 2.25 – 2.17 (m, 15H), 1.87 – 1.67 (m, 5H), 1.40 – 1.17 (m, 5H). **^{13}C NMR** (101 MHz, $CDCl_3$) δ 141.14, 136.15, 133.04, 132.19, 131.50, 126.72, 41.38, 33.02, 26.25, 26.04, 17.83, 16.71, 16.59. **MS (EI, 70 ev)**: *m/z* (relative intensity) = 256 ($M^{+\bullet}$, 93), 241 (100), 185 (25), 173 (38), 160 (54), 148 (56), 133 (17), 107 (10), 79 (8), 55 (6), 41 (7).

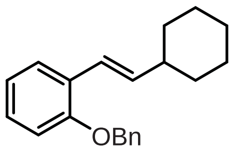

***(E)*-1-(benzyloxy)-2-(2-cyclohexylvinyl)benzene (4m)**: 0.5 mmol (122 mg) **3m** was employed with general procedure GP5 and 137 mg (94 % yield) **4m** was obtained as pale yellow oil using column chromatography (eluent: 100% cyclohexane). **^{1}H NMR** (400 MHz, Chloroform-d) δ 7.53 – 7.49 (m, 3H), 7.44 (t, *J* = 7.4 Hz, 2H), 7.37 (t, *J* = 7.2 Hz, 1H), 7.21 – 7.17 (m, 1H), 6.99 – 6.93 (m, 2H), 6.83 (d, *J* = 16.1 Hz, 1H), 6.25 (dd, *J* = 16.1, 7.1 Hz, 1H), 5.14 (s, 2H), 2.24 – 2.17 (m, 1H), 1.88 – 1.71 (m, 5H), 1.42 – 1.20 (m, 5H). **^{13}C NMR** (101 MHz, CDCl3) δ 155.46, 137.53, 137.35, 128.45, 127.69, 127.62, 127.54, 127.15, 126.30, 121.82, 120.97, 112.59, 70.28, 41.54, 33.05, 26.18, 26.02. **HRMS** (ESI) calcd for $C_{21}H_{24}ONa$ [M]+: 315.1719; found: 315.1718. **MS (EI, 70 ev)**: *m/z* (relative intensity) = 292 (M+•, 14), 201 (54), 157 (6), 119 (21), 107 (15), 91 (100), 55 (12), 41 (5).

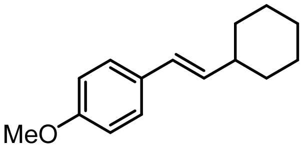

***(E)*-1-(2-cyclohexylvinyl)-4-methoxybenzene (4n)**: 0.5 mmol (84 mg) **3n** was employed with general procedure GP5 and 98 mg (91 % yield) **4n** was obtained as pale yellow oil using column chromatography (eluent: ethyl acetate/cyclohexane = 1:20). **^{1}H NMR** (400 MHz, Chloroform-d) δ 7.28 (d, *J* = 8.7 Hz, 2H), 6.83 (d, *J* = 8.7 Hz, 2H), 6.29 (d, *J* = 16.0 Hz, 1H), 6.04 (dd, *J* = 16.0, 7.0 Hz, 1H), 3.80 (s, 3H), 2.14 – 2.06 (m, 1H), 1.81 – 1.66 (m, 5H), 1.36 – 1.12 (m, 5H). **^{13}C NMR** (101 MHz, CDCl3) δ 158.57, 134.78, 130.87, 126.96, 126.50, 113.87, 55.28, 41.11, 33.08, 26.19, 26.08.

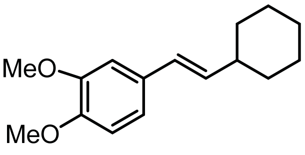

***(E)*-4-(2-cyclohexylvinyl)-1,2-dimethoxybenzene (4o):** 0.5 mmol (99 mg) **3o** was employed with general procedure GP5 and 116 mg (94 % yield) **4o** was obtained as pale yellow oil using column chromatography (eluent: ethyl acetate/cyclohexane = 1:20). **^{1}H NMR** (400 MHz, Chloroform-*d*) δ 6.92 – 6.78 (m, 3H), 6.28 (d, *J* = 15.9 Hz, 1H), 6.04 (dd, *J* = 15.9, 6.9 Hz, 1H), 3.90 (s, 3H), 3.86 (s, 3H), 2.14-2.07 (m, 1H), 1.86 – 1.67 (m, 5H), 1.37 – 1.14 (m, 5H). **^{13}C NMR** (101 MHz, $CDCl_3$) δ 148.92, 148.11, 134.93, 131.14, 126.78, 118.78, 111.11, 108.40, 55.86, 55.72, 41.06, 33.03, 26.14, 26.03.

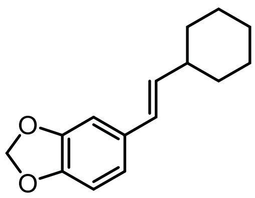

***(E)*-5-(2-cyclohexylvinyl)benzo[*d*][1,3]dioxole (4p)**: 0.5 mmol (91 mg) **3p** was employed with general procedure GP5 and 102 mg (89 % yield) **4p** was obtained as pale yellow oil using column chromatography (eluent: ethyl acetate/cyclohexane = 1:20). **^{1}H NMR** (400 MHz, Chloroform-*d*) δ 6.95 – 6.90 (m, 1H), 6.81 – 6.72 (m, 2H), 6.27 (d, *J* = 15.9 Hz, 1H), 6.02 (dd, *J* = 15.9, 7.0 Hz, 1H), 5.93 (s, 2H), 2.15 – 2.07 (m, 1H), 1.82 – 1.68 (m, 5H), 1.38 – 1.14 (m, 5H). **^{13}C NMR**

(101 MHz, $CDCl_3$) δ 147.87, 146.45, 135.10, 132.56, 126.74, 120.19, 108.13, 105.34, 100.83, 41.01, 33.00, 26.14, 26.03.

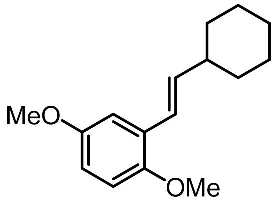

***(E)*-2-(2-cyclohexylvinyl)-1,4-dimethoxybenzene (4q):** 0.5 mmol (99 mg) **3q** was employed with general procedure GP5 and 111 mg (90 % yield) **4q** was obtained as pale yellow oil using column chromatography (eluent: ethyl acetate/cyclohexane = 1:20). **^{1}H NMR** (400 MHz, Chloroform-*d*) δ 7.01 (d, *J* = 2.9 Hz, 1H), 6.79 (d, *J* = 8.9 Hz, 1H), 6.73 (dd, *J* = 8.9, 2.9 Hz, 1H), 6.67 (d, *J* = 16.1 Hz, 1H), 6.16 (dd, *J* = 16.1, 7.0 Hz, 1H), 3.80 (s, 3H), 3.79 (s, 3H), 2.16 (m, 1H), 1.85 – 1.70 (m, 5H), 1.38 – 1.16 (m, 5H). **^{13}C NMR** (101 MHz, $CDCl_3$) δ 153.71, 150.81, 137.69, 127.98, 121.55, 112.61, 112.19, 111.61, 56.22, 55.70, 41.49, 33.00, 26.17, 26.05. **HRMS** (ESI) calcd for $C_{16}H_{22}O_2Na$ [M]+: 269.1512; found: 269.1529. **MS (EI, 70 ev)**: *m/z* (relative intensity) = 246 ($M^{+\bullet}$, 100), 215 (8), 189 (11), 164 (37), 151 (100), 121 (27), 91(15), 55 (10), 41(8).

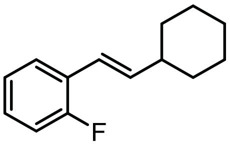

***(E)*-1-(2-cyclohexylvinyl)-2-fluorobenzene (4r)**: 0.5 mmol (78 mg) **3r** was employed with general procedure GP5 and 93 mg (91 % yield) **4r** was obtained as colorless oil using column chromatography (eluent: 100% cyclohexane). **^{1}H NMR** (400 MHz, Chloroform-*d*) δ 7.44 (td, *J* = 7.7, 1.7 Hz, 1H), 7.19 – 7.11 (m, 1H), 7.10 – 6.96 (m, 2H), 6.51 (d, *J* = 16.2 Hz, 1H), 6.25 (dd, *J* = 16.1, 7.0 Hz, 1H), 2.20 – 2.12 (m, 1H), 1.87 – 1.64 (m, 5H), 1.39 – 1.13 (m, 5H). **^{13}C NMR** (101 MHz, $CDCl_3$) δ 169.96 (d, J_{C-F} = 248.8 Hz), 139.34 (d, J_{C-F} = 4.1 Hz), 127.85 (d, J_{C-F} = 8.3 Hz), 126.84 (d, J_{C-F} = 4.1 Hz), 125.74 (d, J_{C-F} = 12.4 Hz), 123.90 (d, J_{C-F} = 3.5 Hz), 119.54 (d, J_{C-F} = 3.9 Hz), 115.55 (d, J_{C-F} = 22.3 Hz), 41.53, 32.84, 26.14, 26.00. **^{19}F NMR** (376 MHz, $CDCl_3$) δ **-**119.08 (ddd, *J* = 10.9, 7.7, 5.2 Hz).

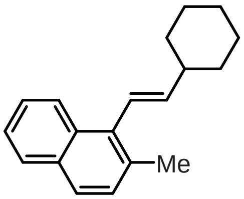

***(E)*-1-(2-cyclohexylvinyl)-2-methylnaphthalene (4s)**: 0.5 mmol (101 mg) **3s** was employed with general procedure GP5 and 114 mg (91 % yield) **4s** was obtained as colorless oil using column chromatography (eluent: 100% cyclohexane). **^{1}H NMR** (400 MHz, Chloroform-*d*) δ 8.11 – 8.09 (m, 1H), 7.80 – 7.77 (m, 1H), 7.64 (d, *J* = 8.4 Hz, 1H), 7.45 – 7.37 (m, 2H), 7.32 (d, *J* = 8.4 Hz, 1H), 6.61 (d, *J* = 16.3 Hz, 1H), 5.79 (dd, *J* = 16.3, 6.9 Hz, 1H), 2.46 (s, 3H), 2.36 – 2.28 (m, 1H), 1.97 – 1.93 (m, 2H), 1.85 – 1.80 (m, 2H), 1.75– 1.70 (m, 1H), 1.45 – 1.26 (m, 5H). **^{13}C NMR** (101 MHz, $CDCl_3$) δ 143.35, 134.53, 132.66, 132.25, 132.17, 128.81, 127.97, 126.28, 125.54, 125.35, 124.57, 123.51, 41.68, 33.12, 26.23, 26.07, 20.87. **MS (EI, 70 ev)**: *m/z* (relative intensity) = 250 ($M^{+\bullet}$, 82), 235 (23), 207 (18), 193 (19), 179 (54), 167 (100), 153 (40), 143 (18), 115 (6), 89 (5), 55 (4), 41 (5).

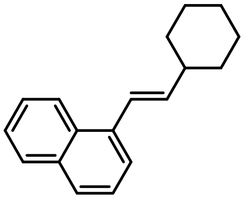

***(E)*-1-(2-cyclohexylvinyl)naphthalene (4t)**: 0.5 mmol (94 mg) **3t** was employed with general procedure GP5 and 111 mg (94 % yield) **4t** was obtained as colorless oil using column chromatography (eluent: 100% cyclohexane). **^{1}H NMR** (400 MHz, Chloroform-*d*) δ 8.08 (d, *J* = 7.7 Hz, 1H), 7.80 – 7.77 (m, 1H), 7.68 (d, *J* = 8.1 Hz, 1H), 7.51 – 7.36 (m, 4H), 7.21 (s, 1H), 7.03 (d, *J* = 15.7 Hz, 1H), 6.15 (dd, *J* = 15.7, 6.9 Hz, 1H), 2.25 – 2.18 (m, 1H), 1.88 – 1.65 (m, 5H), 1.38 – 1.21 (m, 5H). **^{13}C NMR** (101 MHz, $CDCl_3$) δ 140.21, 135.89, 133.61, 131.19, 128.43, 127.11, 125.69, 125.64, 125.56, 124.34, 123.94, 123.40, 41.53, 33.05, 26.21, 26.07.

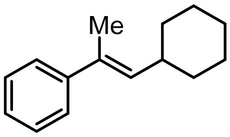

***(E)*-(1-cyclohexylprop-1-en-2-yl)benzene (4u)**: 0.5 mmol (76 mg) **3u** was employed with general procedure GP5 and 94 mg (94 % yield) **4u** was obtained as colorless oil using column chromatography (eluent: 100% cyclohexane). **^{1}H NMR** (400 MHz, Chloroform-*d*) δ 7.39 (d, *J* = 7.7 Hz, 2H), 7.30 (t, *J* = 7.6 Hz, 2H), 7.21 (t, *J* = 7.2 Hz, 1H), 5.64 (d, *J* = 8.9 Hz, 1H), 2.41 – 2.31 (m, 1H), 2.05 (d, *J* = 1.0 Hz, 3H), 1.77 – 1.67 (m, 5H), 1.39 – 1.12 (m, 5H). **^{13}C NMR** (101 MHz, $CDCl_3$) δ 144.03, 134.57, 132.72, 128.08, 126.39, 125.60, 37.75, 33.06, 26.12, 26.01, 15.80.

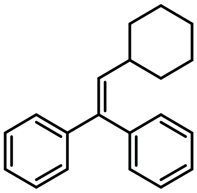

(2-cyclohexylethene-1,1-diyl)dibenzene (4v): 0.5 mmol (107 mg) **3v** was employed with general procedure GP5 and 128 mg (98 % yield) **4v** was obtained as colorless oil using column chromatography (eluent: 100% cyclohexane). **^{1}H NMR** (400 MHz, Chloroform-*d*) δ 7.35 – 7.26 (m, 3H), 7.25 – 7.14 (m, 7H), 5.87 (d, *J* = 10.0 Hz, 1H), 2.13 – 2.06 (m, 1H), 1.66 – 1.56 (m, 5H), 1.21 – 1.10 (m, 5H). **^{13}C NMR** (101 MHz, $CDCl_3$) δ 142.91, 140.56, 139.57, 135.94, 129.76, 128.10, 128.00, 127.17, 126.74, 126.68, 38.29, 33.32, 25.98, 25.58.

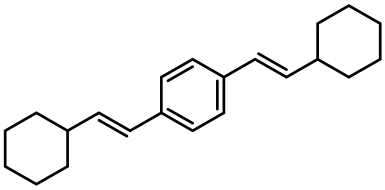

1,4-bis(*(E)*-2-cyclohexylvinyl)benzene (4w): 4.7 mmol (930 mg) **3w** was employed with general procedure GP5 and 1389 mg (49 % yield) **4w** was obtained as white solid using column chromatography (eluent: 100% cyclohexane). Melting point: 93.1 ^{o}C. **^{1}H NMR** (400 MHz, Chloroform-*d*) δ 7.29 (s, 4H), 6.33 (dd, *J* = 16.0, 1.0 Hz, 2H), 6.17 (dd, *J* = 16.0, 6.9 Hz, 2H), 2.14 (ddp, *J* = 10.2, 6.7, 3.6, 3.1 Hz, 2H), 1.88 – 1.66 (m, 10H), 1.41 – 1.28 (m, 5H), 1.26 – 1.15 (m, 5H). **^{13}C NMR** (101 MHz, $CDCl_3$) δ 136.56, 136.28, 126.97, 126.02, 41.18, 32.97, 26.18, 26.06.

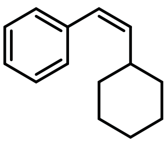

***(Z)*-(2-cyclohexylvinyl)benzene (4x)**: 0.5 mmol (69 mg) **3x** was employed with general procedure GP5 and 86 mg (cis: trans = 88:12, 93 % yield) **4x** was obtained as colorless oil using column chromatography (eluent: 100% cyclohexane). **^{1}H NMR** (400 MHz, Chloroform-*d*) δ 7.37 – 7.22 (m, 5H), 6.33 (d, *J* = 11.7 Hz, 1H), 5.51 (dd, *J* = 11.6, 10.2 Hz, 1H), 2.65 – 2.56 (m, 1H), 1.82 – 1.67 (m, 5H), 1.38 –

1.27 (m, 3H), 1.22 – 1.13 (m, 2H). **^{13}C NMR** (101 MHz, $CDCl_3$) δ 138.99, 137.95, 128.58, 128.15, 126.80, 126.39, 36.89, 33.26, 26.03, 25.67.

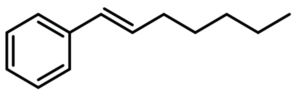

***(E)*-hept-1-en-1-ylbenzene (5a)**: The Grignard reagent was prepared according to GP6 starting from 0.93 mL (7.5 mmol) of 1-bromopentane in 15 mL THF (0.47 M). Titration: C = 0.37 M. Based on the concentration of the Grignard reagent, 6.5 mL THF was added to the vial of *(E)*-(2-chlorovinyl)benzene (**3a**, 2 mmol, 276 mg, 0.31 M) ($C_{Grignard\ reagent}$ = 1.2 C_{Sub}) with $Fe(acac)_3$ (7.0 mg, 1 mol%). Prepared according to general procedure GP7, the solution of the substrate was charged to a syringe pump and mixed with Grignard reagent pumped with T-mixture, the combined solution was passed through a 0.5 mL PFA (I.D. 0.75 mm) reactor at room temperature (Rt = 30 s). 165 mg (95 % yield, took 6.5 mL mixture after reaction containing 1.0 mmol *(E)*-(2-chlorovinyl)benzene for isolation) **5a** was obtained as colorless oil using column chromatography (eluent: 100% cyclohexane). **^{1}H NMR** (400 MHz, Chloroform-*d*) δ 7.37 – 7.33 (m, 2H), 7.32 – 7.28 (m, 2H), 7.22 – 7.18 (m, 1H), 6.39 (d, *J* = 15.8 Hz, 1H), 6.24 (dt, *J* = 15.8, 6.8 Hz, 1H), 2.25 – 2.19 (m, 2H), 1.53 – 1.45 (m, 2H), 1.38 – 1.30 (m, 4H), 0.92 (t, *J* = 7.0 Hz, 3H). **^{13}C NMR** (101 MHz, $CDCl_3$) δ 137.94, 131.24, 129.66, 128.44, 126.71, 125.88, 33.01, 31.44, 29.06, 22.56, 14.06.

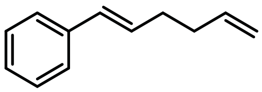

***(E)*-hexa-1,5-dien-1-ylbenzene (5b)**: The Grignard reagent was prepared according to GP6 starting from 0.7 mL (7 mmol) of 4-bromobut-1-ene in 10 mL THF (0.65 M). Titration: C = 0.42 M. Based on the concentration of the Grignard reagent, 3 mL THF was added to the vial of *(E)*-(2-chlorovinyl)benzene (**3a**, 1 mmol, 138 mg, 0.33 M) ($C_{Grignard\ reagent}$ = 1.2 C_{Sub}) with $Fe(acac)_3$ (7.0 mg, 2 mol%). Prepared according to general procedure GP7, the solution of the substrate was charged to a syringe pump and mixed with Grignard reagent pumped with T-mixture, the combined solution was passed through a 1.5 mL PFA (I.D. 0.75 mm) reactor at room temperature (Rt = 90 s). 144 mg (91 % yield, took all mixture after reaction containing 1.0 mmol *(E)*-(2-chlorovinyl)benzene for isolation) **5b** was obtained as colorless oil using column chromatography (eluent: 100% cyclohexane). **^{1}H NMR** (400 MHz, Chloroform-*d*) δ 7.37 – 7.27 (m, 4H), 7.22 – 7.17 (m, 1H), 6.41 (d, *J* = 15.9 Hz, 1H), 6.23 (dt, *J* = 15.8, 6.6 Hz, 1H), 5.87 (ddt, *J* = 16.7, 10.2, 6.4 Hz, 1H), 5.11 – 4.96 (m, 2H), 2.36 – 2.30 (m, 2H), 2.28 – 2.21 (m, 2H). **^{13}C NMR** (101 MHz, $CDCl_3$) δ 138.09, 137.74, 130.17, 130.11, 128.46, 126.86, 125.94, 114.90, 33.54, 32.42.

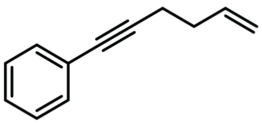

hex-5-en-1-yn-1-ylbenzene (5c): The Grignard reagent was prepared according to GP7 starting from 0.7 mL (7 mmol) of 4-bromobut-1-ene in 10 mL THF (0.65 M). Titration: C = 0.42 M. Based on the concentration of the Grignard reagent, 3 mL THF was added to the vial of (chloroethynyl)benzene (**1a**, 1 mmol, 136 mg, 0.33 M) ($C_{Grignard\ reagent}$ = 1.2 C_{Sub}) with $Fe(acac)_3$ (7.0 mg, 2 mol%). Prepared according to general procedure GP7, the solution of the substrate was charged to a syringe pump and mixed with

Grignard reagent pumped with T-mixture, the combined solution was passed through a 1.0 mL PFA (I.D. 0.75 mm) reactor at 0 ^{o}C (Rt = 60 s). 145 mg (93 % yield, took all mixture after reaction containing 1.0 mmol (chloroethynyl)benzene for isolation) **5c** was obtained as colorless oil using column chromatography (eluent: 100% cyclohexane). **^{1}H NMR** (400 MHz, Chloroform-*d*) δ 7.43 – 7.38 (m, 2H), 7.32 – 7.24 (m, 3H), 5.94 (ddt, *J* = 16.9, 10.2, 6.6 Hz, 1H), 5.14 (dq, *J* = 17.1, 1.6 Hz, 1H), 5.07 (dd, *J* = 10.2, 1.6 Hz, 1H), 2.51 (t, *J* = 7.3 Hz, 2H), 2.40 – 2.30 (m, 2H). **^{13}C NMR** (101 MHz, $CDCl_3$) δ 136.94, 131.54, 128.16, 128.08, 127.55, 123.89, 115.68, 89.48, 81.00, 32.95, 19.26.

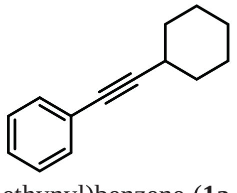

(cyclohexylethynyl)benzene (5d): The Grignard reagent was prepared according to GP7 starting from 0.9 mL (7.5 mmol) of chlorocyclohexane in 15 mL THF (0.47 M). Titration: C = 0.45 M. Based on the concentration of the Grignard reagent, 2.7 mL THF was added to the vial of (chloroethynyl)benzene (**1a**, 1 mmol, 136 mg, 0.37 M) ($C_{Grignard\ reagent}$ = 1.2 C_{Sub}) with $Fe(acac)_3$ (3.5 mg, 1 mol%). Prepared according to general procedure GP7, the solution of the substrate was charged to a syringe pump and mixed with Grignard reagent pumped with T-mixture, the combined solution was passed through a 1.0 mL PFA (I.D. 0.75 mm) reactor at 0 ^{o}C (Rt = 60 s). 110 mg (96 % yield, took 3 mL mixture after reaction containing 0.625 mmol (chloroethynyl)benzene for isolation) **5d** was obtained as colorless oil using column chromatography (eluent: 100% cyclohexane). **^{1}H NMR** (400 MHz, Chloroform-*d*) δ 7.42 – 7.39 (m, 2H), 7.31 – 7.27 (m, 3H), 2.63 – 2.57 (m, 1H), 1.91 – 1.88 (m, 2H), 1.79 – 1.74 (m, 2H), 1.59 – 1.51 (m, 3H), 1.39 –1.39 (m, 3H). **^{13}C NMR** (101 MHz, $CDCl_3$) δ 131.55, 128.12, 127.37, 124.12, 94.45, 80.48, 32.71, 29.66, 25.93, 24.91.

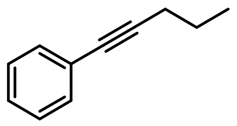

pent-1-yn-1-ylbenzene (5e): The Grignard reagent was prepared according to GP7 starting from 0.68 mL (7.5 mmol) of 1-bromopropane in 15 mL THF (0.48 M). Titration: C = 0.39 M. Based on the concentration of the Grignard reagent, 3.1 mL THF was added to the vial of (chloroethynyl)benzene (**1a**, 1 mmol, 136 mg, 0.32 M) ($C_{Grignard\ reagent}$ = 1.2 C_{Sub}) with $Fe(acac)_3$ (3.5 mg, 1 mol%). Prepared according to general procedure GP7, the solution of the substrate was charged to a syringe pump and mixed with Grignard reagent pumped with T-mixture, the combined solution was passed through a 1.0 mL PFA (I.D. 0.75 mm) reactor at 0 ^{o}C (Rt = 60 s). 136 mg (94 % yield, took all mixture after reaction containing 1.0 mmol (chloroethynyl)benzene for isolation) **5e** was obtained as colorless oil using column chromatography (eluent: 100% cyclohexane). **^{1}H NMR** (400 MHz, Chloroform-*d*) δ 7.43 – 7.38 (m, 2H), 7.30 – 7.25 (m, 3H), 2.40 (t, *J* = 7.0 Hz, 2H), 1.65 (h, *J* = 7.3 Hz, 3H), 1.06 (t, *J* = 7.4 Hz, 3H). **^{13}C NMR** (101 MHz, $CDCl_3$) δ 131.52, 128.15, 127.44, 124.07, 90.24, 80.68, 22.22, 21.39, 13.54.

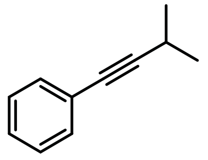

(3-methylbut-1-yn-1-yl)benzene (5f): The Grignard reagent was prepared according to GP7 starting from 0.70 mL (7.5 mmol) of 2-bromopropane in 15 mL THF (0.48 M). Titration: C = 0.36 M. Based on the concentration of

the Grignard reagent, 3.3 mL THF was added to the vial of (chloroethynyl)benzene (**1a**, 1 mmol, 136 mg, 0.30 M) ($C_{Grignard\ reagent}$ = 1.2 C_{Sub}) with $Fe(acac)_3$ (3.5 mg, 1 mol%). Prepared according to general procedure GP7, the solution of the substrate was charged to a syringe pump and mixed with Grignard reagent pumped with T-mixture, the combined solution was passed through a 1.0 mL PFA (I.D. 0.75 mm) reactor at 0 ^{o}C (Rt = 60 s). 68 mg (94 % yield, took 3.3 mL mixture after reaction containing 0.5 mmol (chloroethynyl)benzene for isolation) **5f** was obtained as colorless oil using column chromatography (eluent: 100% cyclohexane). **^{1}H NMR** (400 MHz, Chloroform-d) δ 7.42 – 7.36 (m, 2H), 7.30 – 7.26 (m, 3H), 2.78 (hept, *J* = 6.9 Hz, 1H), 1.28 (s, 3H), 1.26 (s, 3H). **^{13}C NMR** (101 MHz, CDCl3) δ 131.52, 128.12, 127.42, 124.00, 95.75, 79.69, 23.03, 21.11.

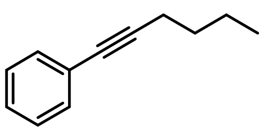

hex-1-yn-1-ylbenzene (5g): The Grignard reagent was prepared according to GP7 starting from 0.85 mL (7.5 mmol) of 1-iodobutane in 15 mL THF (0.47 M). Titration: C = 0.46 M. Based on the concentration of the Grignard reagent, 2.6 mL THF was added to the vial of (chloroethynyl)benzene (**1a**, 1 mmol, 136 mg, 0.37 M) ($C_{Grignard\ reagent}$ = 1.2 C_{Sub}) with $Fe(acac)_3$ (3.5 mg, 1 mol%). Prepared according to general procedure GP7, the solution of the substrate was charged to a syringe pump and mixed with Grignard reagent pumped with T-mixture, the combined solution was passed through a 1.0 mL PFA (I.D. 0.75 mm) reactor at 0 ^{o}C (Rt = 60 s). 155 mg (98 % yield, took all mixture after reaction containing 0.5 mmol (chloroethynyl)benzene for isolation) **5g** was obtained as colorless oil using column chromatography (eluent: 100% cyclohexane). **^{1}H NMR** (400 MHz, Chloroform-d) δ 7.43 – 7.36 (m, 2H), 7.31 – 7.26 (m, 3H), 2.41 (t, *J* = 7.0 Hz, 2H), 1.65 – 1.56 (m, 2H), 1.52 – 1.43 (m, 2H), 0.96 (t, *J* = 7.3 Hz, 3H). **^{13}C NMR** (101 MHz, CDCl3) δ 131.51, 128.15, 127.42, 124.08, 90.40, 80.52, 30.84, 22.01, 19.09, 13.64.

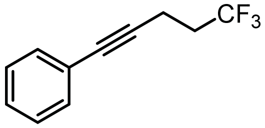

(5,5,5-trifluoropent-1-yn-1-yl)benzene (5h): The Grignard reagent was prepared according to GP7 starting from 0.85 mL (7.5 mmol) of 1,1,1-trifluoro-3-iodopropane in 15 mL THF (0.47 M). Titration: C = 0.44 M. Based on the concentration of the Grignard reagent, 2.7 mL THF was added to the vial of (chloroethynyl)benzene (**1a**, 1 mmol, 136 mg, 0.37 M) ($C_{Grignard\ reagent}$ = 1.2 C_{Sub}) with $Fe(acac)_3$ (3.5 mg, 1 mol%). Prepared according to general procedure GP7, the solution of the substrate was charged to a syringe pump and mixed with Grignard reagent pumped with T-mixture, the combined solution was passed through a 1.0 mL PFA (I.D. 0.75 mm) reactor at 0 ^{o}C (Rt = 60 s). 96 mg (97 % yield, took 2.7 mL mixture after reaction containing 0.5 mmol (chloroethynyl)benzene for isolation) **5h** was obtained as colorless oil using column chromatography (eluent: 100% cyclohexane). **^{1}H NMR** (400 MHz, Chloroform-d) δ 7.43 – 7.37 (m, 2H), 7.35 – 7.27 (m, 3H), 2.78 – 2.63 (m, 2H), 2.53 – 2.36 (m, 2H). **^{13}C NMR** (101 MHz, CDCl3) δ 131.56, 128.26, 128.04, 123.13, 86.07, 81.64, 33.44 (q, J_{C-F} = 29.1 Hz), 13.06 (q, J_{C-F} = 4.2 Hz). **^{19}F NMR** (376 MHz, CDCl3) δ -66.97 (t, *J* = 10.4 Hz).

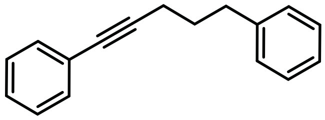

pent-1-yne-1,5-diyldibenzene (5i): The Grignard reagent was prepared according to GP7 starting from 1.1 mL (7.5 mmol) of (3-bromopropyl)benzene in 15 mL THF (0.47 M). Titration: C = 0.29 M. Based on the concentration of the Grignard reagent, 4.1 mL THF was added to the vial of (chloroethynyl)benzene (**1a**, 1 mmol, 136 mg, 0.24 M) ($C_{Grignard\ reagent}$ = 1.2 C_{Sub}) with Fe(acac)$_3$ (3.5 mg, 1 mol%). Prepared according to general procedure GP7, the solution of the substrate was charged to a syringe pump and mixed with Grignard reagent pumped with T-mixture, the combined solution was passed through a 1.0 mL PFA (I.D. 0.75 mm) reactor at 0 ^{o}C (Rt = 60 s). 207 mg (94 % yield, took all mixture after reaction containing 1.0 mmol (chloroethynyl)benzene for isolation) **5i** was obtained as colorless oil using column chromatography (eluent: 100% cyclohexane). ^{1}**H NMR** (400 MHz, Chloroform-*d*) δ 7.46 – 7.39 (m, 2H), 7.35 – 7.27 (m, 5H), 7.22 (dd, *J* = 15.4, 7.1 Hz, 3H), 2.88 – 2.74 (m, 2H), 2.44 (t, *J* = 7.0 Hz, 2H), 1.94 (p, *J* = 7.1 Hz, 2H). ^{13}**C NMR** (101 MHz, CDCl$_3$) δ 141.62, 131.54, 128.55, 128.35, 128.19, 127.54, 125.88, 123.96, 89.81, 81.13, 34.83, 30.31, 18.82.

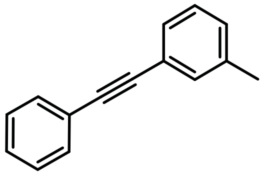

1-methyl-3-(phenylethynyl)benzene (5j): The Grignard reagent was prepared according to GP7 starting from 0.91 mL (7.5 mmol) of 1-bromo-3-methylbenzene in 15 mL THF (0.47 M). Titration: C = 0.39 M. Based on the concentration of the Grignard reagent, 3.1 mL THF was added to the vial of (chloroethynyl)benzene (**1a**, 1 mmol, 136 mg, 0.32 M) ($C_{Grignard\ reagent}$ = 1.2 C_{Sub}) with Fe(acac)$_3$ (3.5 mg, 1 mol%). Prepared according to general procedure GP7, the solution of the substrate was charged to a syringe pump and mixed with Grignard reagent pumped with T-mixture, the combined solution was passed through a 1.0 mL PFA (I.D. 0.75 mm) reactor at 0 ^{o}C (Rt = 60 s). 183 mg (95 % yield, took all mixture after reaction containing 1.0 mmol (chloroethynyl)benzene for isolation) **5j** was obtained as colorless oil using column chromatography (eluent: 100% cyclohexane). ^{1}**H NMR** (400 MHz, Chloroform-*d*) δ 7.56 – 7.49 (m, 2H), 7.39 – 7.31 (m, 5H), 7.27 – 7.21 (m, 1H), 7.17 – 7.12 (m, 1H), 2.36 (s, 3H). ^{13}**C NMR** (101 MHz, CDCl$_3$) δ 137.99, 132.17, 131.58, 129.14, 128.67, 128.30, 128.22, 128.15, 123.36, 123.05, 89.54, 89.01, 21.23.

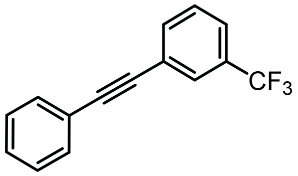

1-(phenylethynyl)-3-(trifluoromethyl)benzene (5k): The Grignard reagent was prepared according to GP7 starting from 1.0 mL (7.5 mmol) of 1-bromo-3-(trifluoromethyl)benzene in 15 mL THF (0.47 M). Titration: C = 0.41 M. Based on the concentration of the Grignard reagent, 2.9 mL THF was added to the vial of (chloroethynyl)benzene (**1a**, 1 mmol, 136 mg, 0.34 M) ($C_{Grignard\ reagent}$ = 1.2 C_{Sub}) with Fe(acac)$_3$ (3.5 mg, 1 mol%). Prepared according to general procedure GP7, the solution of the substrate was charged to a syringe pump and mixed with Grignard reagent pumped with T-mixture, the combined solution was passed through a 1.0 mL PFA (I.D. 0.75 mm) reactor at 0 ^{o}C (Rt = 60 s). 239 mg (97 % yield, took all mixture after reaction containing 1.0 mmol (chloroethynyl)benzene for isolation) **5k** was obtained as colorless oil using column chromatography

(eluent: 100% cyclohexane). ^{1}**H NMR** (400 MHz, Chloroform-*d*) δ 7.81 (s, 1H), 7.70 (d, *J* = 7.7 Hz, 1H), 7.61 – 7.53 (m, 3H), 7.48 (t, *J* = 7.8 Hz, 1H), 7.41 – 7.35 (m, 3H). ^{13}**C NMR** (101 MHz, $CDCl_3$) δ 134.63, 131.69, 130.81 (q, $J_{C\text{-}F}$ = 32.8 Hz), 128.85, 128.74, 128.43, 128.36 (q, $J_{C\text{-}F}$ = 3.9 Hz), 124.73 (q, $J_{C\text{-}F}$ = 3.8 Hz), 124.25, 123.9 (q, $J_{C\text{-}F}$ = 272.7 Hz), 122.59, 90.89, 87.77. ^{19}**F NMR** (376 MHz, CDCl3) δ -62.94.

8.2 Characterization of the starting material:

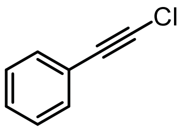

(chloroethynyl)benzene (1a): 7 mmol (814 mg) ethynylbenzene was employed with general procedure GP1 and 780 mg (82 % yield) **1a** was obtained as colorless oil using column chromatography (eluent: 100% cyclohexane). **^{1}H NMR** (400 MHz, Chloroform-*d*) δ 7.45 (dd, *J* = 7.6, 1.8 Hz, 2H), 7.38 – 7.28 (m, 3H). **^{13}C NMR** (101 MHz, $CDCl_3$) δ 131.95, 128.56, 128.34, 122.13, 69.36, 67.99.

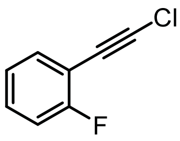

1-(chloroethynyl)-2-fluorobenzene (1b): 5 mmol (600 mg) 1-ethynyl-2-fluorobenzene was employed with general procedure GP1 and 647 mg (84 % yield) **1b** was obtained as pale yellow oil using column chromatography (eluent: 100% cyclohexane). **^{1}H NMR** (400 MHz, Chloroform-*d*) δ 7.47 – 7.40 (m, 1H), 7.35 – 7.29 (m, 1H), 7.13 – 7.02 (m, 2H). **^{13}C NMR** (101 MHz, $CDCl_3$) δ 163.33 (d, *J* = 253.0 Hz), 133.83 (d, *J* = 1.0 Hz), 130.28 (d, *J* = 8.0 Hz), 123.96 (d, *J* = 3.8 Hz), 115.55 (d, *J* = 20.9 Hz), 110.71 (d, *J* = 15.7 Hz), 73.01 (d, *J* = 3.2 Hz), 63.10 (d, *J* = 0.5 Hz). **^{19}F NMR** (376 MHz, $CDCl_3$) δ **-** 110.24 (ddd, *J* = 9.4, 6.9, 5.1 Hz).

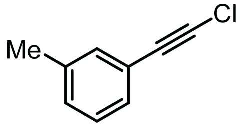

1-(chloroethynyl)-3-methylbenzene (1c): 5 mmol (580 mg) 1-ethynyl-3-methylbenzene was employed with general procedure GP1 and 503 mg (67 % yield) **1c** was obtained as colorless oil using column chromatography (eluent: 100% cyclohexane). **^{1}H NMR** (400 MHz, Chloroform-*d*) δ 7.29 – 7.13 (m, 4H), 2.33 (s, 3H). **^{13}C NMR** (101 MHz, $CDCl_3$) δ 138.06, 132.51, 129.47, 129.01, 128.23, 121.92, 69.51, 67.54, 21.16.

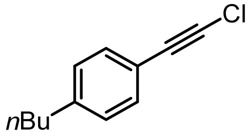

1-butyl-4-(chloroethynyl)benzene (1d): 5 mmol (790 mg) 1-butyl-4-ethynylbenzene was employed with general procedure GP1 and 760 mg (79 % yield) **1d** was obtained as colorless oil using column chromatography (eluent: 100% cyclohexane). **^{1}H NMR** (400 MHz, Chloroform-*d*) δ 7.38 (d, *J* = 8.2 Hz, 2H), 7.14 (d, *J* = 8.2 Hz, 2H), 2.62 (t, *J* = 7.7 Hz, 2H), 1.61 (p, *J* = 7.5 Hz, 2H), 1.37 (dq, *J* = 14.6, 7.3 Hz, 2H), 0.96 (t, *J* = 7.3 Hz, 3H). **^{13}C NMR** (101 MHz, $CDCl_3$) δ 143.71, 131.83, 128.44, 119.23, 69.53, 67.11, 35.56, 33.34, 22.29, 13.89.

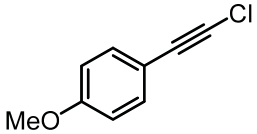

1-(chloroethynyl)-4-methoxybenzene (1e): 5 mmol (660 mg) 1-ethynyl-4-methoxy-benzene was employed with general procedure GP1 and 690 mg (83 % yield) **1e** was obtained as pale yellow oil using column chromatography (eluent: 100% cyclohexane). **^{1}H NMR** (400 MHz, Chloroform-*d*) δ 7.38 (d, *J* = 8.9 Hz, 2H), 6.84 (d, *J* = 8.8 Hz, 2H), 3.81 (s, 3H). **^{13}C NMR** (101 MHz, $CDCl_3$) δ 159.77, 133.36, 114.14, 113.96, 69.26, 66.34, 55.24.

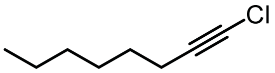

1-chlorooct-1-yne (1f): 7 mmol (880 mg) oct-1-yne was employed with general procedure GP1 and 756 mg (75 % yield) **1f** was obtained as pale yellow oil using column chromatography (eluent: 100% cyclohexane). **^{1}H NMR** (400 MHz, Chloroform-*d*) δ 2.20 – 2.12 (m, 2H), 1.50 (dt, *J* = 14.4, 6.7 Hz, 2H), 1.42 – 1.24 (m, 6H), 0.89 (t, *J* = 7.0 Hz, 3H). **^{13}C NMR** (101 MHz, $CDCl_3$) δ 69.78, 56.90, 31.28, 30.92, 28.46, 28.33, 22.52, 18.75, 14.03.

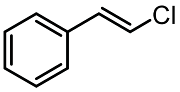

***(E)*-(2-chlorovinyl)benzene (3a)**: 7 mmol (1036 mg) cinnamic acid was employed with general procedure GP2 and 754 mg (78 % yield) **3a** was obtained as colorless oil using column chromatography (eluent: 100% cyclohexane). **^{1}H NMR** (400 MHz, Chloroform-*d*) δ 7.40 – 7.27 (m, 5H), 6.87 (d, *J* = 13.7 Hz, 1H), 6.67 (d, *J* = 13.7 Hz, 1H). **^{13}C NMR** (101 MHz, $CDCl_3$) δ 134.82, 133.23, 128.74, 128.10, 126.07, 118.65.

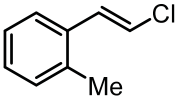

***(E)*-1-(2-chlorovinyl)-2-methylbenzene (3i)**: 3 mmol (486 mg) *(E)*-3-(*o*-tolyl)acrylic acid was employed with general procedure GP2 and 96 mg (21 % yield) **3i** was obtained as colorless oil using column chromatography (eluent: 100% cyclohexane). **^{1}H NMR** (400 MHz, Chloroform-*d*) δ 7.32 (d, *J* = 7.2 Hz, 1H), 7.24 – 7.14 (m, 3H), 7.05 (d, *J* = 13.5 Hz, 1H), 6.52 (d, *J* = 13.5 Hz, 1H), 2.34 (s, 3H). **^{13}C NMR** (101 MHz, $CDCl_3$) δ 135.34, 133.96, 131.56, 130.43, 128.16, 126.22, 125.78, 119.30, 19.81.

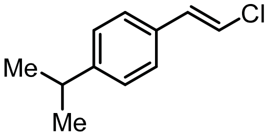

***(E)*-1-(2-chlorovinyl)-4-isopropylbenzene (3j)**: 2 mmol (380 mg) *(E)*-3-(4-isopropyl-phenyl)acrylic acid was employed with general procedure GP2 and 170 mg (47 % yield) **3j** was obtained as colorless oil using column chromatography (eluent: 100% cyclohexane). **^{1}H NMR** (400 MHz, Chloroform-*d*) δ 7.22 (q, *J* = 8.2 Hz, 4H), 6.82 (d, *J* = 13.6 Hz, 1H), 6.60 (d, *J* = 13.6 Hz, 1H), 2.96 – 2.84 (m, 1H), 1.25 (d, *J* = 6.9 Hz, 6H). **^{13}C NMR** (101 MHz, $CDCl_3$) δ 149.06, 133.12, 132.46, 126.83, 126.09, 117.72, 33.88, 23.85. **MS (EI, 70 ev)**: *m/z* (relative intensity) = 180 ($M^{+\bullet}$, 46), 165 (100), 145 (4), 129 (43), 103 (6), 77 (10), 51 (8).

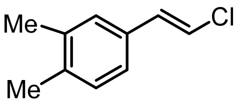

***(E)*-4-(2-chlorovinyl)-1,2-dimethylbenzene (3k)**: 2 mmol (352 mg) *(E)*-3-(3,4-dimethyl-phenyl)acrylic acid was employed with general procedure GP2 and 220 mg (66 % yield) **3k** was obtained as colorless oil using column chromatography (eluent: 100% cyclohexane). **^{1}H NMR** (400 MHz, Chloroform-*d*) δ 7.12 – 7.05(m, 3H), 6.80 (d, *J* = 13.6 Hz, 1H), 6.60 (d, *J* = 13.6 Hz, 1H), 2.28 (s, 6H). **^{13}C NMR** (101 MHz, $CDCl_3$) δ 136.91, 136.76, 133.19, 132.47, 129.98, 127.32, 123.53, 117.41, 77.32, 77.00, 76.68, 19.72, 19.52.

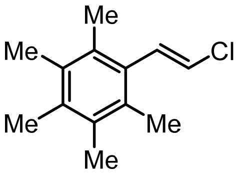

***(E)*-1-(2-chlorovinyl)-2,3,4,5,6-pentamethylbenzene (3l)**: 2 mmol (436 mg) *(E)*-3-(2,3,4, 5,6-pentamethylphenyl)acrylic acid was employed with general procedure GP2 and 141 mg (34 % yield) **3l** was obtained as white solid using column chromatography (eluent: 100% cyclohexane). Melting

point: 65.9 °C. **^{1}H NMR** (400 MHz, Chloroform-*d*) δ 6.92 (d, *J* = 13.8 Hz, 1H), 6.03 (d, *J* = 13.8 Hz, 1H), 2.29 (s, 3H), 2.27 (s, 12H). **^{13}C NMR** (101 MHz, $CDCl_3$) δ 134.57, 133.31, 132.59, 131.97, 131.83, 120.73, 17.96, 16.78, 16.51. **MS (EI, 70 ev)**: *m/z* (relative intensity) = 208 ($M^{+•}$, 75), 193 (6), 173 (100), 158 (57), 143 (34), 131 (54), 115 (16), 105 (5), 91 (8), 77 (8), 65 (6), 39 (6).

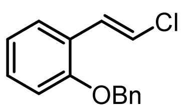

***(E)*-1-(benzyloxy)-2-(2-chlorovinyl)benzene (3m)**: 2 mmol (508 mg) *(E)*-3-(2-(benzyloxy) phenyl)acrylic acid was employed with general procedure GP2 and 350 mg (72 % yield) **3m** was obtained as pale yellow oil using column chromatography (eluent: ethyl acetate/cyclohexane = 1:20). **^{1}H NMR** (400 MHz, Chloroform-*d*) δ 7.48 – 7.21 (m, 7H), 7.11 (d, *J* = 13.7 Hz, 1H), 6.94 – 6.98 (m, 2H), 6.83 (dd, *J* = 13.6, 1.0 Hz, 1H), 5.13 (s, 2H). **^{13}C NMR** (101 MHz, $CDCl_3$) δ 155.71, 136.63, 129.16, 129.06, 128.63, 128.03, 127.97, 127.34, 124.07, 121.00, 119.85, 112.40, 70.32. **MS (EI, 70 ev)**: *m/z* (relative intensity) = 244 ($M^{+•}$, 9), 208 (12), 118 (10), 91 (100), 65 (14), 39 (4).

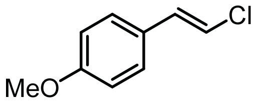

***(E)*-1-(2-chlorovinyl)-4-methoxybenzene (3n)**: 7 mmol (1246 mg) *(E)*-3-(4-methoxyphe-nyl)acrylic acid was employed with general procedure GP2 and 730 mg (62 % yield) **3n** was obtained as pale yellow oil using column chromatography (eluent: ethyl acetate/cyclohexane = 1:20). **^{1}H NMR** (400 MHz, Chloroform-*d*) δ 7.24 (d, *J* = 7.3 Hz, 2H), 6.86 (d, *J* = 7.2 Hz, 2H), 6.78 (d, *J* = 13.6 Hz, 1H), 6.51 (dd, *J* = 13.6, 1.0 Hz, 1H), 3.85 – 3.79 (m, 3H). **^{13}C NMR** (101 MHz, $CDCl_3$) δ 159.55, 132.66, 127.61, 127.32, 116.37, 114.17, 55.28.

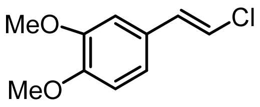

***(E)*-4-(2-chlorovinyl)-1,2-dimethoxybenzene (3o)**: 3 mmol (624 mg) *(E)*-3-(3,4-di-methoxyphenyl)acrylic acid was employed with general procedure GP2 and 457 mg (77 % yield) **3o** was obtained as pale yellow oil using column chromatography (eluent: ethyl acetate/cyclohexane = 1:20). **^{1}H NMR** (400 MHz, Chloroform-*d*) δ 6.87 – 6.78 (m, 3H), 6.75 (d, *J* = 13.6 Hz, 1H), 6.51 (d, *J* = 13.6 Hz, 1H), 3.88 (s, 3H), 3.87 (s, 3H). **^{13}C NMR** (101 MHz, $CDCl_3$) δ 149.15, 149.06, 132.87, 127.83, 119.25, 116.60, 111.15, 108.56, 55.85, 55.79.

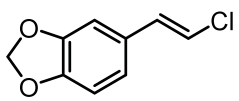

***(E)*-5-(2-chlorovinyl)benzo*[d]*[1,3]dioxole (3p)**: 5 mmol (960 mg) *(E)*-3-(benzo[*d*][1,3] dioxol-5-yl)acrylic acid was employed with general procedure GP2 and 470 mg (52 % yield) **3p** was obtained as pale yellow oil using column chromatography (eluent: ethyl acetate/cyclohexane = 1:20). **^{1}H NMR** (400 MHz, Chloroform-*d*) δ 6.81 (s, 1H), 6.78 – 6.70 (m, 3H), 6.48 (d, *J* = 13.6 Hz, 1H), 5.96 (s, 2H). **^{13}C NMR** (101 MHz, $CDCl_3$) δ 148.09, 147.62, 132.82, 129.11, 120.82, 116.84, 108.42, 105.41, 101.20.

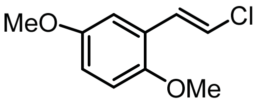

***(E)*-2-(2-chlorovinyl)-1,4-dimethoxybenzene (3q)**: 5 mmol (1040 mg) *(E)*-3-(2,5-dimethoxyphenyl)acrylic acid was employed with general procedure GP2 and 762 mg (77 % yield) **3q** was obtained as pale yellow oil using column

chromatography (eluent: ethyl acetate/cyclohexane = 1:20). **^{1}H NMR** (400 MHz, Chloroform-*d*) δ 7.00 (d, *J* = 13.6 Hz, 1H), 6.85 – 6.76 (m, 4H), 3.81 (s, 3H), 3.77 (s, 3H). **^{13}C NMR** (101 MHz, $CDCl_3$) δ 153.52, 151.04, 129.06, 124.43, 120.21, 113.86, 113.35, 112.06, 55.98, 55.70. **MS (EI, 70 ev)**: *m/z* (relative intensity) = 198 ($M^{+\bullet}$, 100), 183 (23), 155 (83), 133 (17), 105 (10), 91 (25), 77 (14), 51 (8).

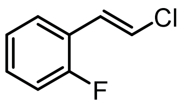

***(E)*-1-(2-chlorovinyl)-2-fluorobenzene (3r)**: 3 mmol (498 mg) *(E)*-3-(2-fluorophenyl)acrylic acid was employed with general procedure GP2 and 318 mg (68 % yield) **3r** was obtained as pale yellow oil using column chromatography (eluent: 100% cyclohexane). **^{1}H NMR** (400 MHz, Chloroform-*d*) δ 7.33 – 7.21 (m, 2H), 7.13 – 7.02 (m, 2H), 6.90 (d, *J* = 13.8 Hz, 1H), 6.80 (d, *J* = 13.8 Hz, 1H). **^{13}C NMR** (101 MHz, $CDCl_3$) δ 159.88 (d, $J_{C\text{-}F}$ = 250.3 Hz), 129.40 (d, $J_{C\text{-}F}$ = 8.5 Hz), 128.01 (d, $J_{C\text{-}F}$ = 3.7 Hz), 126.74 (d, $J_{C\text{-}F}$ = 1.8 Hz), 124.31 (d, $J_{C\text{-}F}$ = 3.6 Hz), 122.63 (d, $J_{C\text{-}F}$ = 12.8 Hz), 121.67 (d, $J_{C\text{-}F}$ = 8.7 Hz), 115.94 (d, $J_{C\text{-}F}$ = 22.0 Hz). **^{19}F NMR** (376 MHz, $CDCl_3$) δ -115.78 (ddd, *J* = 10.9, 7.4, 5.4 Hz).

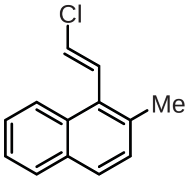

***(E)*-1-(2-chlorovinyl)-2-methylnaphthalene (3s)**: 1 mmol (212 mg) *(E)*-3-(2-methylnaphthal-en-1-yl)acrylic acid was employed with general procedure GP2 and 148 mg (73 % yield) **3s** was obtained as colorless oil using column chromatography (eluent: 100% cyclohexane). **^{1}H NMR** (400 MHz, Chloroform-*d*) δ 8.02 (d, *J* = 8.4 Hz, 1H), 7.81 (d, *J* = 7.9 Hz, 1H), 7.72 (d, *J* = 8.4 Hz, 1H), 7.47 (dt, *J* = 21.0, 7.0 Hz, 2H), 7.34 (d, *J* = 8.4 Hz, 1H), 7.18 (d, *J* = 13.8 Hz, 1H), 6.33 (d, *J* = 14.2 Hz, 1H), 2.49 (s, 3H). **^{13}C NMR** (101 MHz, $CDCl_3$) δ 133.65, 132.07, 130.38, 130.10, 128.74, 128.13, 127.77, 126.30, 125.07, 124.77, 122.78, 20.87. **MS (EI, 70 ev)**: *m/z* (relative intensity) = 202 ($M^{+\bullet}$, 30), 167 (100), 152 (39), 115 (5), 100 (5), 82 (20), 63 (4).

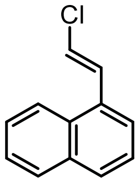

***(E)*-1-(2-chlorovinyl)naphthalene (3t)**: 1 mmol (198 mg) *(E)*-3-(naphthalen-1-yl)acrylic acid was employed with general procedure GP2 and 147 mg (78 % yield) **3t** was obtained as colorless oil using column chromatography (eluent: 100% cyclohexane). **^{1}H NMR** (400 MHz, Chloroform-*d*) δ 7.91 – 7.85 (m, 1H), 7.74 – 7.63 (m, 2H), 7.44 – 7.25 (m, 5H), 6.49 (d, *J* = 13.4 Hz, 1H). **^{13}C NMR** (101 MHz, $CDCl_3$) δ 133.57, 132.36, 131.05, 130.80, 128.69, 128.54, 126.43, 126.09, 125.51, 124.22, 123.74, 120.54.

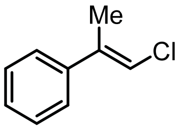

***(E)*-(1-chloroprop-1-en-2-yl)benzene (3u)**: 2 mmol (324 mg) *(E)*-3-phenylbut-2-enoic acid was employed with general procedure GP2 and 122 mg (40 % yield) **3u** was obtained as colorless oil using column chromatography (eluent: 100% cyclohexane). **^{1}H NMR** (400 MHz, Chloroform-*d*) δ 7.37 – 7.27 (m, 5H), 6.33 (d, *J* = 1.3 Hz, 1H), 2.21 (d, *J* = 1.3 Hz, 3H). **^{13}C NMR** (101 MHz, $CDCl_3$) δ 140.30, 138.51, 128.50, 127.74, 125.90, 115.75, 16.87.

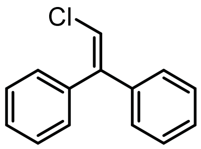

(2-chloroethene-1,1-diyl)dibenzene (3v): 1 mmol (224 mg) 3,3-diphenylacrylic acid was employed with general procedure GP2 and 146 mg (68 % yield) **3v** was obtained as colorless oil using column chromatography (eluent: 100% cyclohexane). **^{1}H NMR** (400 MHz, Chloroform-*d*) δ 7.45 – 7.31 (m, 8H), 7.25 – 7.20 (m, 2H), 6.62 (s, 1H). **^{13}C NMR** (101 MHz, $CDCl_3$) δ 143.84, 140.10, 137.55, 129.84, 128.40, 128.18, 128.05, 127.94, 127.69, 115.85.

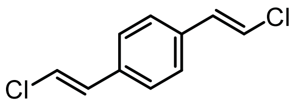

1,4-bis(*(E)*-2-chlorovinyl)benzene (3w): 18 mmol (3924 mg) (*2E,2'E*)-3,3'-(1,4-phenyl-ene)diacrylic acid was employed with general procedure GP2 and 1093 mg (30 % yield) **3w** was obtained as colorless oil using column chromatography (eluent: 100% cyclohexane). **^{1}H NMR** (400 MHz, Chloroform-*d*) δ 7.26 (s, 4H), 6.81 (d, *J* = 13.7 Hz, 2H), 6.66 (d, *J* = 13.7 Hz, 2H). **^{13}C NMR** (101 MHz, $CDCl_3$) δ 134.63, 132.66, 126.48, 119.13.

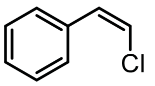

***(Z)*-(2-chlorovinyl)benzene (3x)**: 2 mmol (416 mg) (2,2,2-trichloroethyl)benzene was employed with general procedure GP3 and 201 mg (*cis:tans* = 91:9, 73 % yield) **3x** was obtained as colorless oil using column chromatography (eluent: 100% cyclohexane). **^{1}H NMR** (400 MHz, Chloroform-*d*) δ 7.71 – 7.65 (m, 2H), 7.42 – 7.35 (m, 2H), 7.33 – 7.28 (m, 1H), 6.64 (d, *J* = 8.1 Hz, 1H), 6.27 (d, *J* = 8.1 Hz, 1H). **^{13}C NMR** (101 MHz, $CDCl_3$) δ 134.10, 129.27, 129.23, 128.27, 128.17, 117.57.

9. Reference

1. Shi, D.; Liu, Z.; Zhang, Z.; Shi, W.; Chen, H., Silver-Catalyzed Synthesis of 1-Chloroalkynes Directly from Terminal Alkynes. *ChemCatChem* **2015,** *7*, 1424-1426.

2. Falck, J.; He, A.; Bejot, R.; Mioskowski, C., Preparation of *(Z)*-1-Halo-1-alkenes and *(Z)*-1-Halo-2-alkoxy-1-alkenes Using Cr (II/III) and Fe (0). *Synlett* **2006,** *2006*, 2652-2654.

3. Huck, L.; de la Hoz, A.; Díaz-Ortiz, A.; Alcázar, J., Grignard Reagents on a Tab: Direct Magnesium Insertion under Flow Conditions. *Org. Lett.* **2017,** *19*, 3747-3750.

10.NMR spectra

(cyclohexylethynyl)benzene (2a)

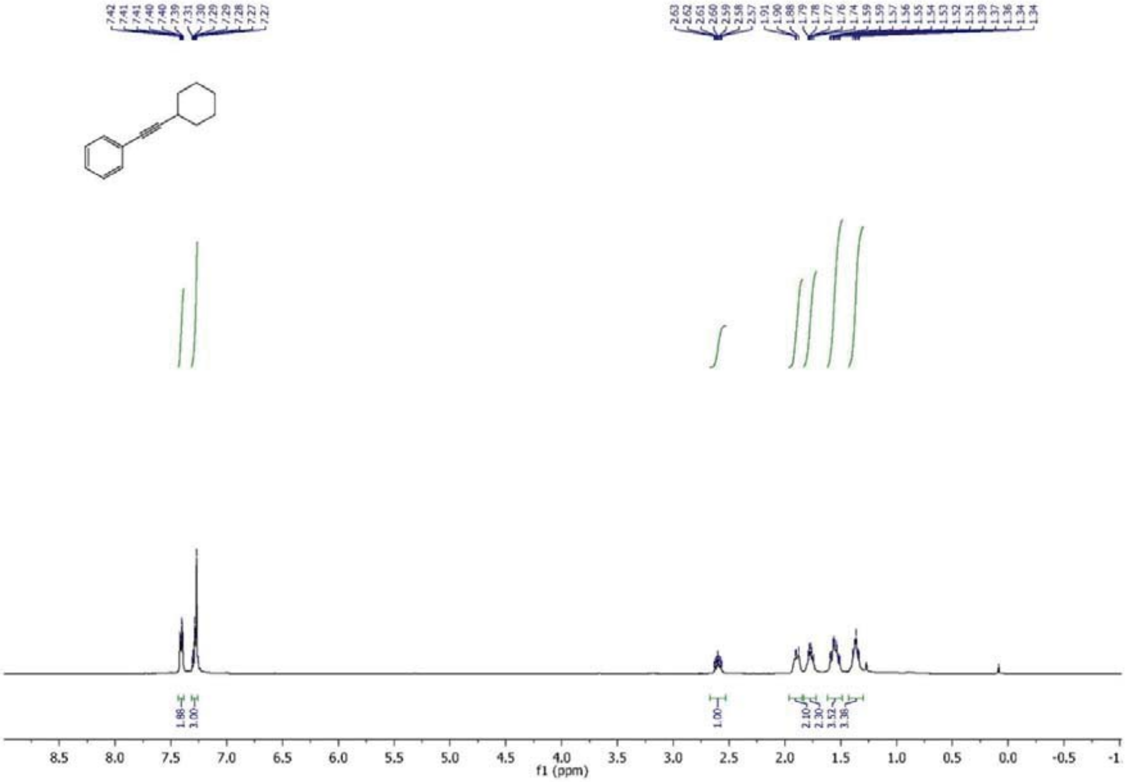

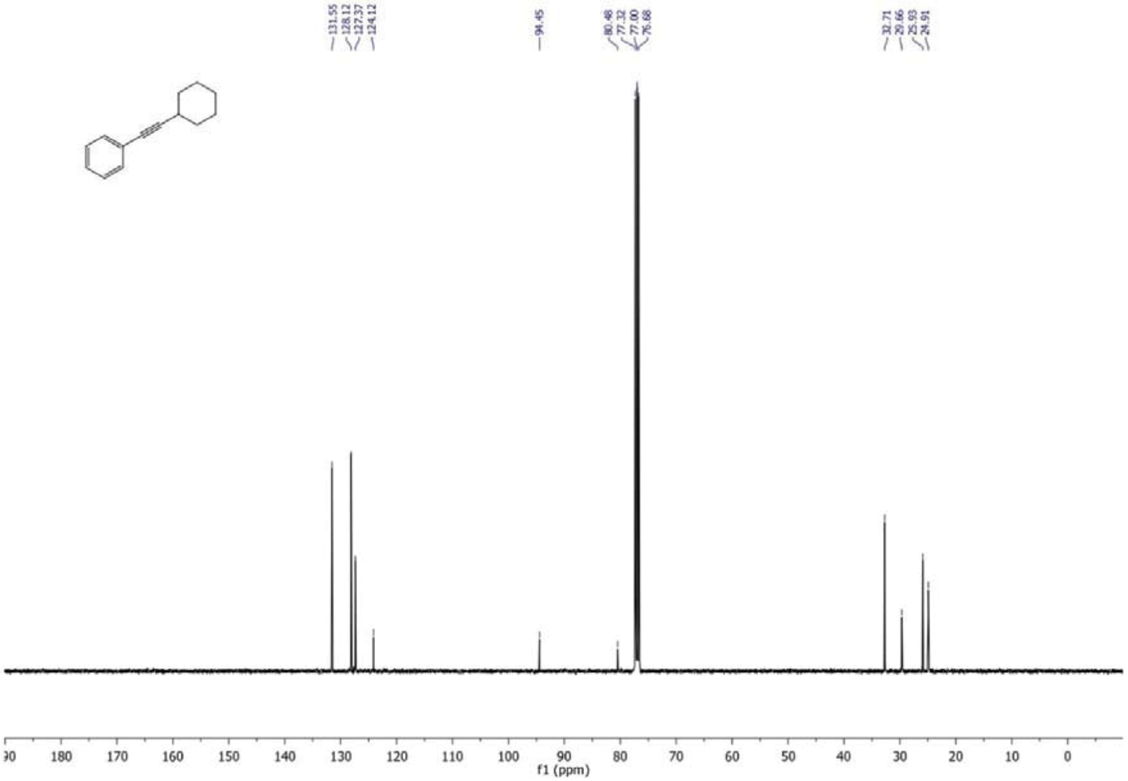

1-(cyclohexylethynyl)-2-fluorobenzene (2b)

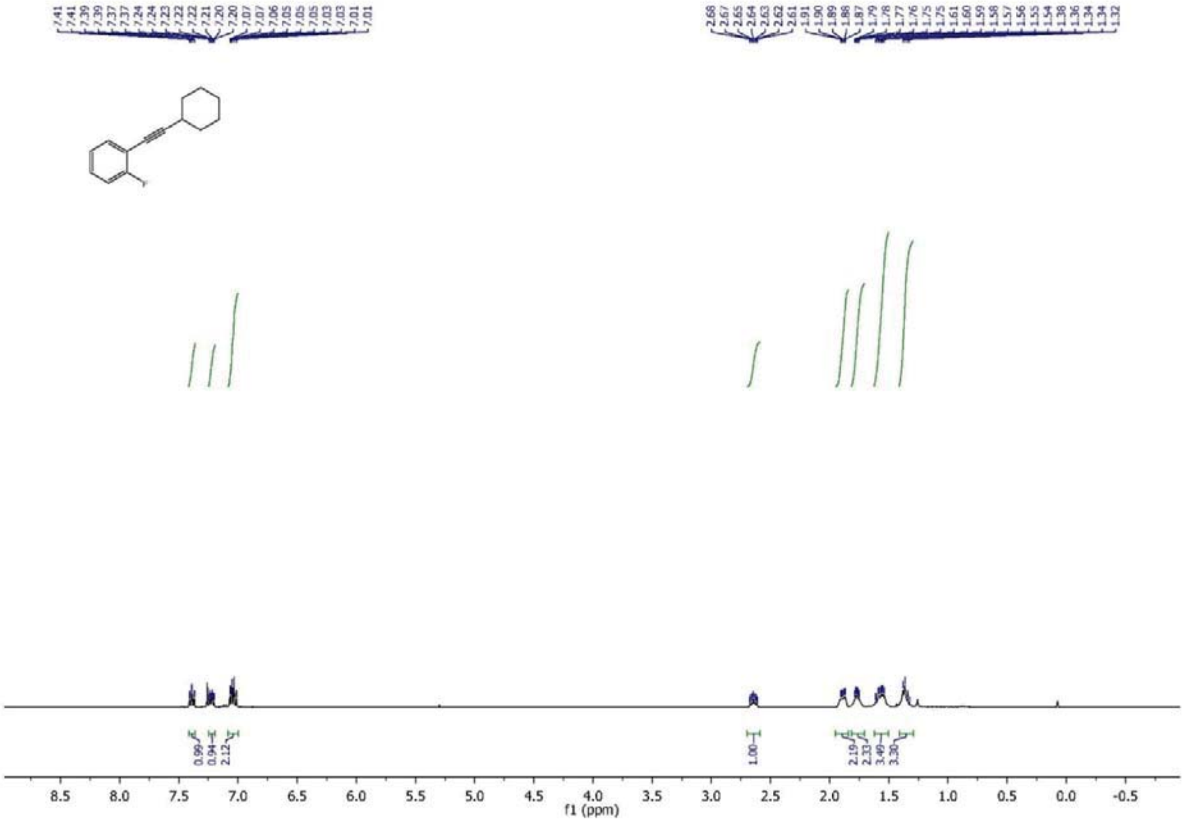

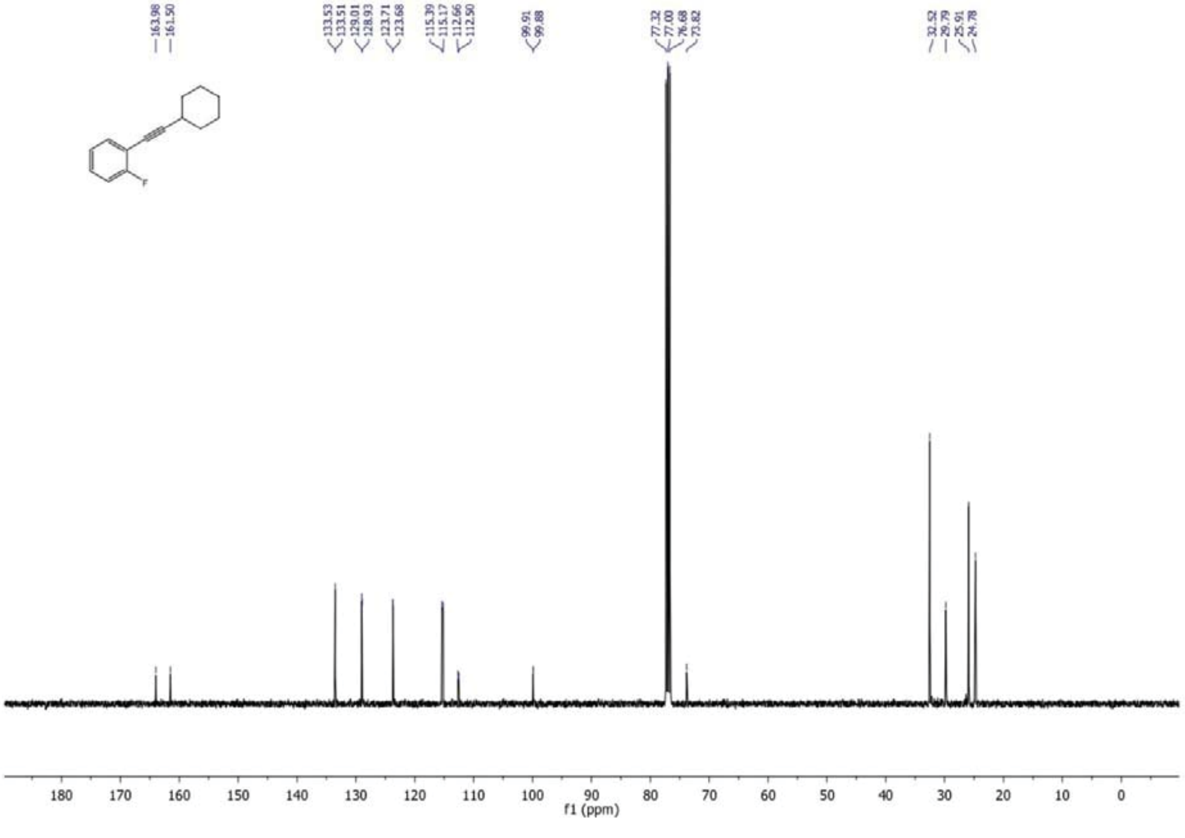

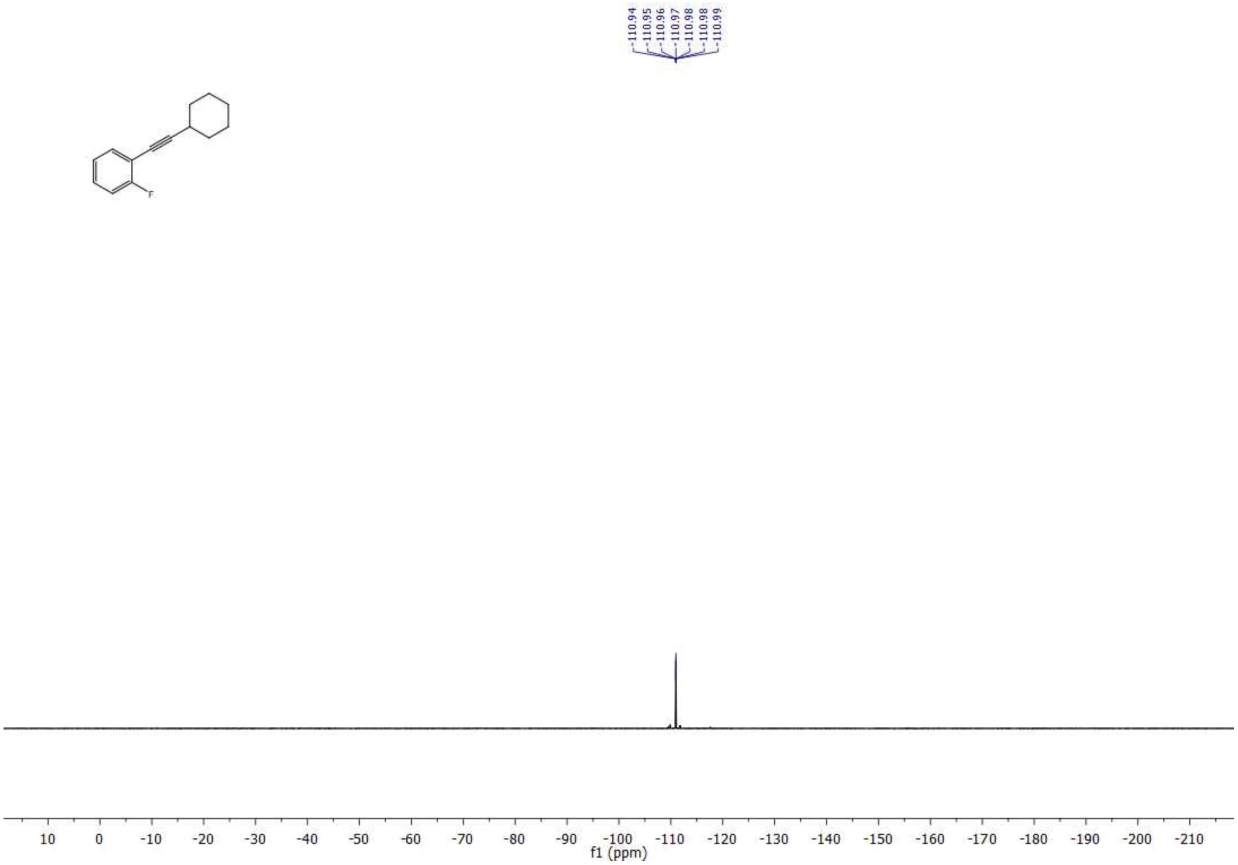

1-(cyclohexylethynyl)-3-methylbenzene (2c)

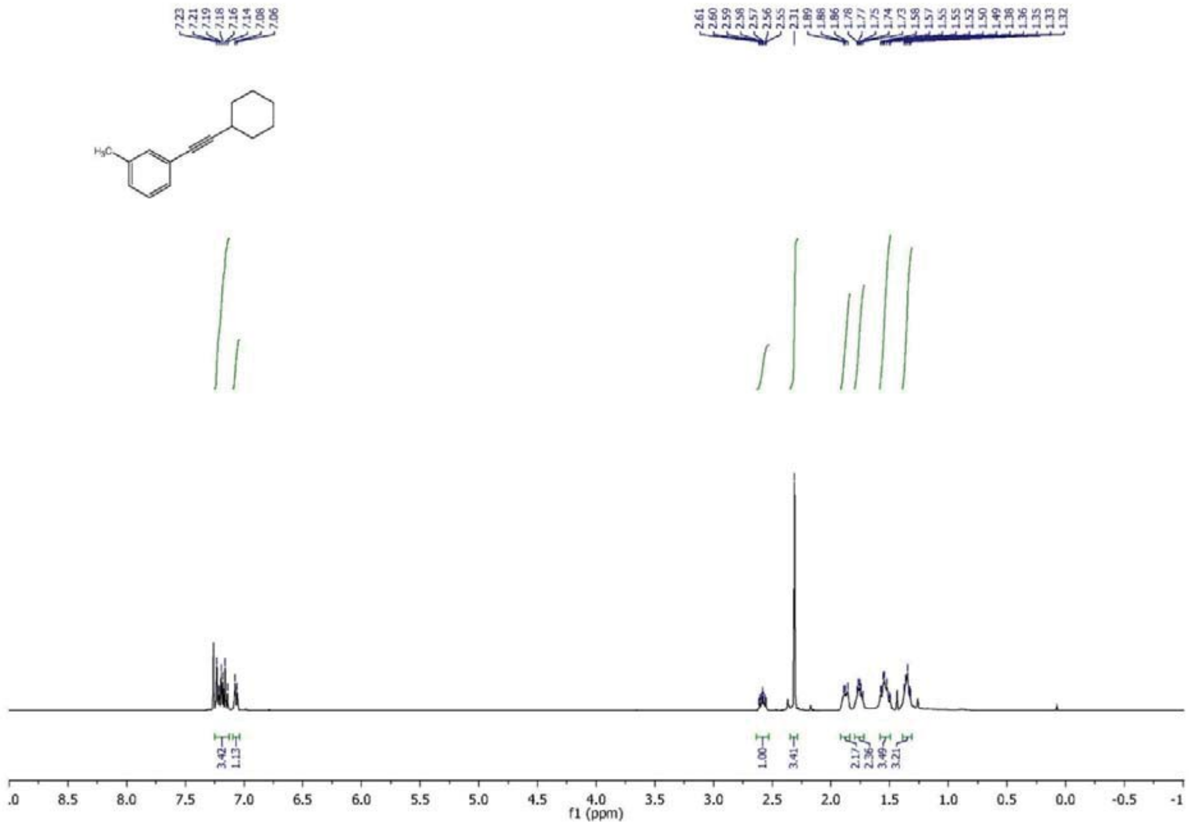

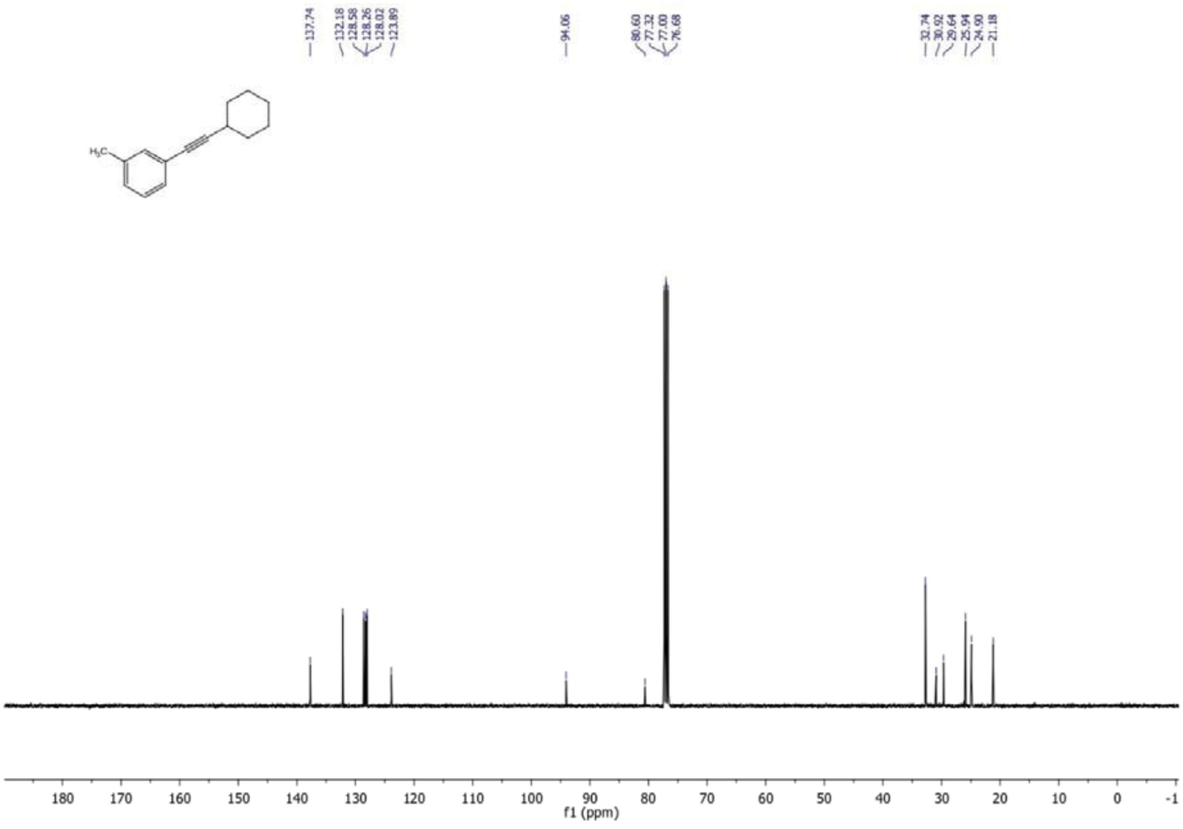

1-butyl-4-(cyclohexylethynyl)benzene (2d)

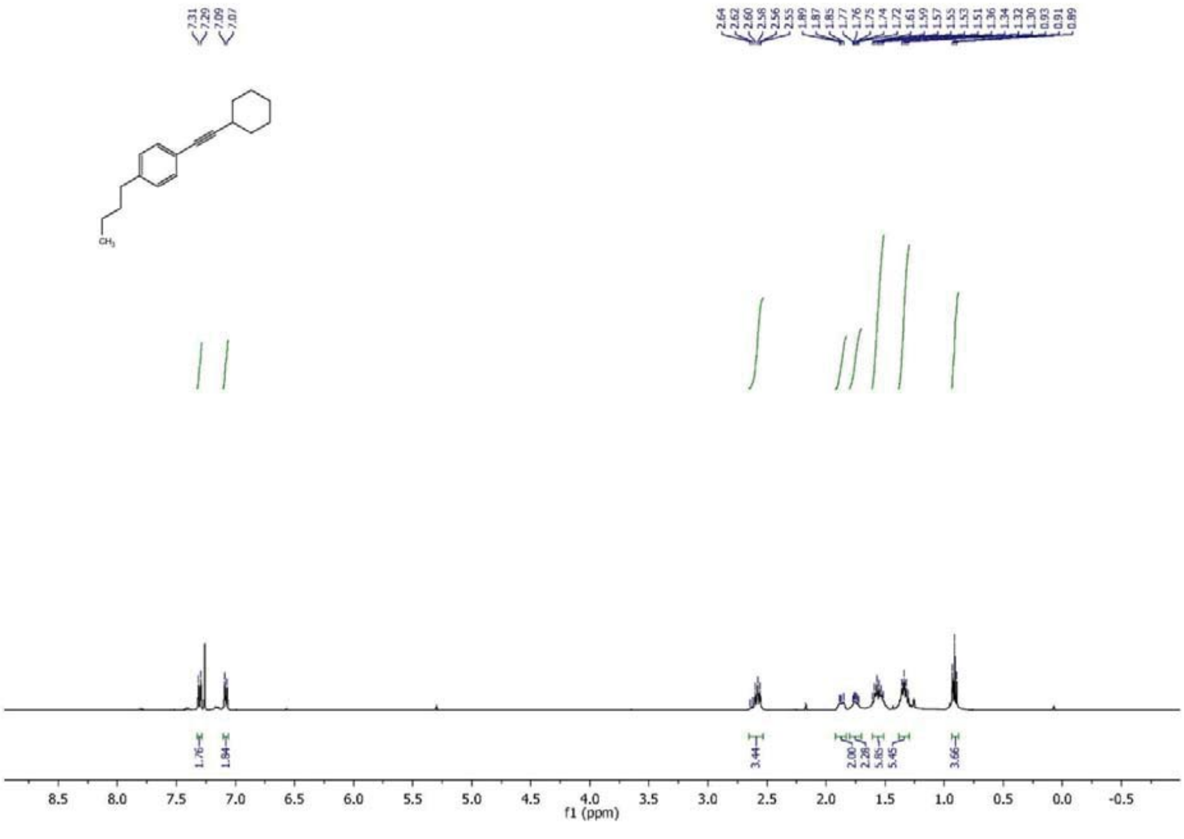

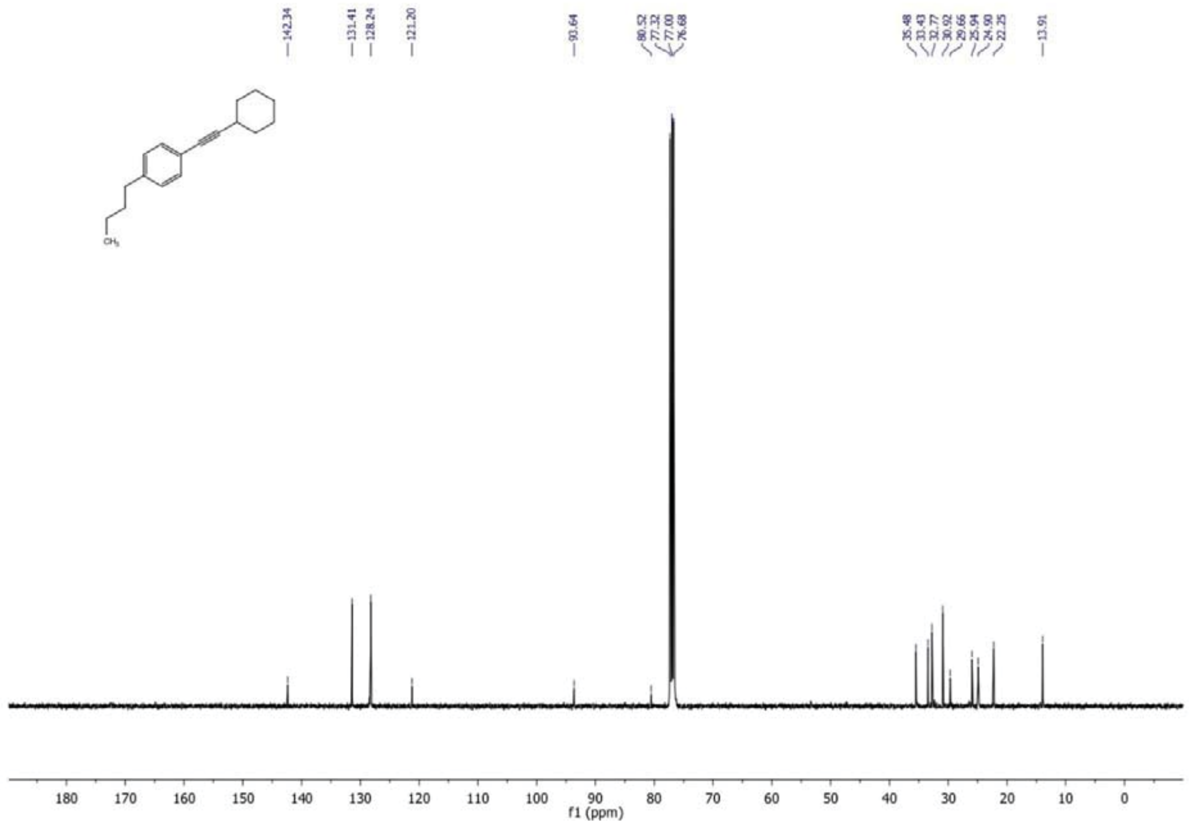

1-(cyclohexylethynyl)-4-methoxybenzene (2e)

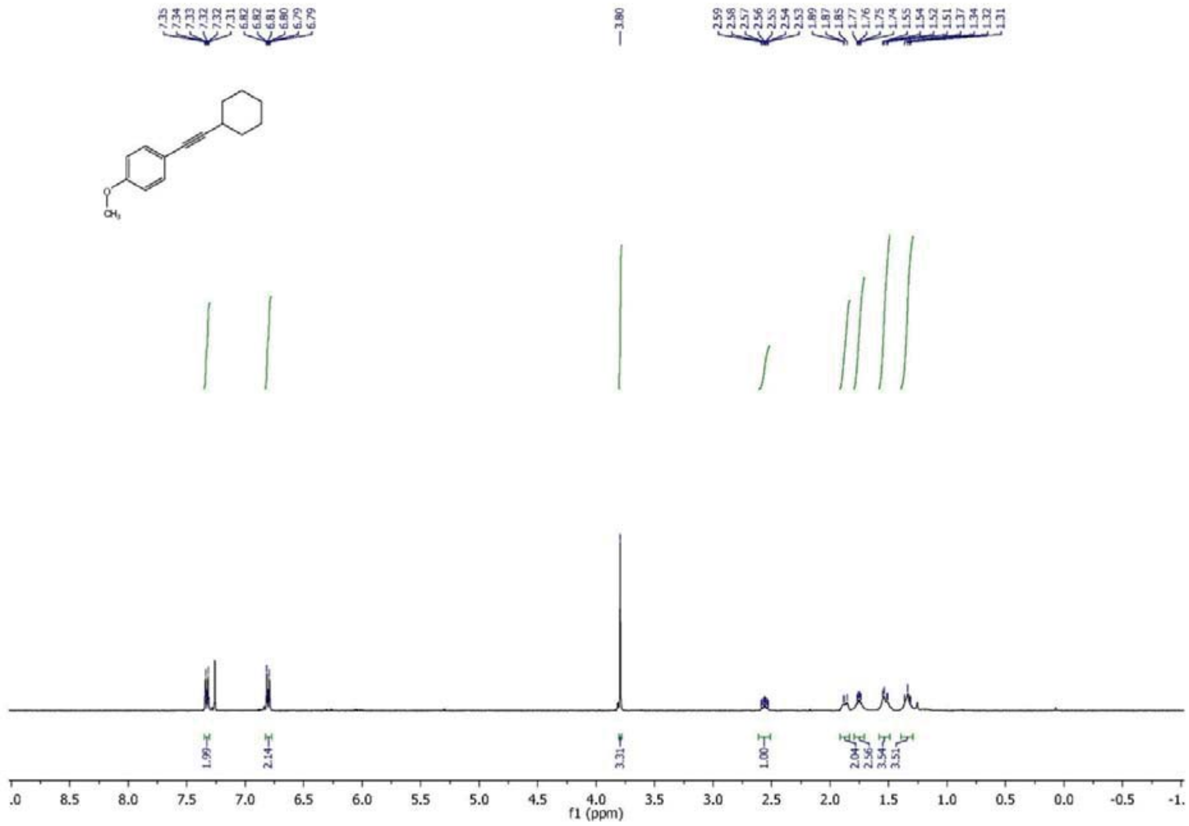

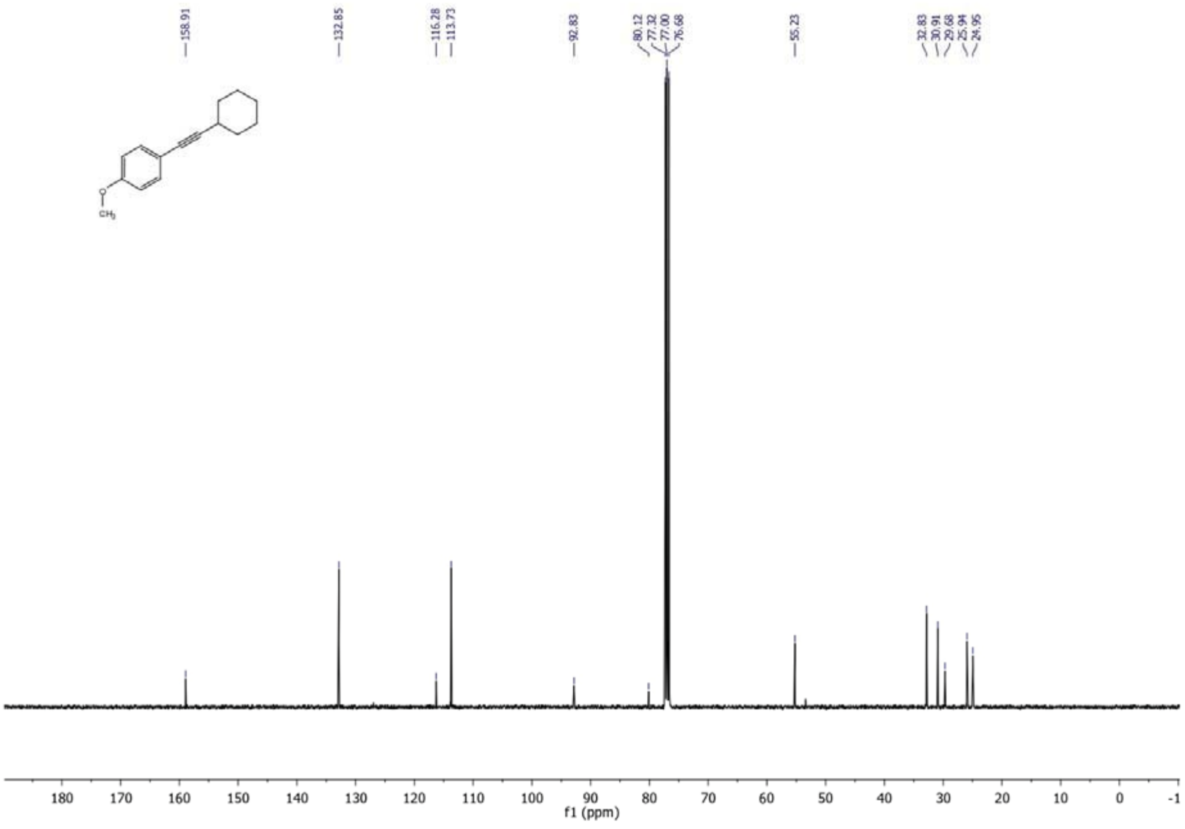

1,2-diphenylethyne (2f)

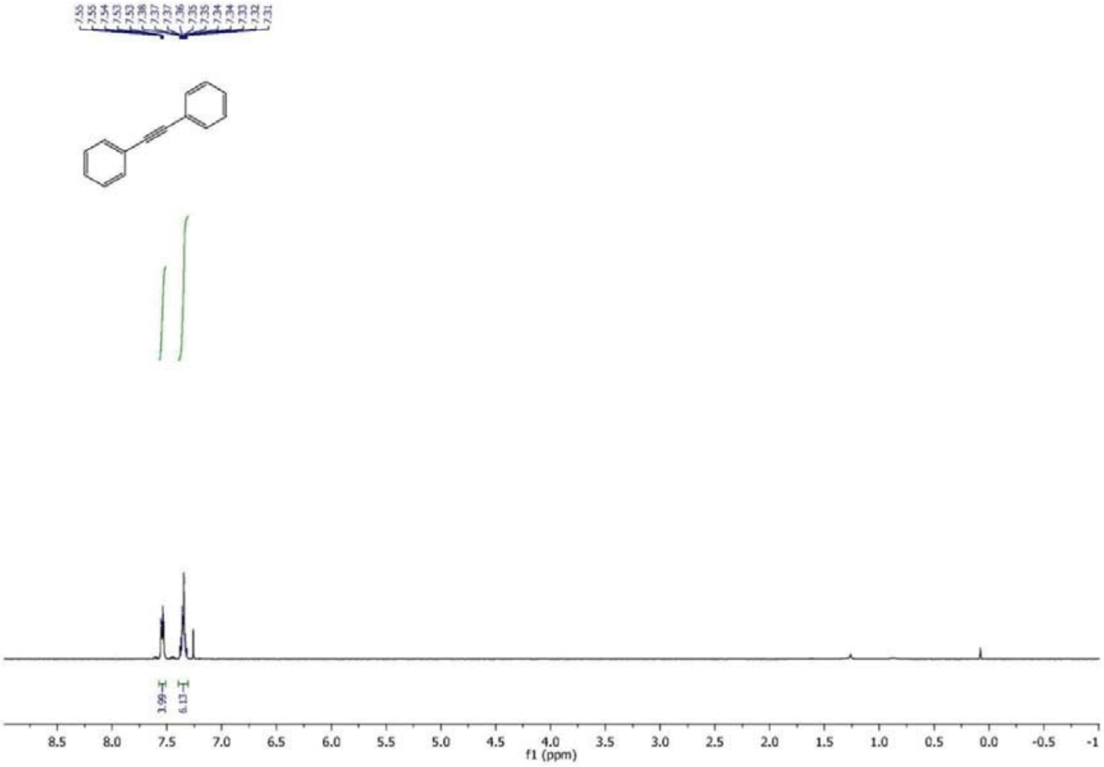

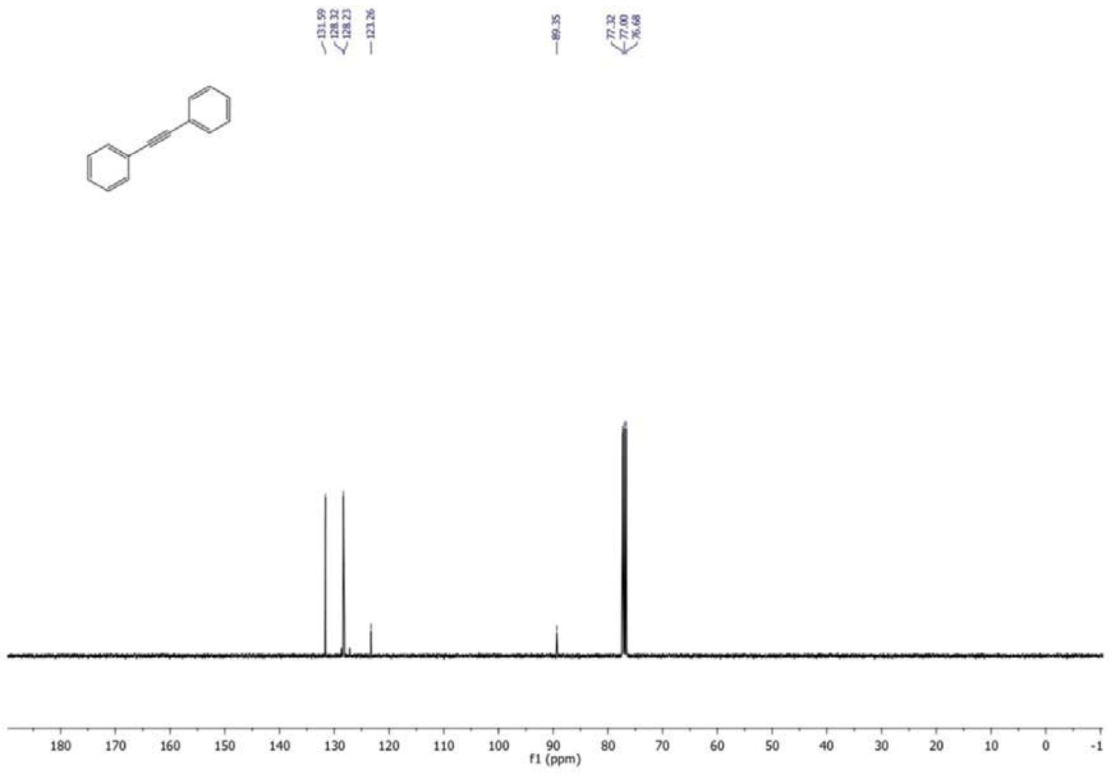

pent-1-yn-1-ylbenzene (2g)

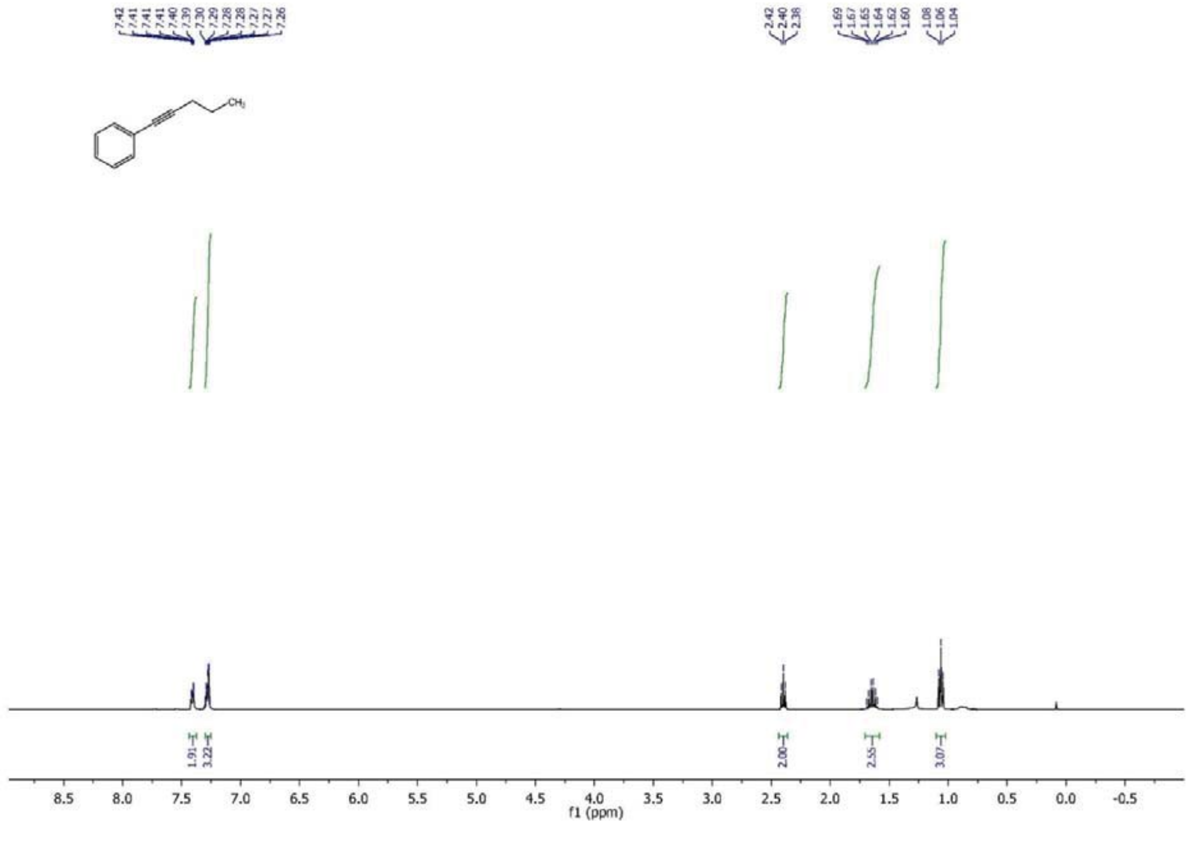

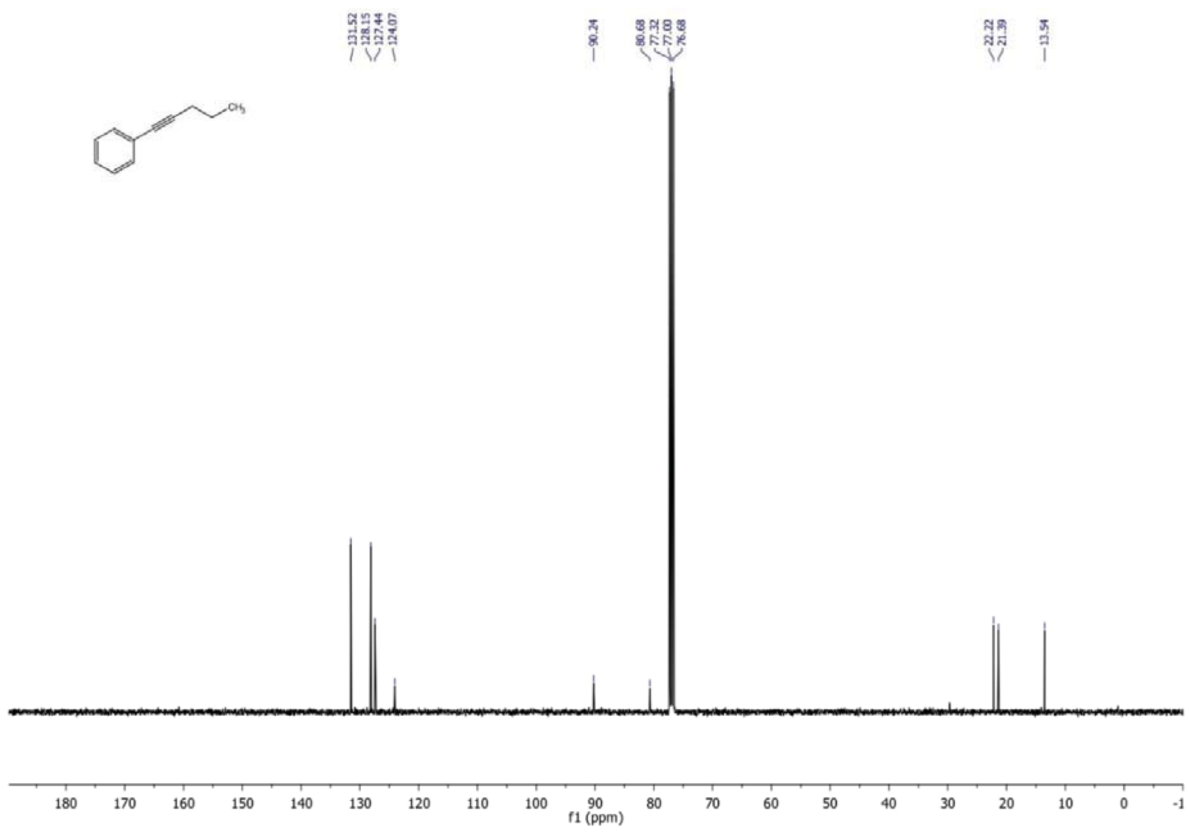

prop-1-yn-1-ylbenzene (2h)

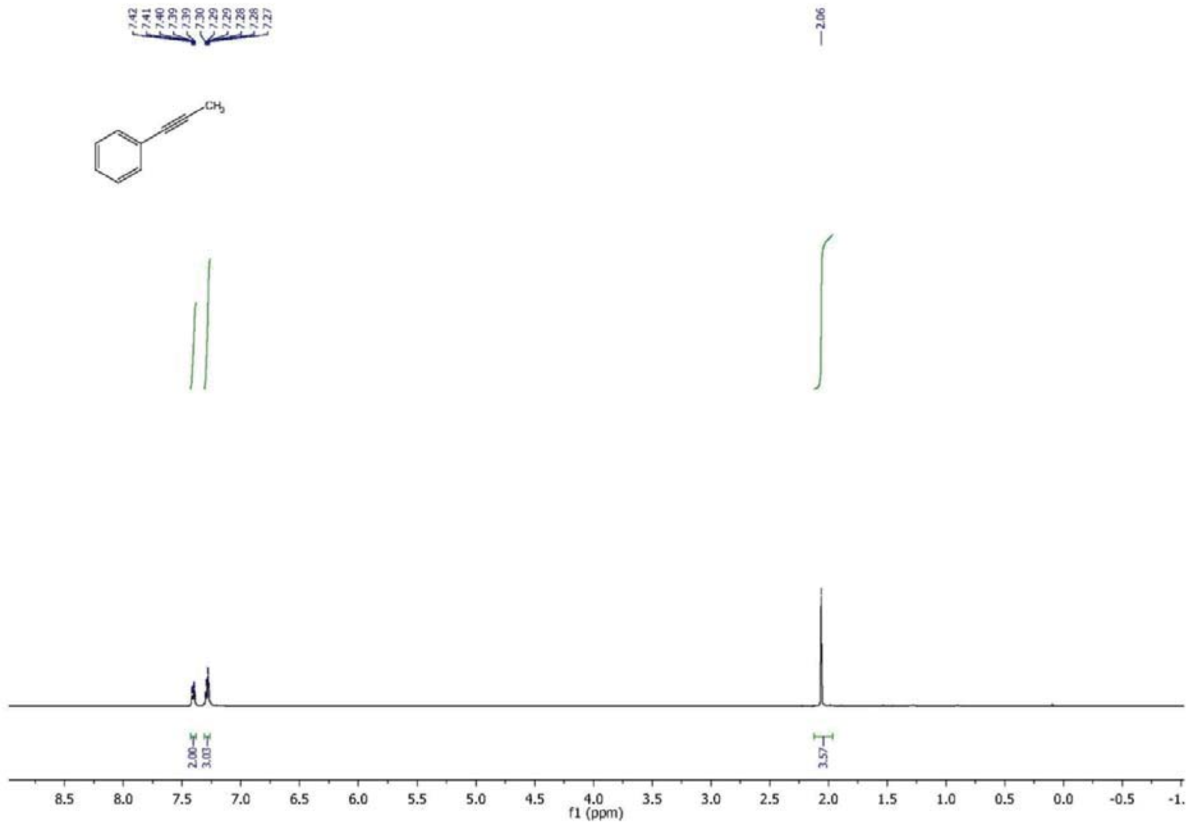

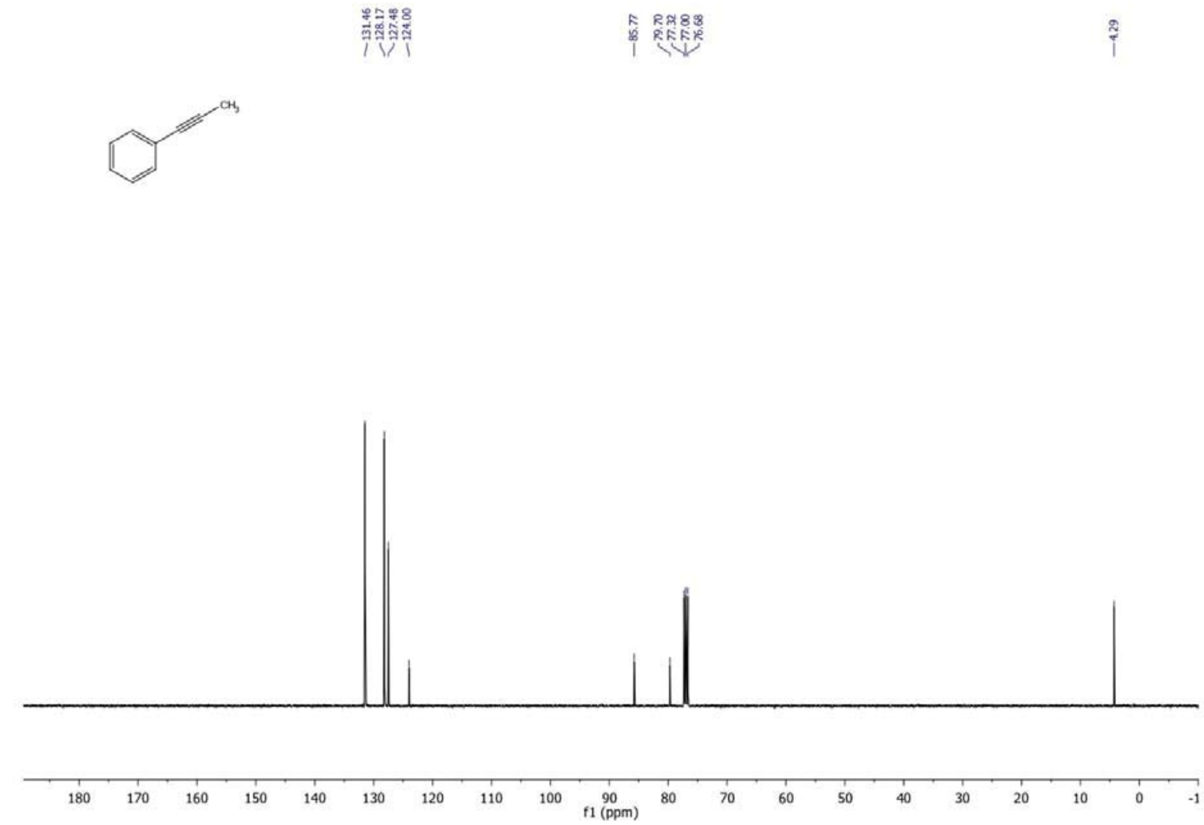

trimethyl(3-phenylprop-2-yn-1-yl)silane (2i)

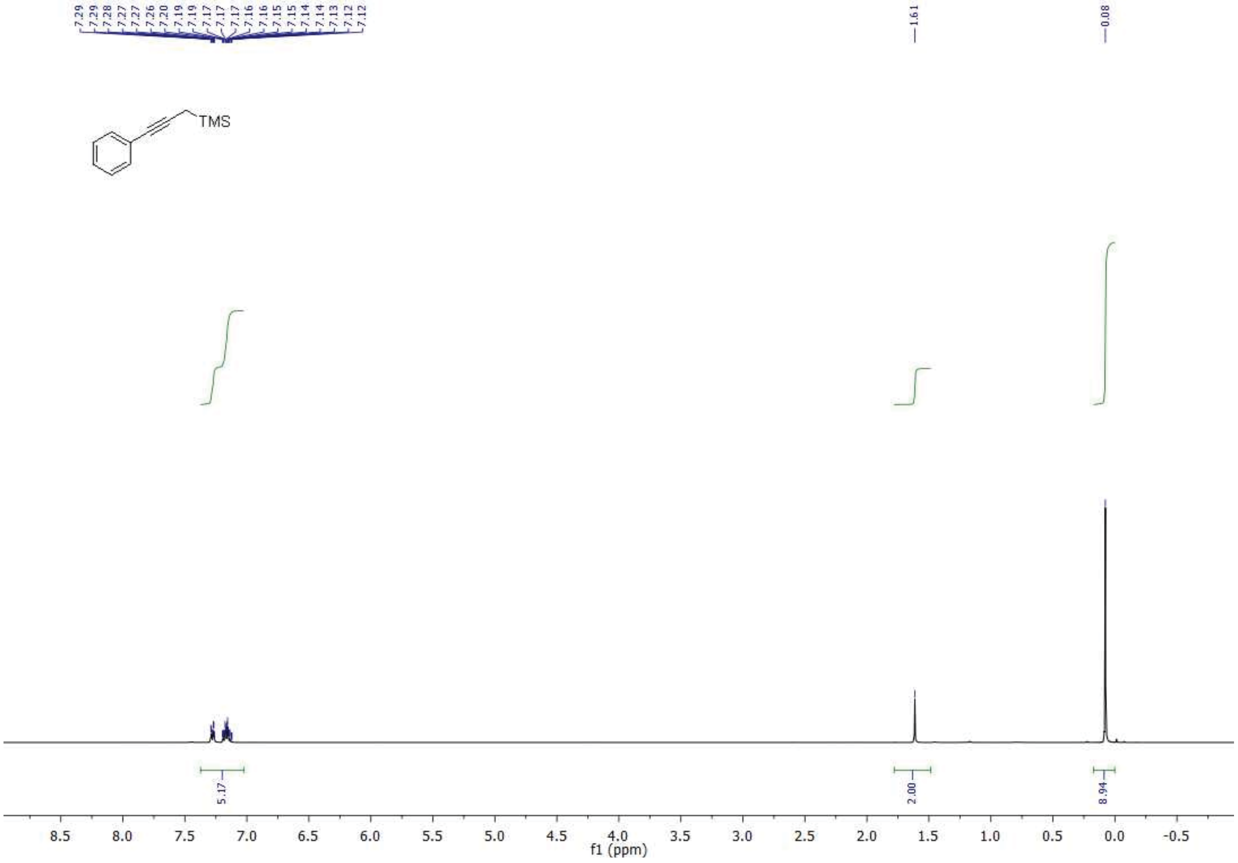

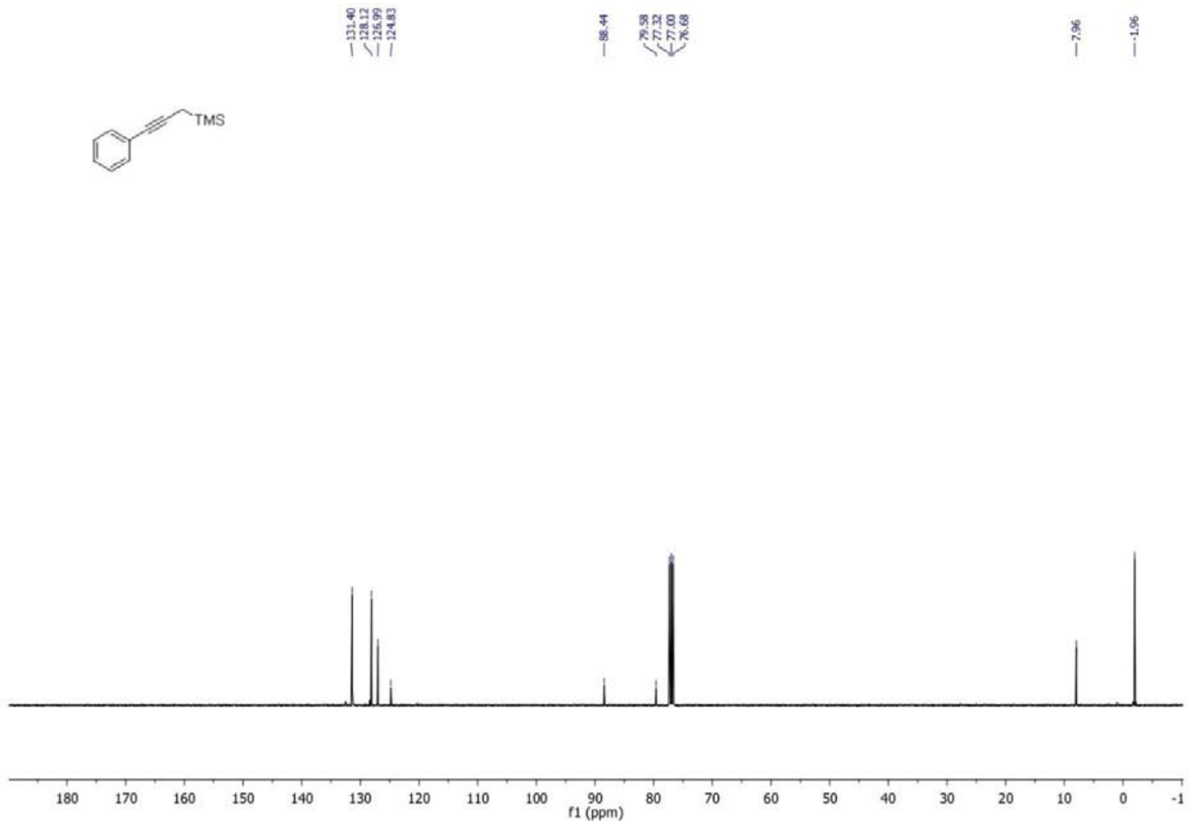

(cyclopentylethynyl)benzene (2j)

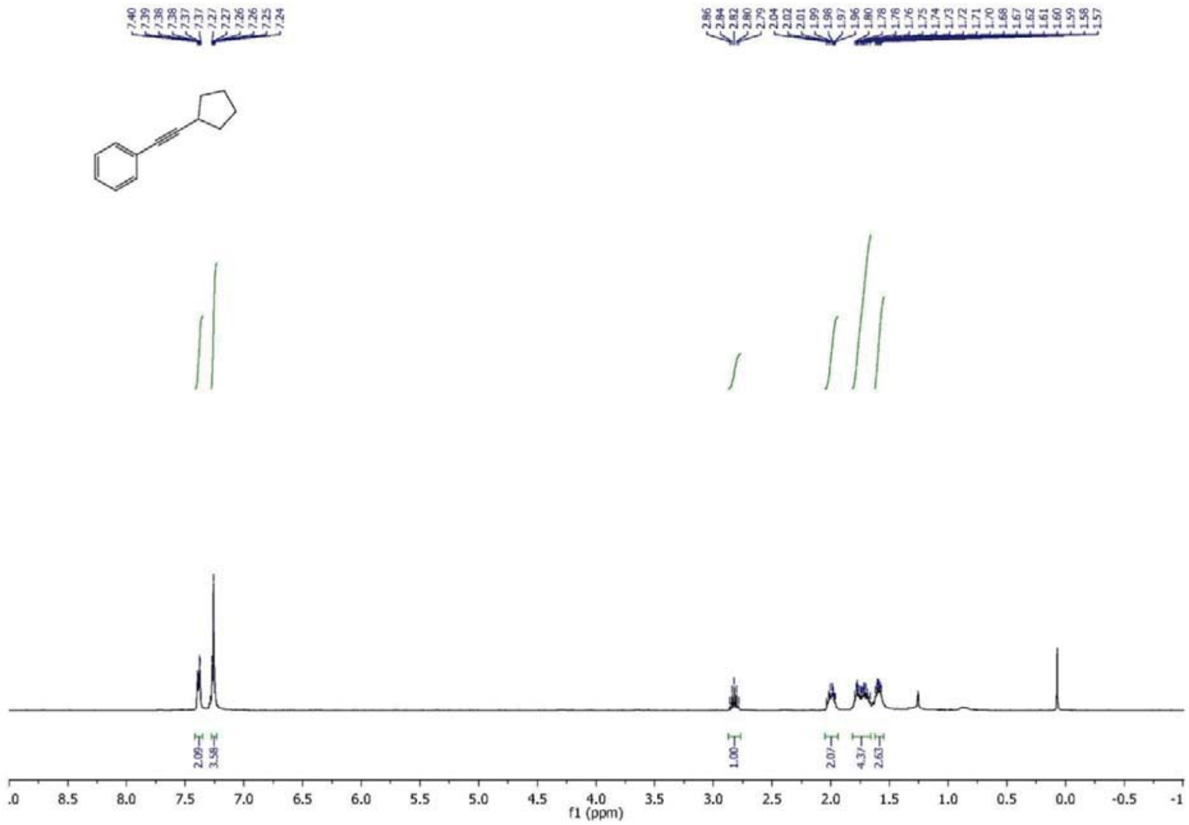

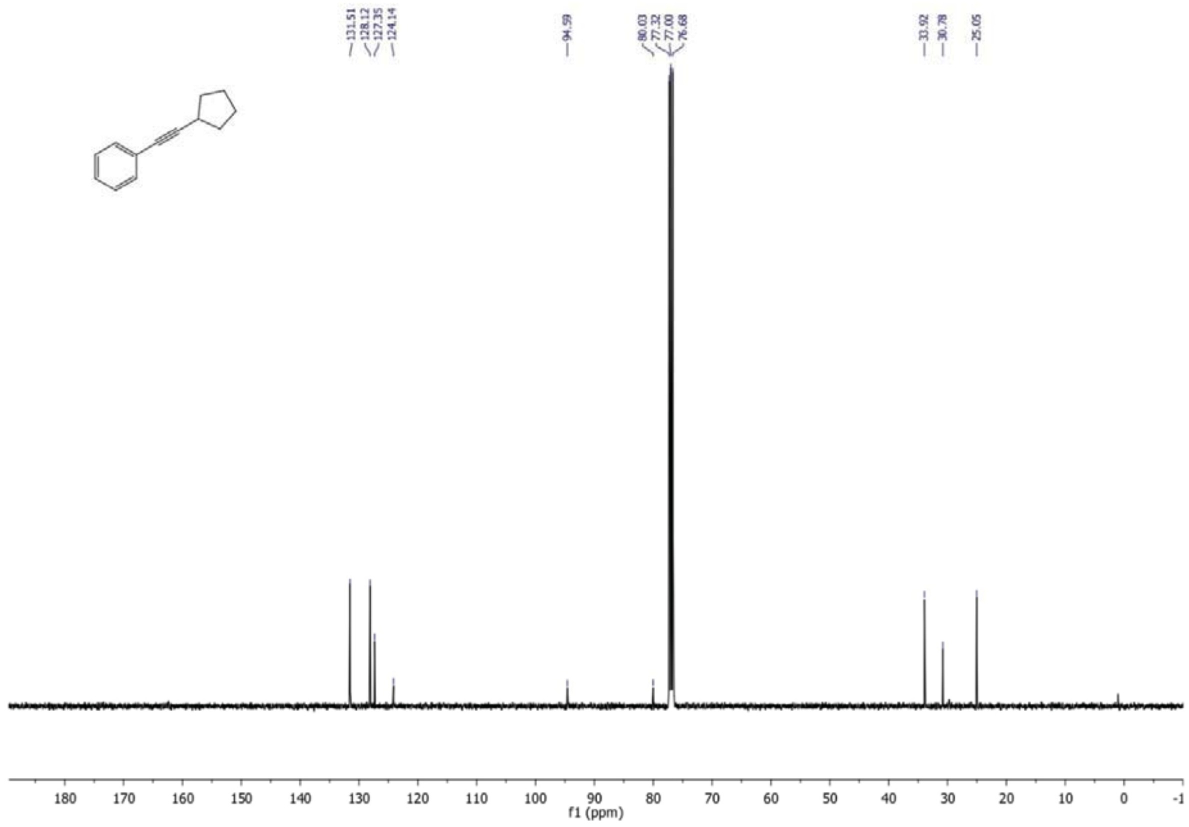

1-methyl-4-(phenylethynyl)piperidine (2k)

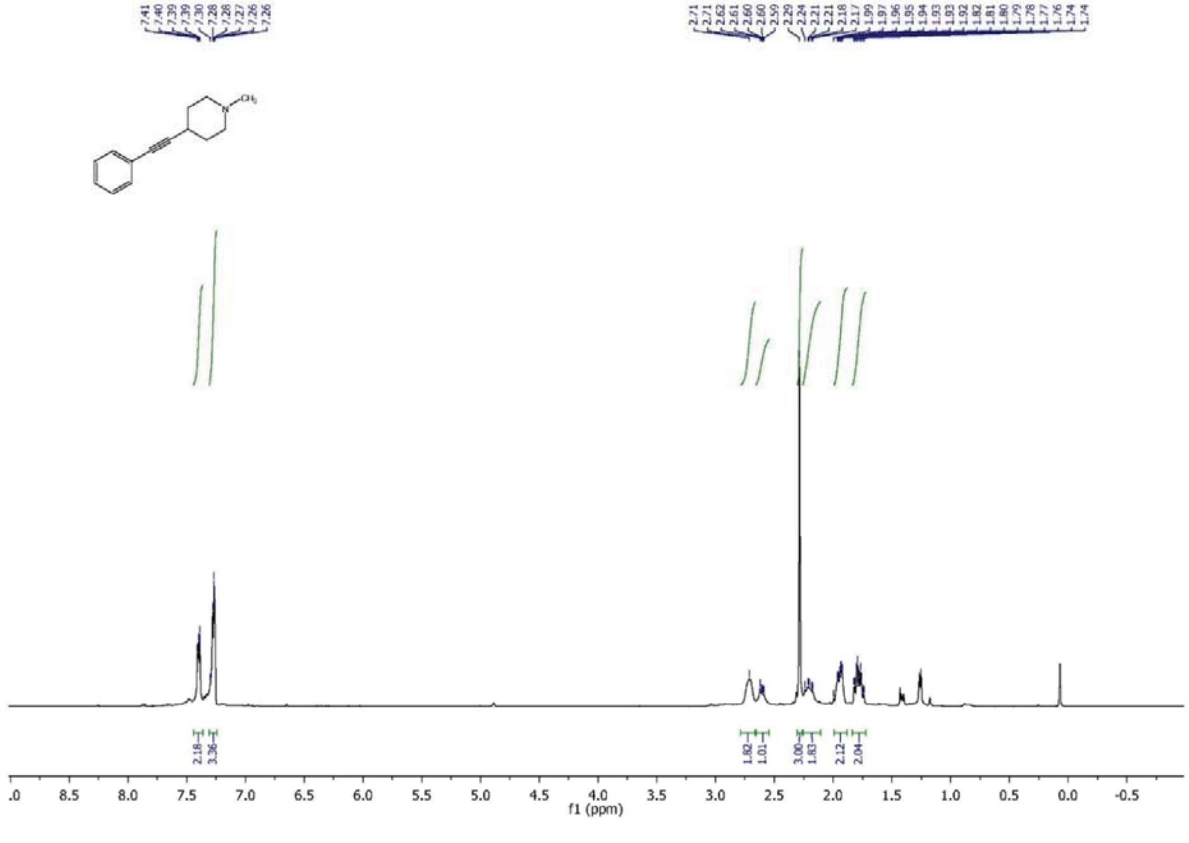

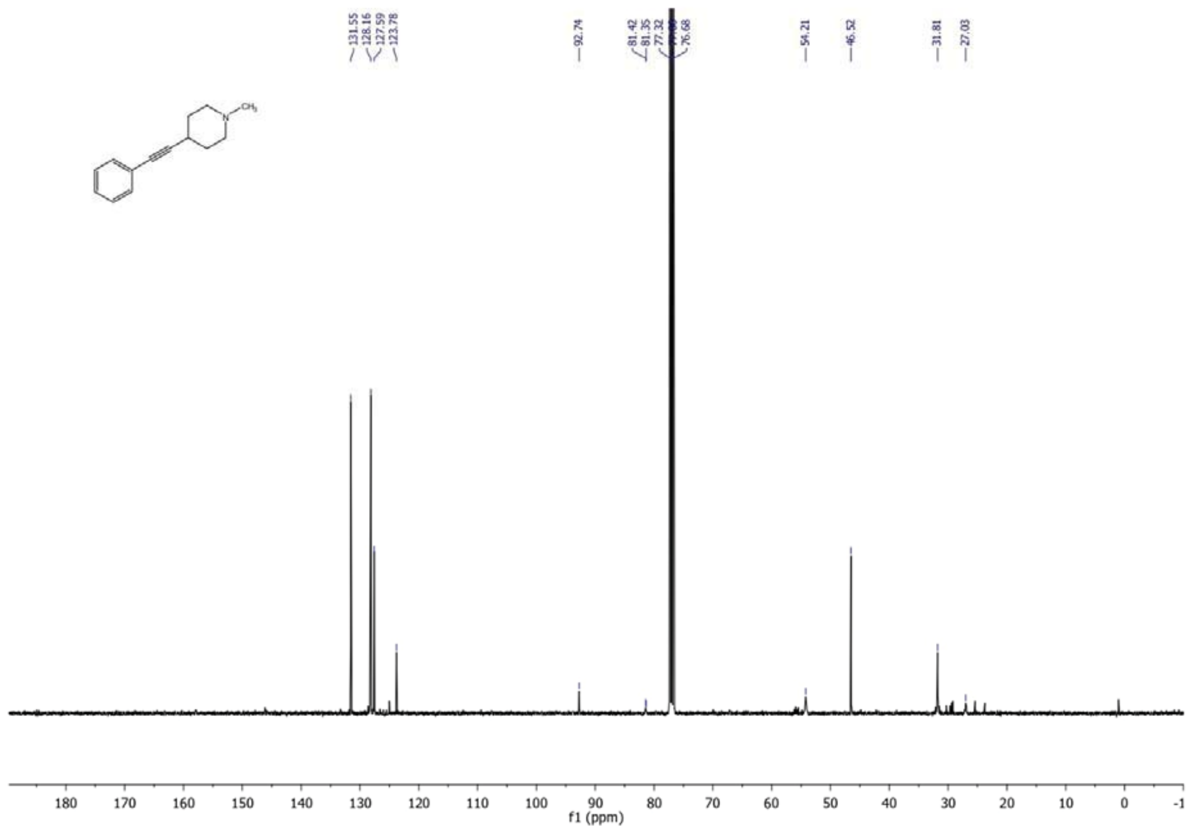

GC-MS spectra of 2k

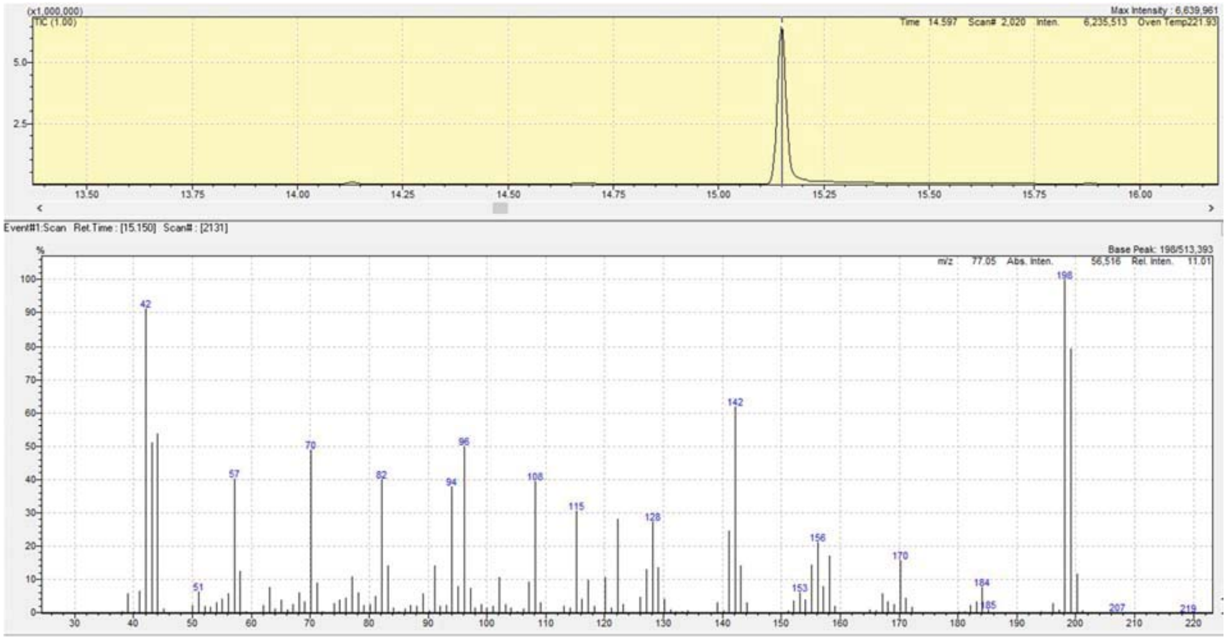

oct-1-yn-1-ylbenzene (2l)

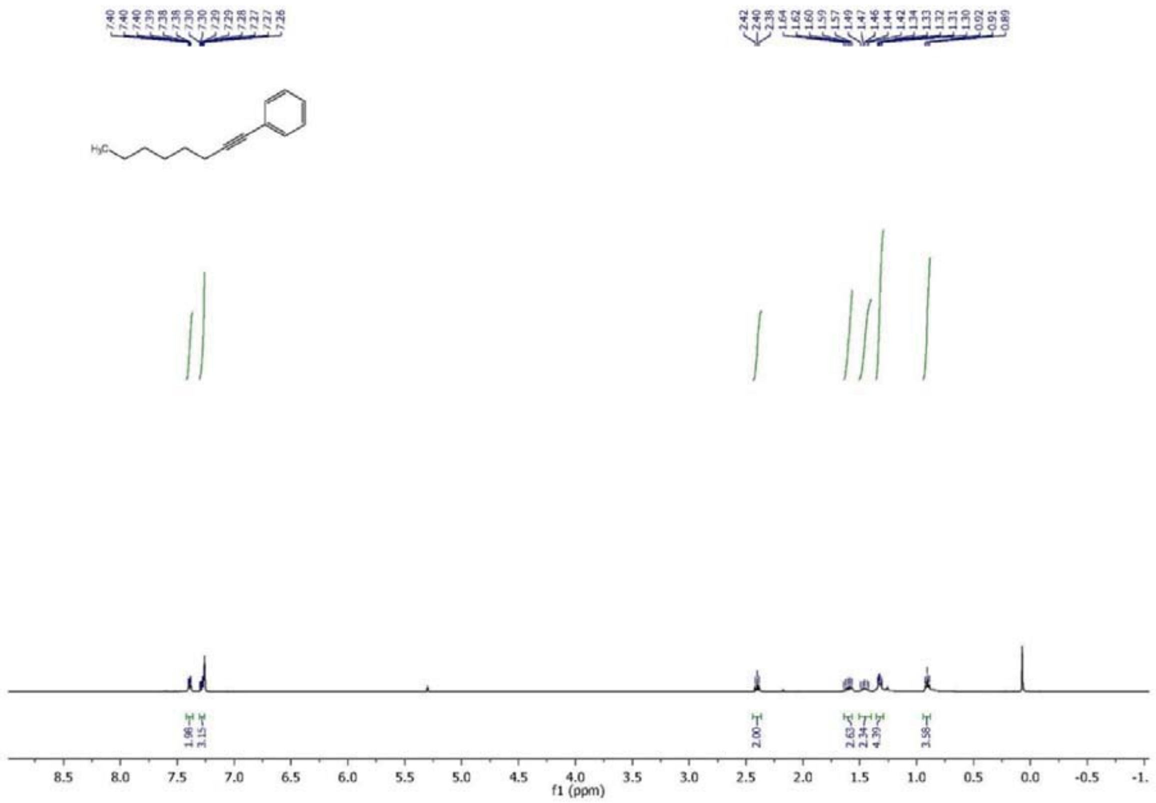

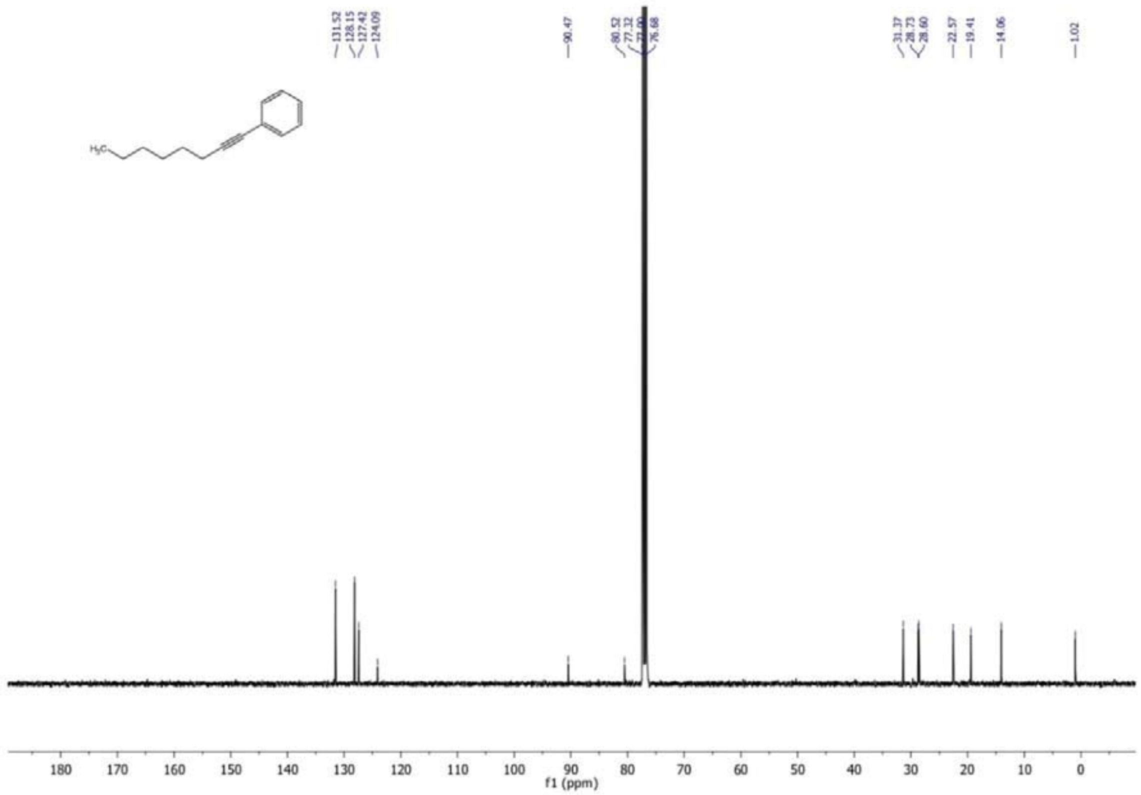

***(E)*-prop-1-en-1-ylbenzene (4a)**

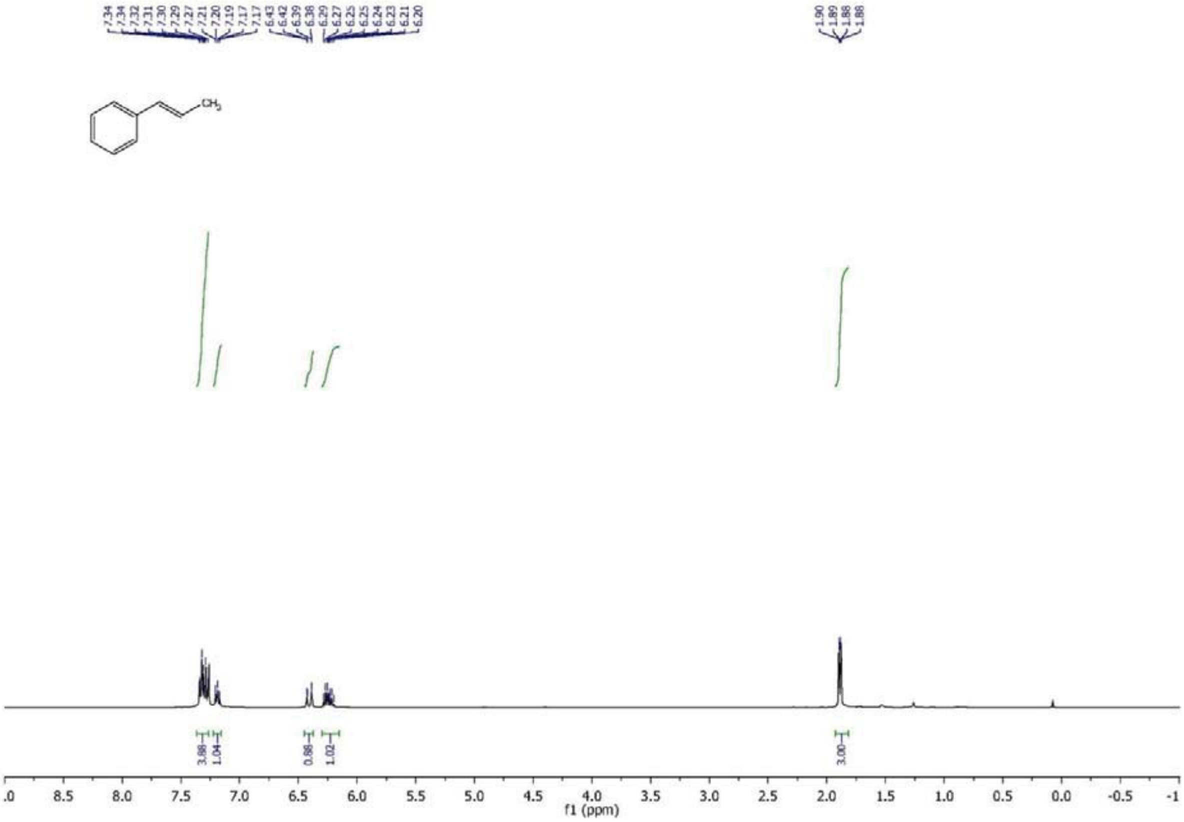

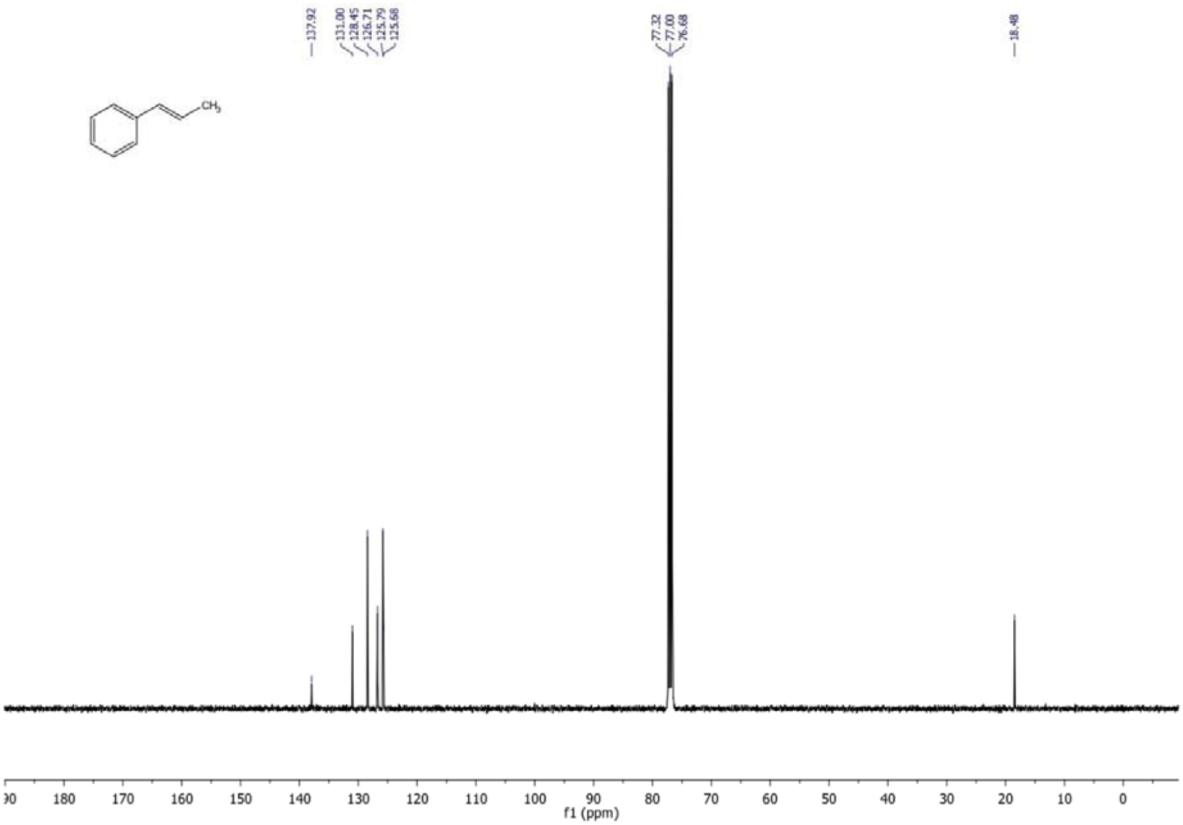

***(E)*-pent-1-en-1-ylbenzene (4b)**

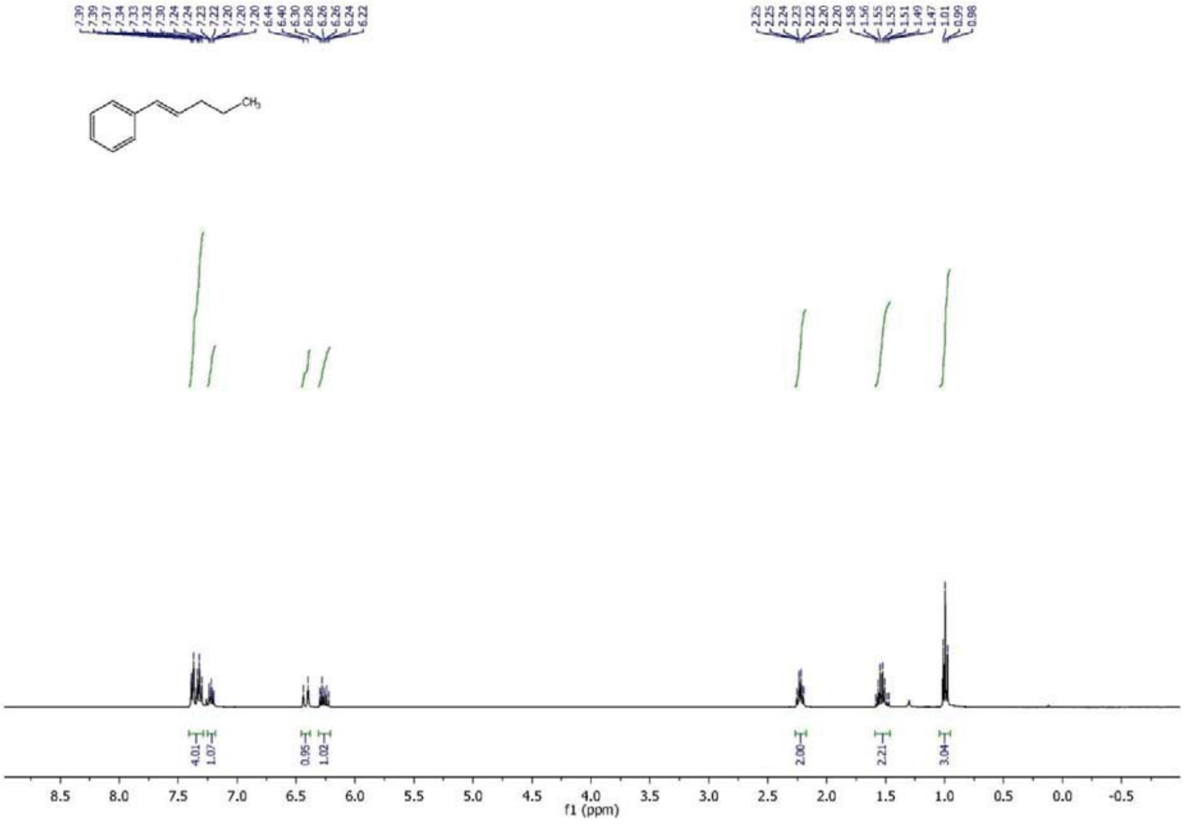

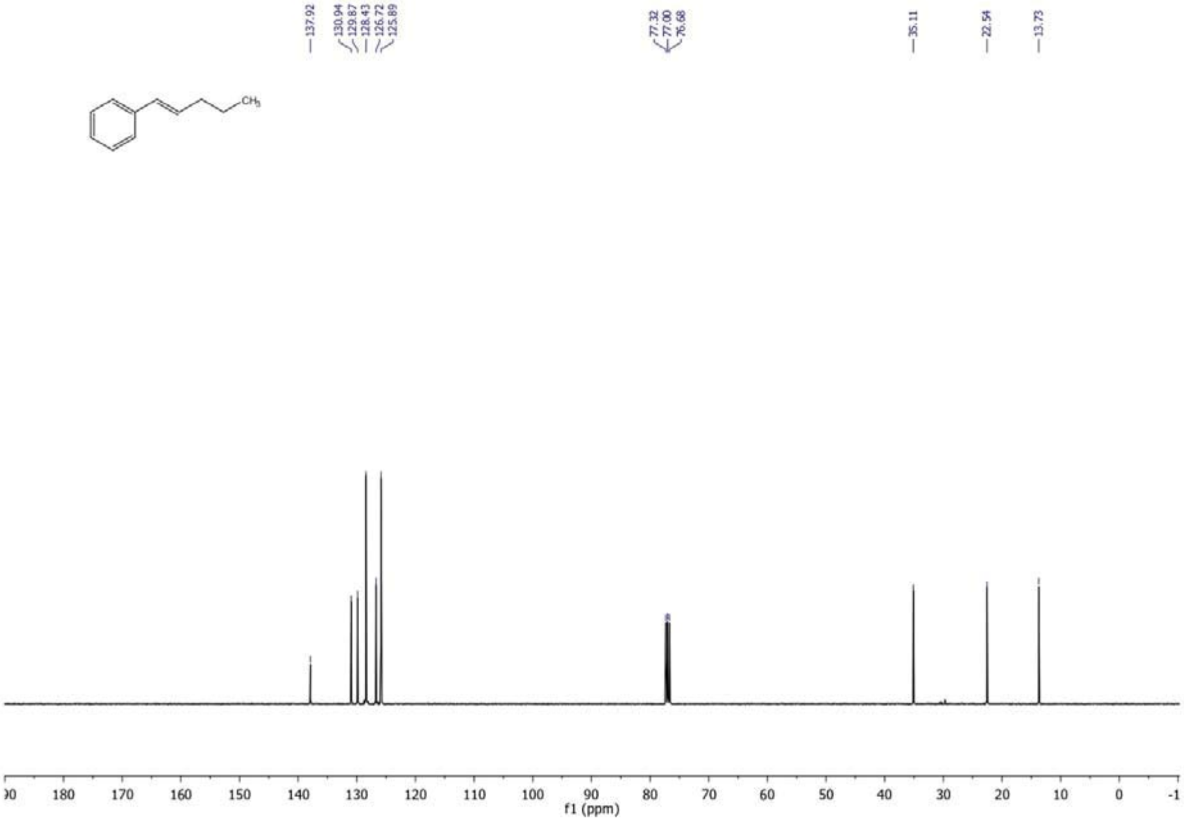

***(E)*-prop-1-ene-1,3-diyldibenzene (4c)**

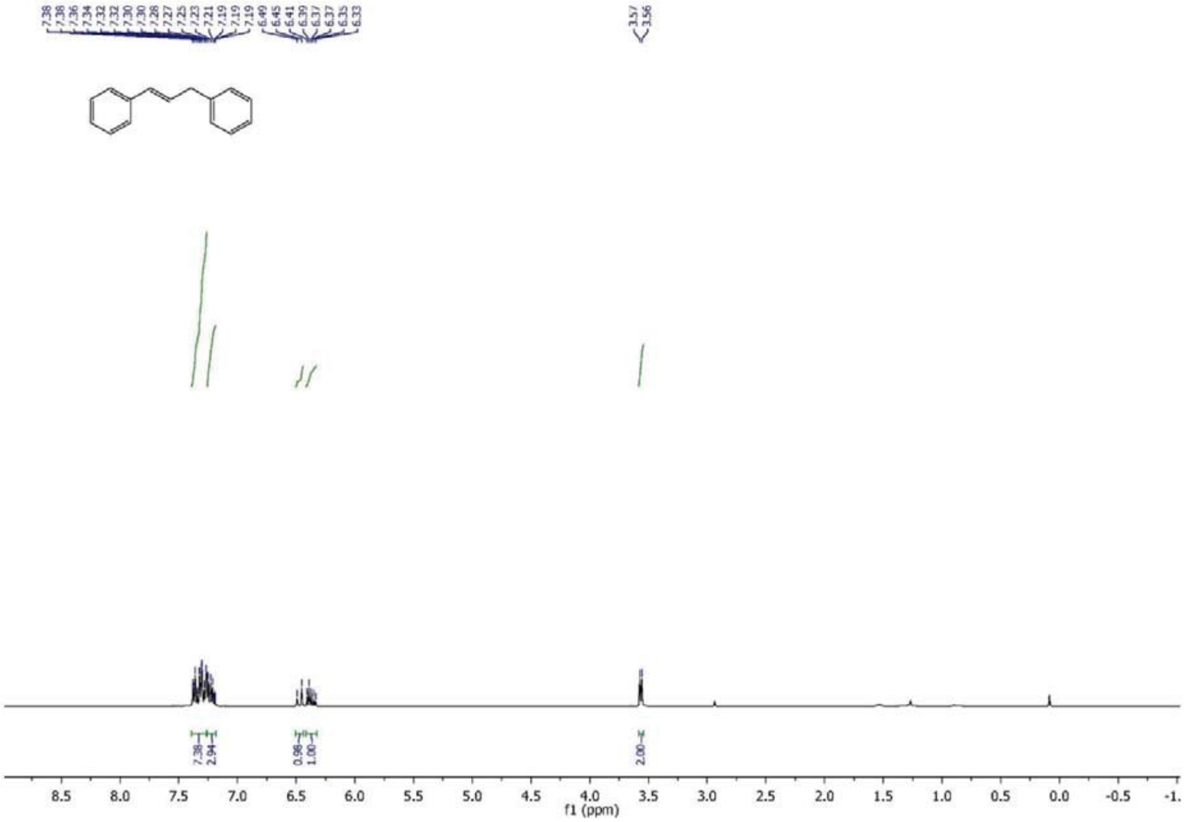

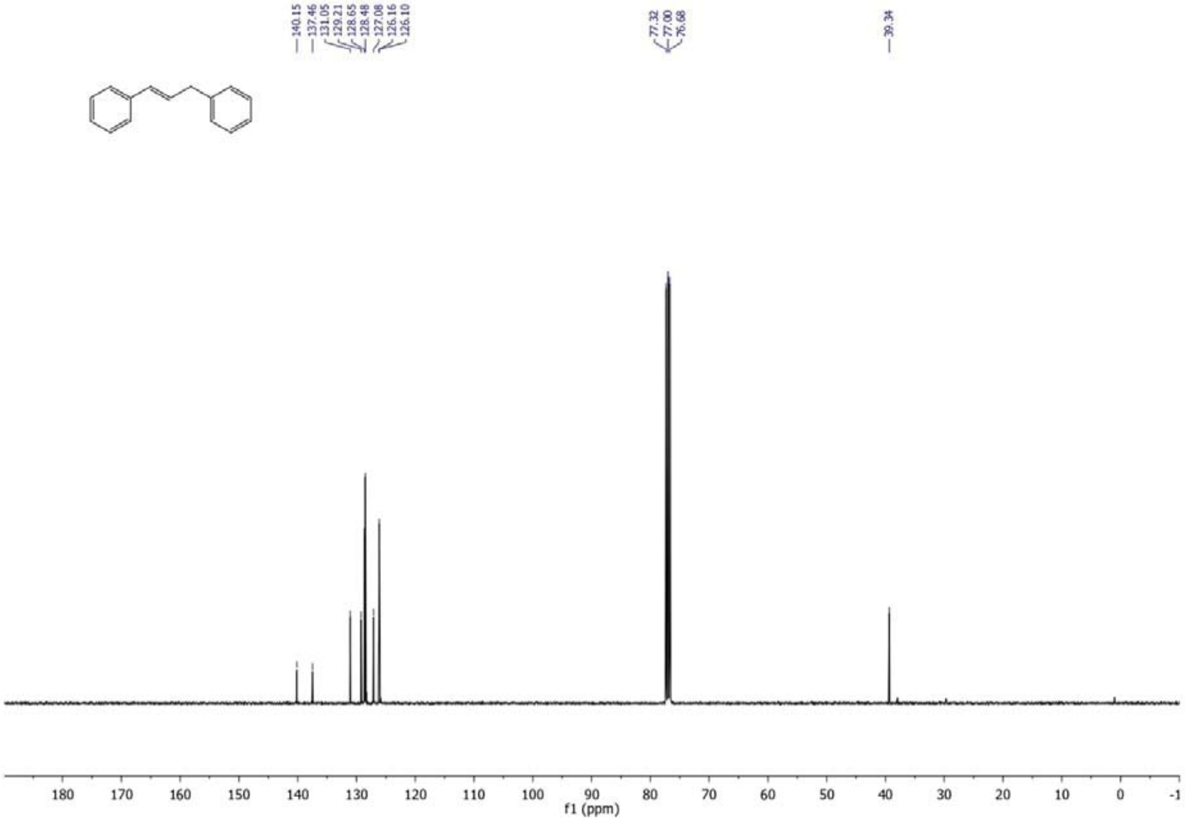

(*E*)-(3-methylbut-1-en-1-yl)benzene (4d)

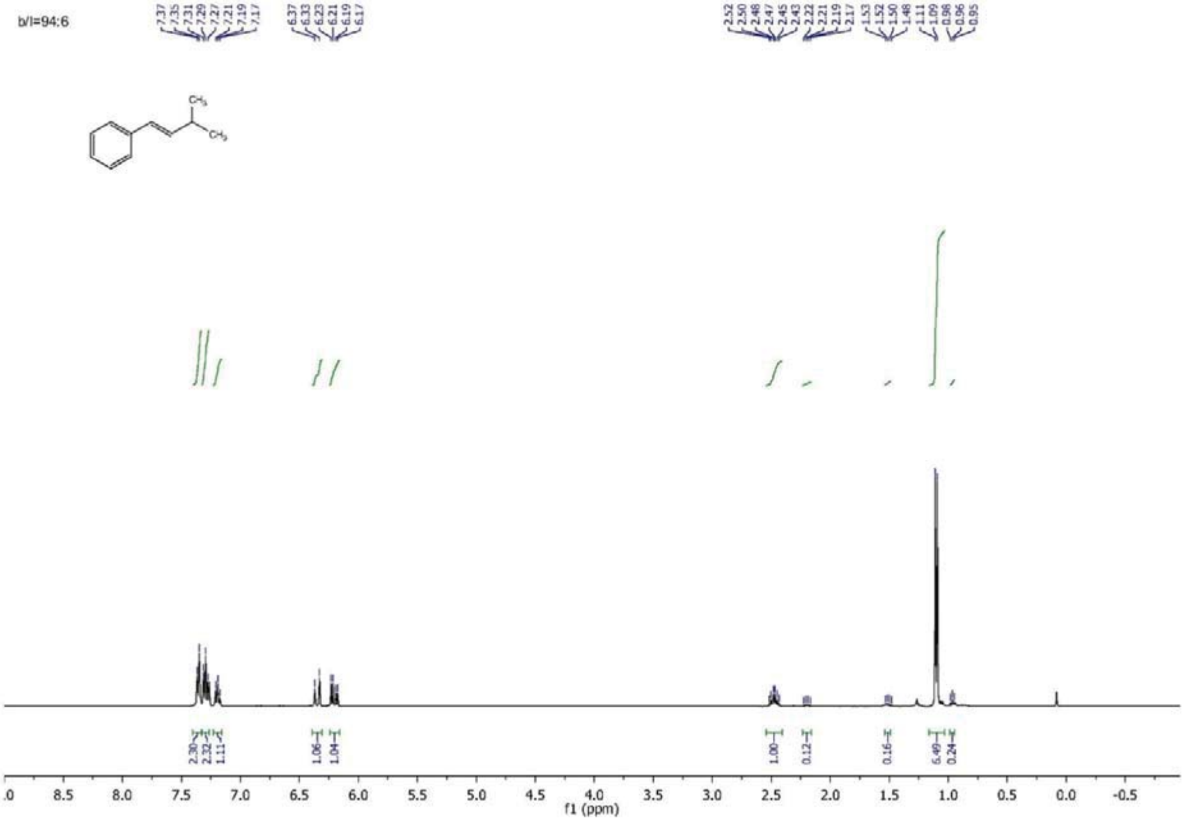

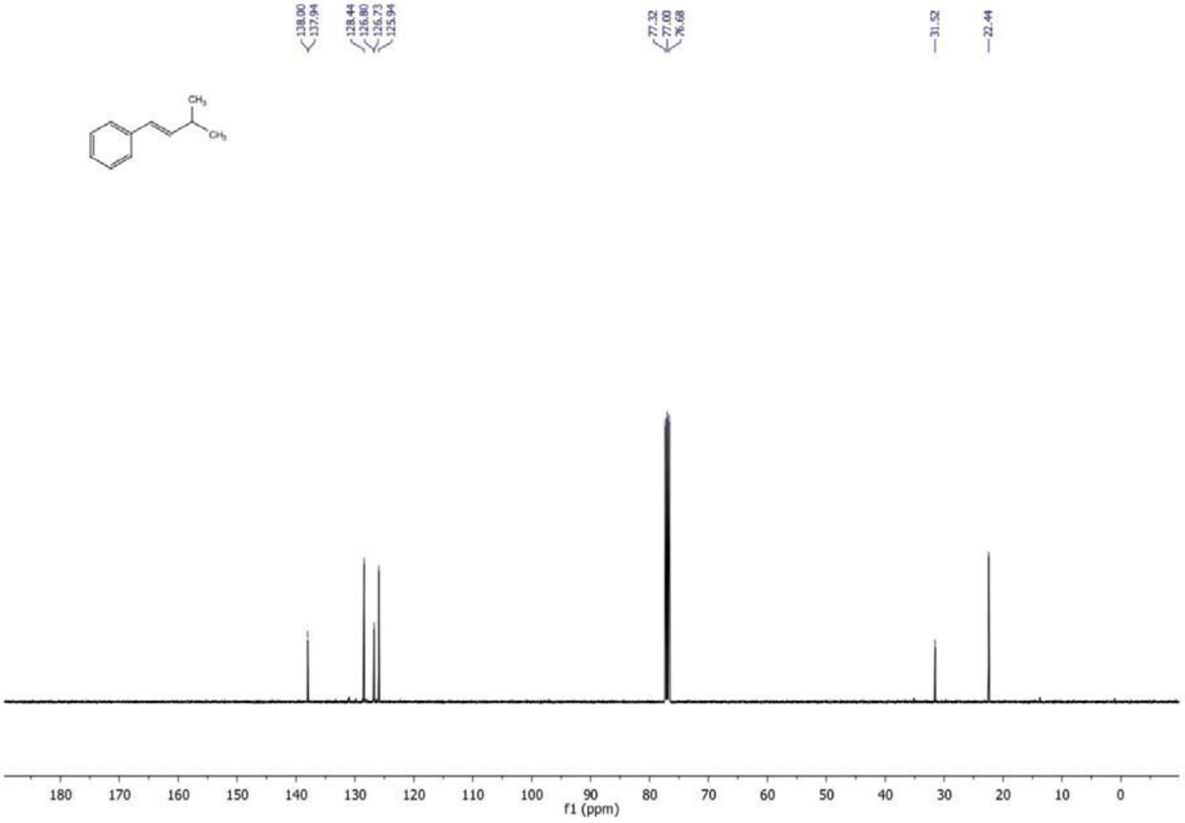

***(E)*-(2-cyclopentylvinyl)benzene (4e)**

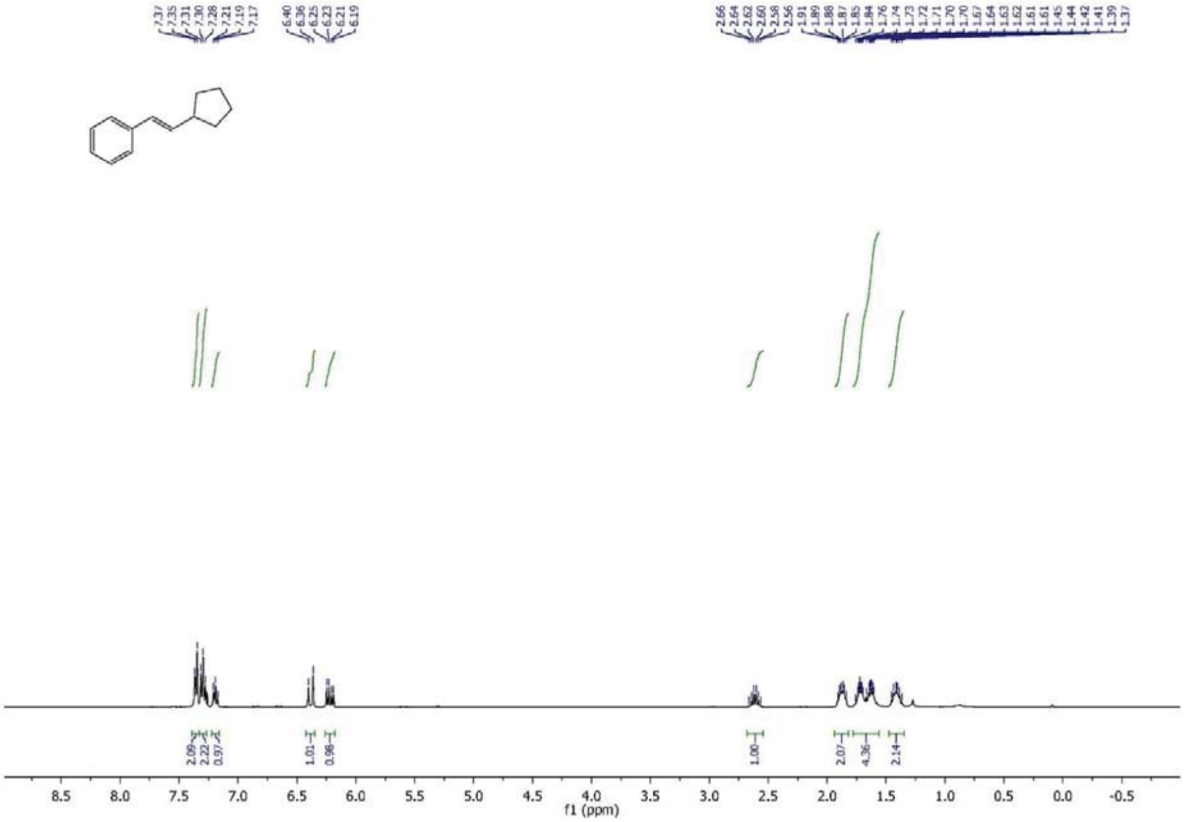

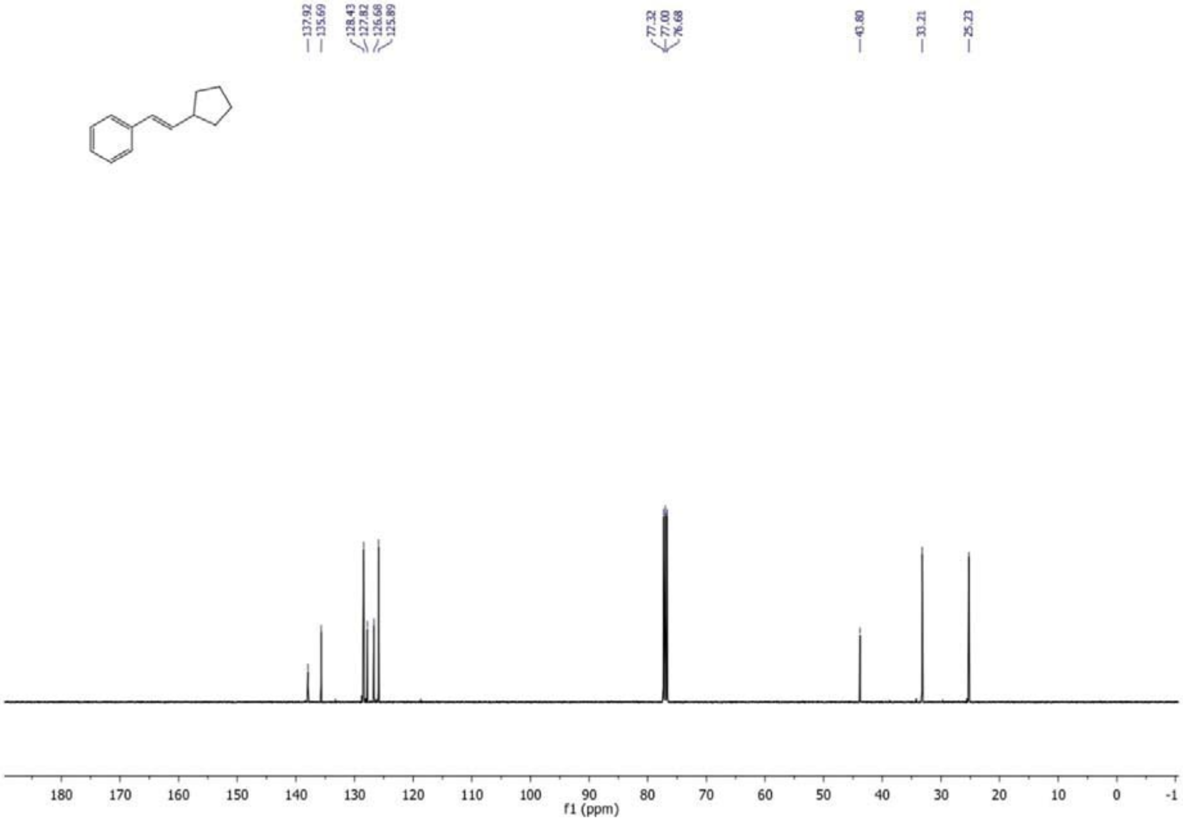

(E)-(2-cyclohexylvinyl)benzene (4f)

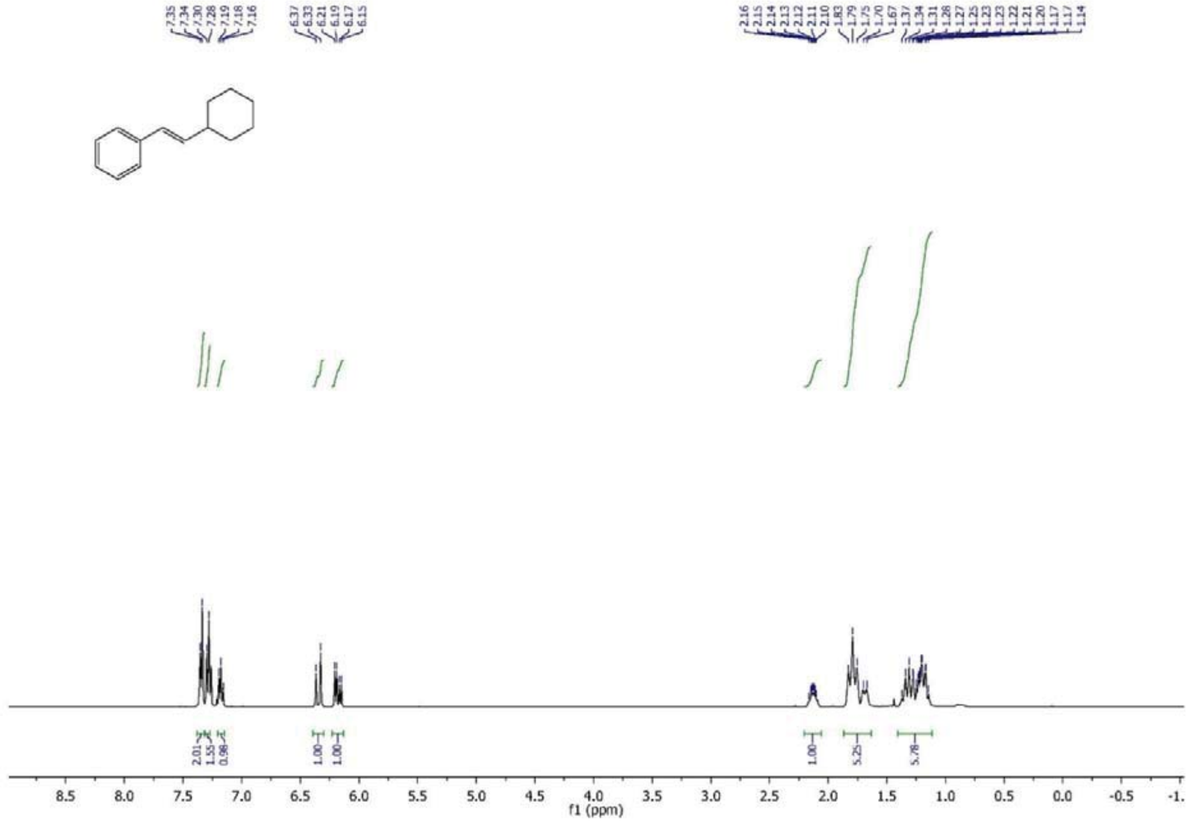

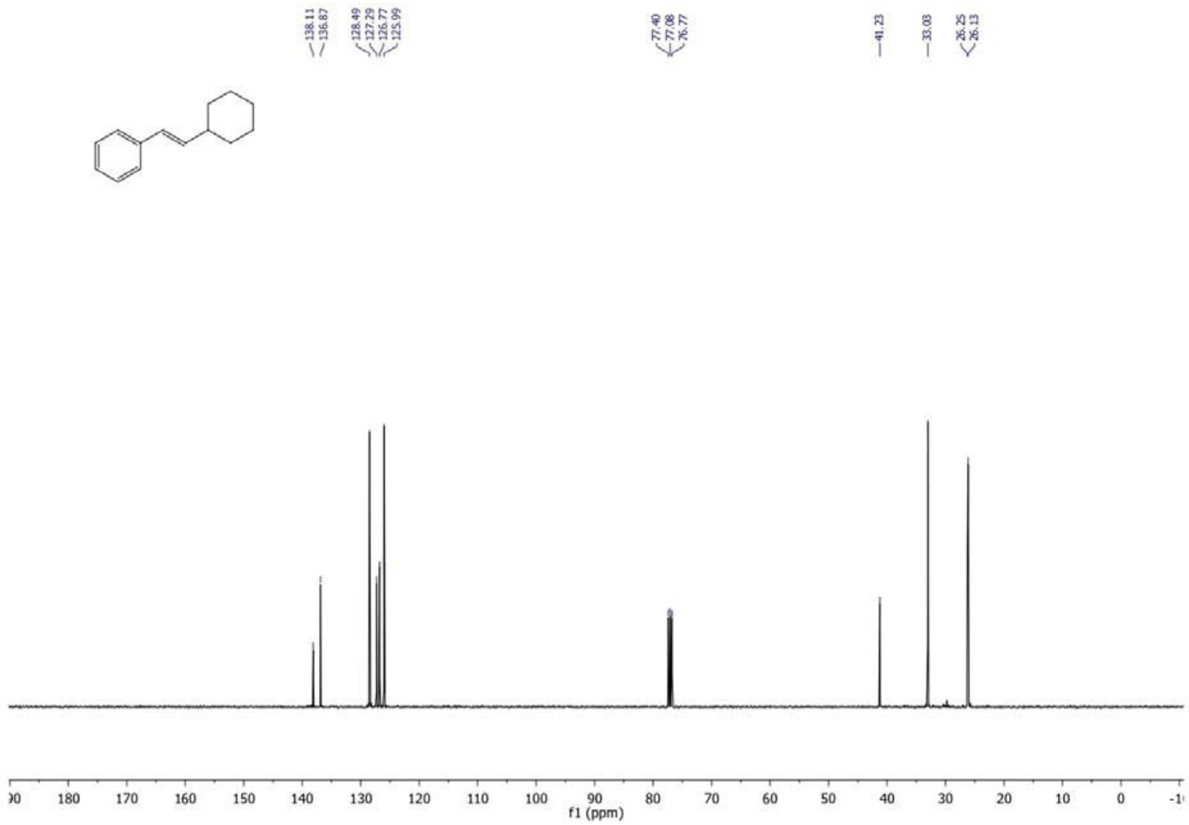

(E)-1-methyl-4-styrylpiperidine (4g)

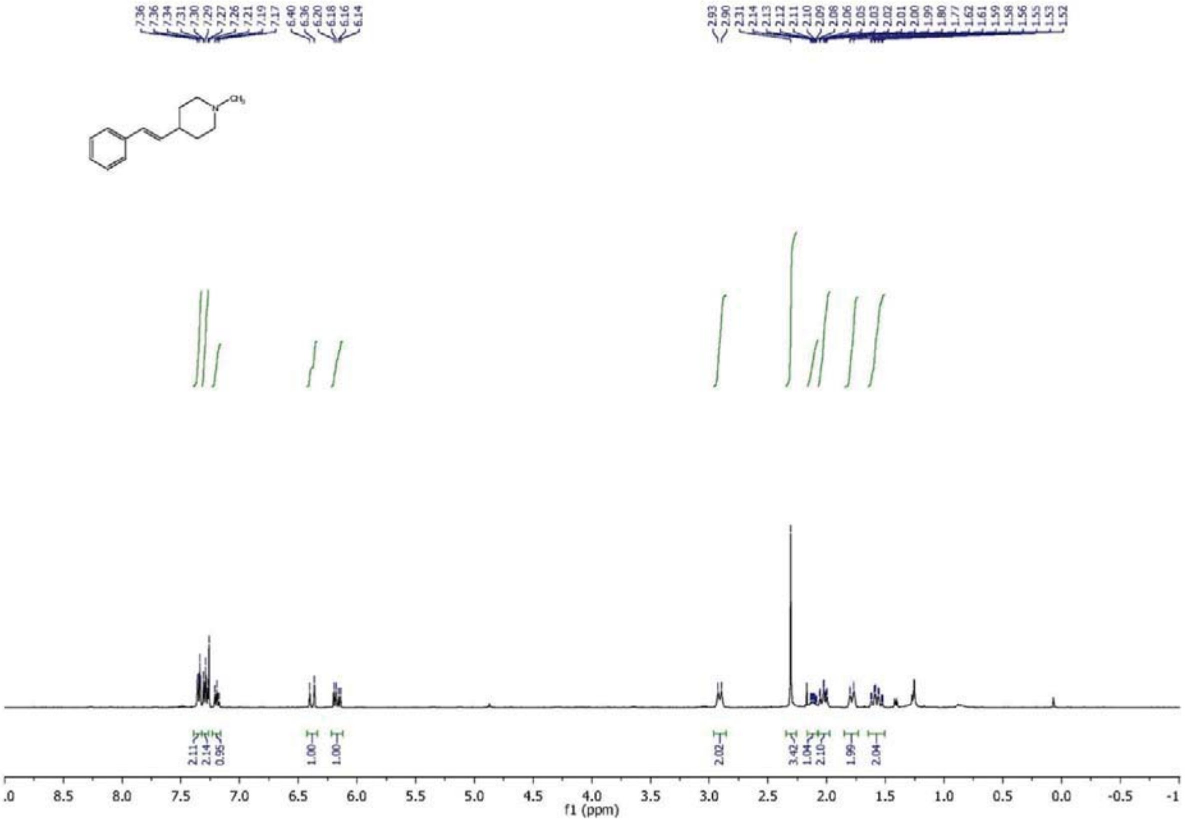

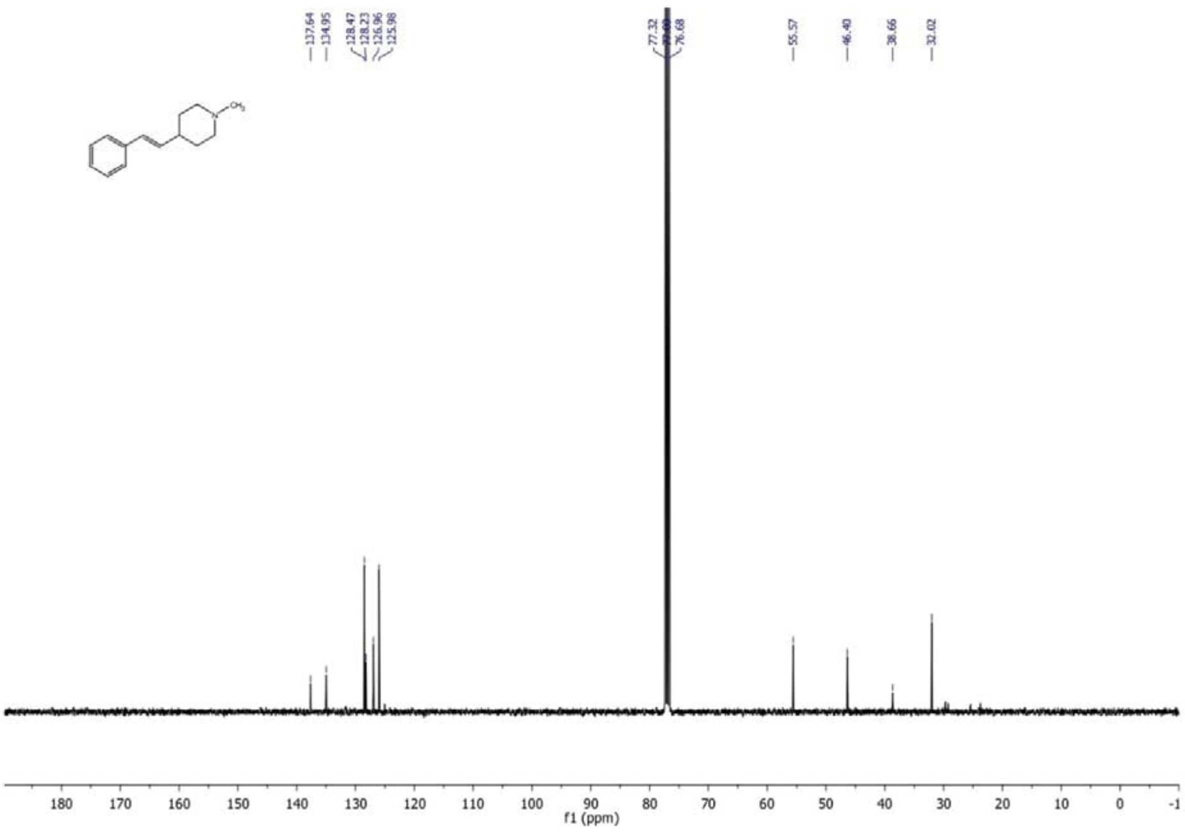

(*E*)-1,2-diphenylethene (4h)

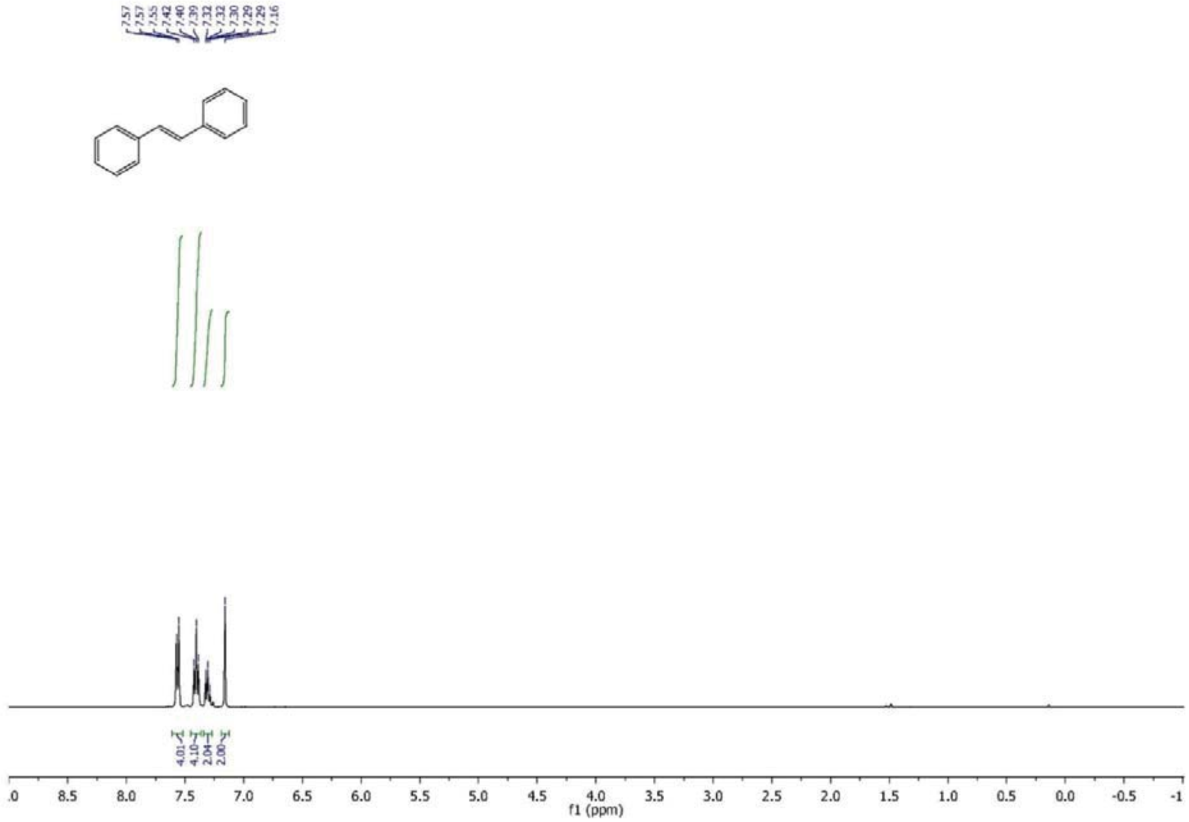

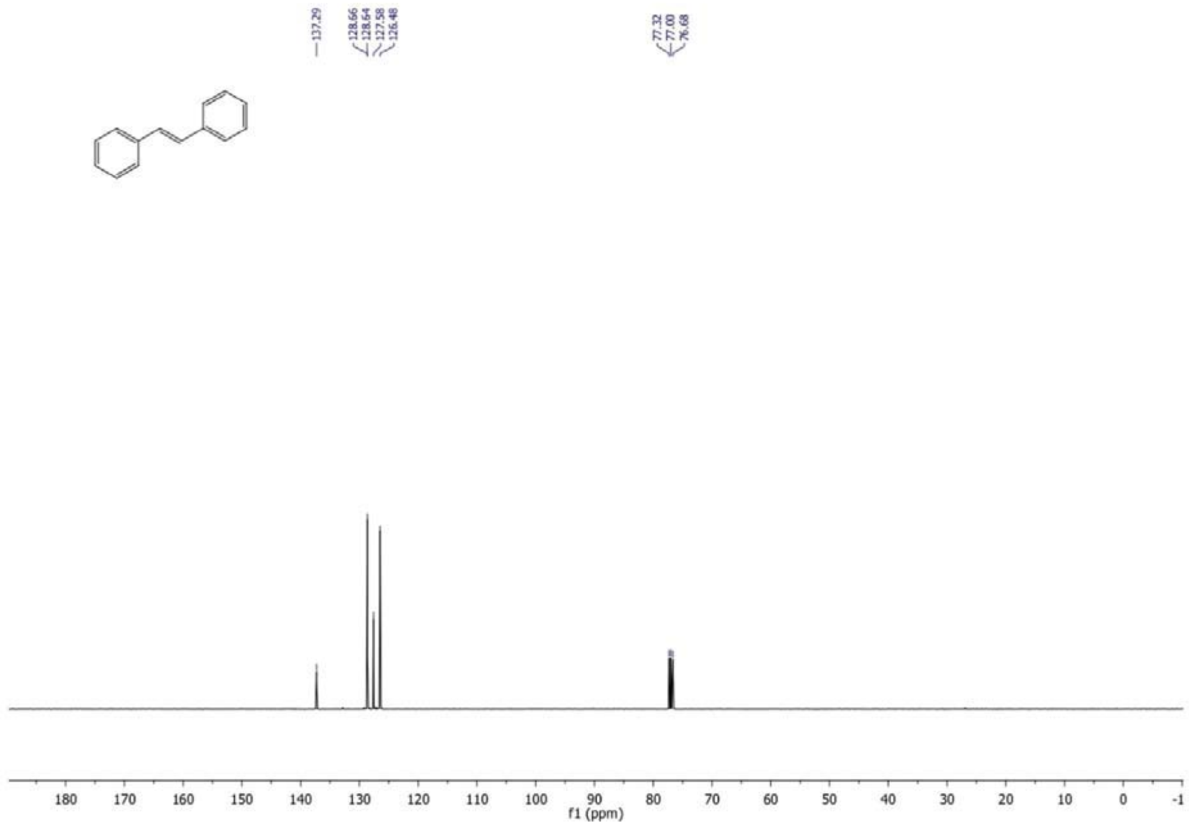

***(E)*-1-(2-cyclohexylvinyl)-2-methylbenzene (4i)**

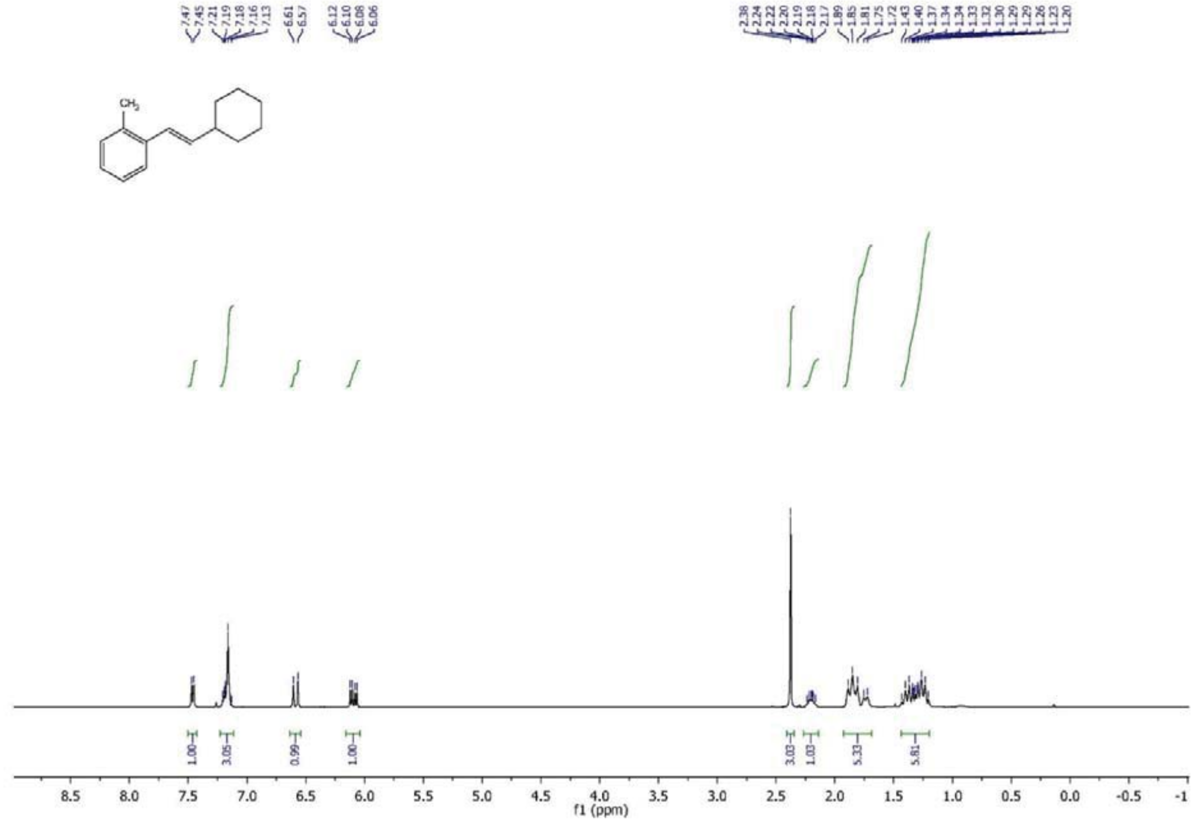

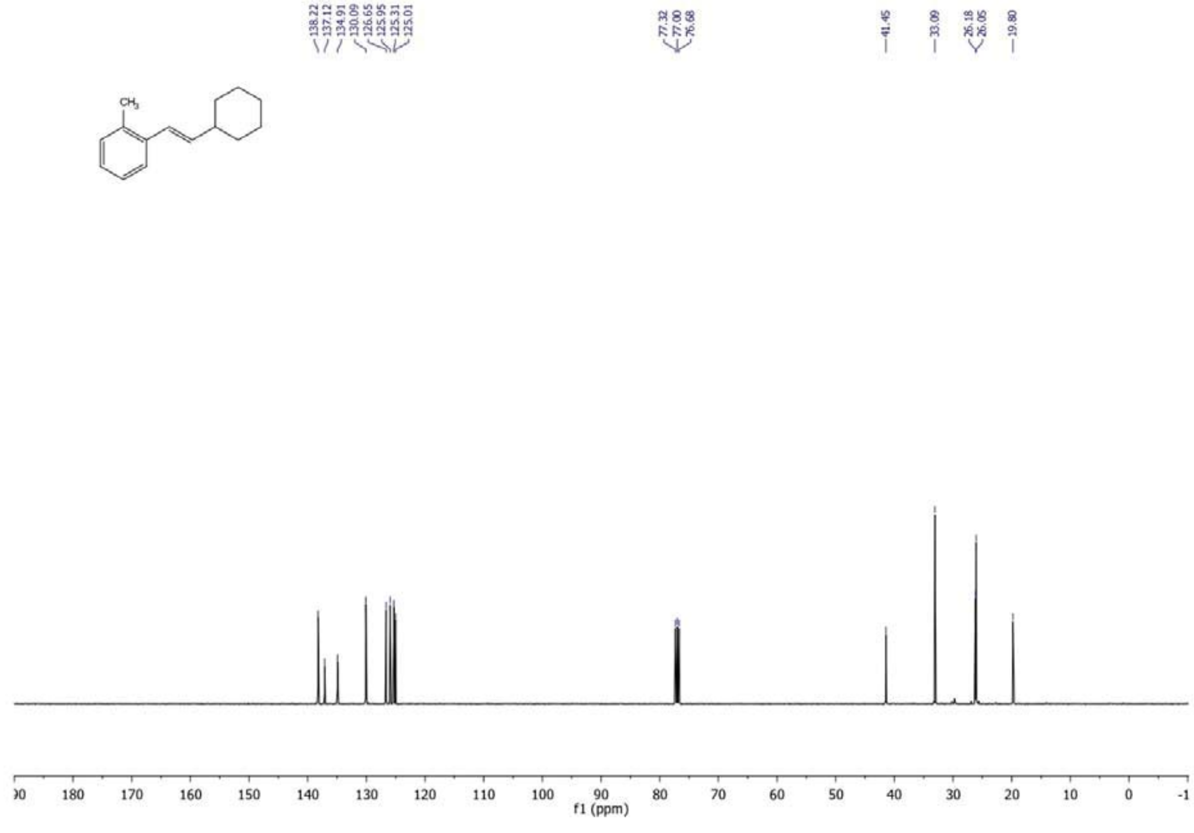

***(E)*-1-(2-cyclohexylvinyl)-4-isopropylbenzene (4j)**

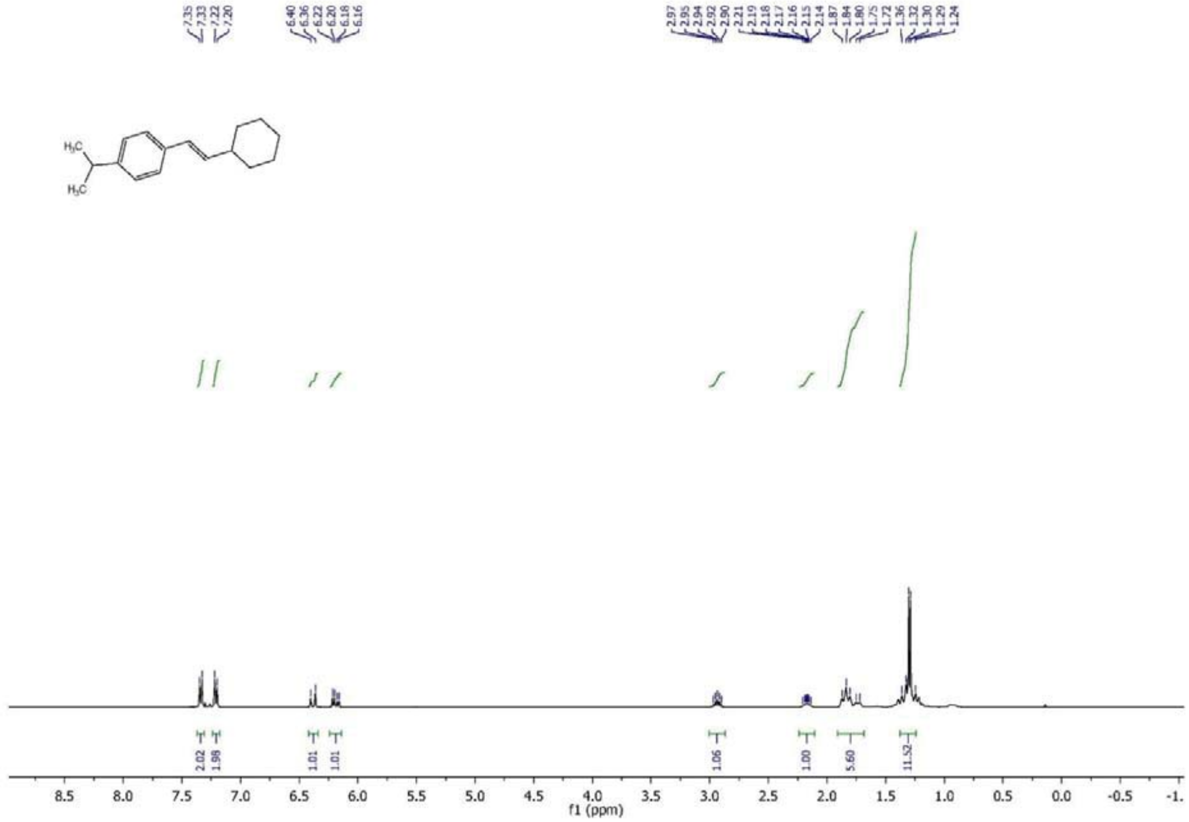

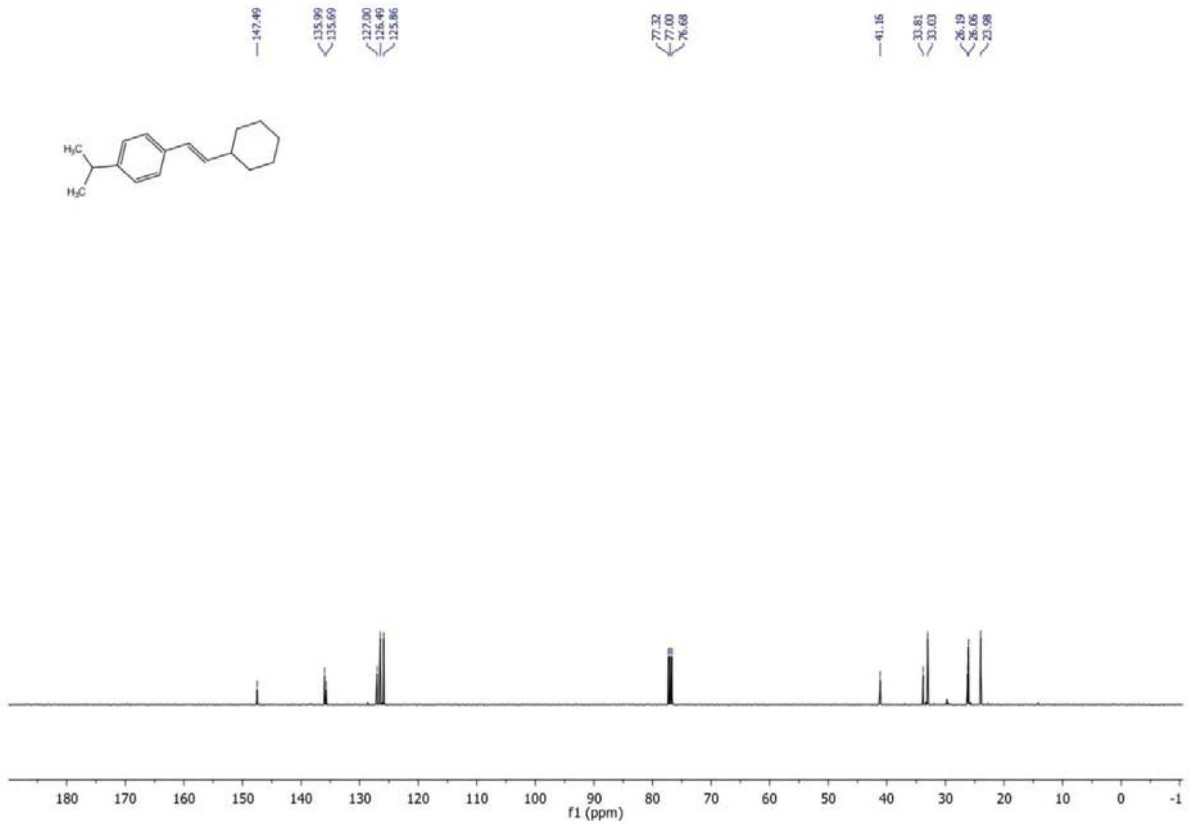

***(E)*-4-(2-cyclohexylvinyl)-1,2-dimethylbenzene (4k)**

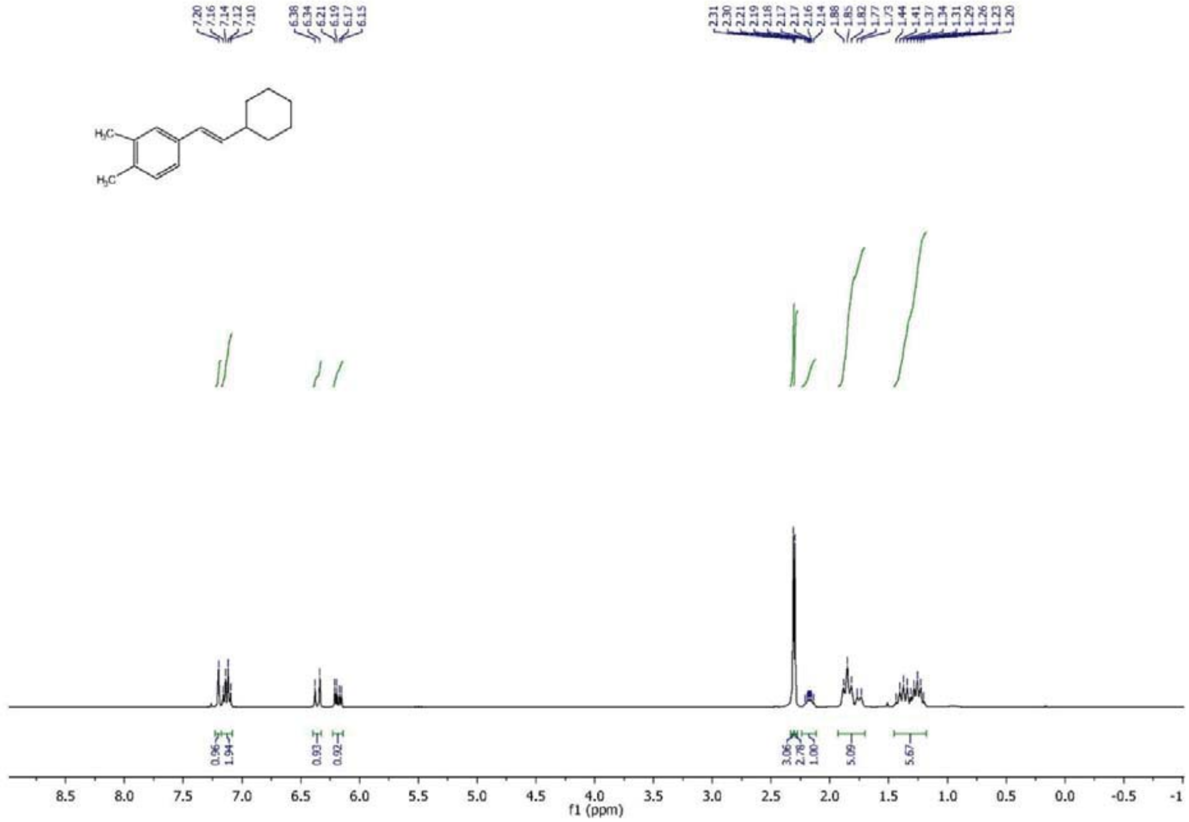

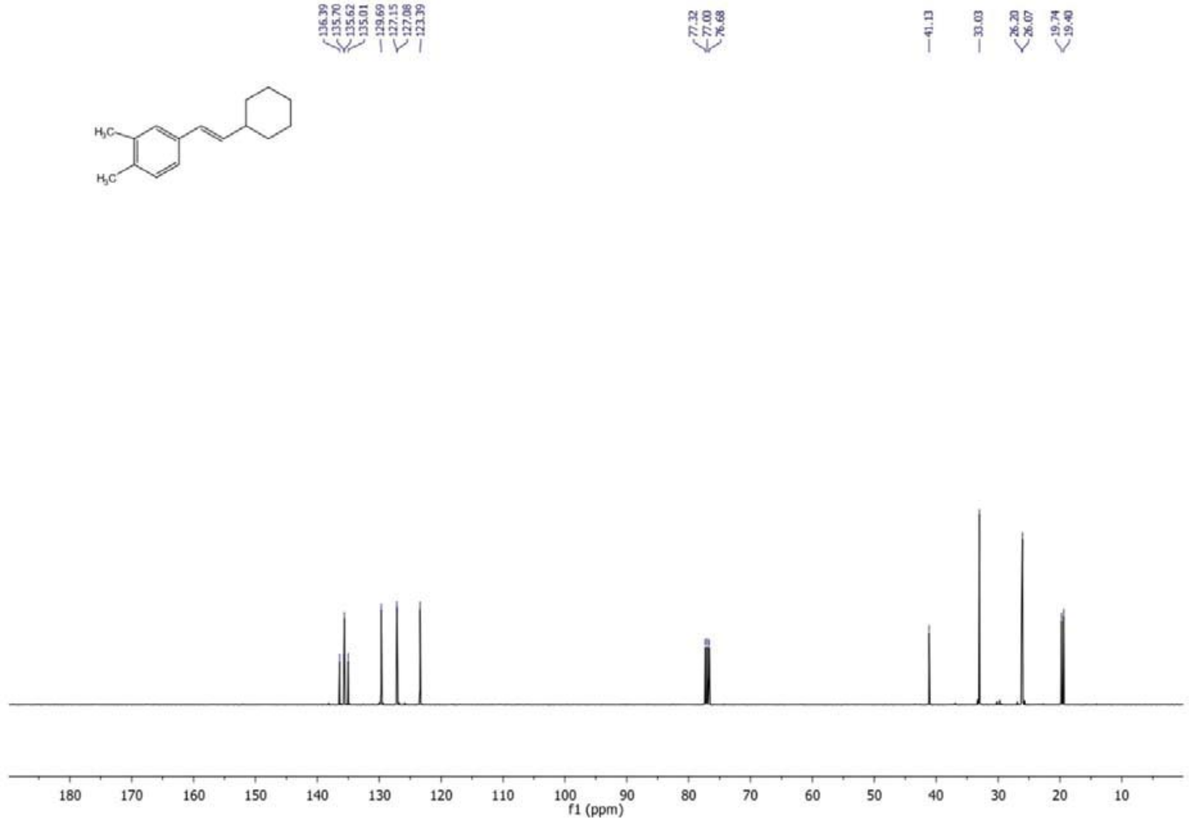

***(E)*-1-(2-cyclohexylvinyl)-2,3,4,5,6-pentamethylbenzene (4l)**

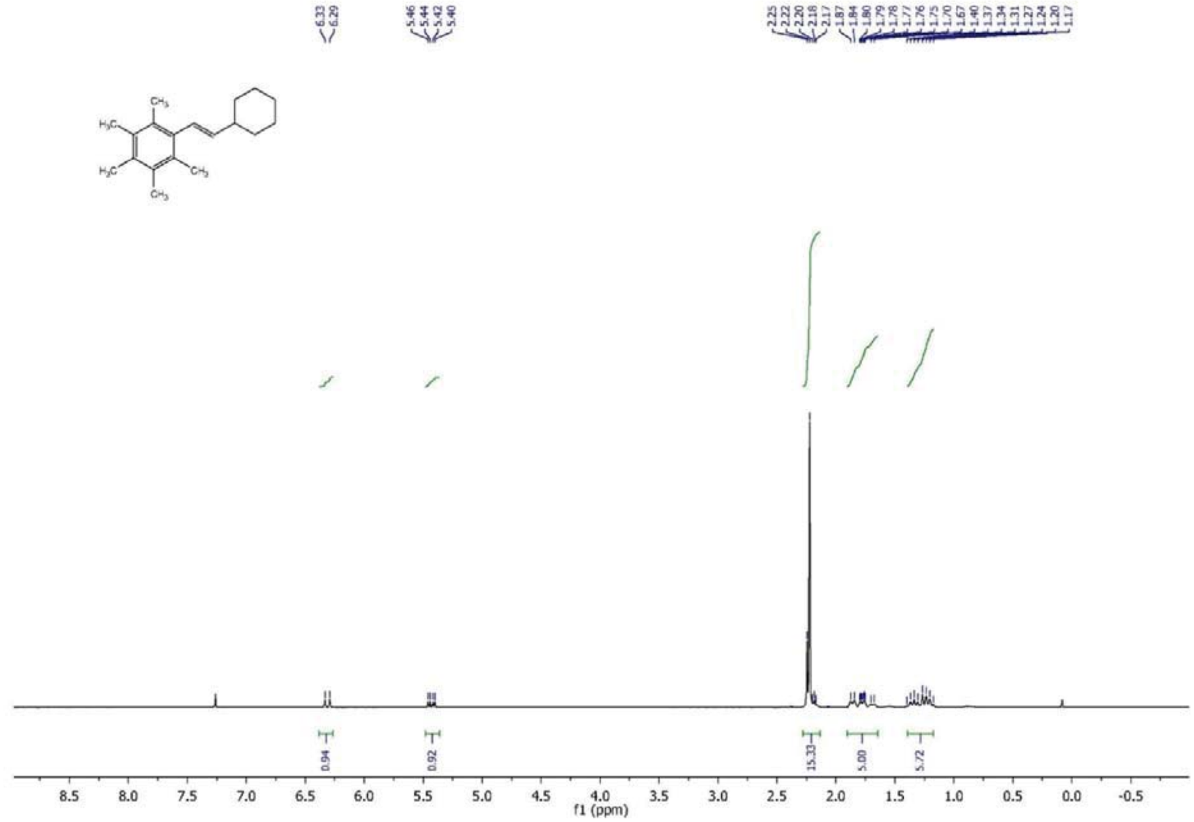

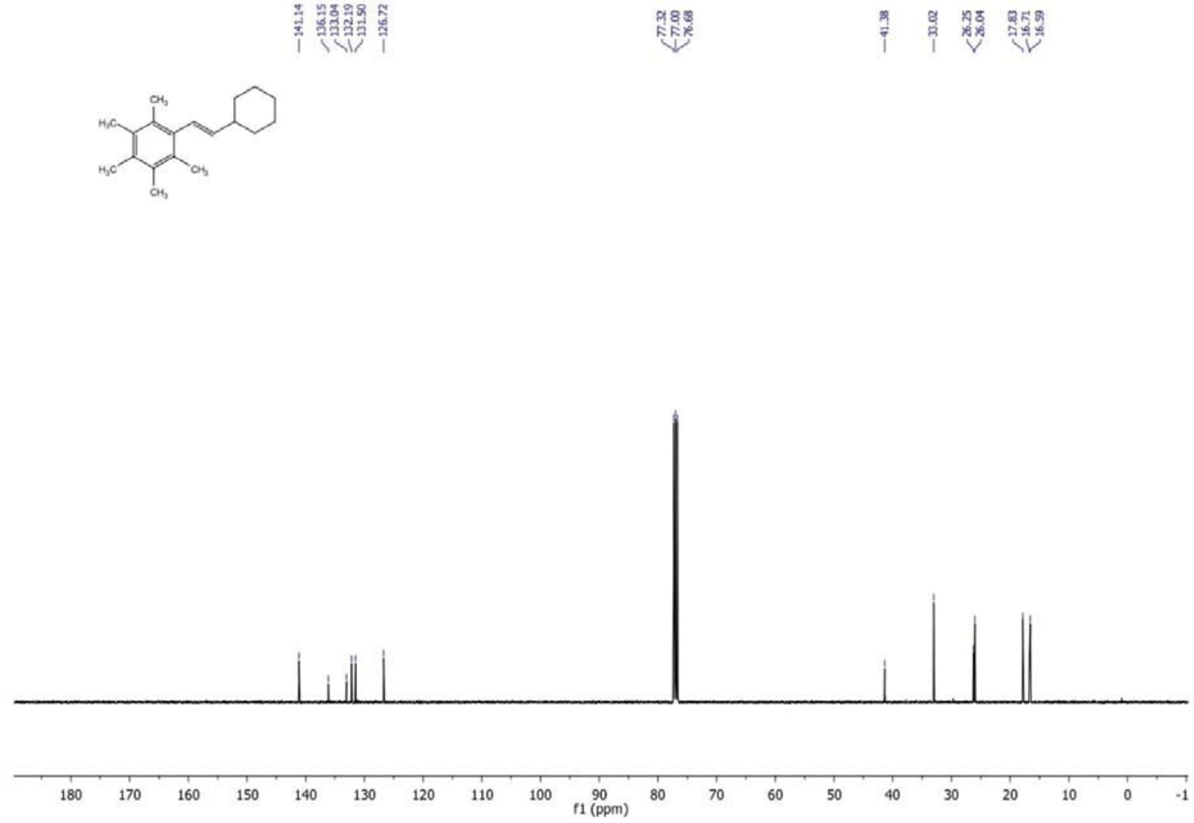

***(E)*-1-(benzyloxy)-2-(2-cyclohexylvinyl)benzene (4m)**

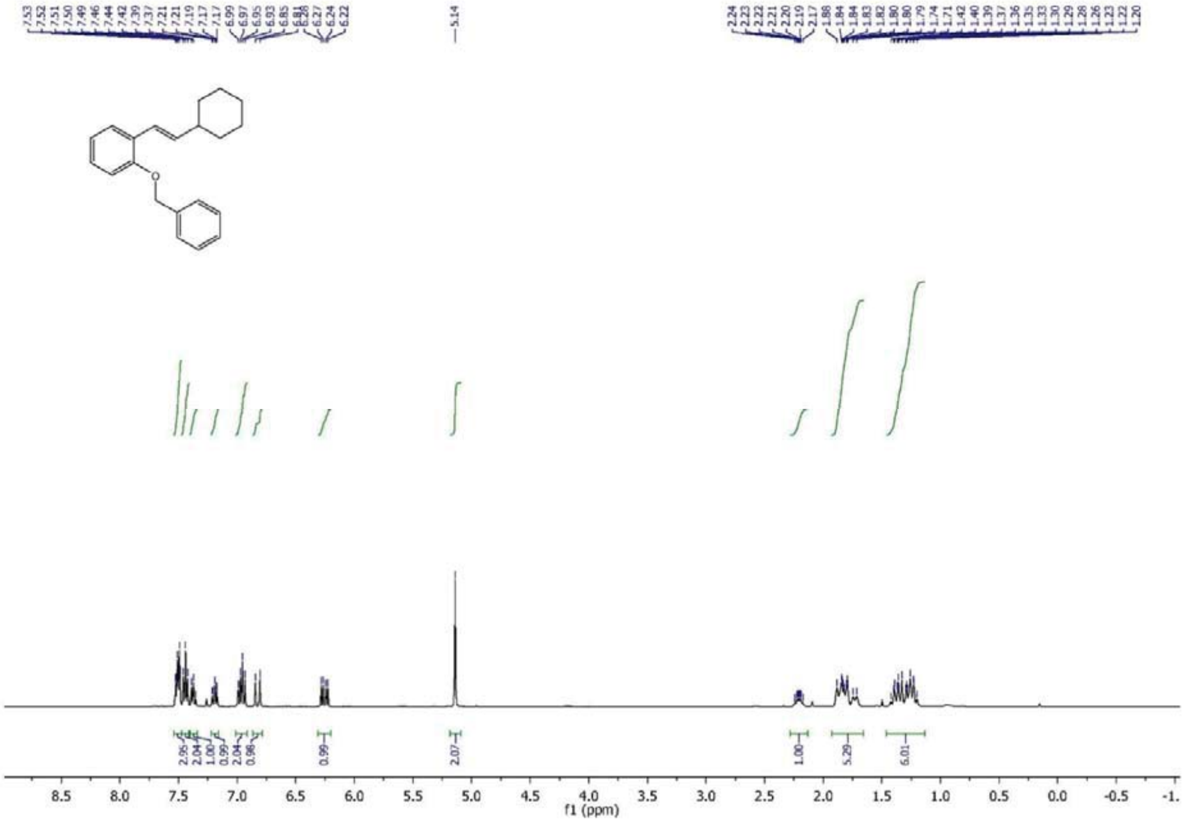

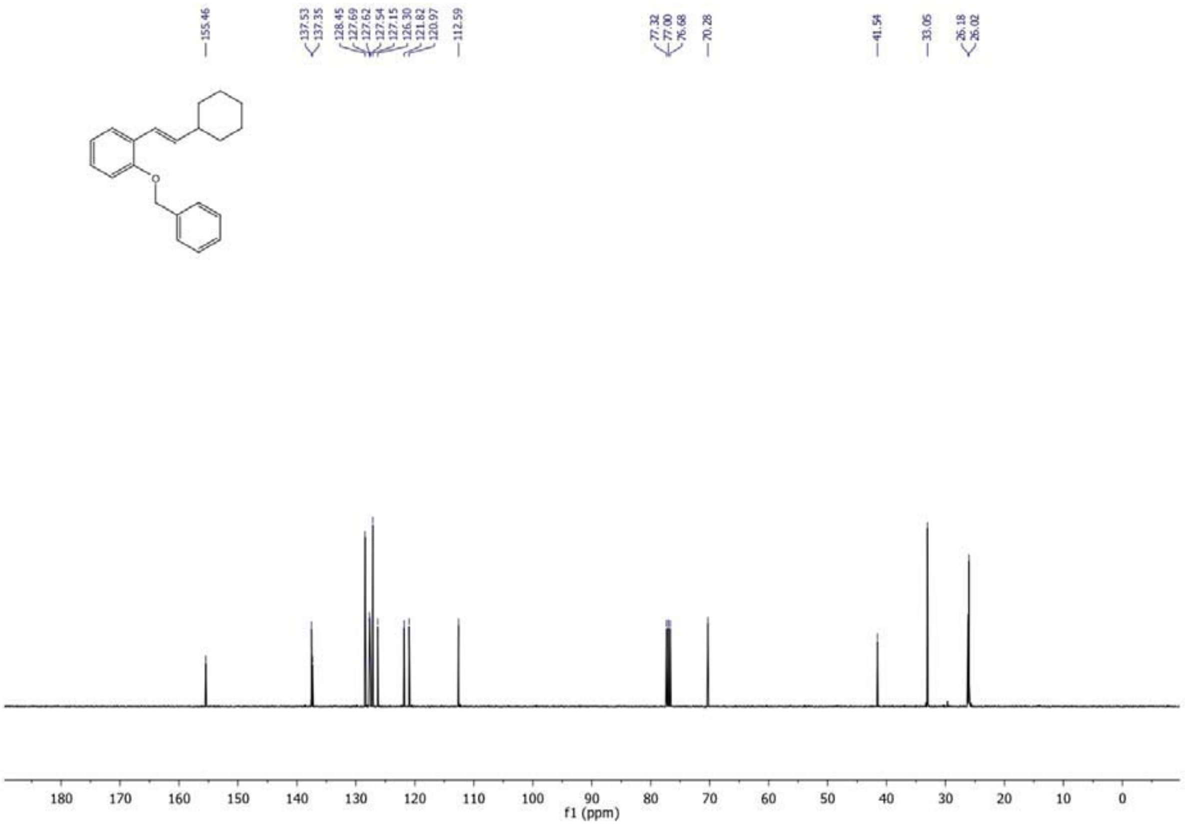

***(E)*-1-(2-cyclohexylvinyl)-4-methoxybenzene (4n)**

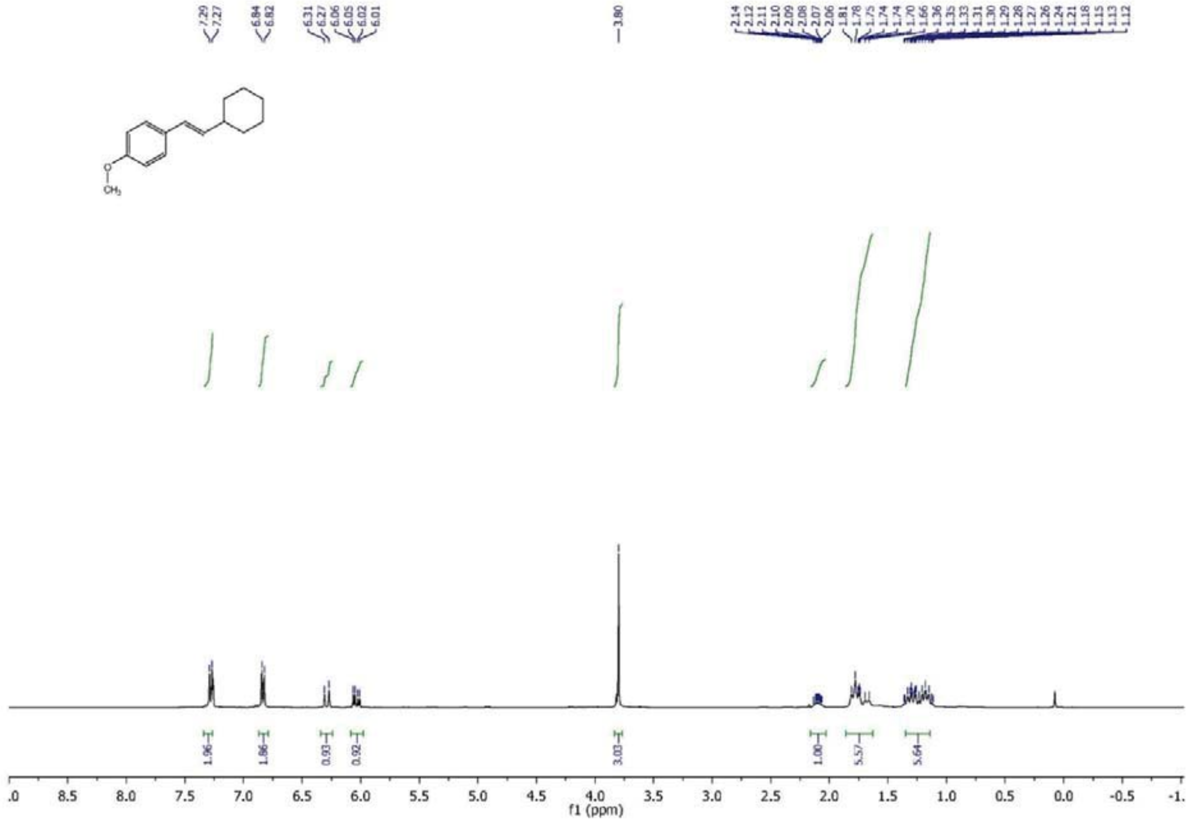

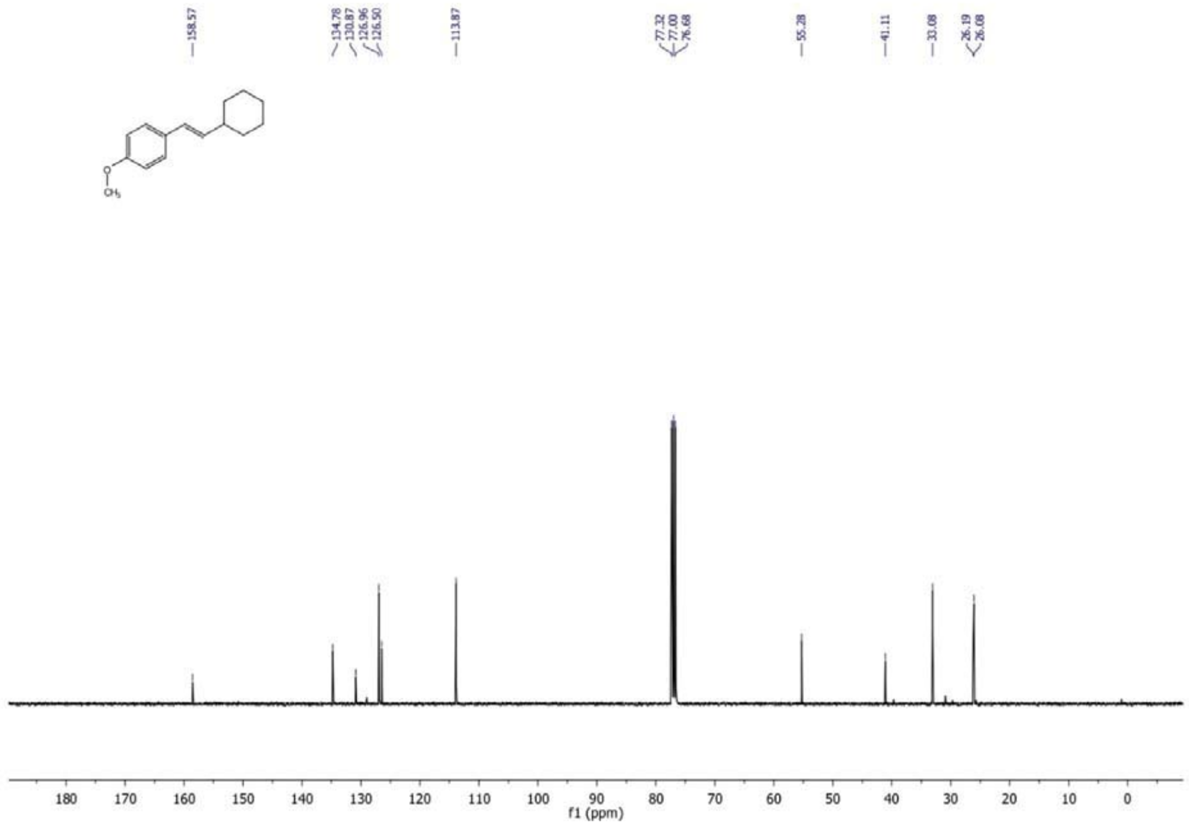

(E)-4-(2-cyclohexylvinyl)-1,2-dimethoxybenzene (4o)

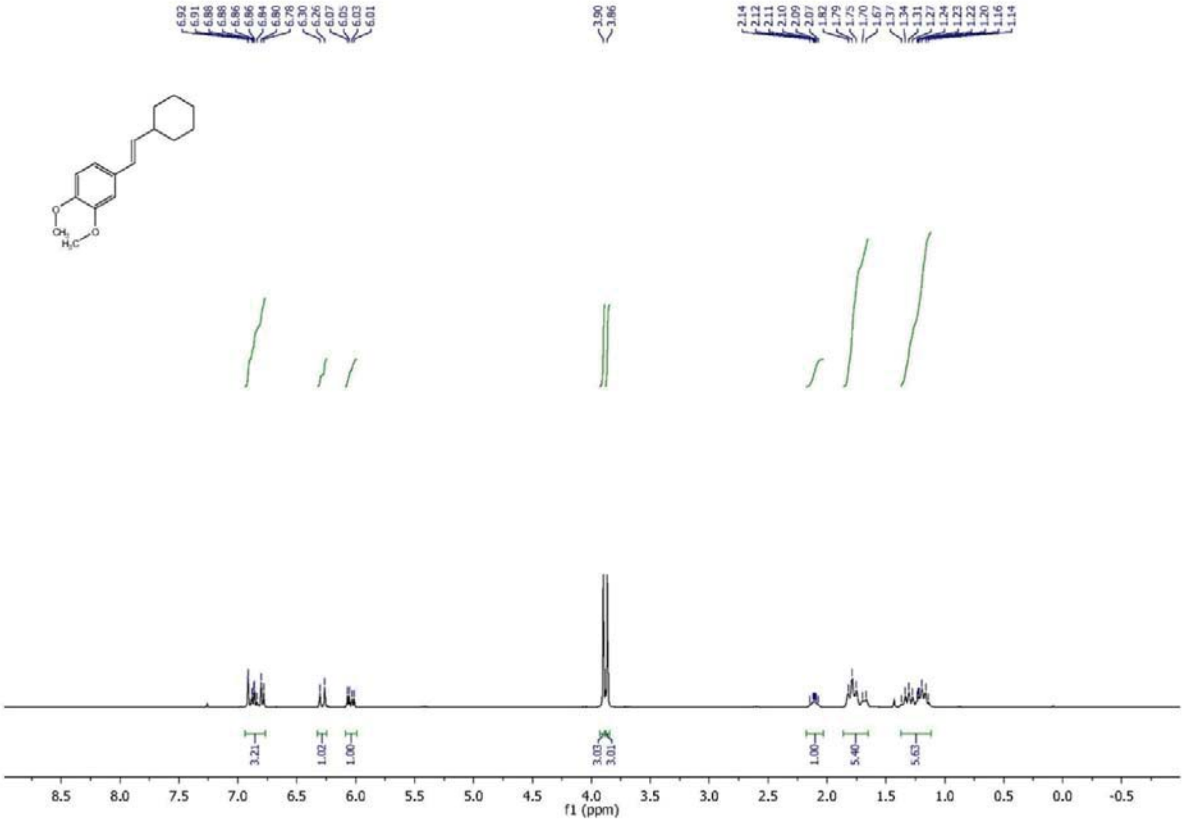

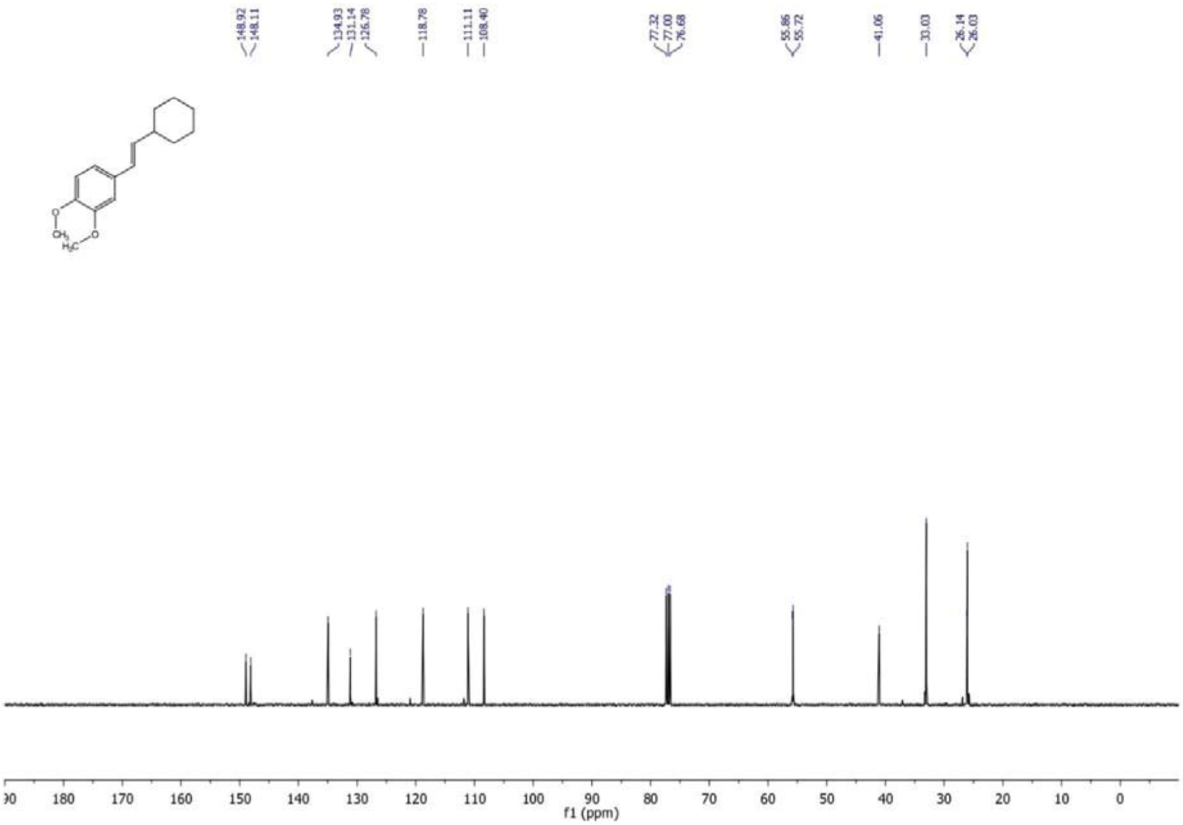

***(E)*-5-(2-cyclohexylvinyl)benzo[*d*][1,3]dioxole (4p)**

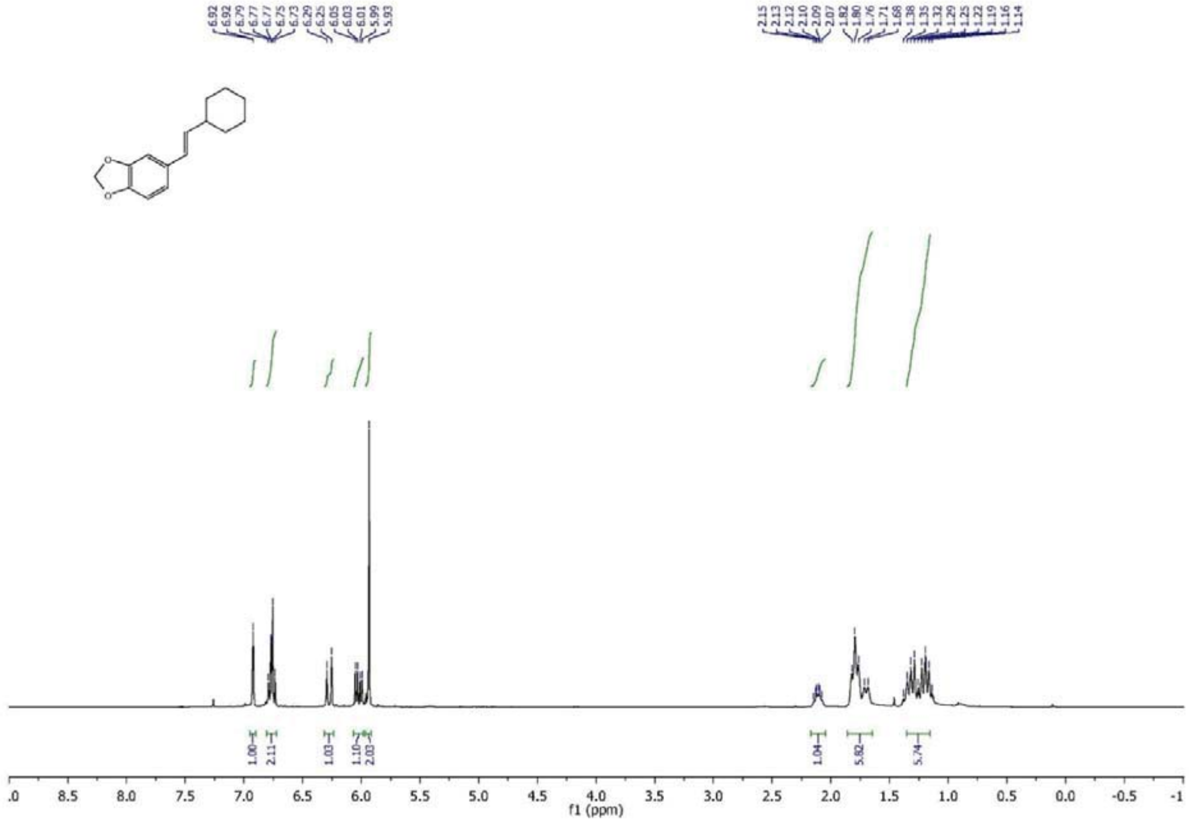

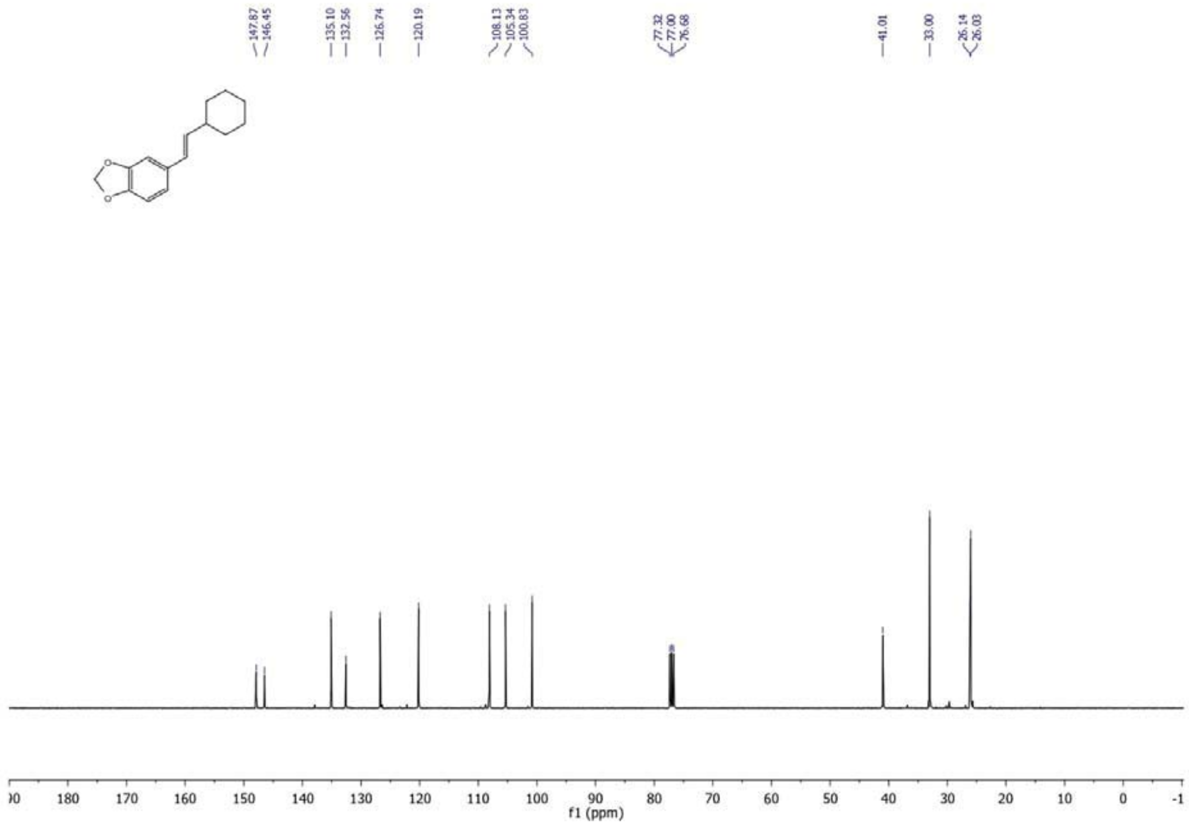

***(E)*-2-(2-cyclohexylvinyl)-1,4-dimethoxybenzene (4q)**

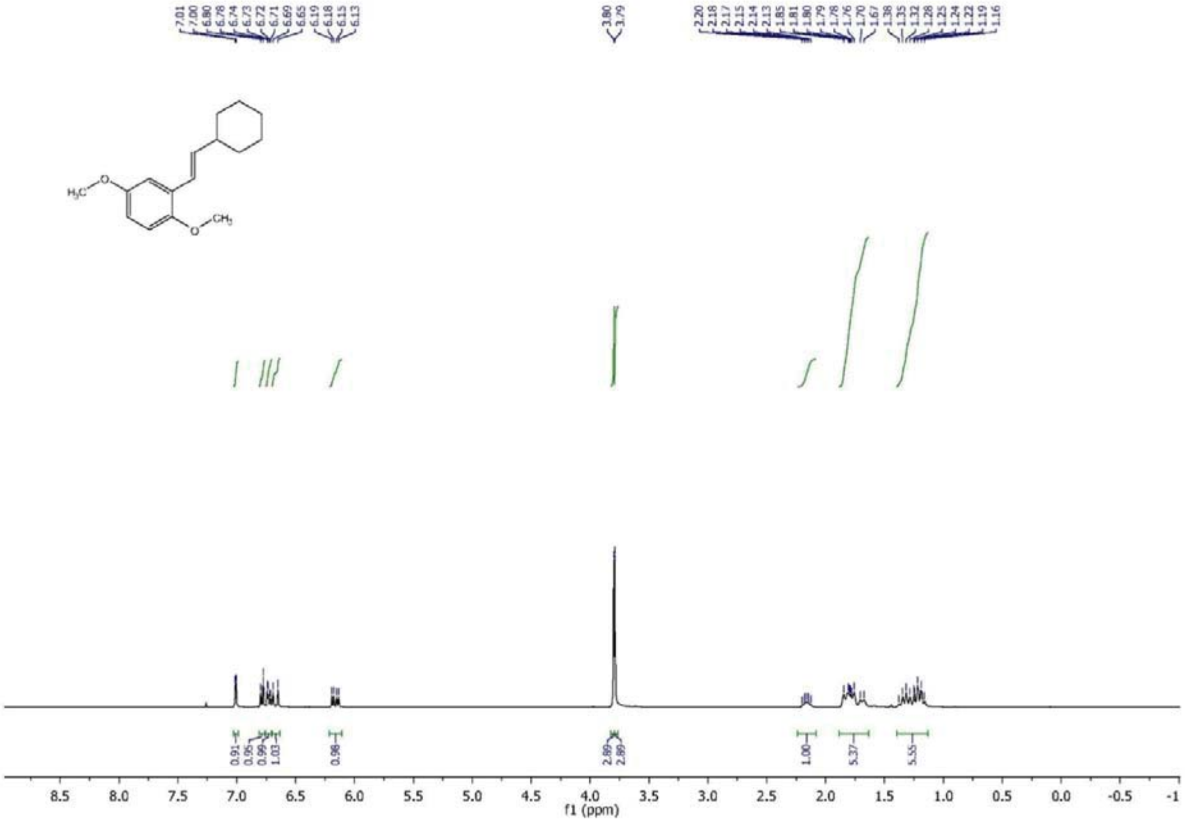

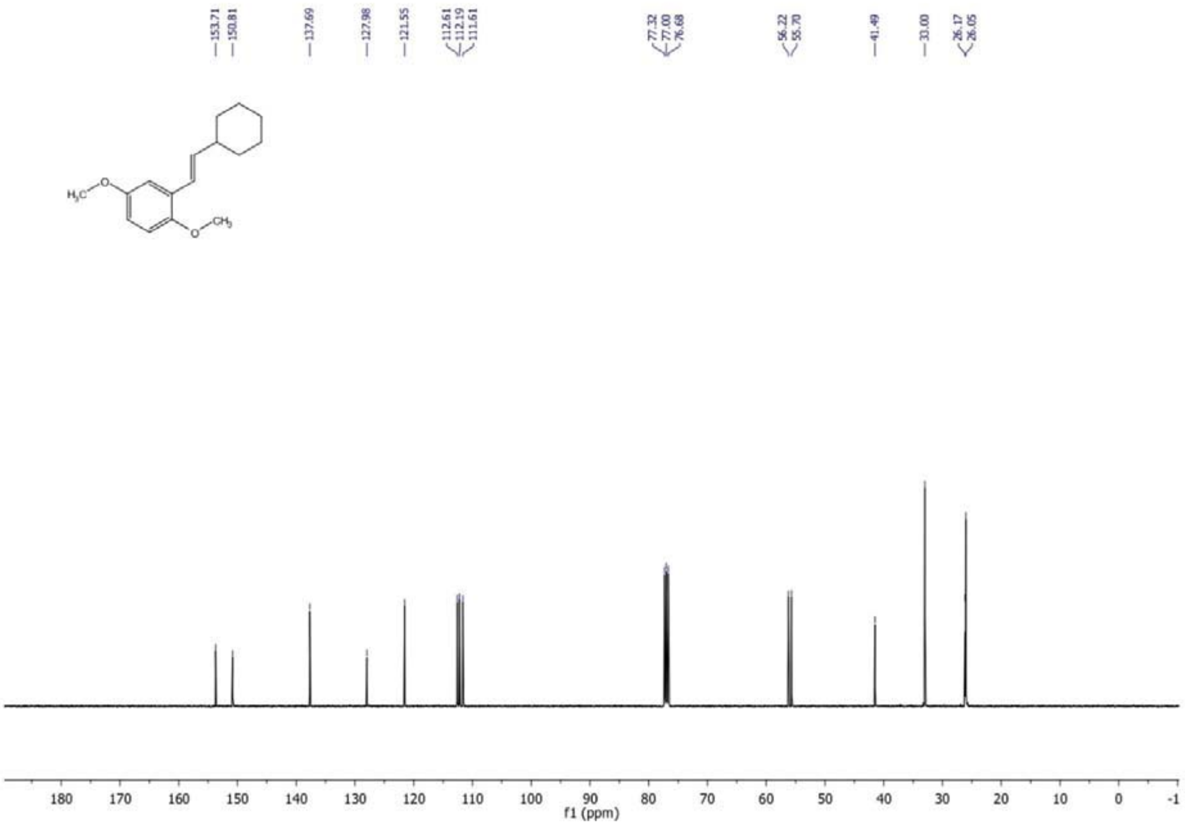

***(E)*-1-(2-cyclohexylvinyl)-2-fluorobenzene (4r)**

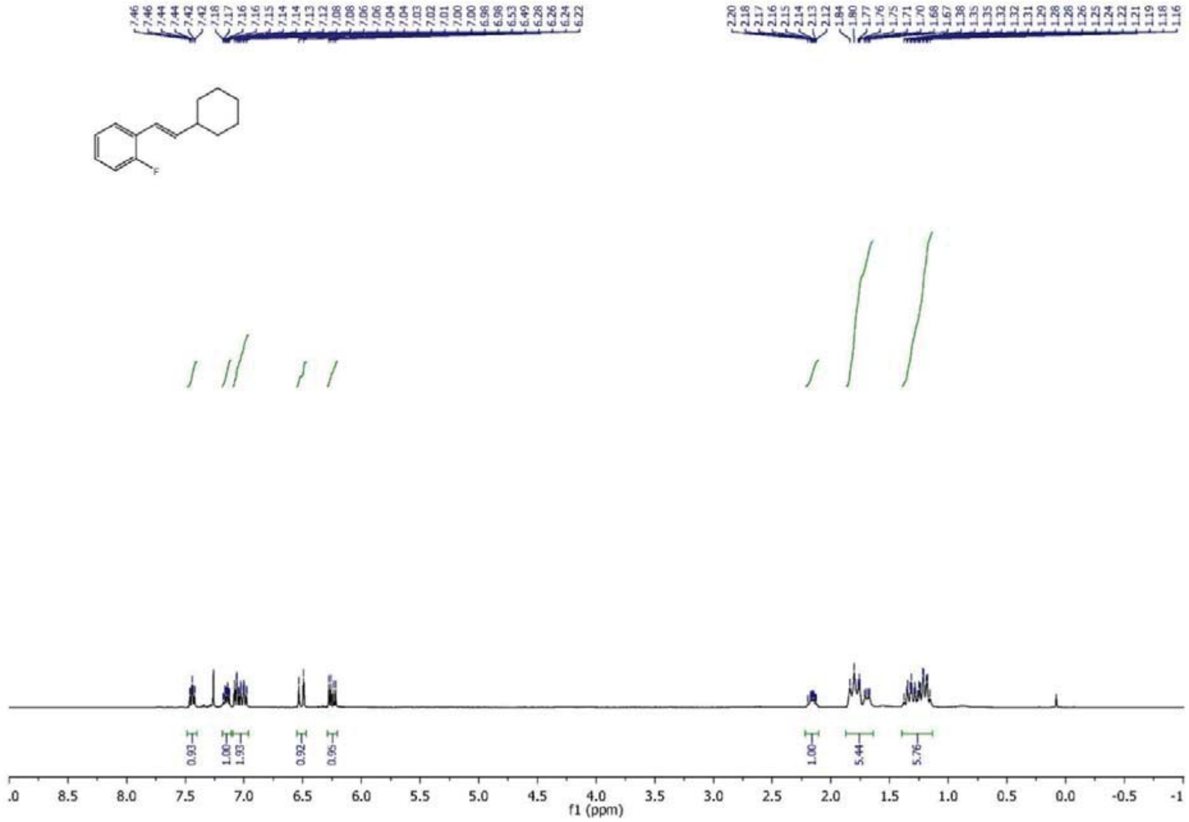

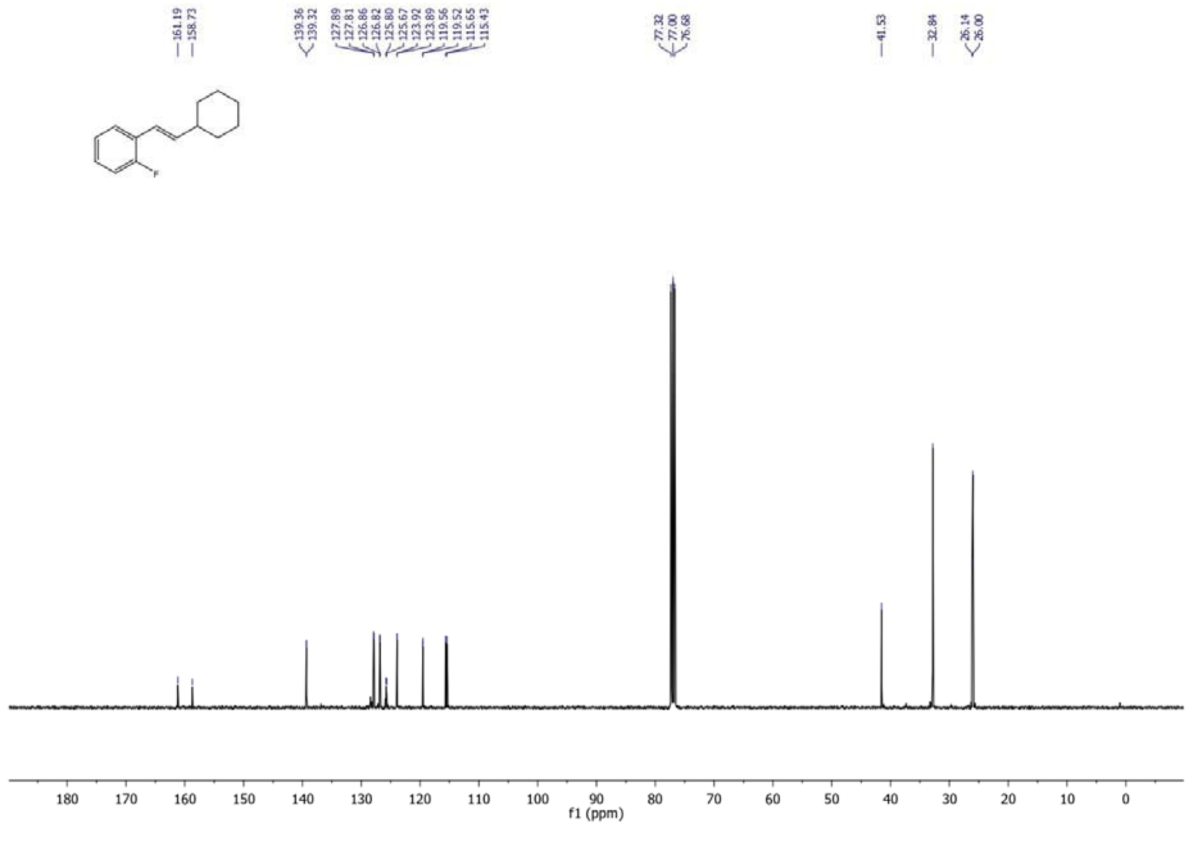

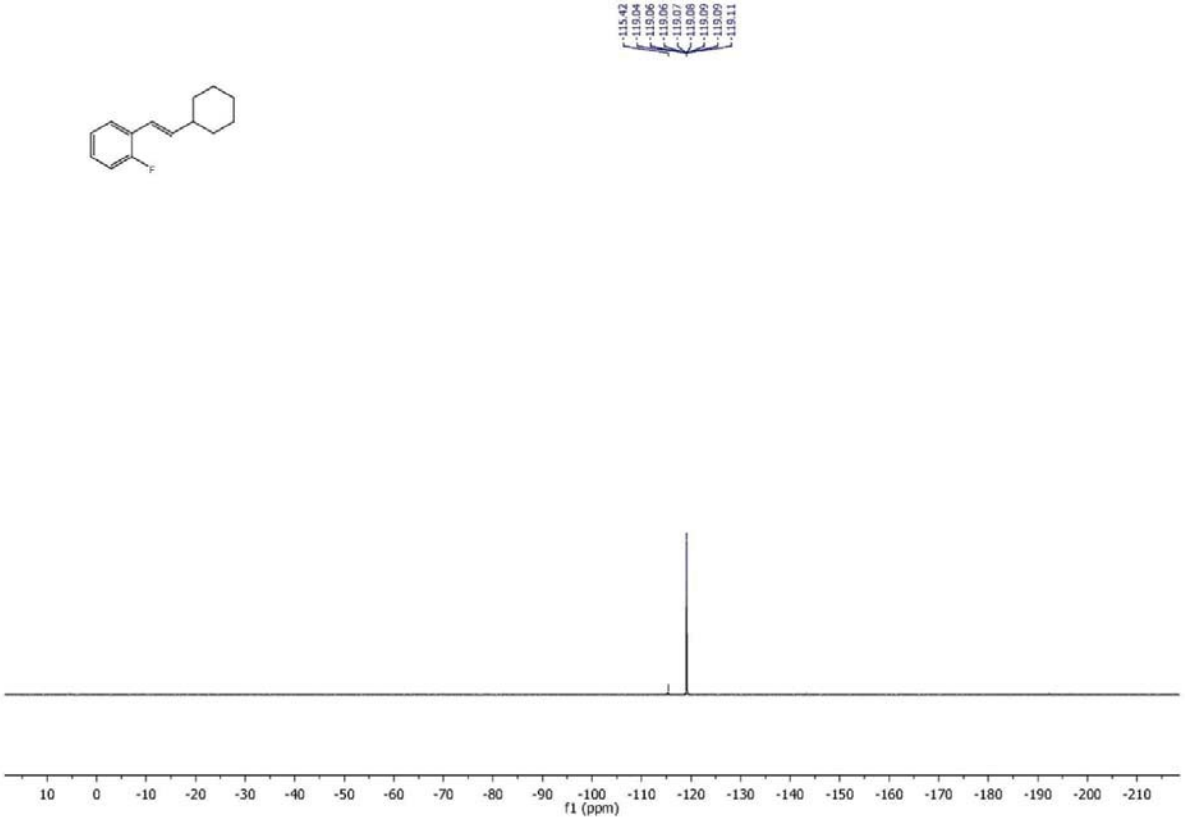

***(E)*-1-(2-cyclohexylvinyl)-2-methylnaphthalene (4s)**

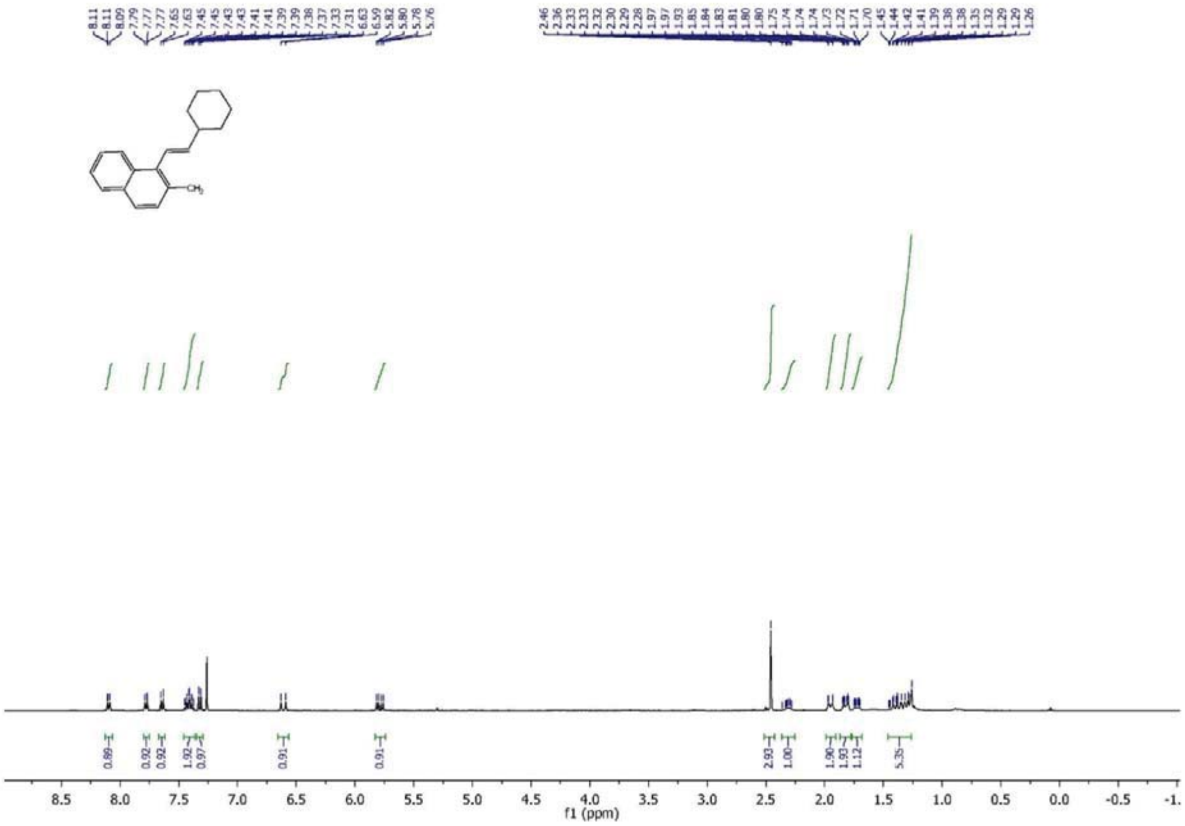

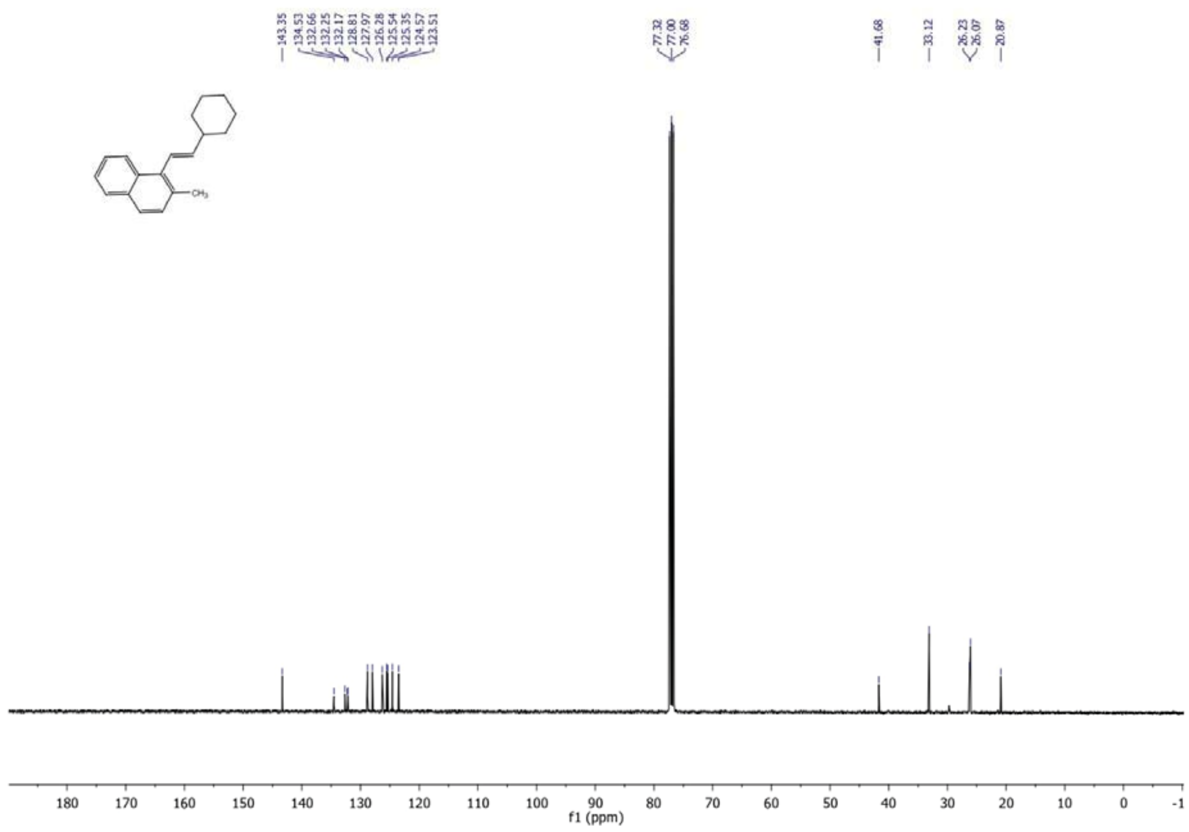

***(E)*-1-(2-cyclohexylvinyl)naphthalene (4t)**

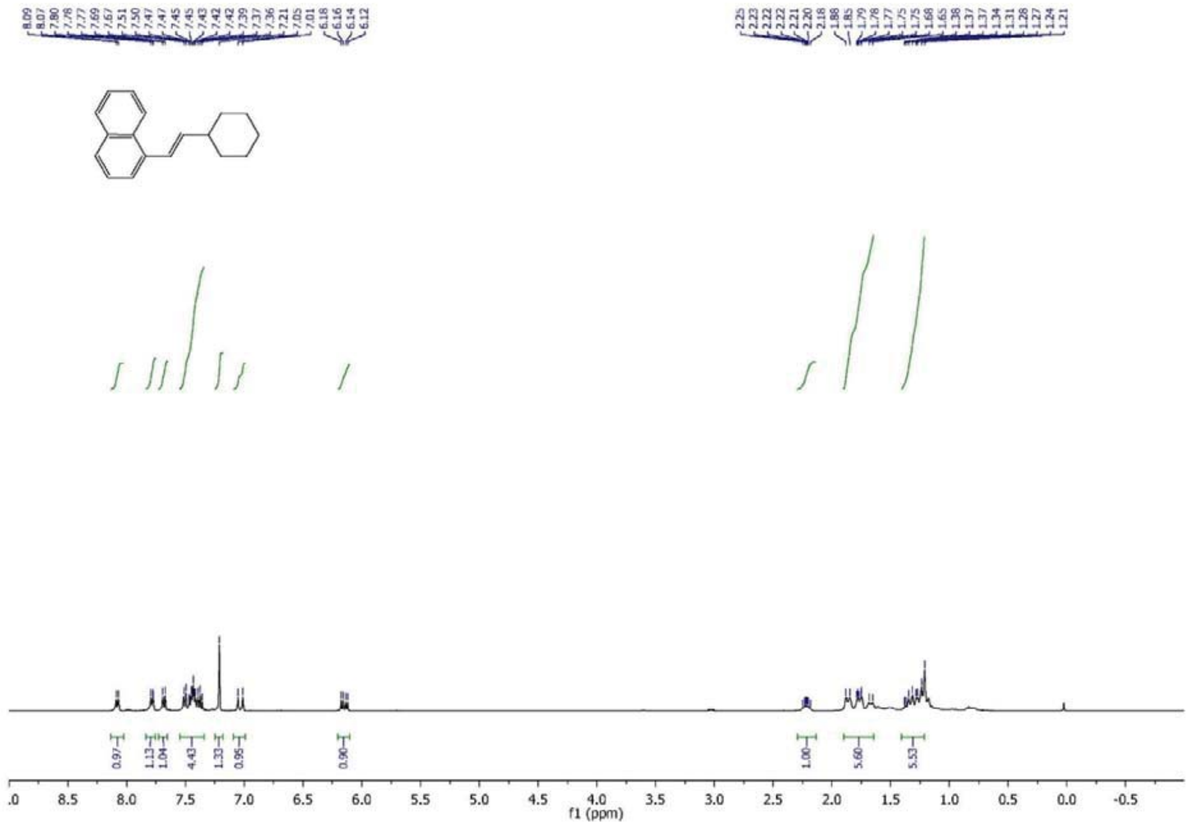

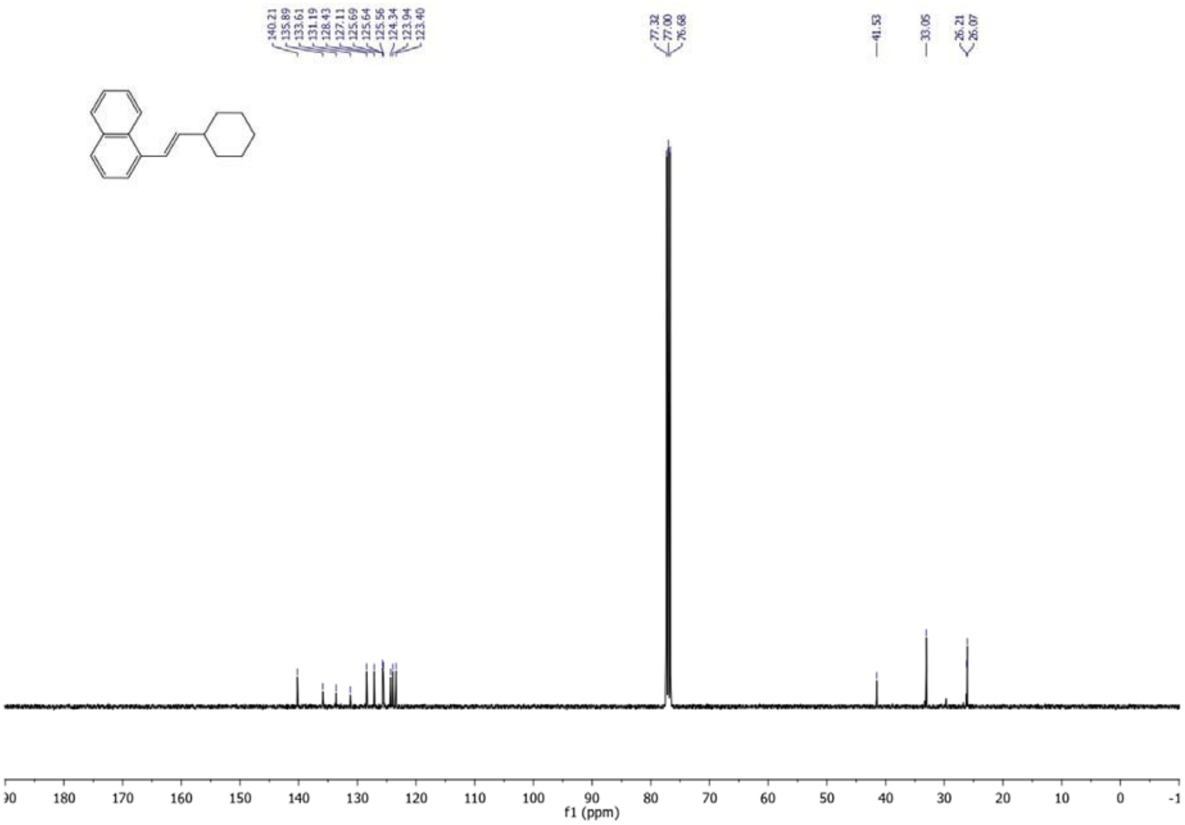

(*E*)-(1-cyclohexylprop-1-en-2-yl)benzene (4u)

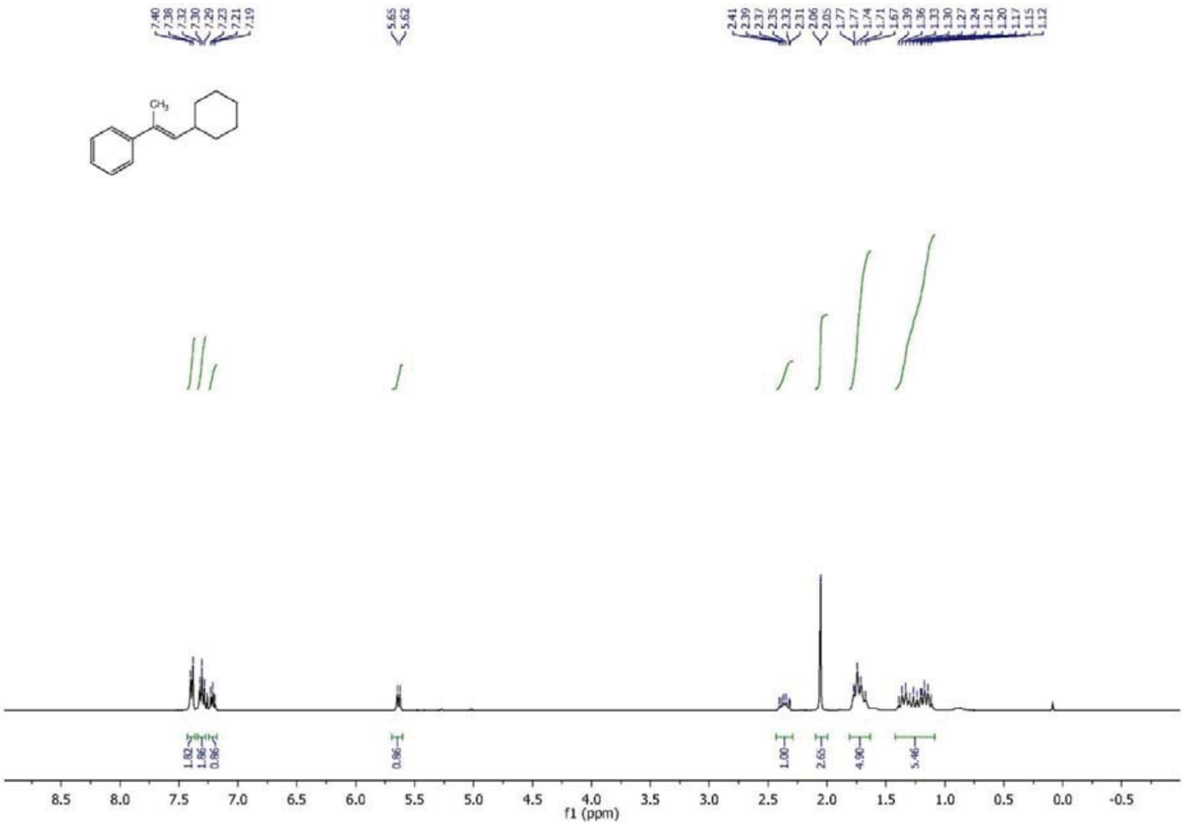

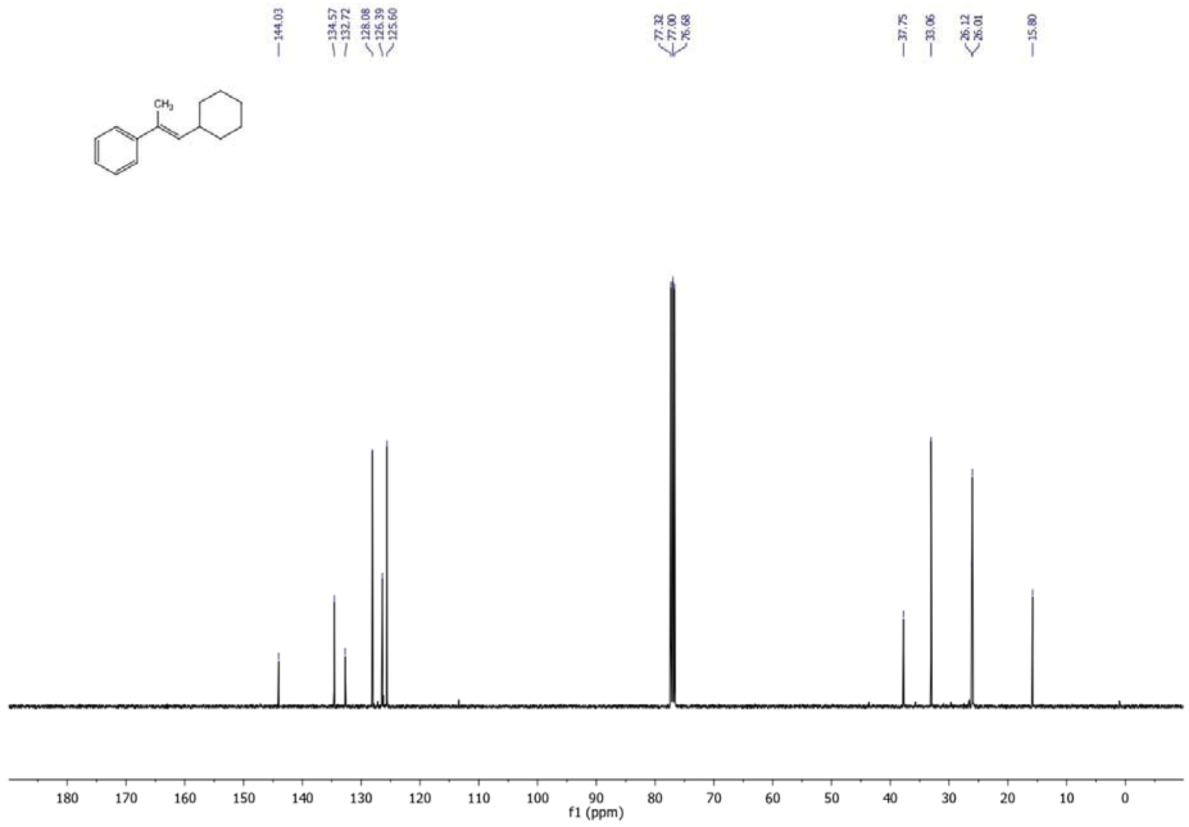

(2-cyclohexylethene-1,1-diyl)dibenzene (4v)

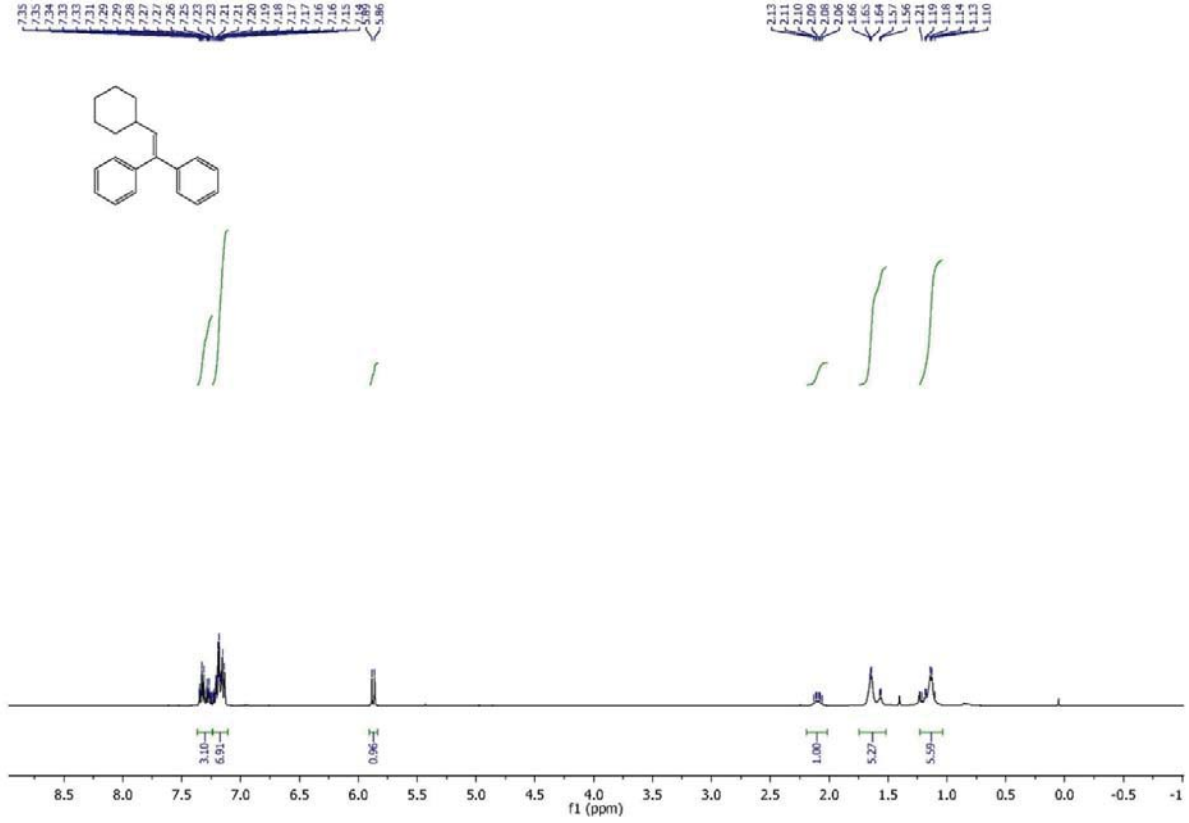

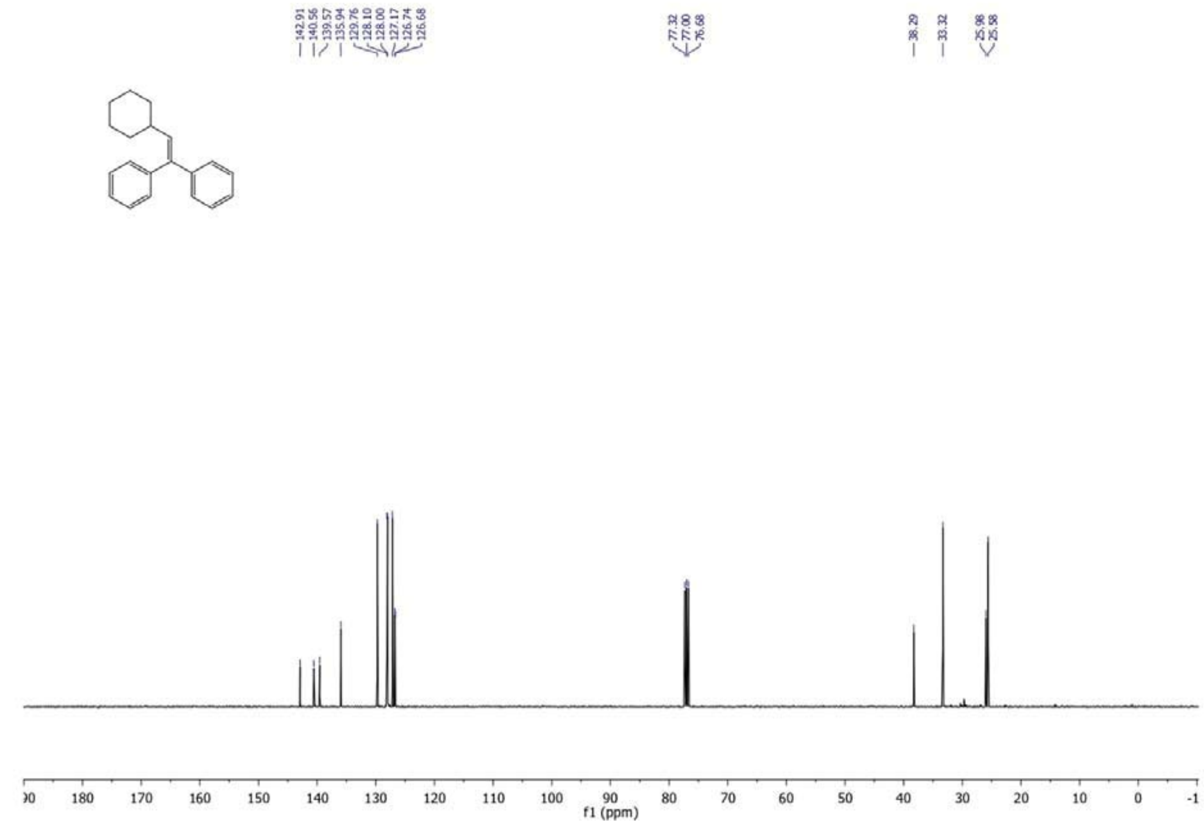

1,4-bis(*(E)*-2-cyclohexylvinyl)benzene (4w)

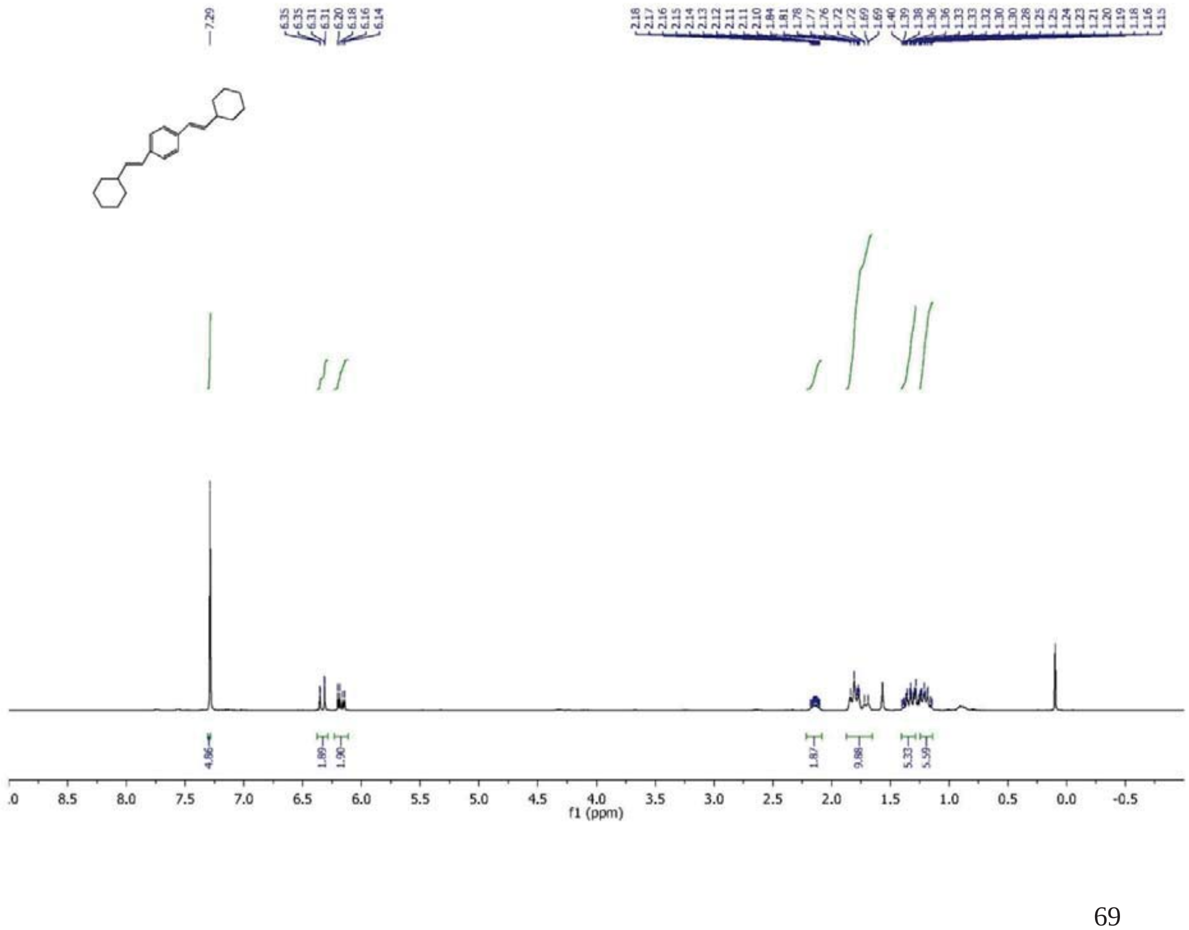

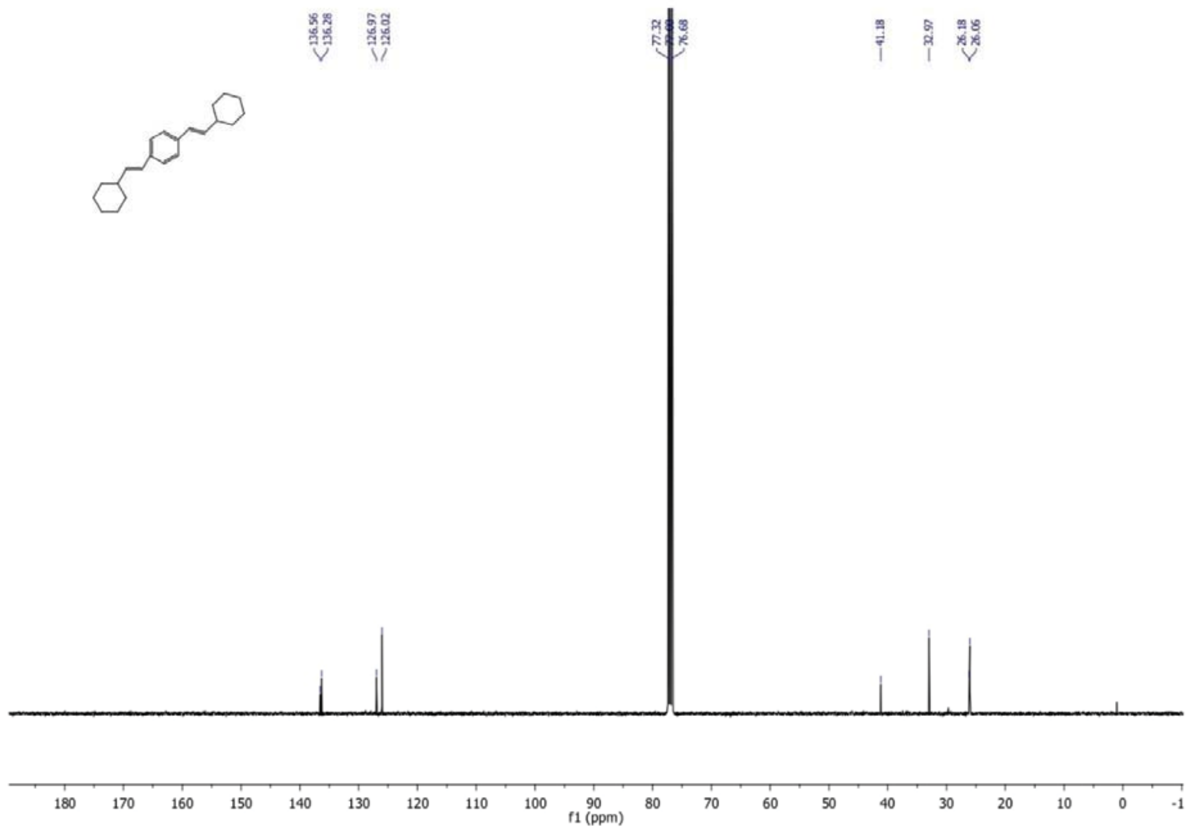

***(Z)*-(2-cyclohexylvinyl)benzene (4x)**

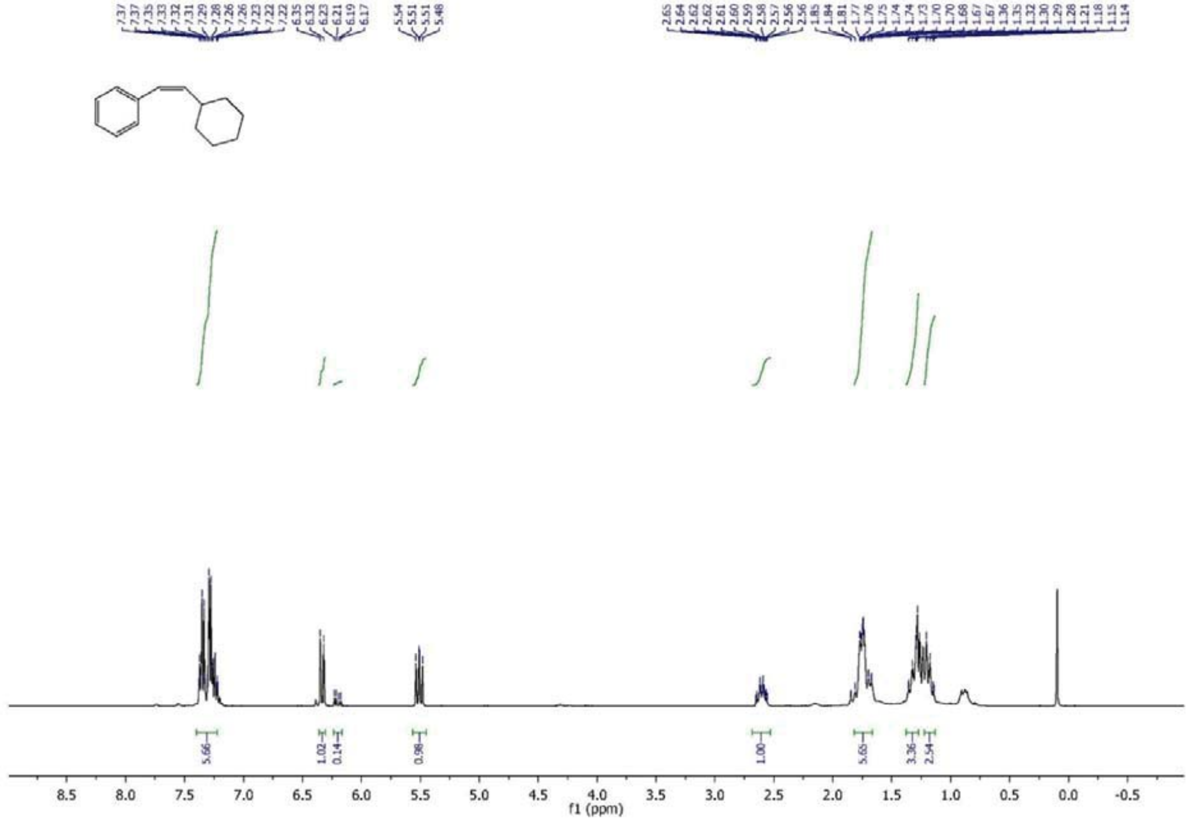

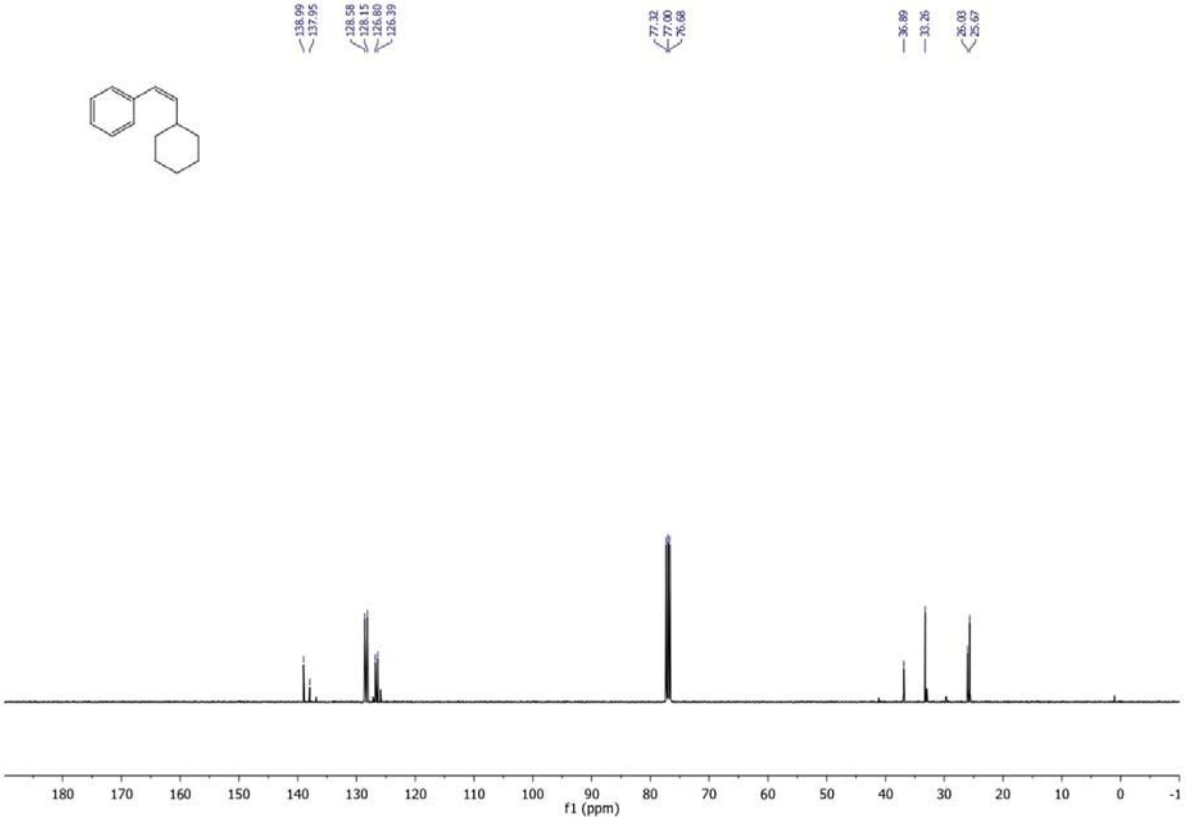

(*E*)-hept-1-en-1-ylbenzene (5a)

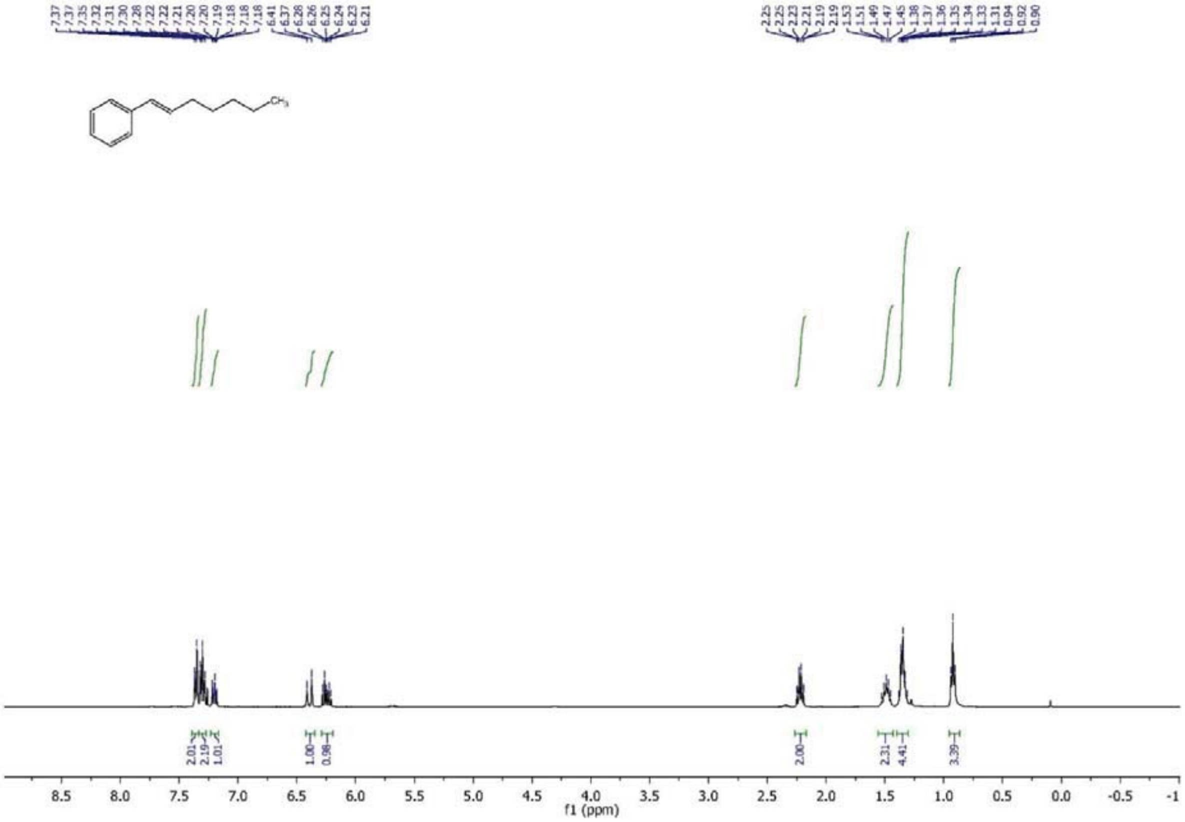

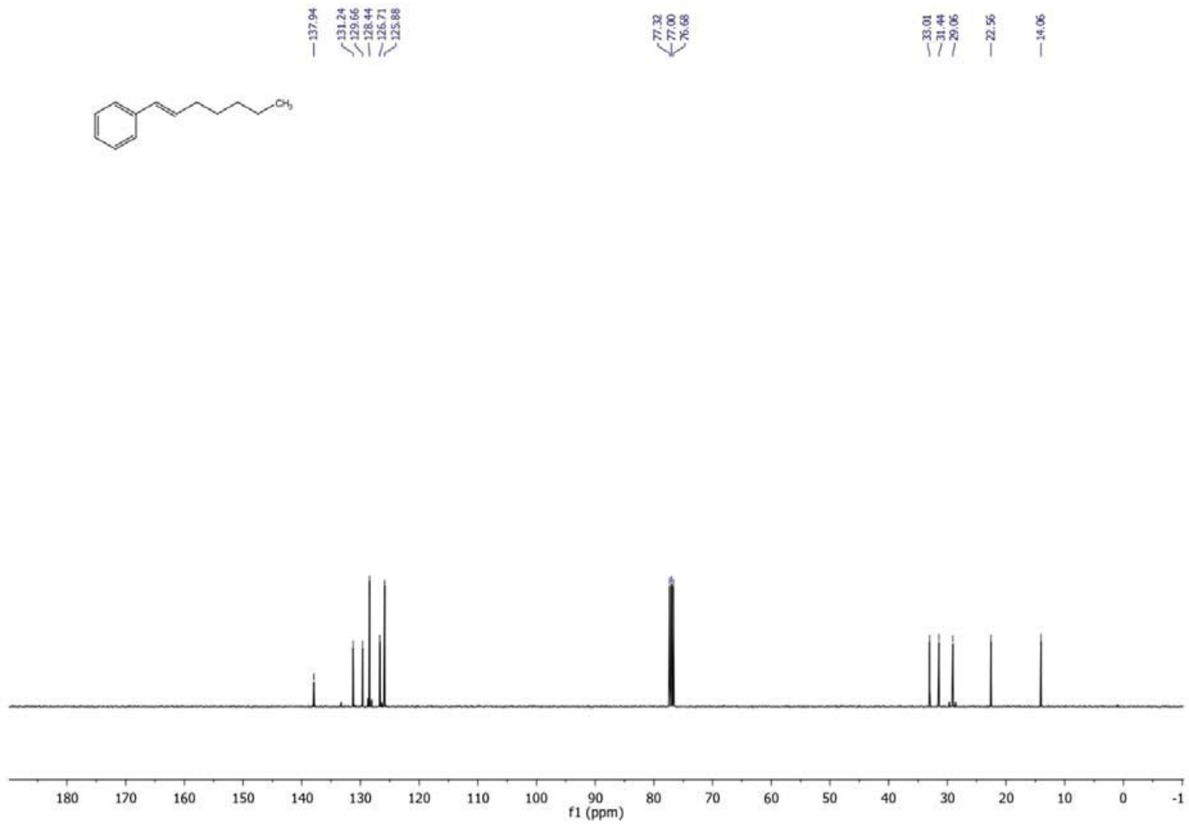

(*E*)-hexa-1,5-dien-1-ylbenzene (5b)

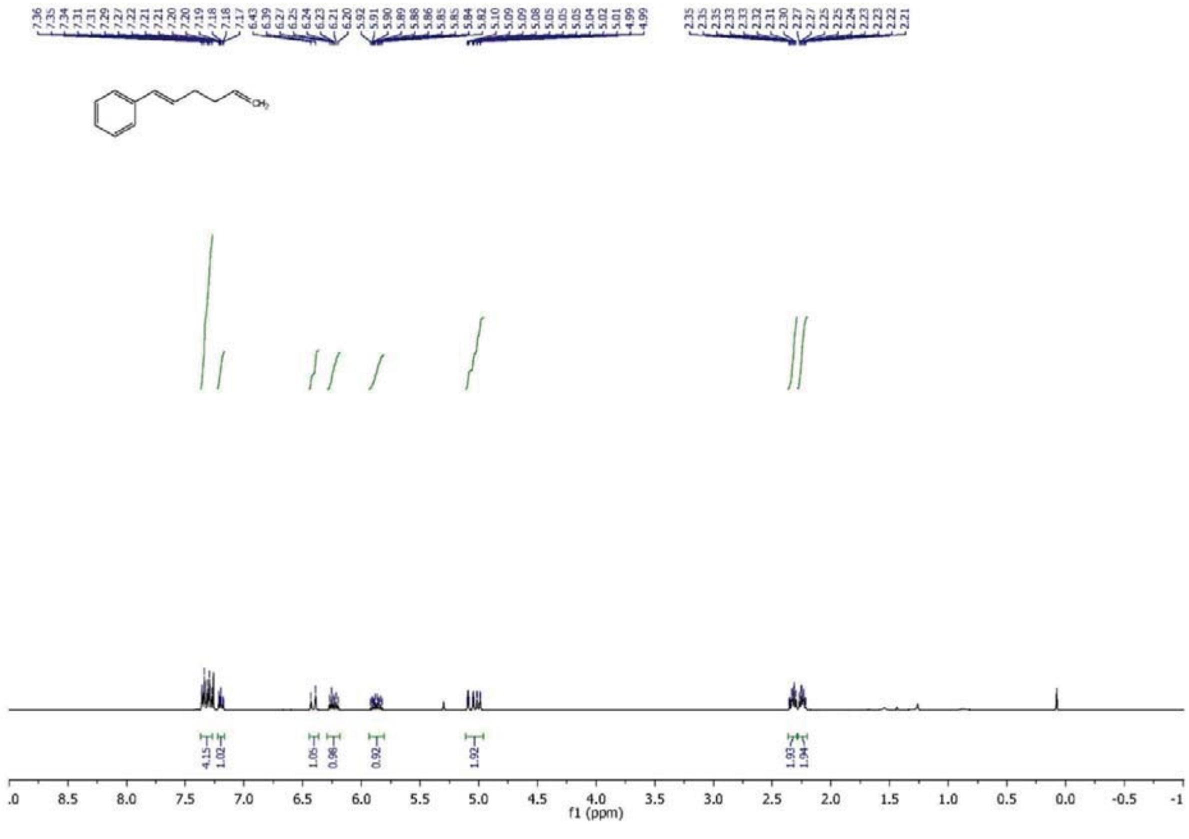

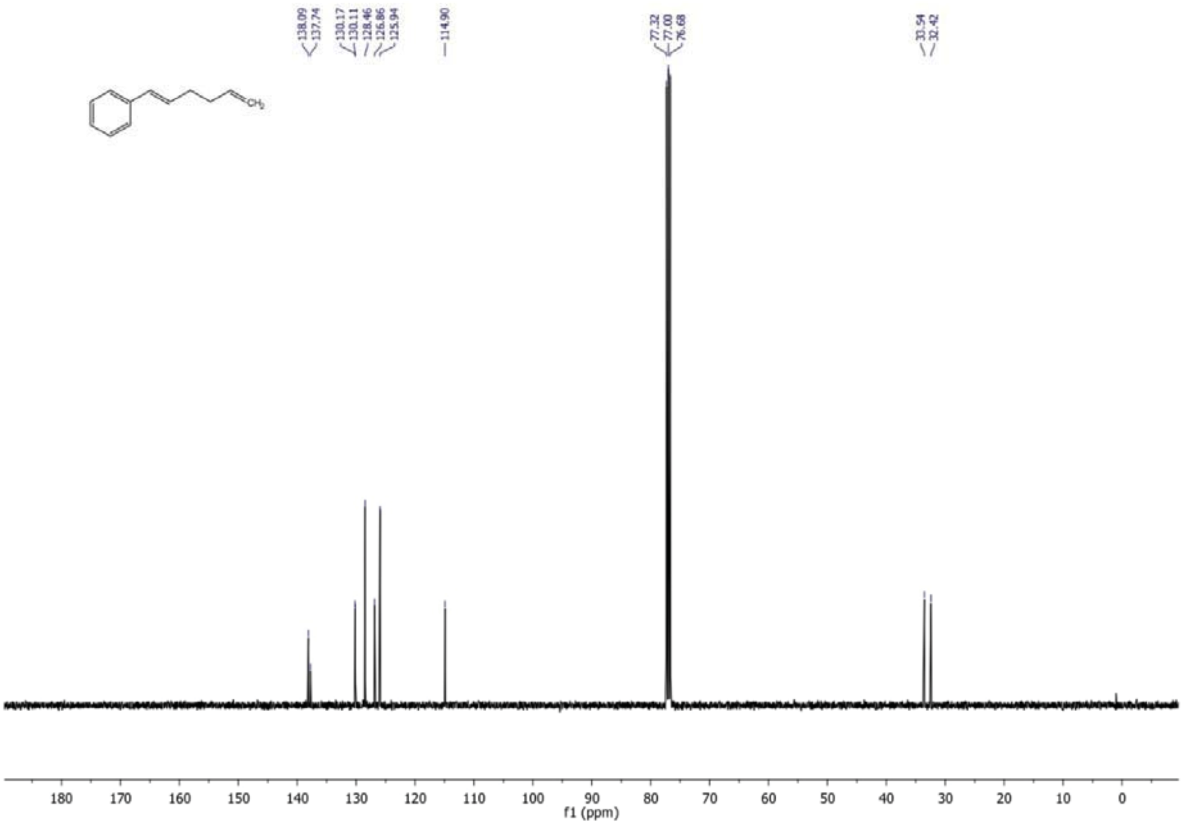

hex-5-en-1-yn-1-ylbenzene (5c)

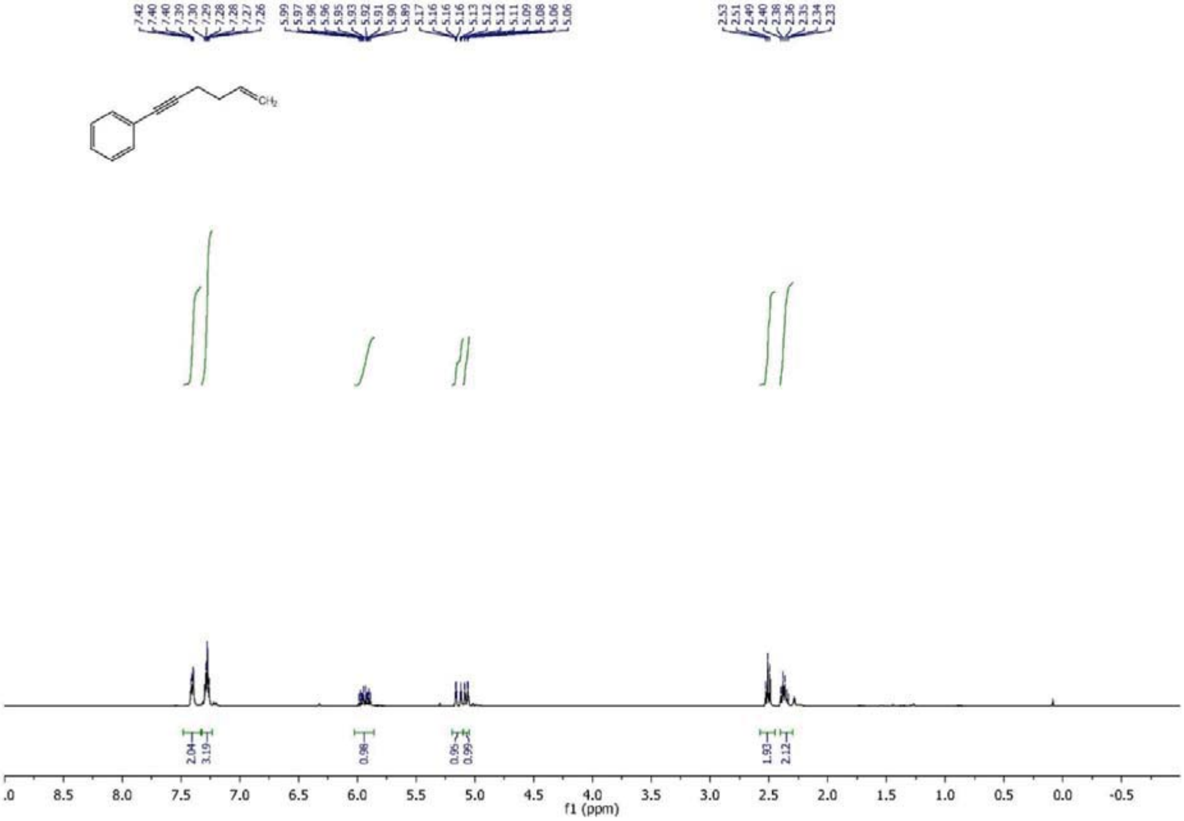

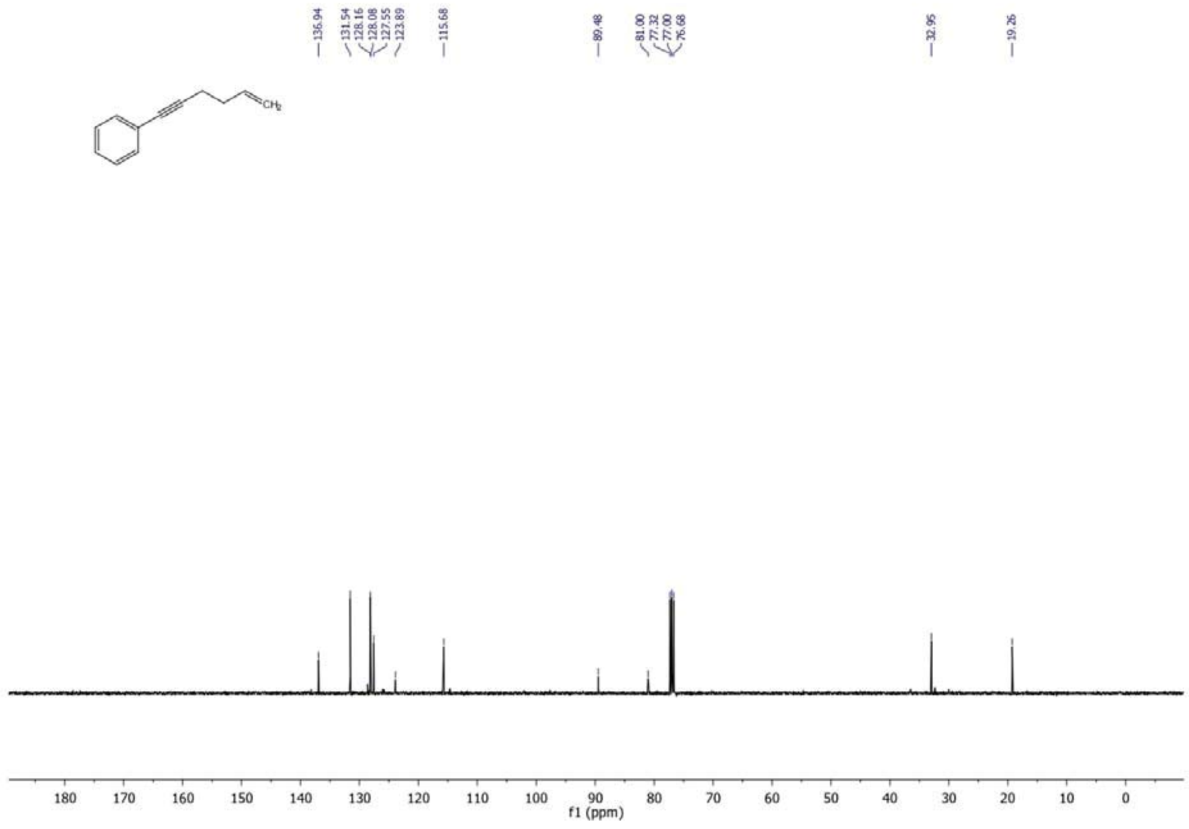

(3-methylbut-1-yn-1-yl)benzene (5f)

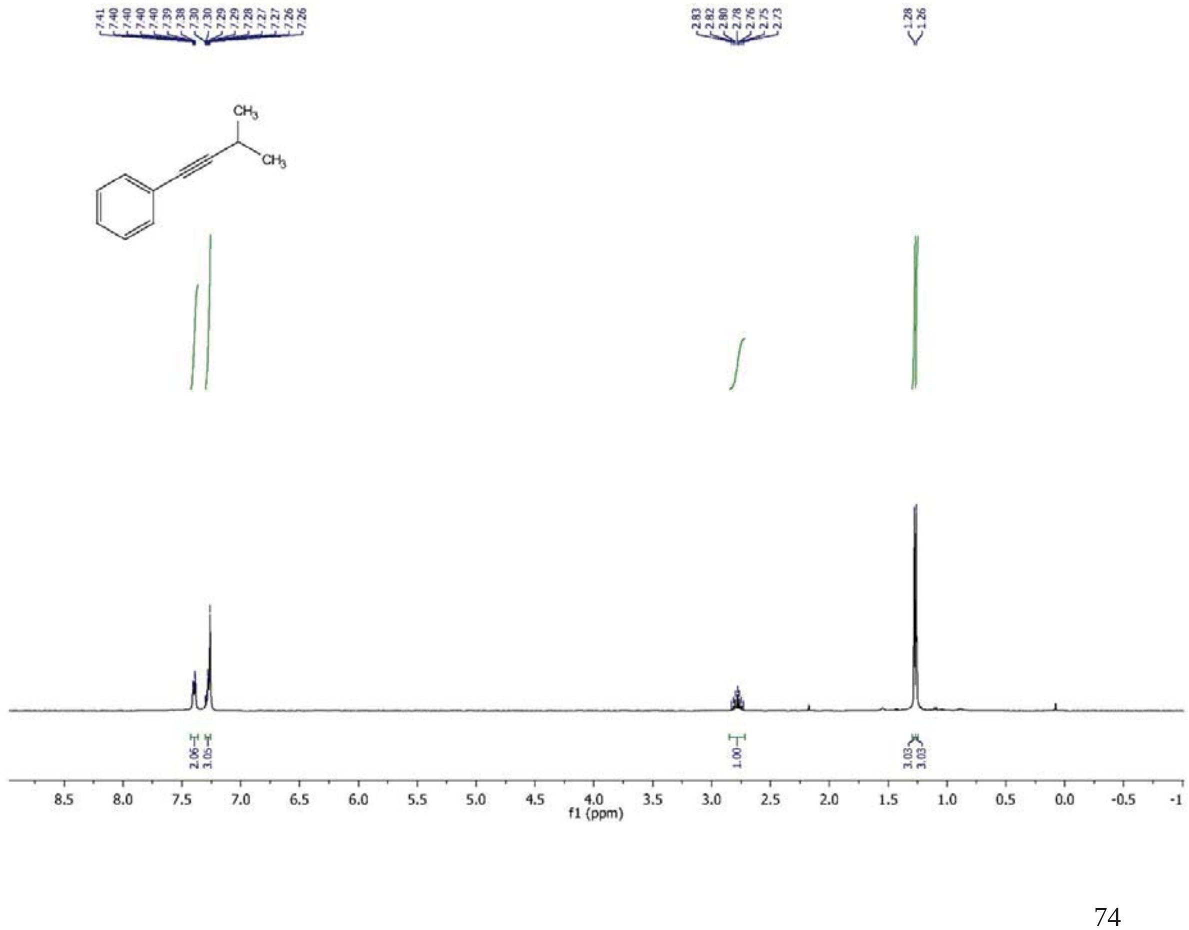

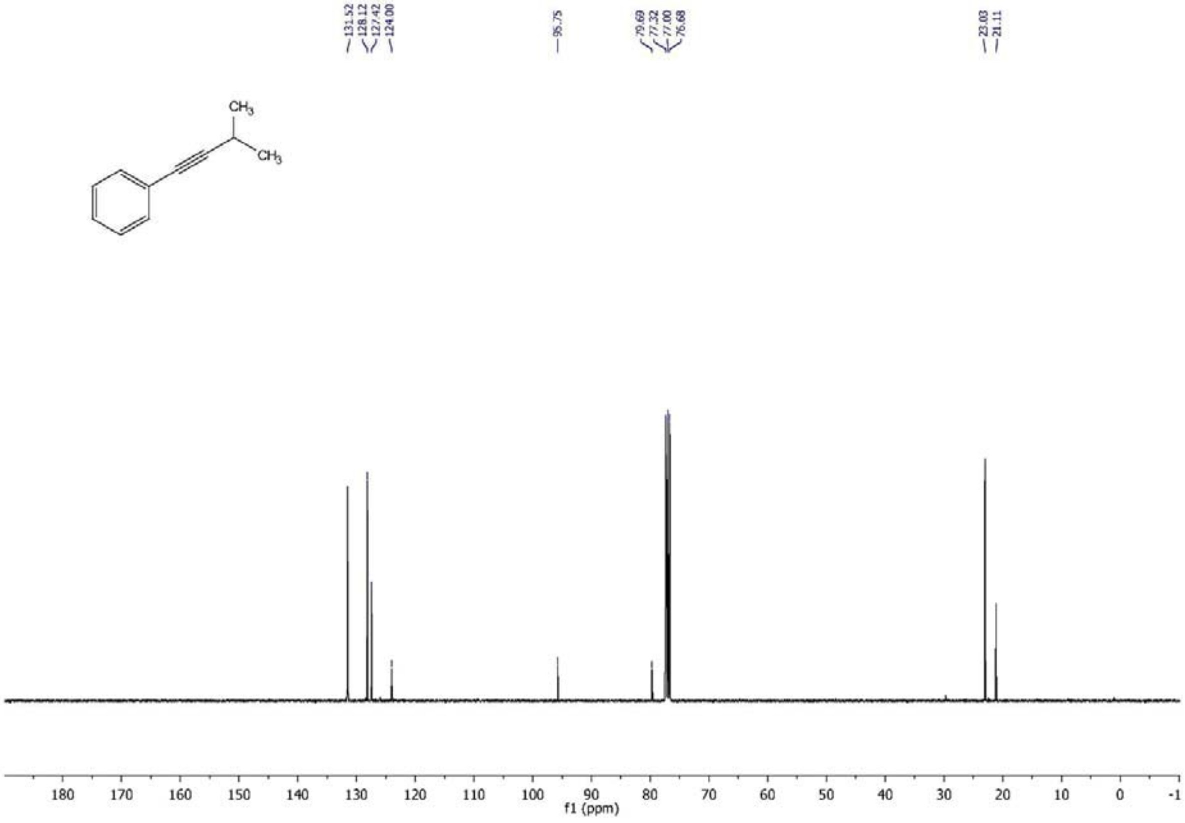

hex-1-yn-1-ylbenzene (5g)

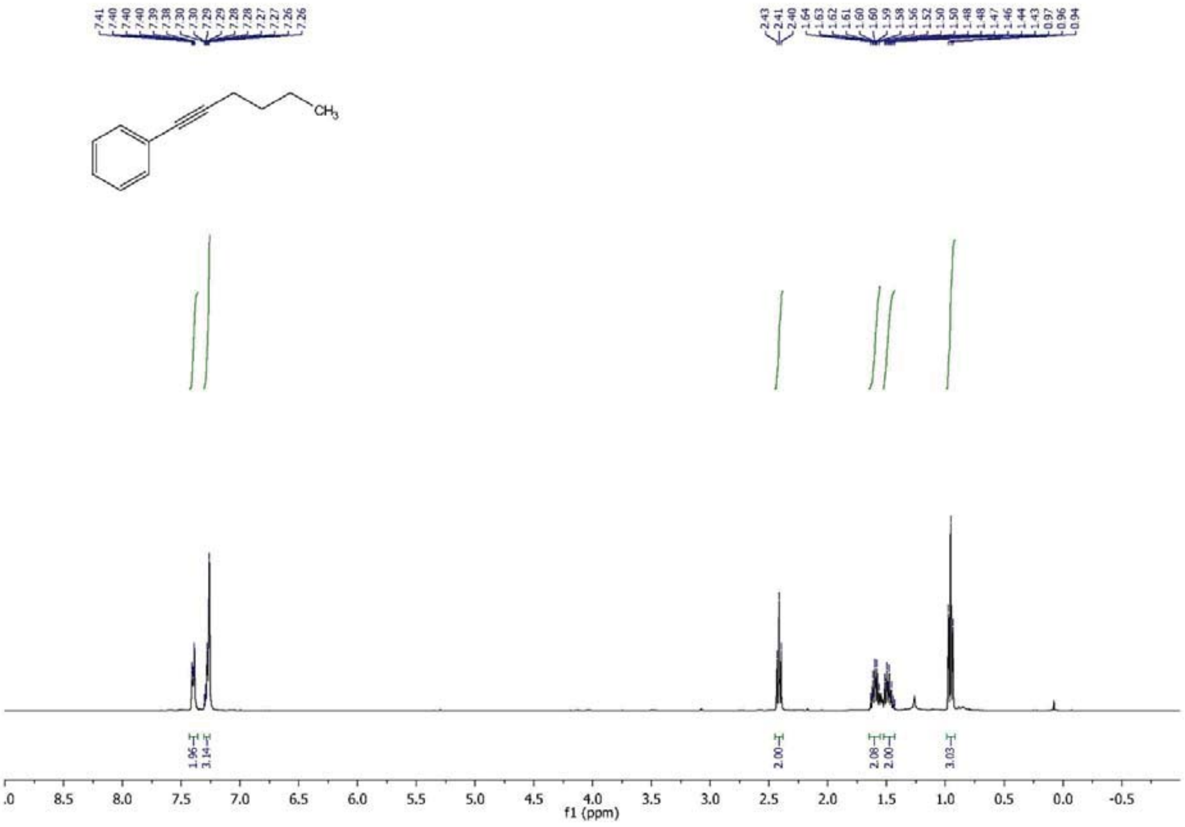

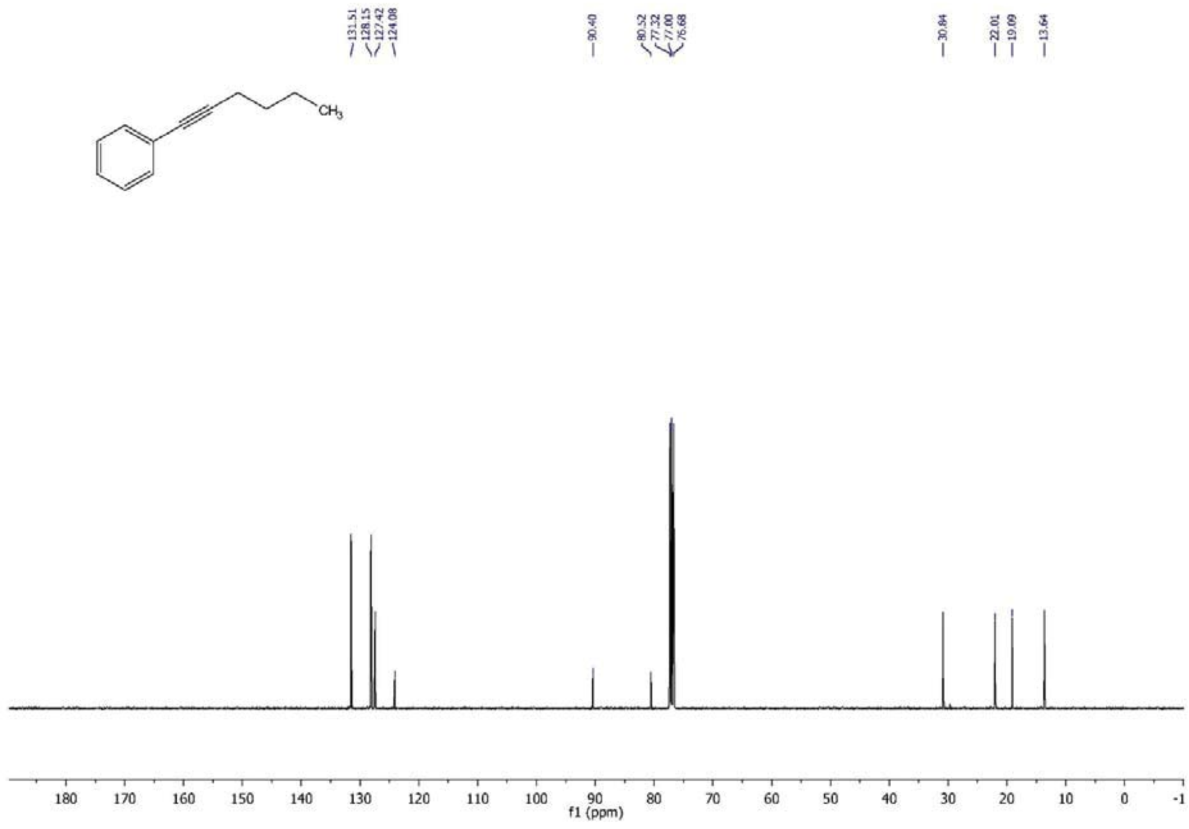

(5,5,5-trifluoropent-1-yn-1-yl)benzene (5h)

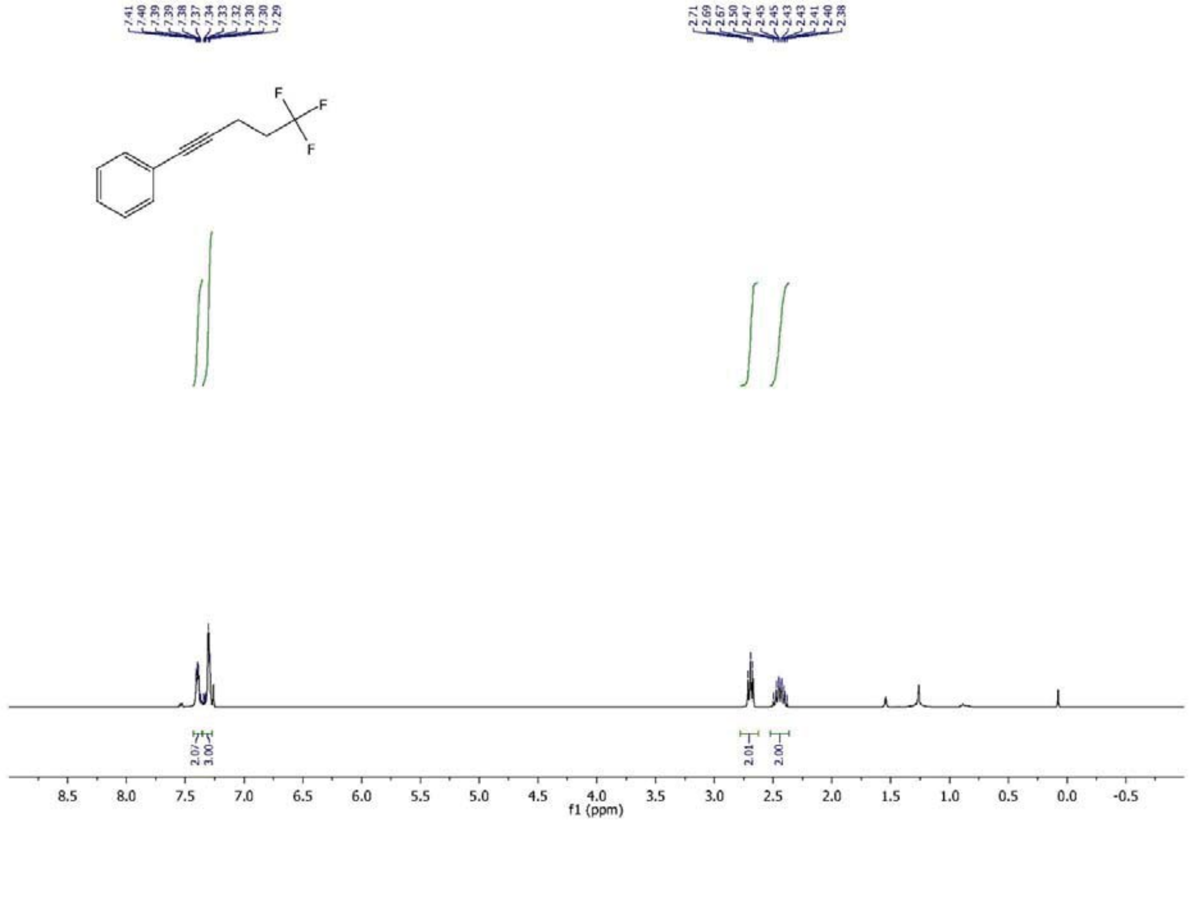

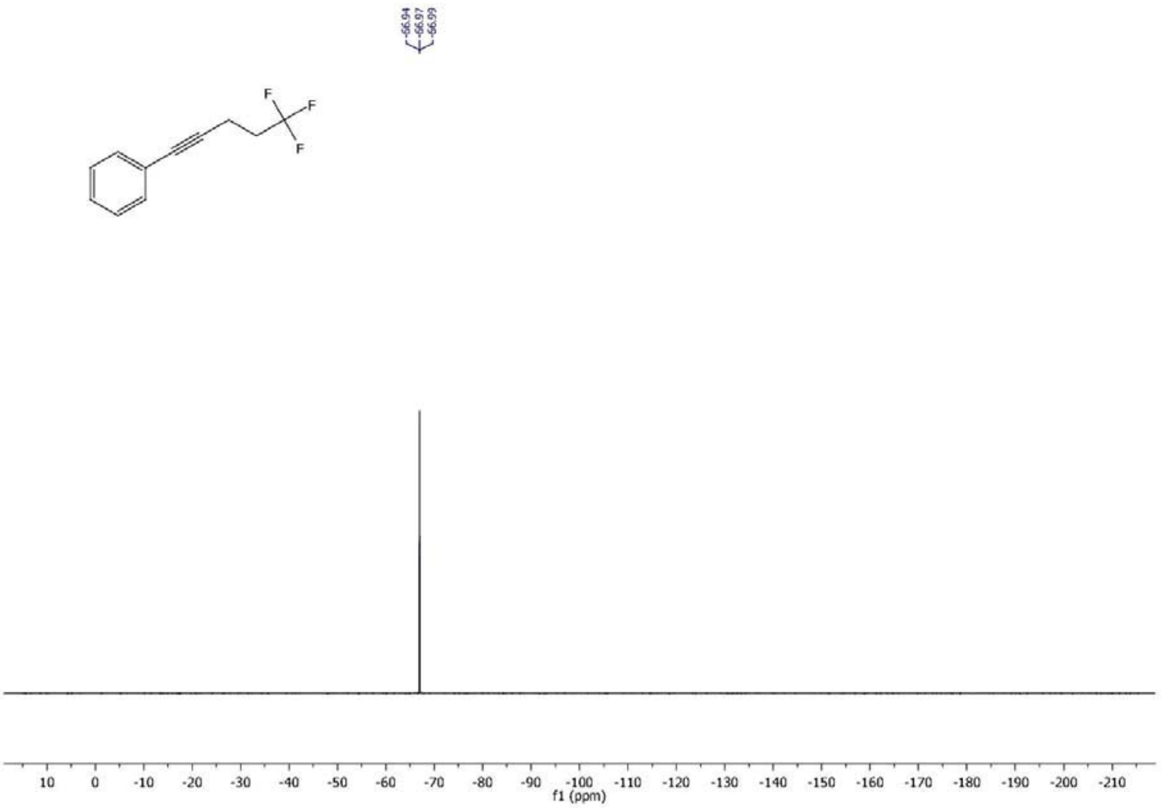
-66.94
-66.97
-66.99
F
F
F
10 0 -10 -20 -30 -40 -50 -60 -70 -80 -90 -100 -110 -120 -130 -140 -150 -160 -170 -180 -190 -200 -210
f1 (ppm)

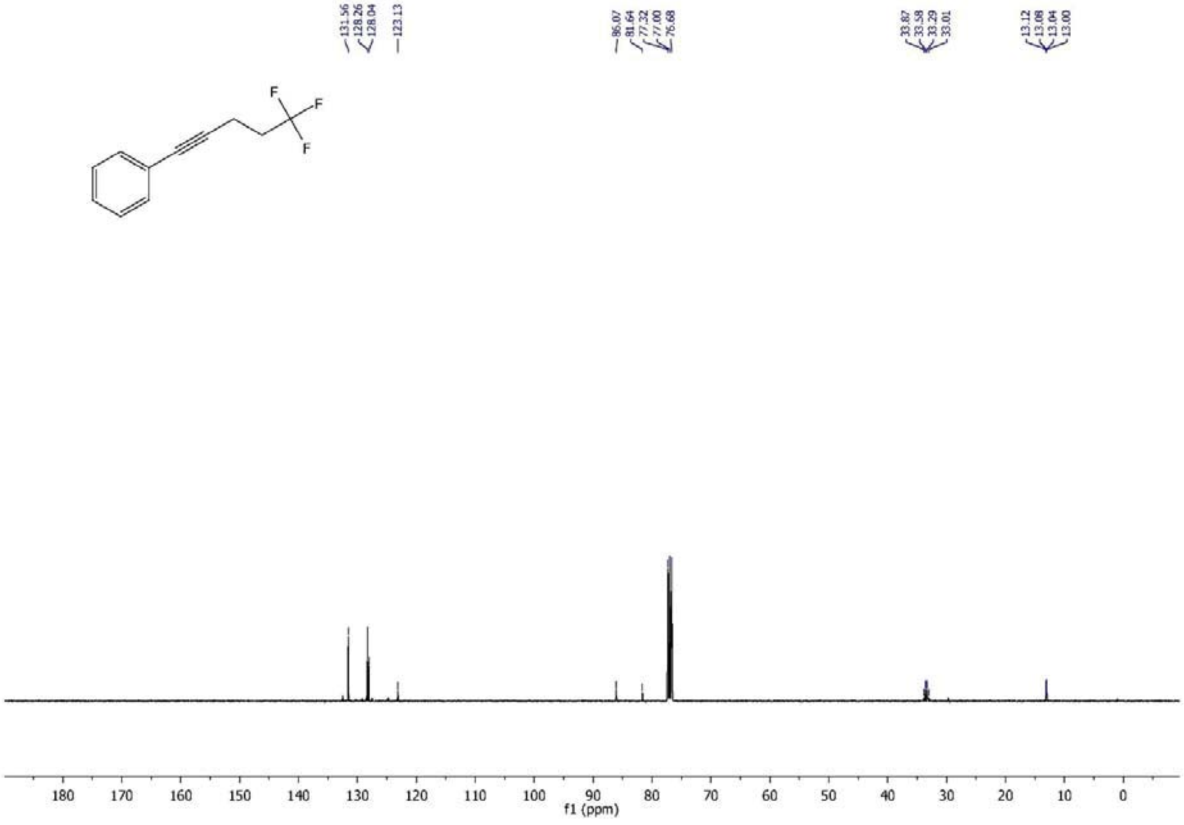
131.56
128.26
128.04
123.13
86.07
81.64
77.32
77.00
76.68
33.87
33.58
33.29
33.01
13.12
13.08
13.04
13.00
F
F
F
180 170 160 150 140 130 120 110 100 90 80 70 60 50 40 30 20 10 0
f1 (ppm)

pent-1-yne-1,5-diyldibenzene (5i)

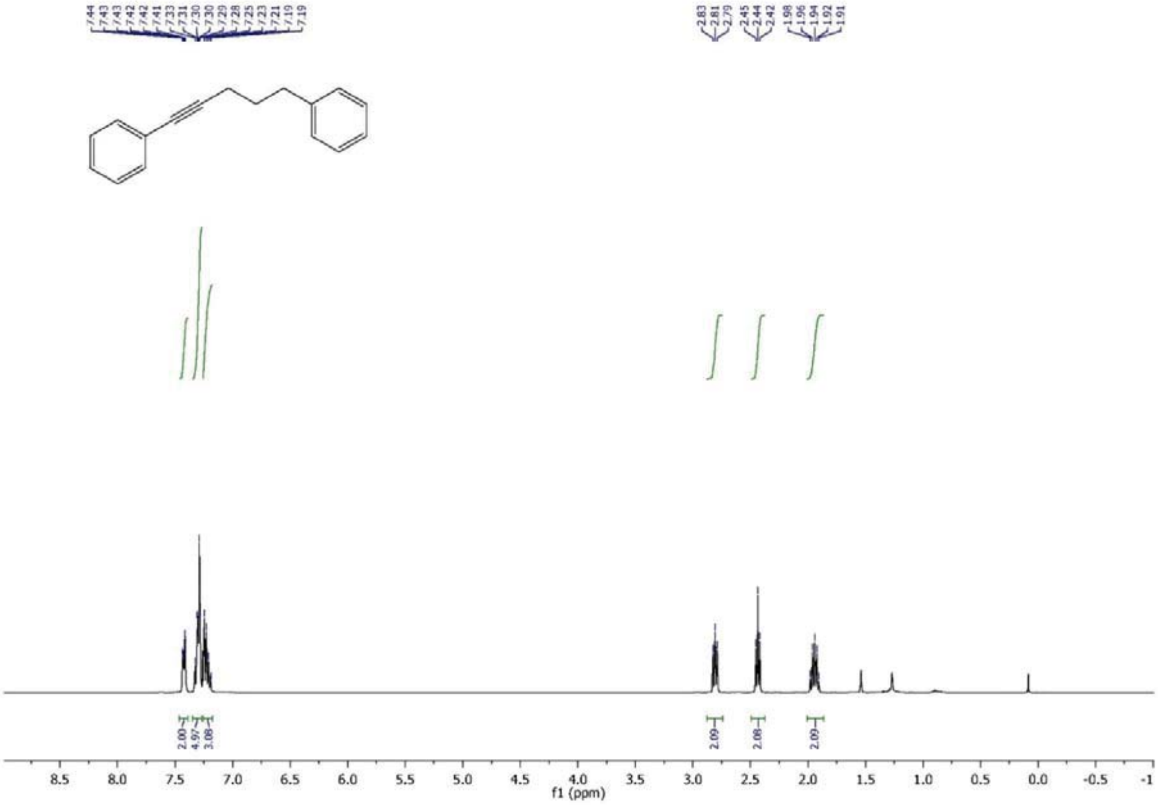

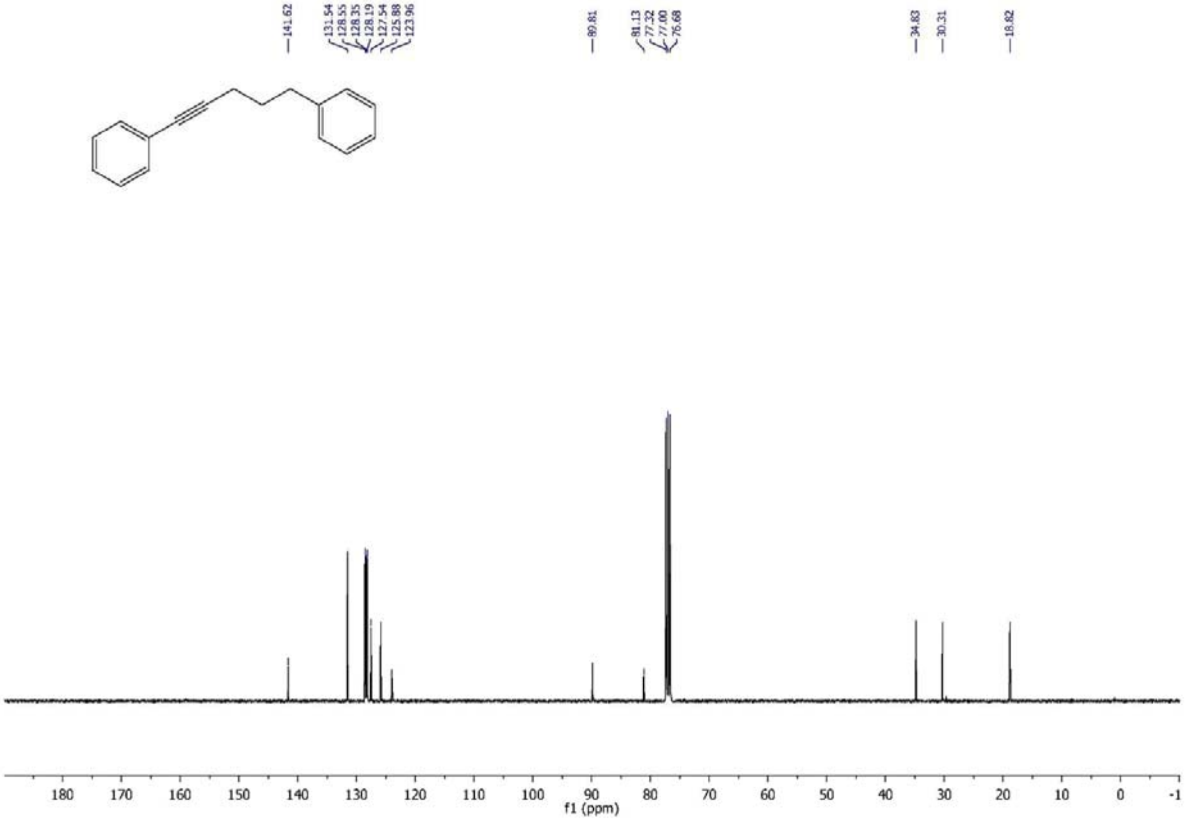

1-methyl-3-(phenylethynyl)benzene (5j)

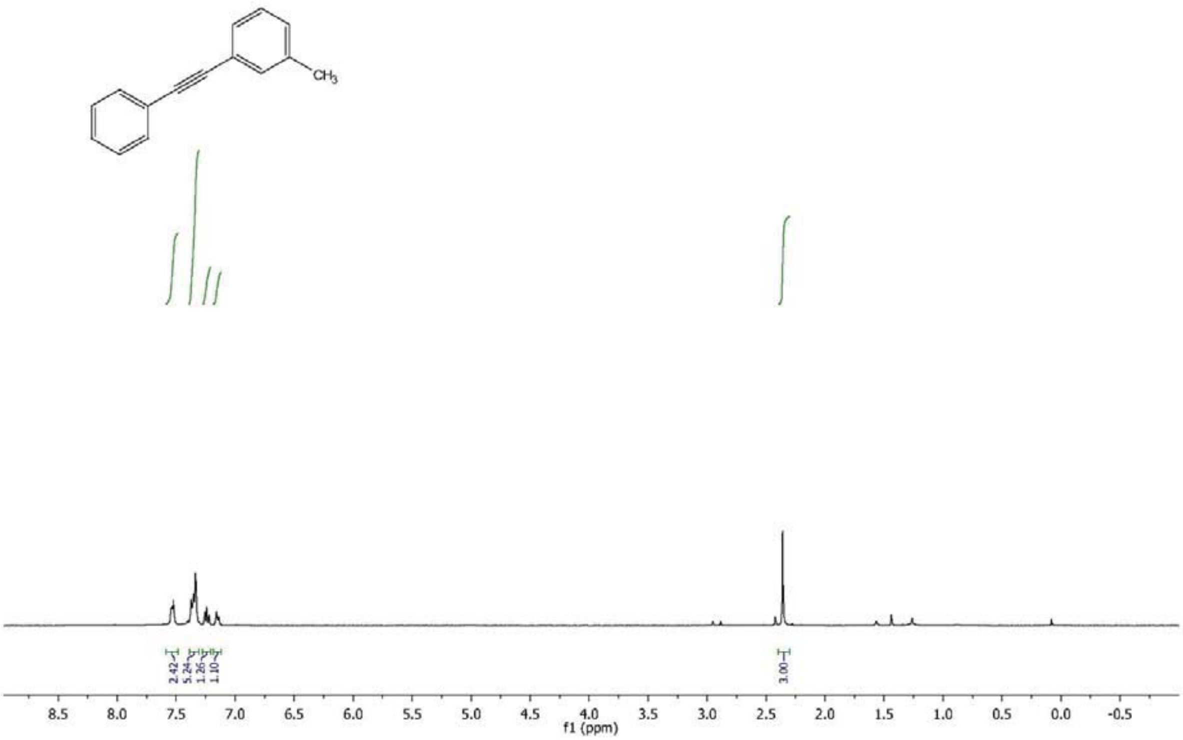

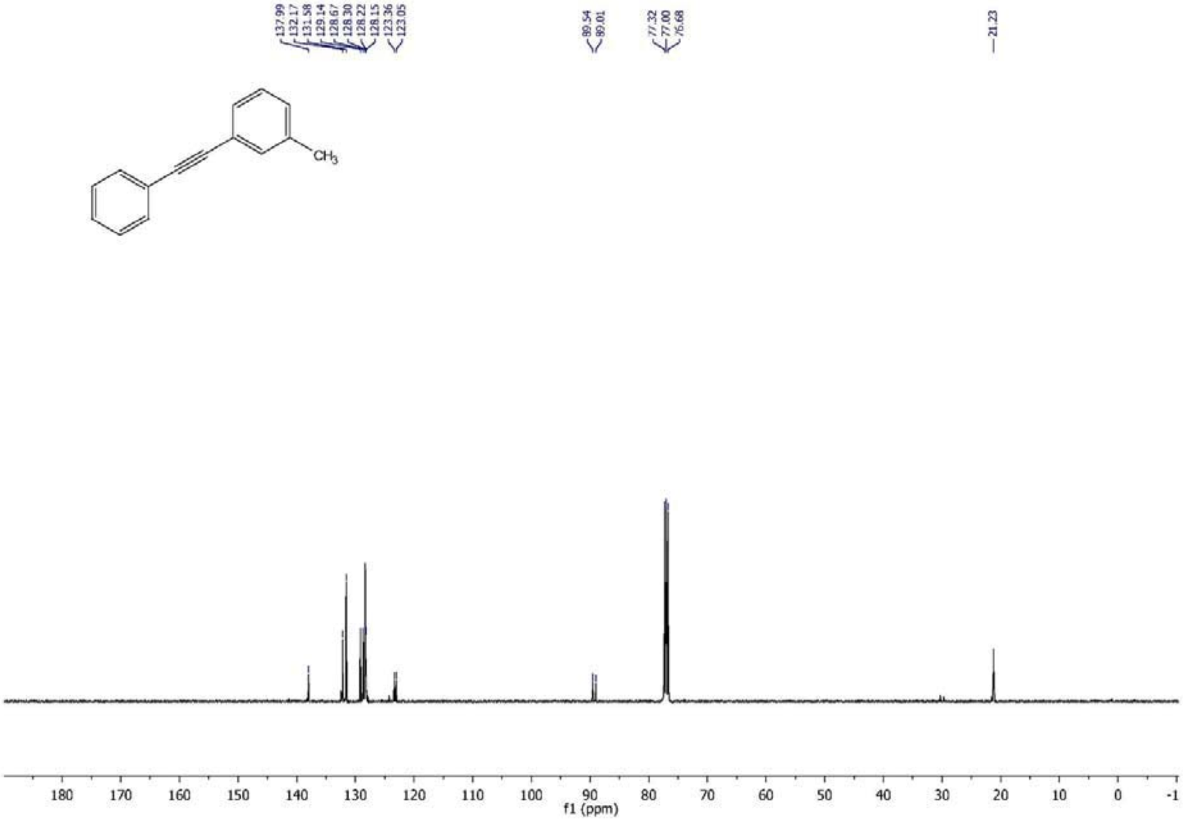

1-(phenylethynyl)-3-(trifluoromethyl)benzene (5k)

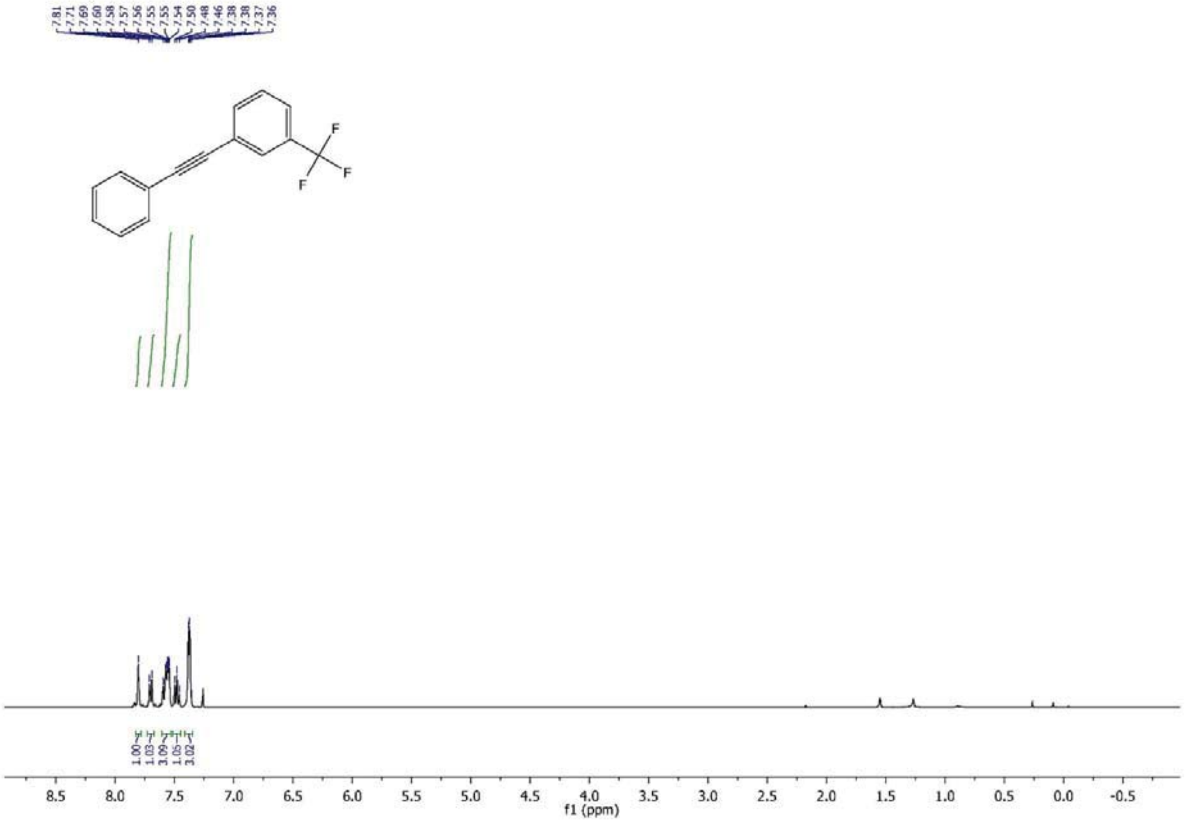

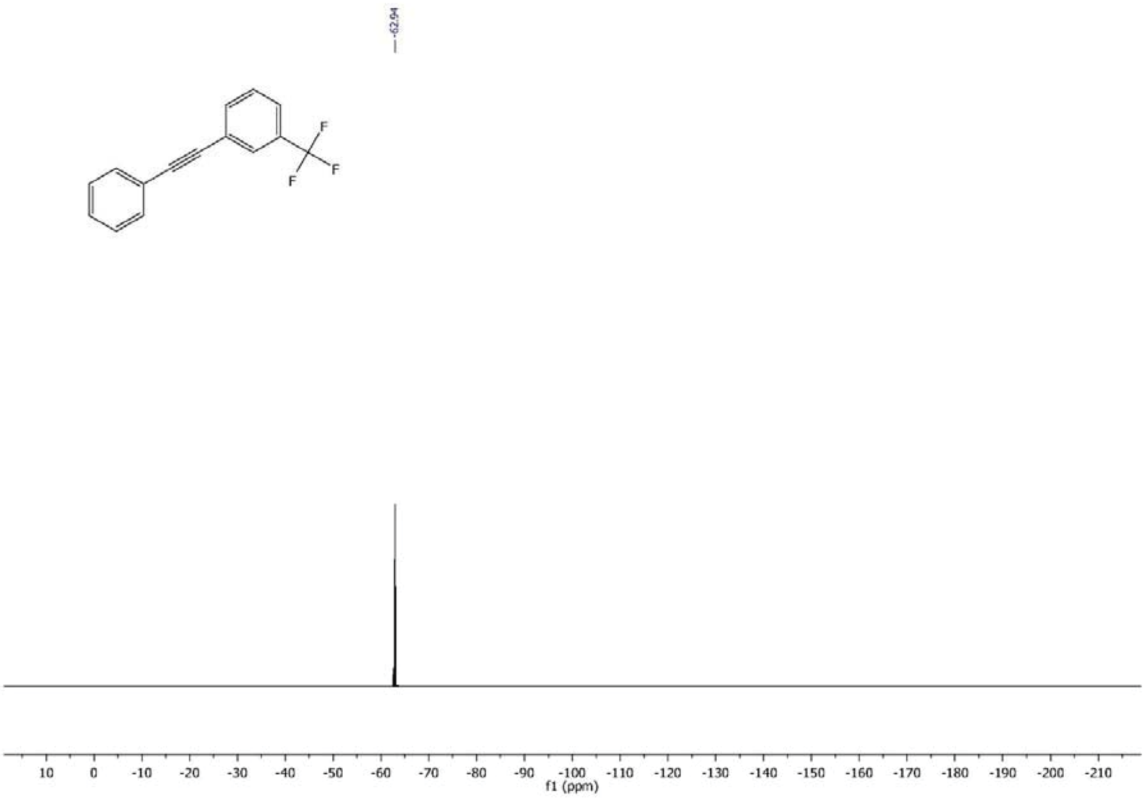

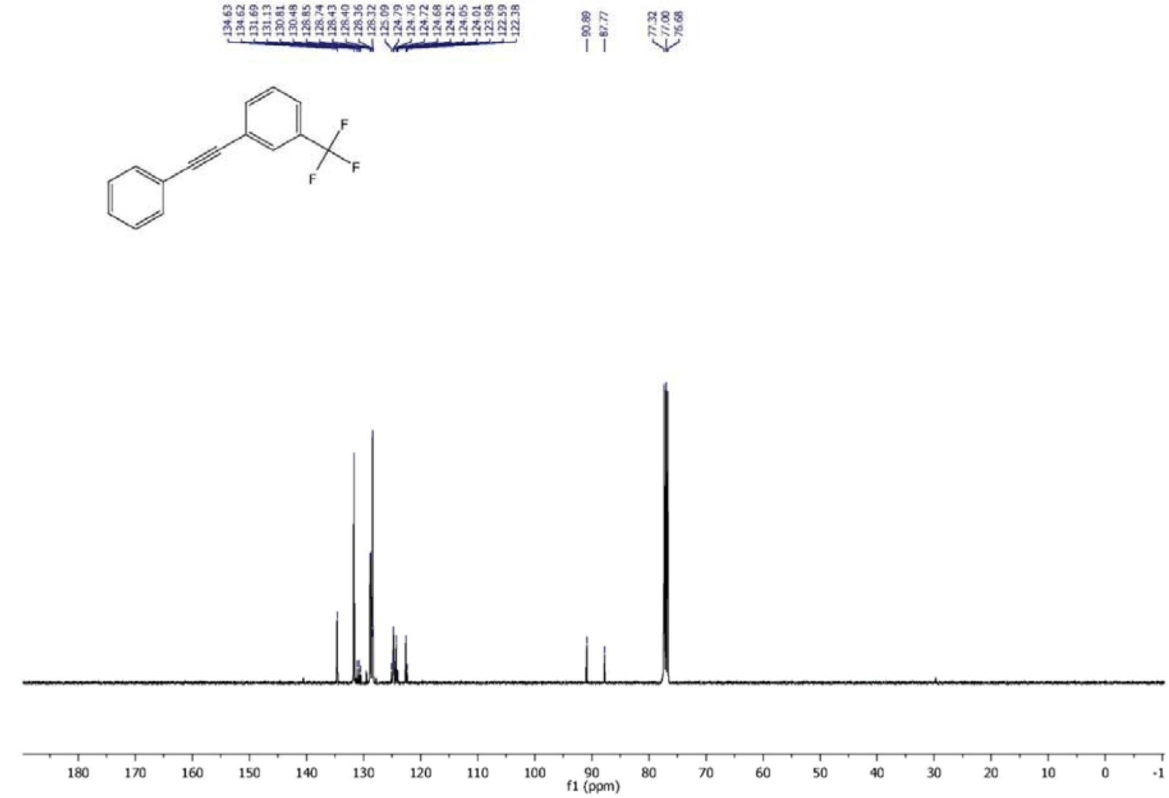

(chloroethynyl)benzene (1a)

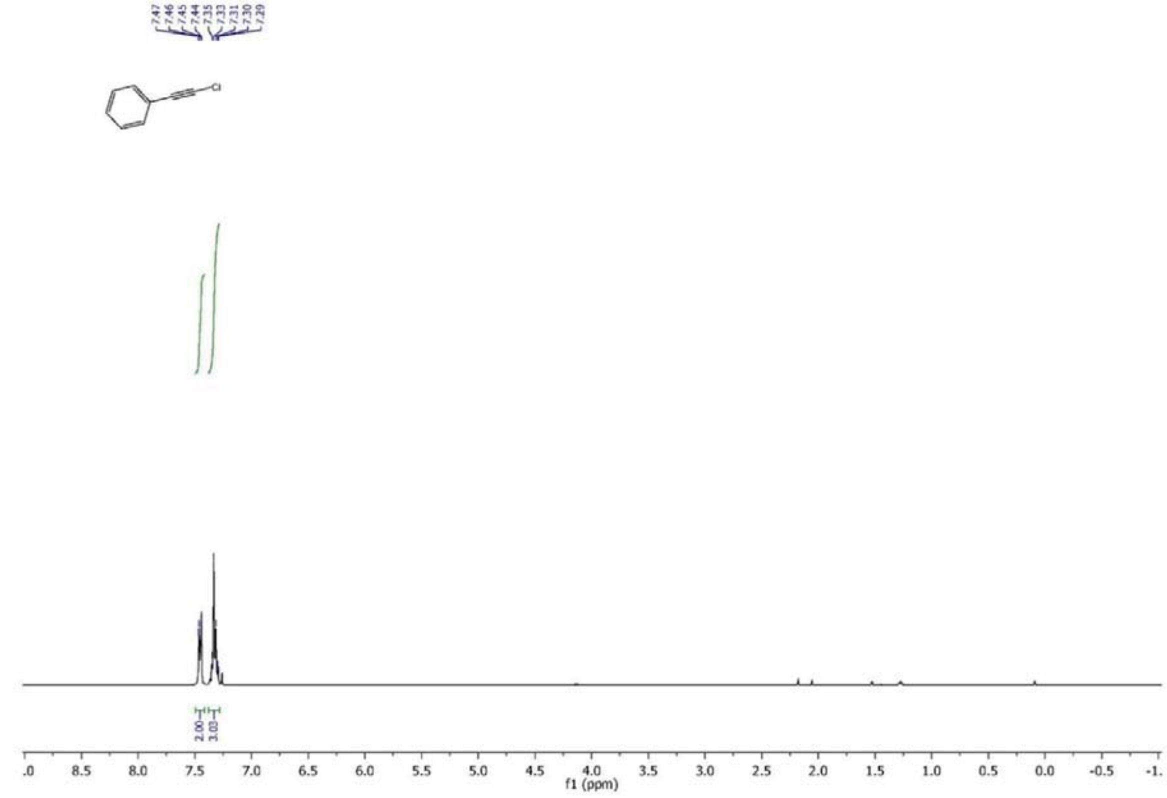

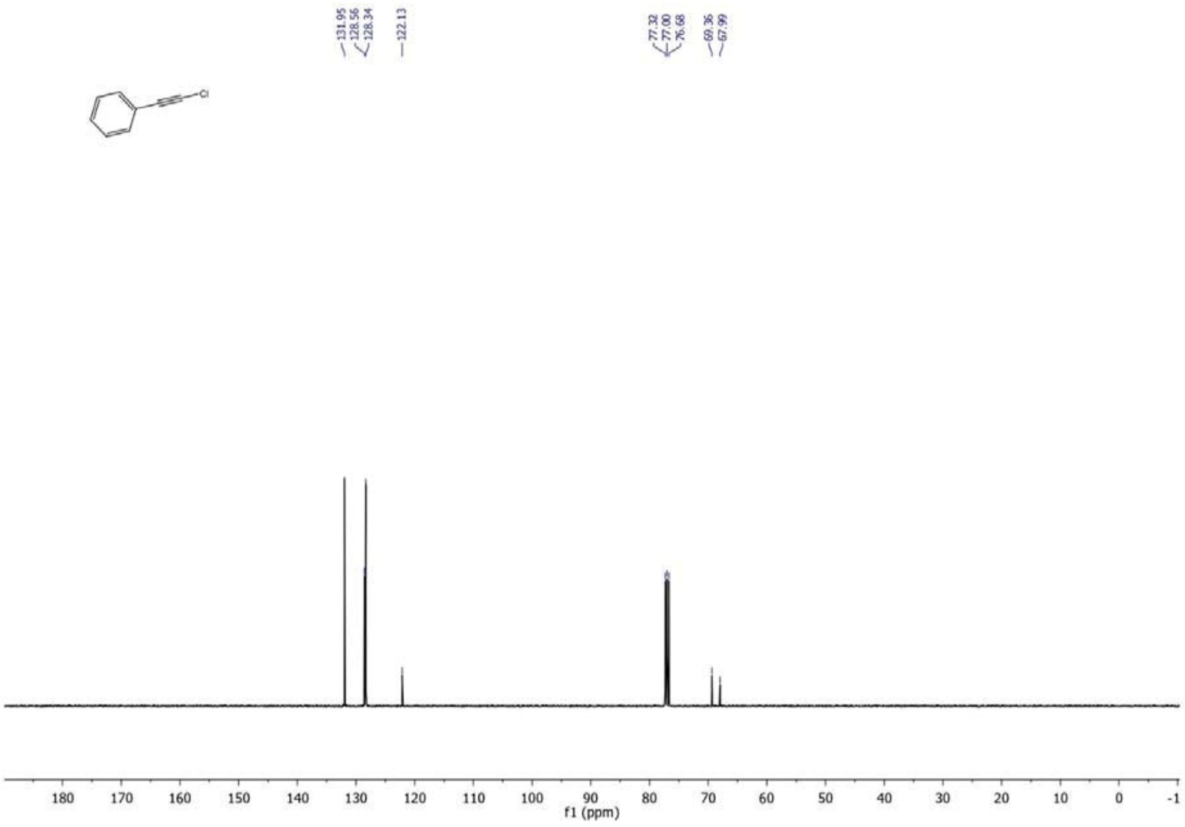

1-(chloroethynyl)-2-fluorobenzene (1b)

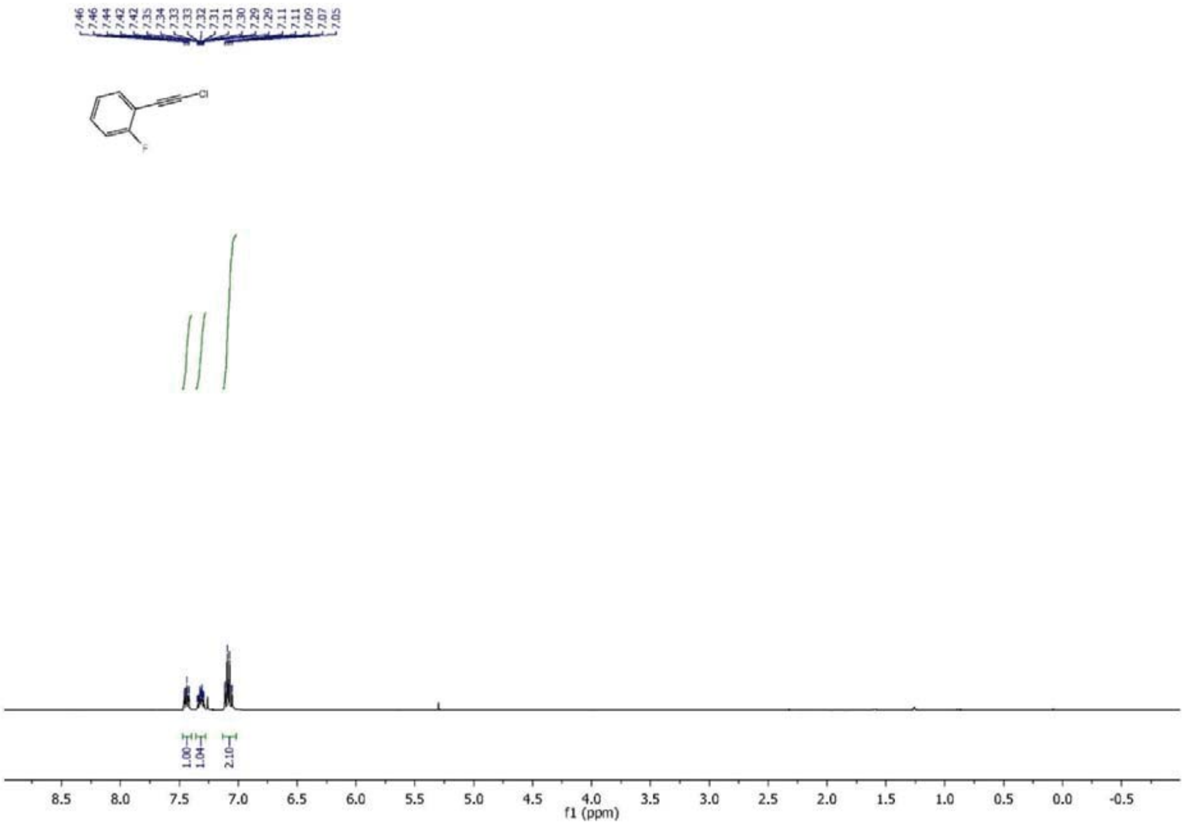

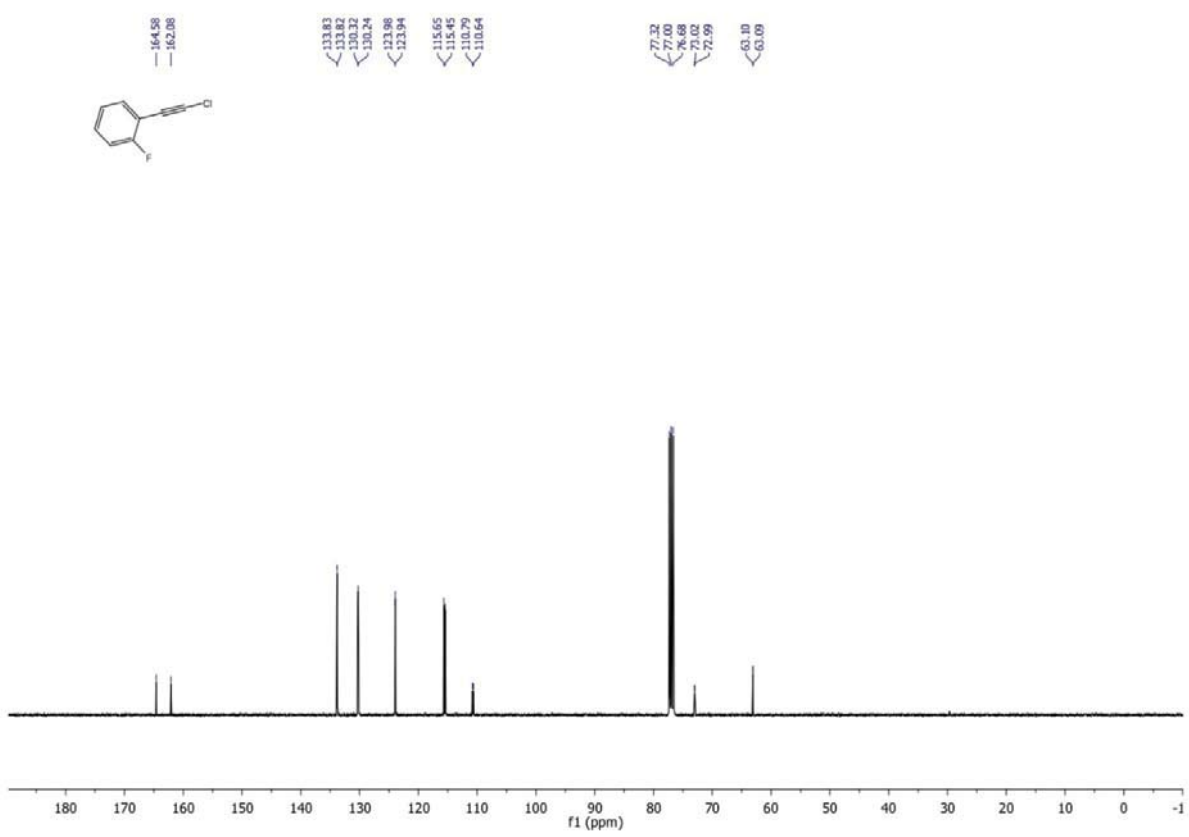
164.58
162.08
133.83
133.82
130.32
130.24
123.98
123.94
115.65
115.45
110.79
110.64
77.32
77.00
76.68
73.02
72.99
63.10
63.09
Cl
F
180 170 160 150 140 130 120 110 100 90 80 70 60 50 40 30 20 10 0 -1
f1 (ppm)

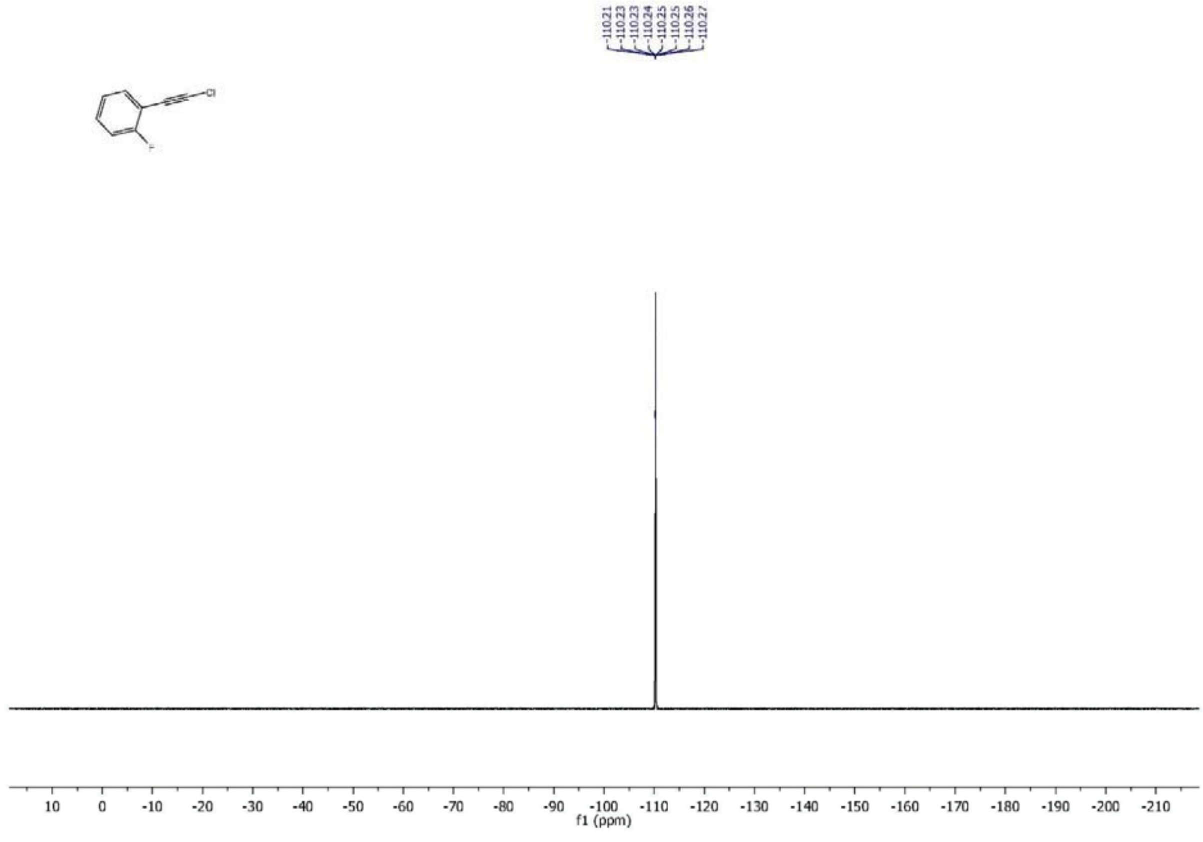
110.21
110.23
110.23
110.24
110.25
110.25
110.26
110.27
Cl
F
10 0 -10 -20 -30 -40 -50 -60 -70 -80 -90 -100 -110 -120 -130 -140 -150 -160 -170 -180 -190 -200 -210
f1 (ppm)

1-(chloroethynyl)-3-methylbenzene (1c)

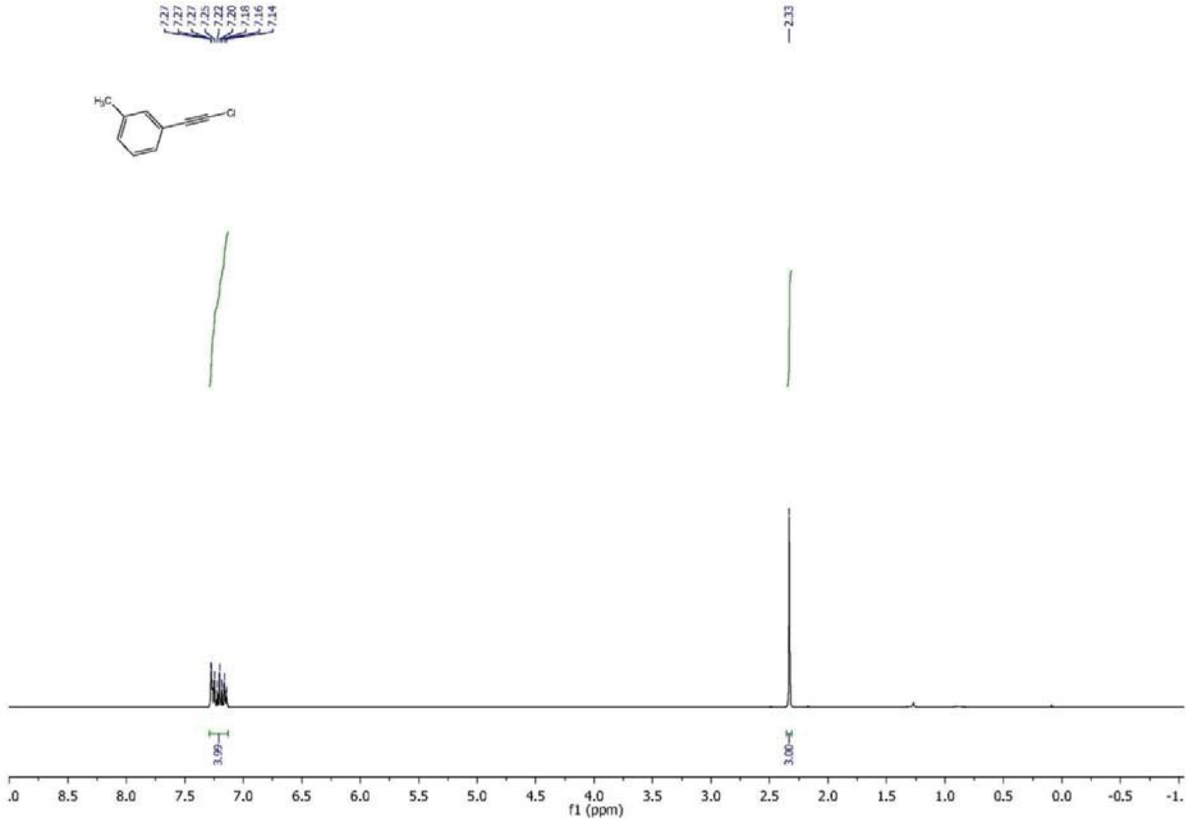

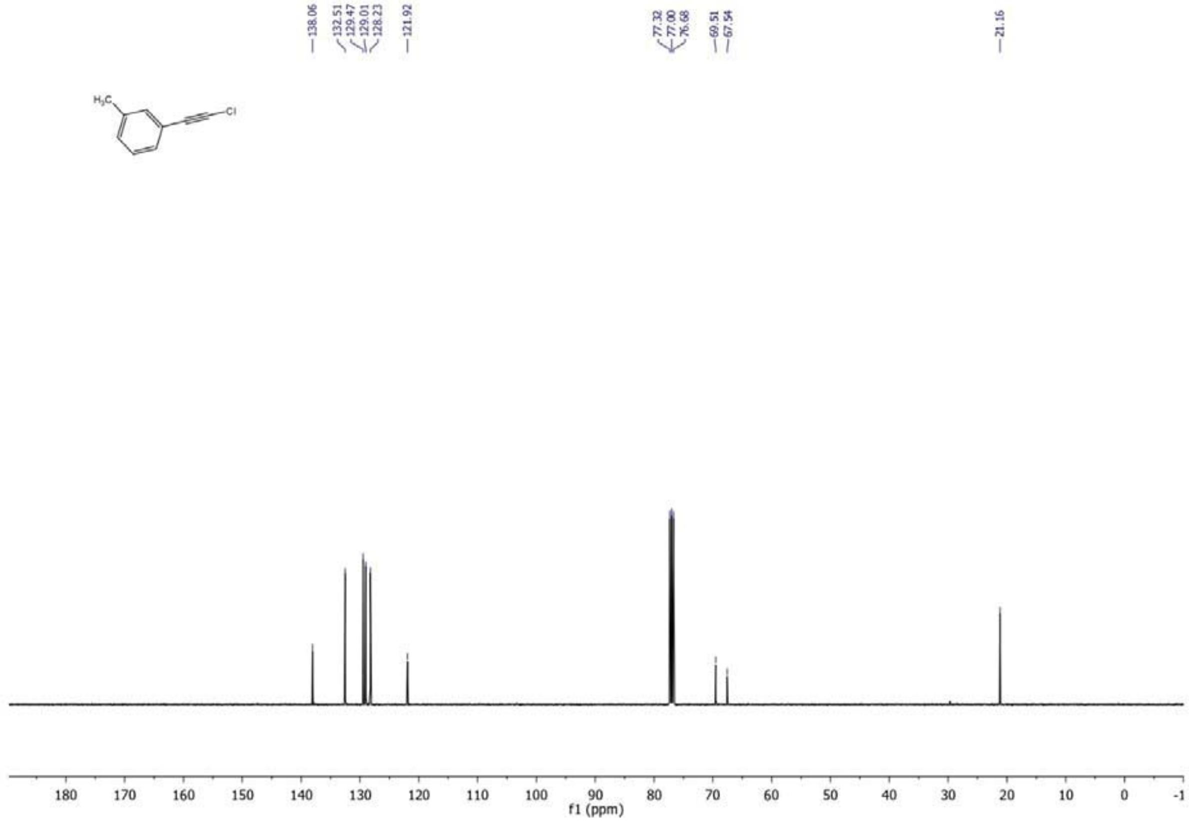

1-butyl-4-(chloroethynyl)benzene (1d)

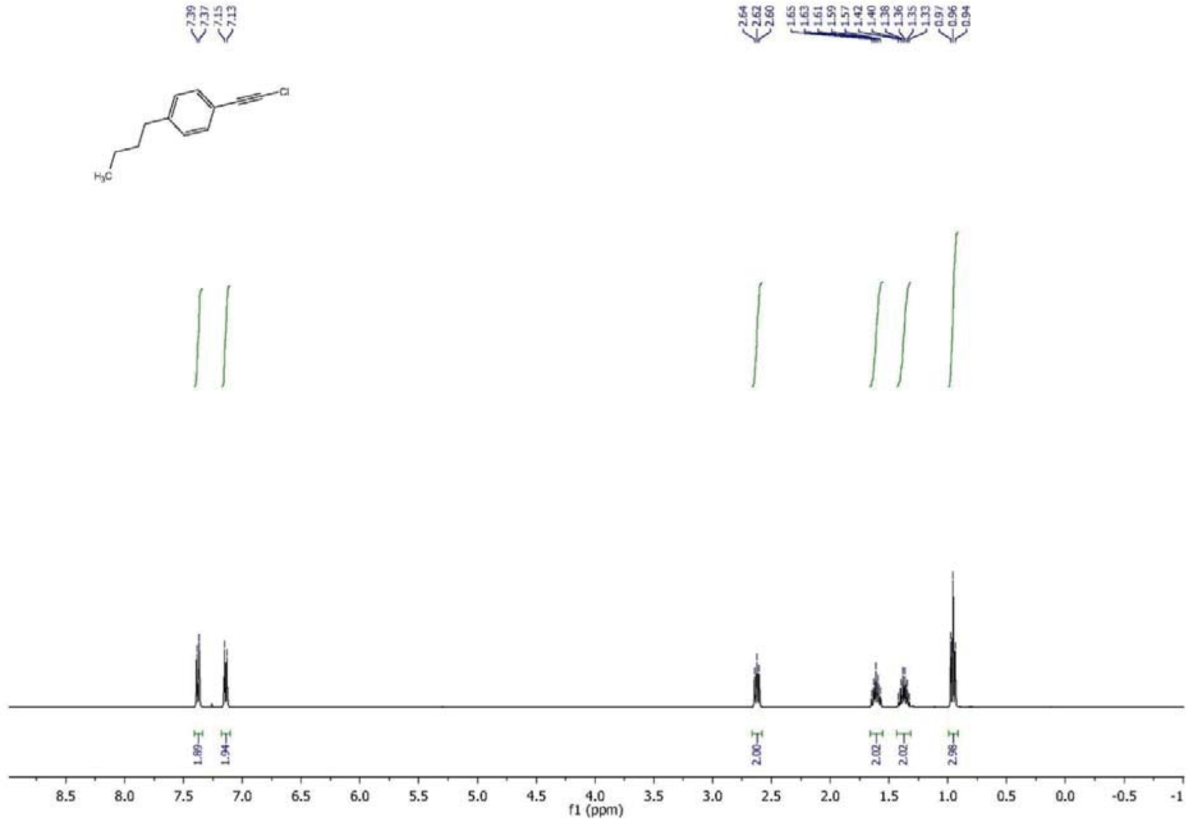

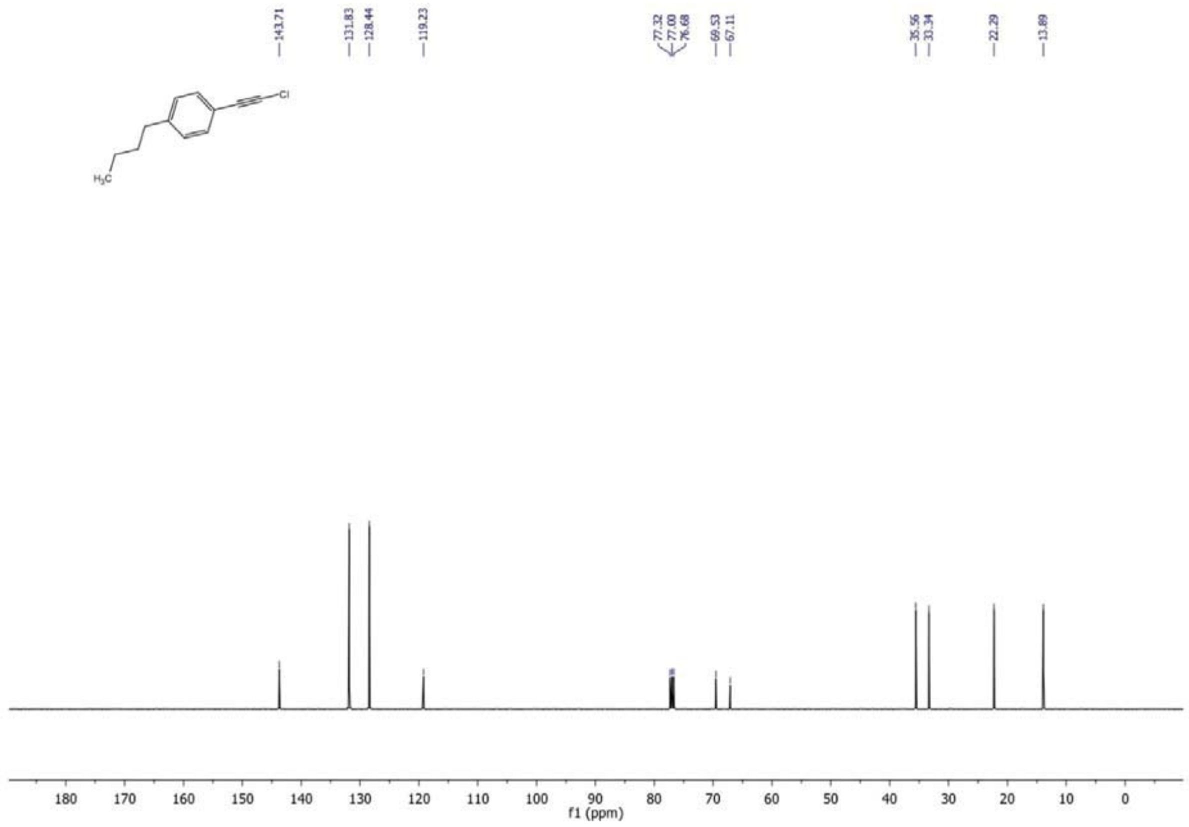

1-(chloroethynyl)-4-methoxybenzene (1e)

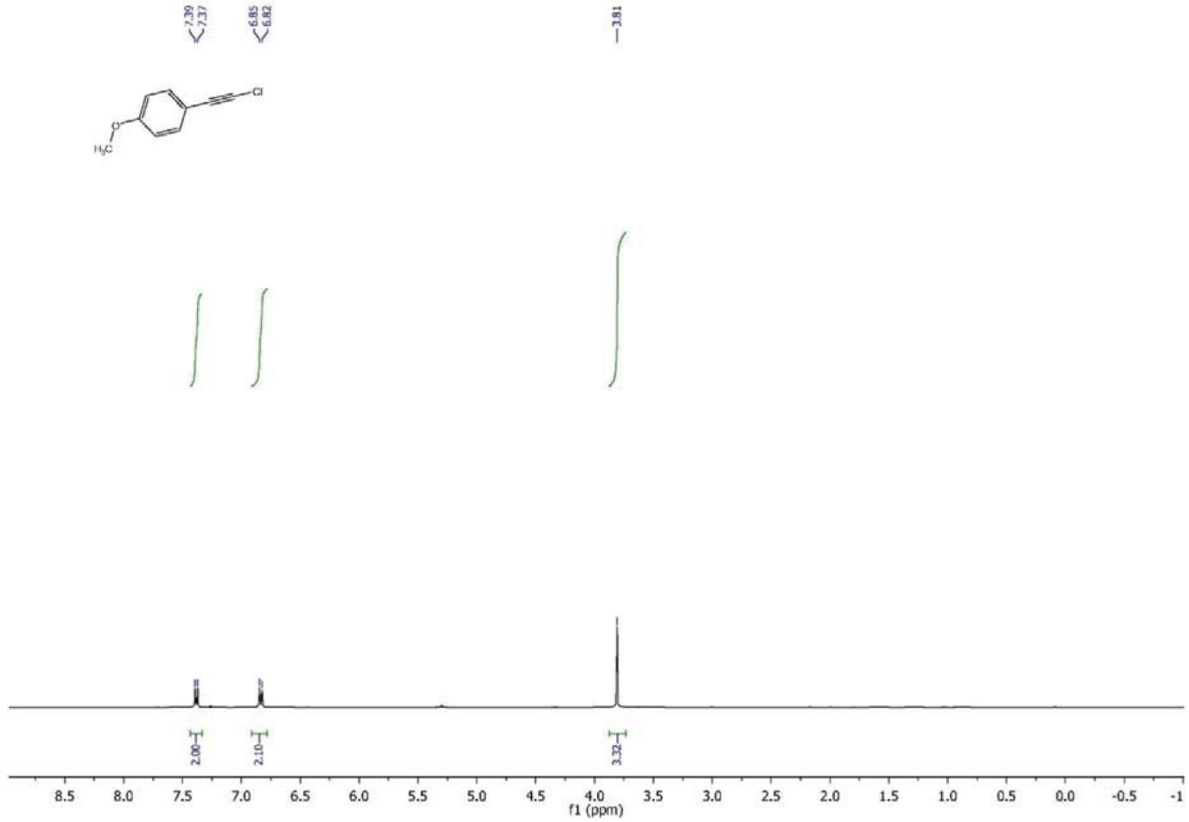

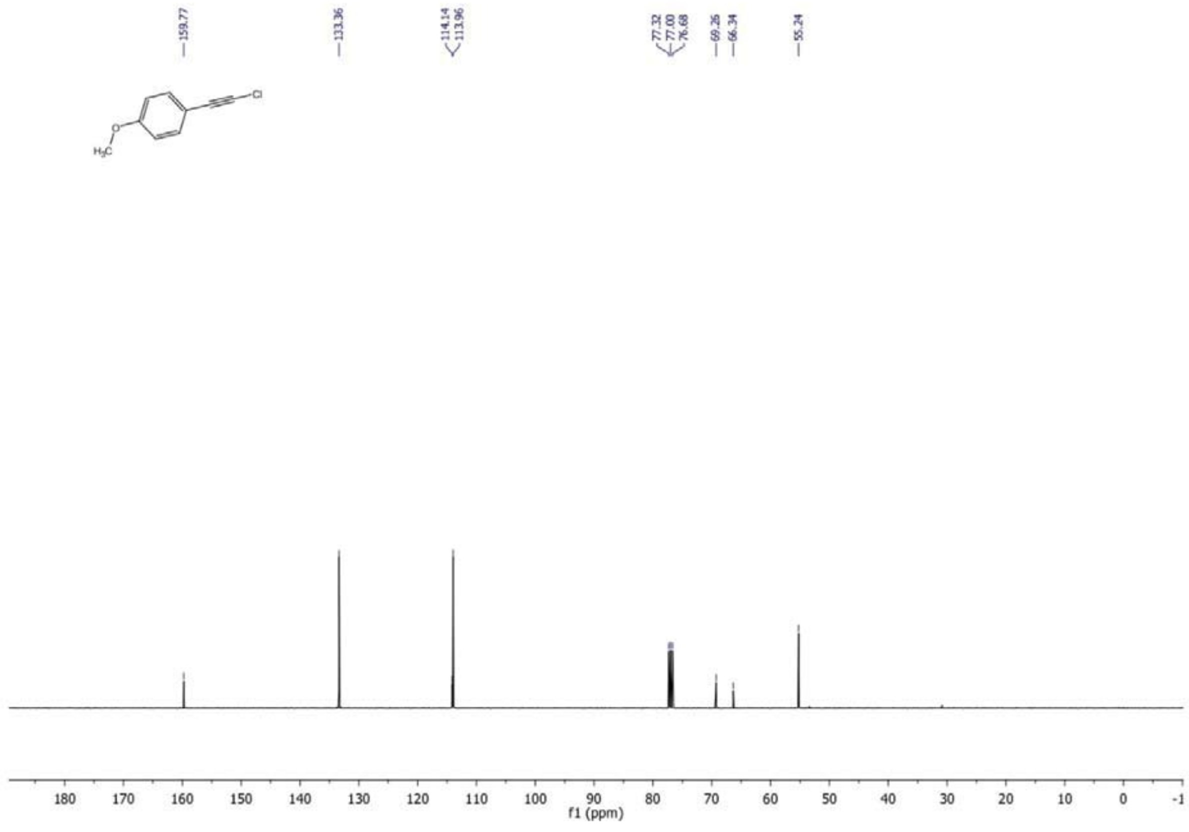

1-chlorooct-1-yne (1f)

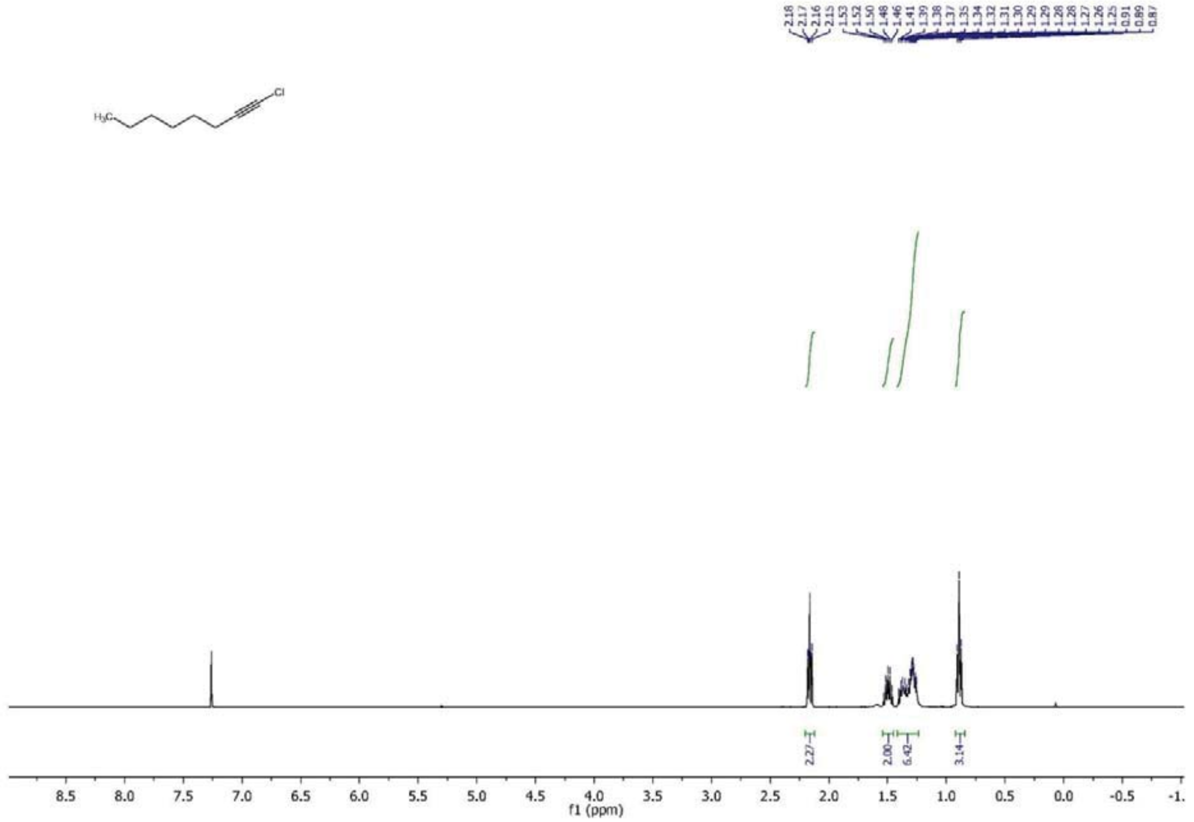

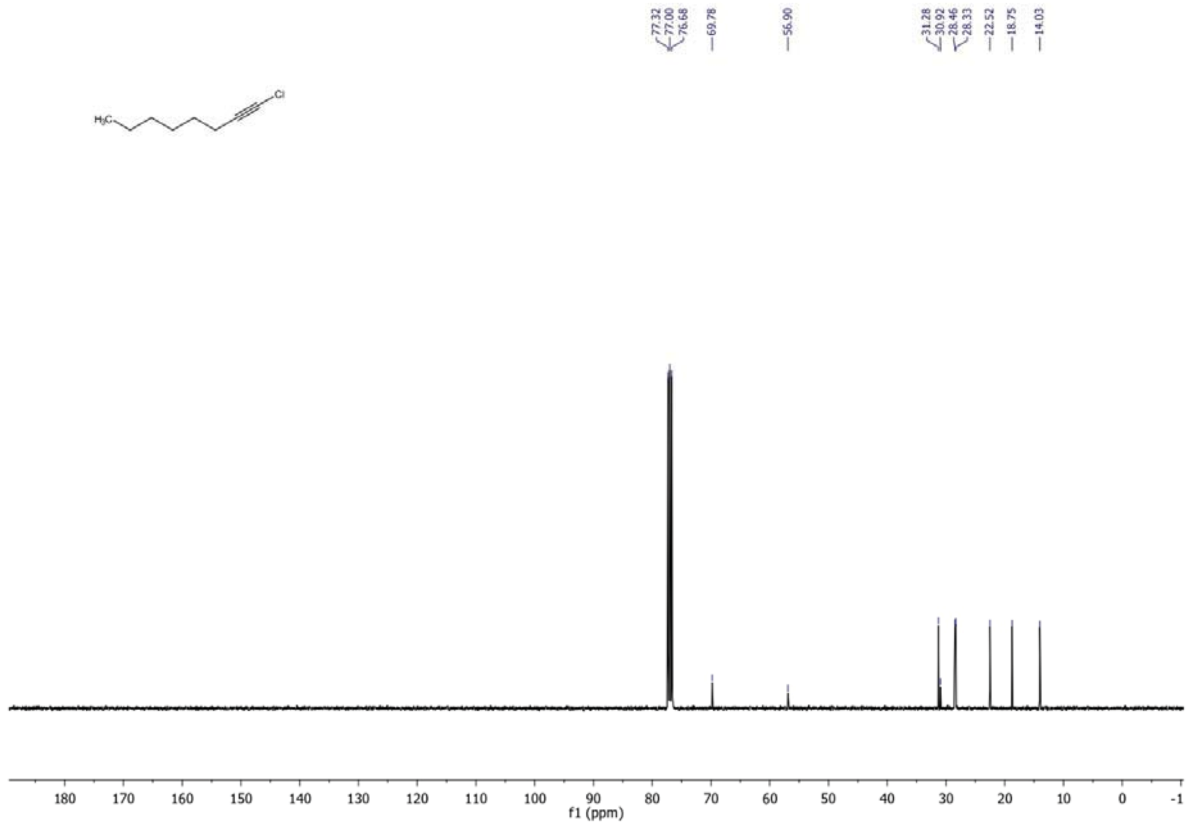

***(E)*-(2-chlorovinyl)benzene (3a)**

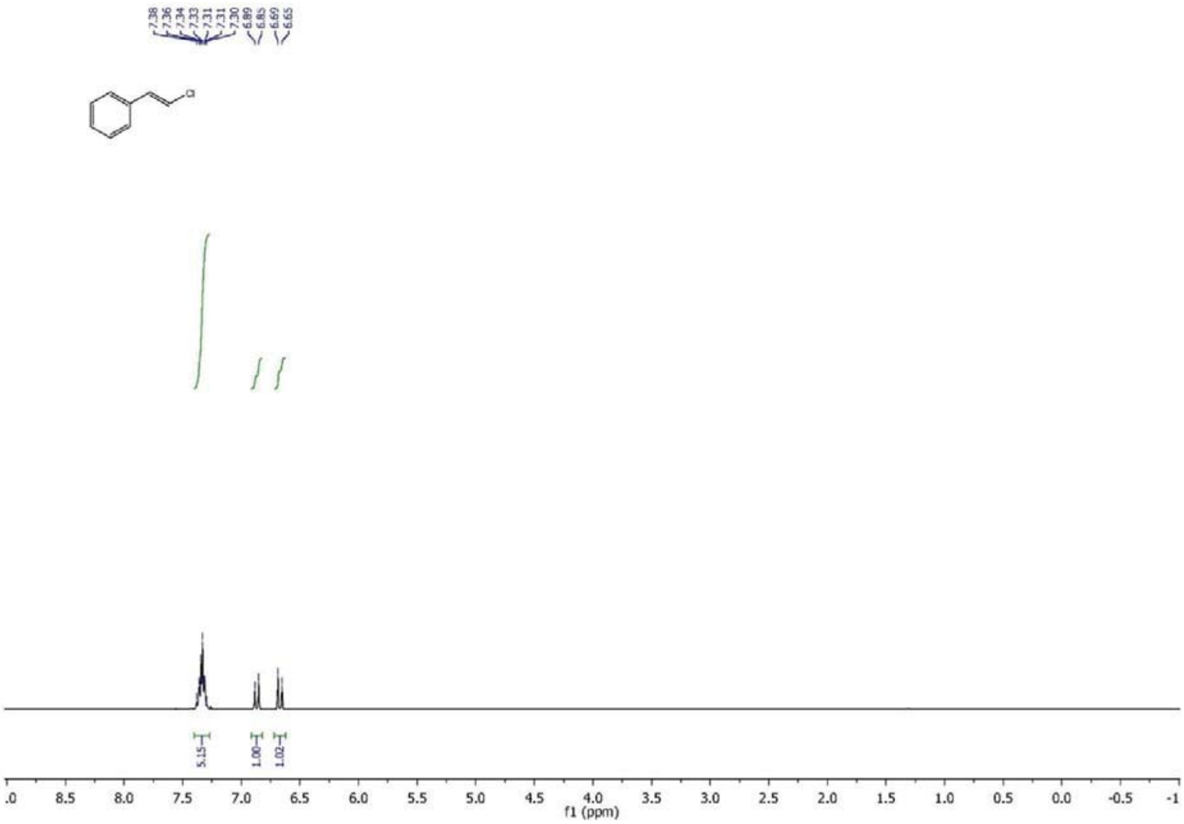

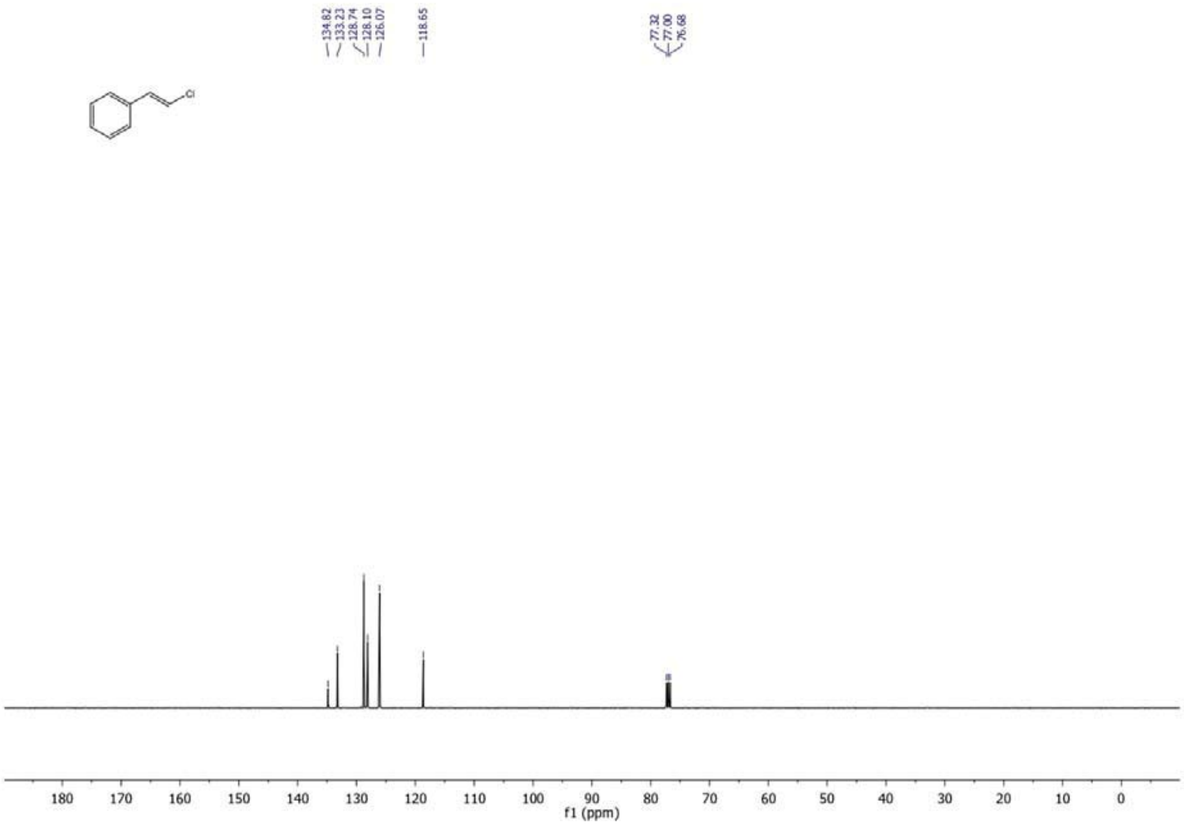

***(E)*-1-(2-chlorovinyl)-2-methylbenzene (3i)**

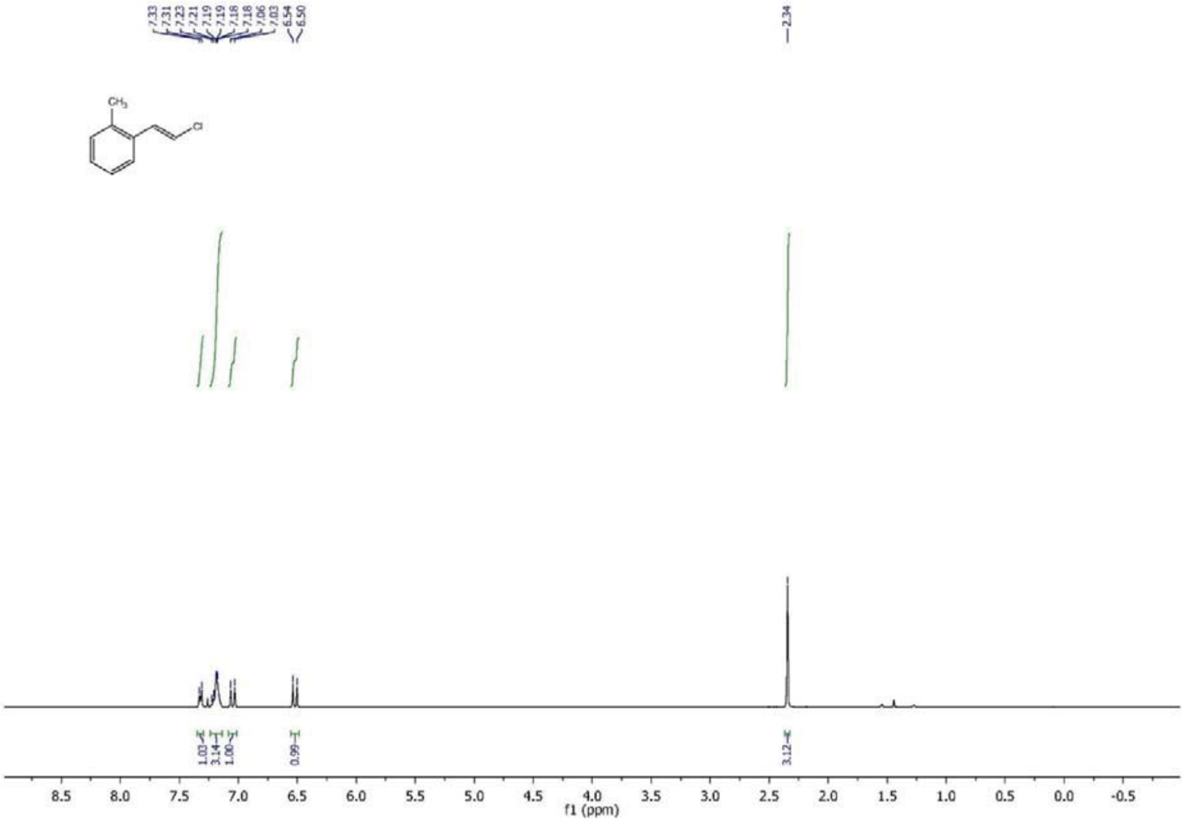

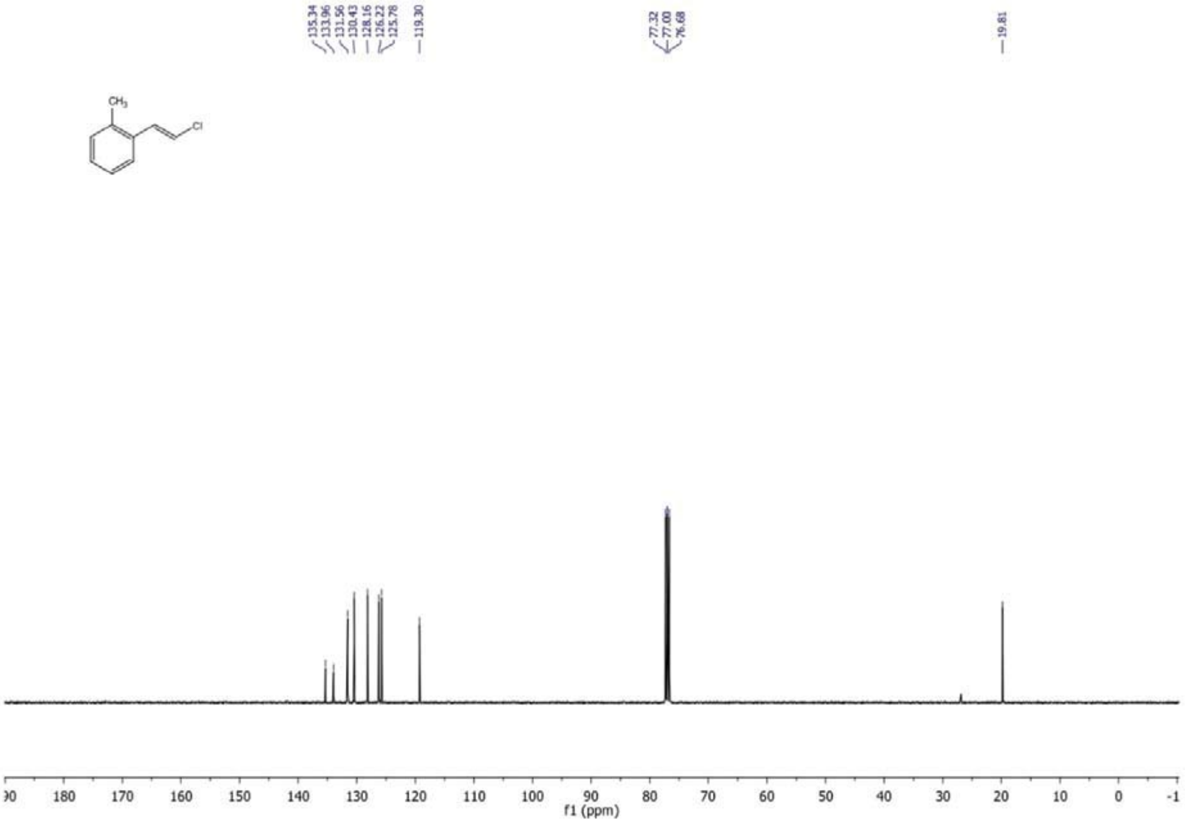

(E)-1-(2-chlorovinyl)-4-isopropylbenzene (3j)

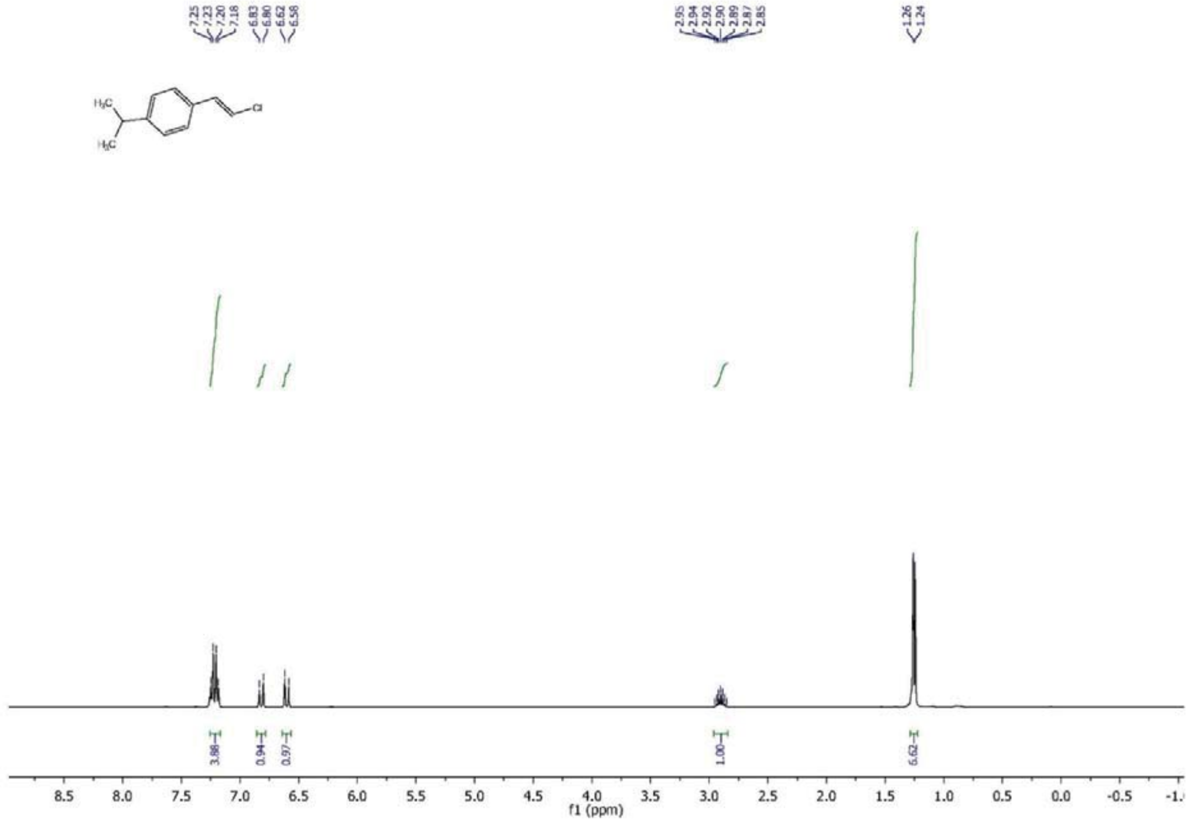

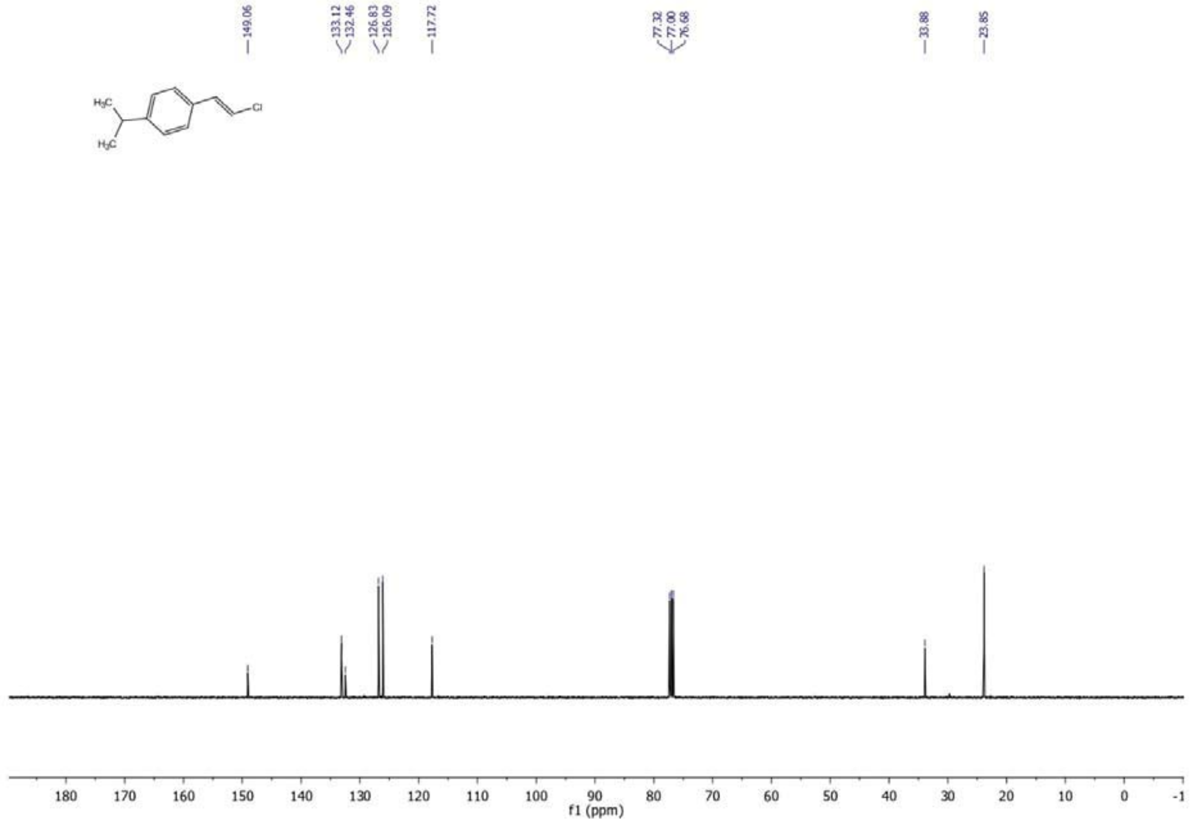

(E)-4-(2-chlorovinyl)-1,2-dimethylbenzene (3k)

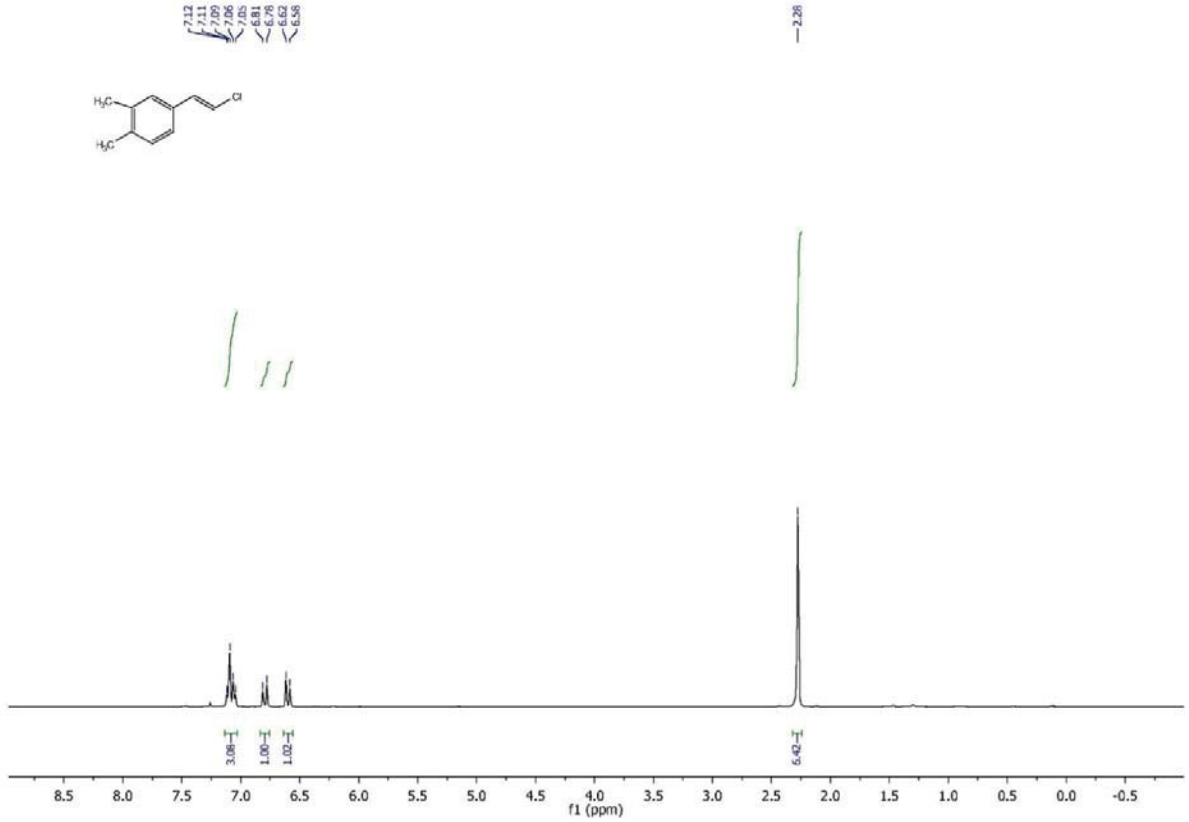

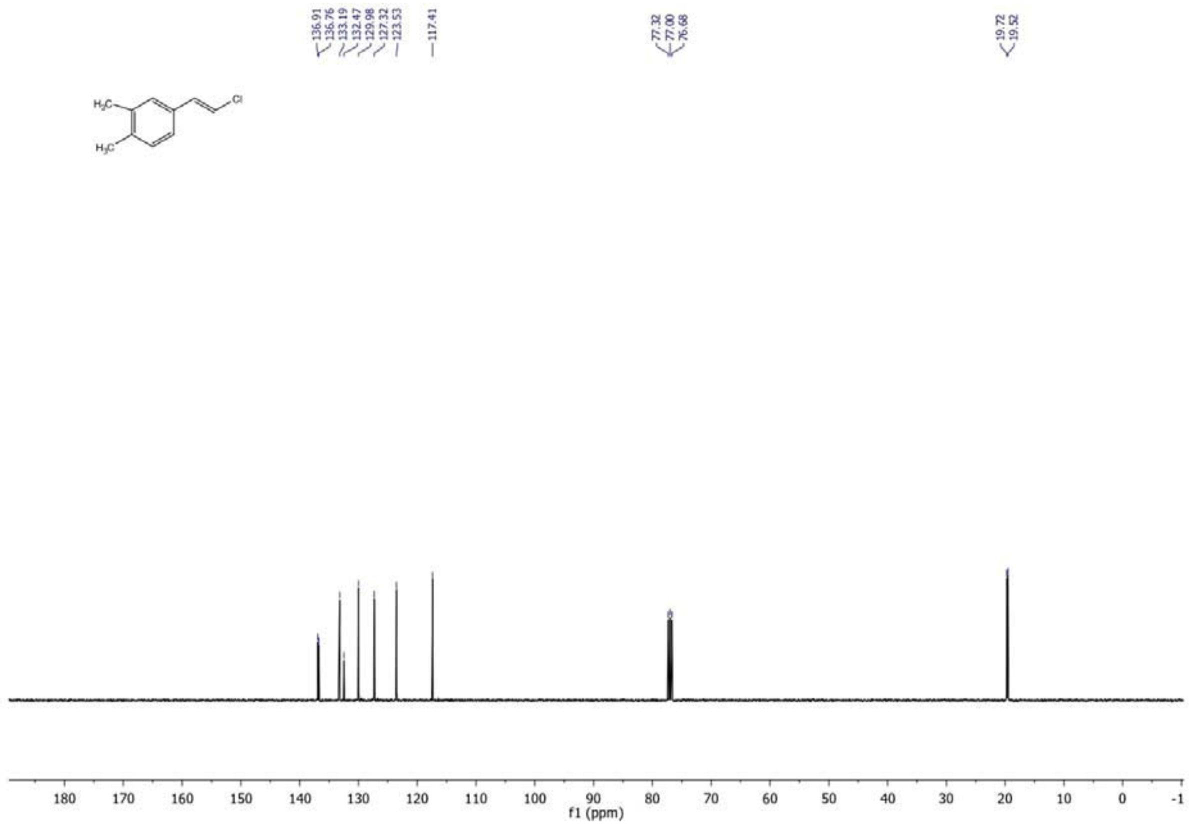

***(E)*-1-(2-chlorovinyl)-2,3,4,5,6-pentamethylbenzene (3l)**

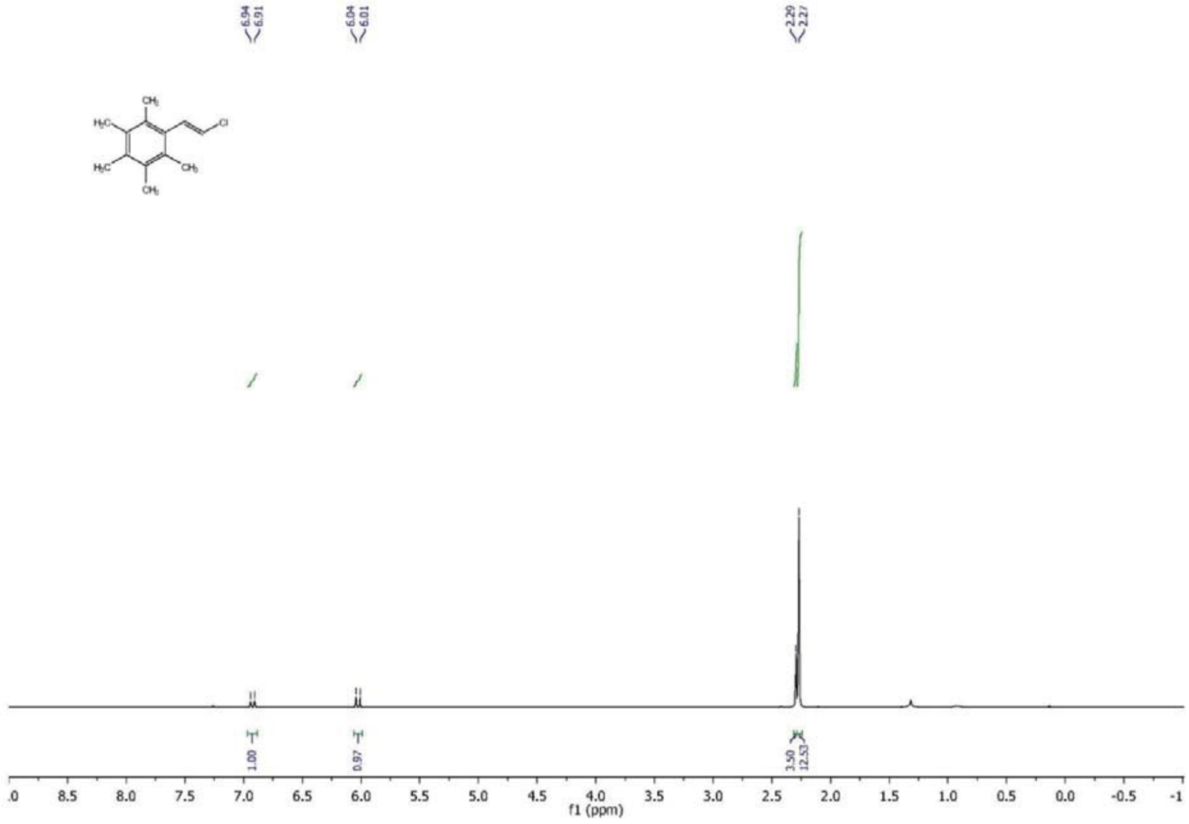

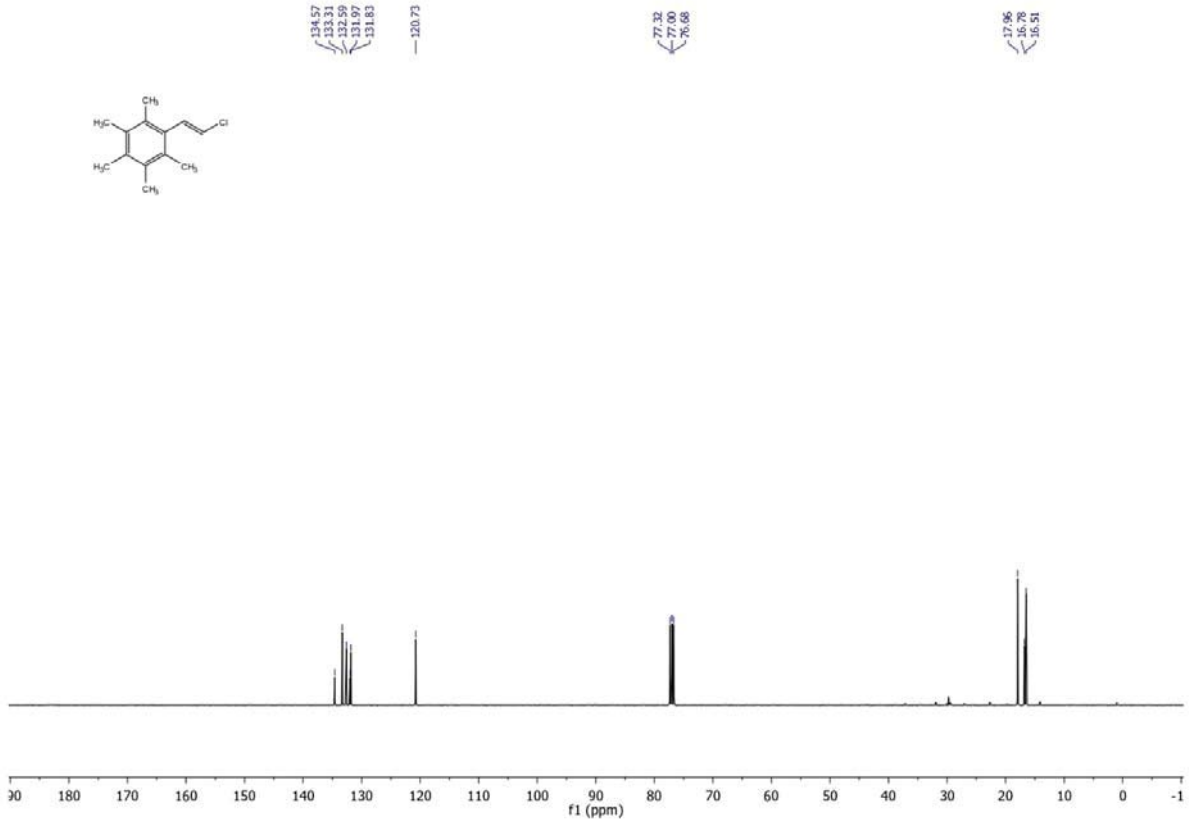

***(E)*-1-(benzyloxy)-2-(2-chlorovinyl)benzene (3m)**

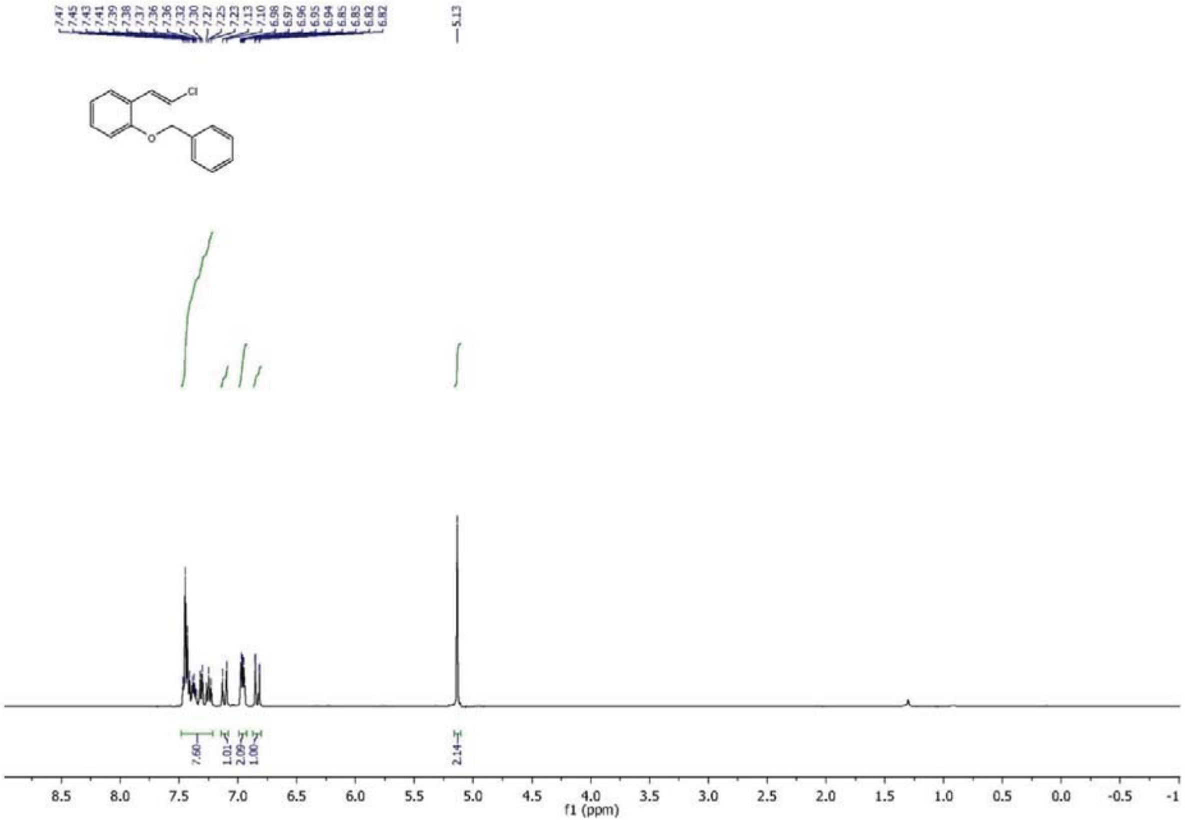

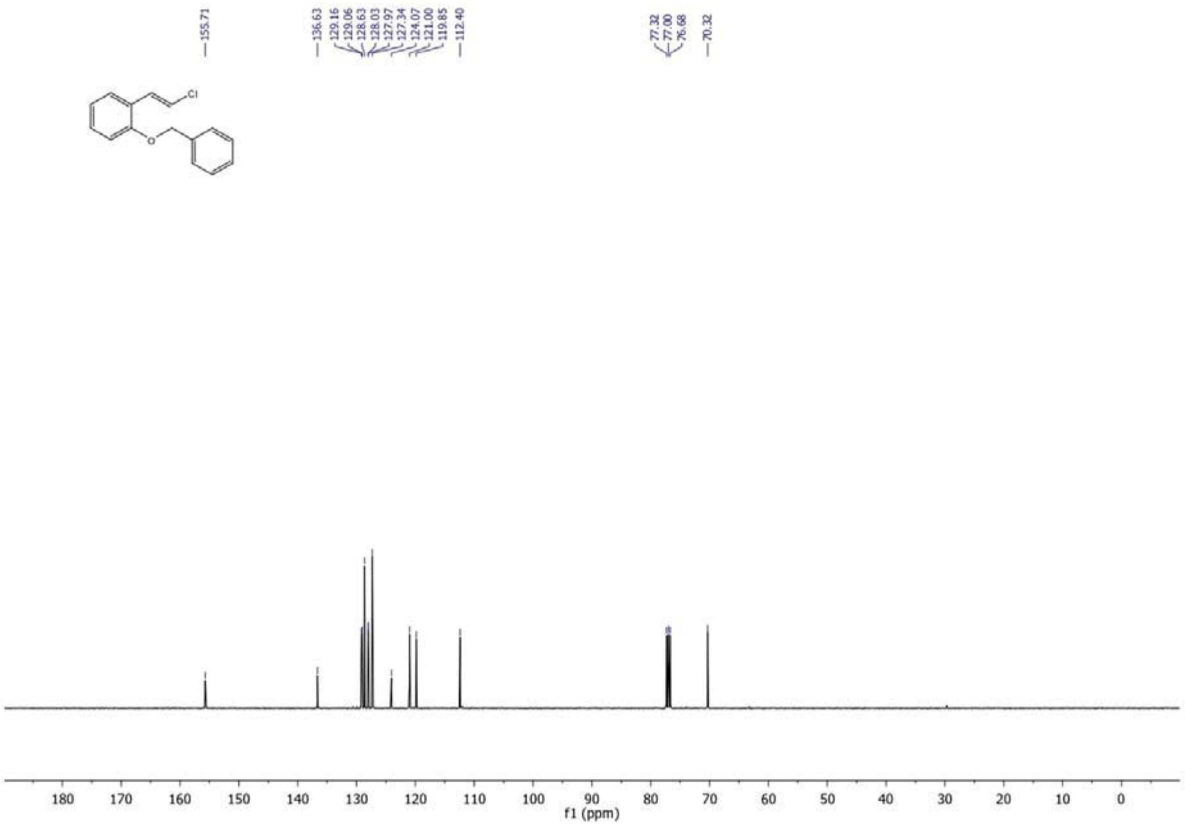

***(E)*-1-(2-chlorovinyl)-4-methoxybenzene (3n)**

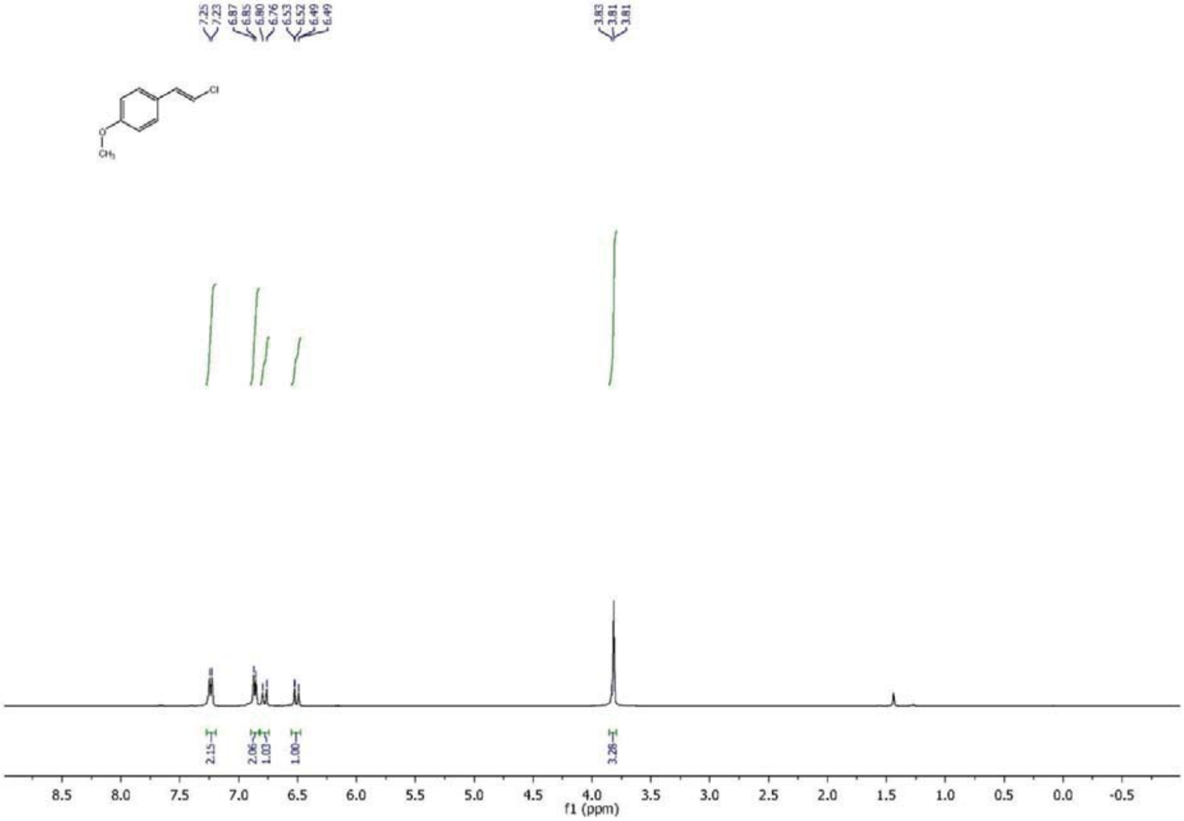

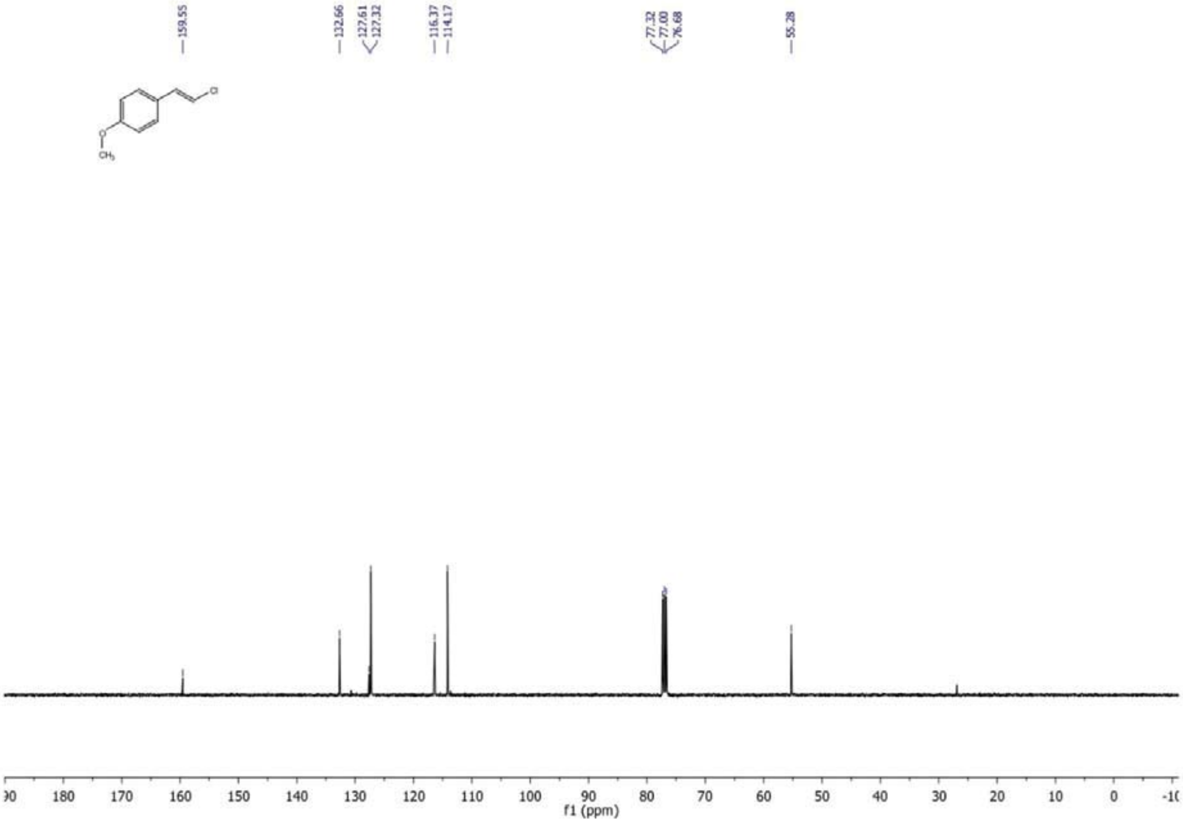

***(E)*-4-(2-chlorovinyl)-1,2-dimethoxybenzene (3o)**

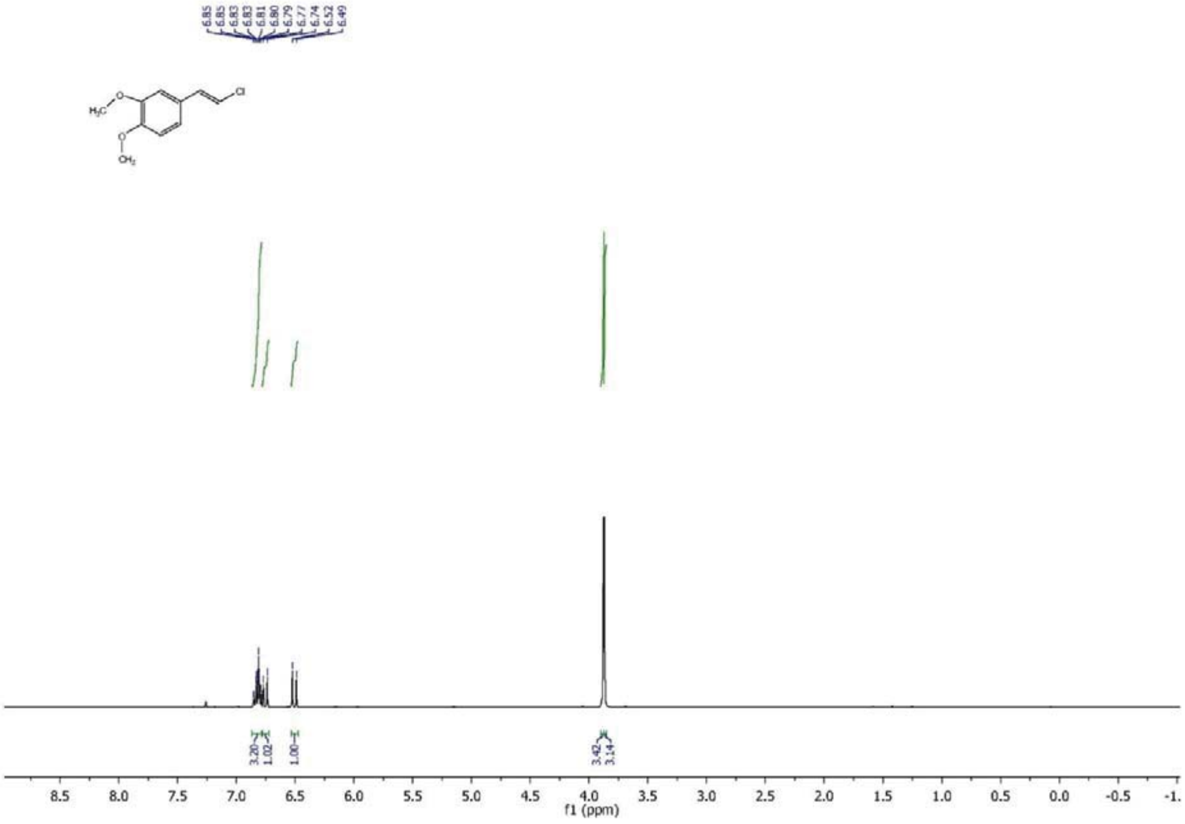

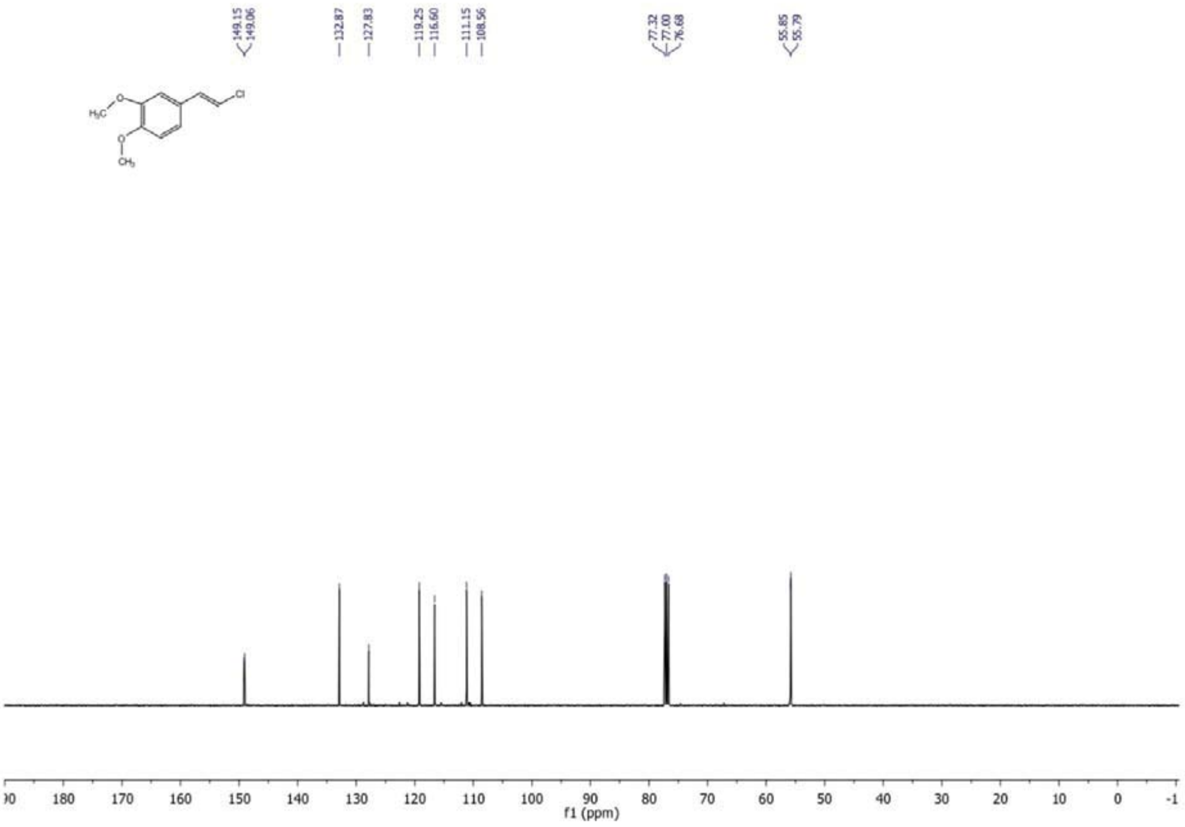

***(E)*-5-(2-chlorovinyl)benzo*[d]*[1,3]dioxole (3p)**

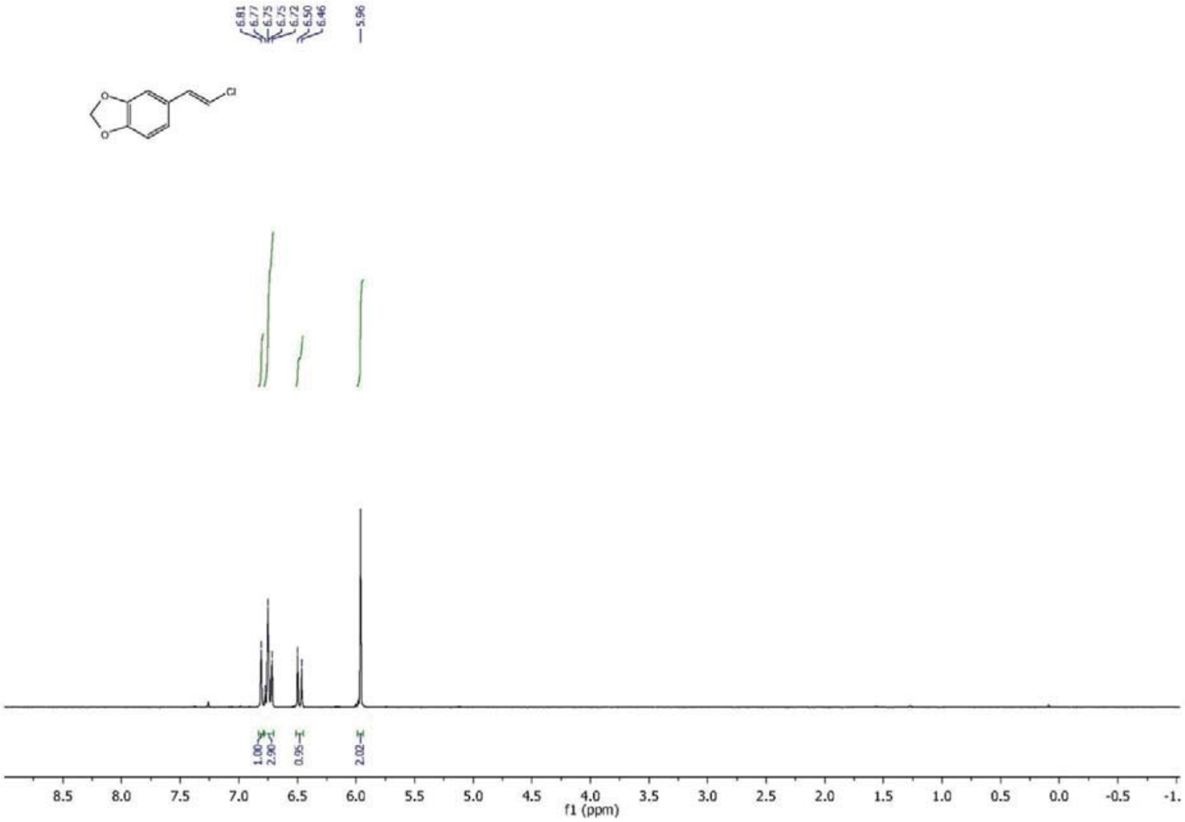

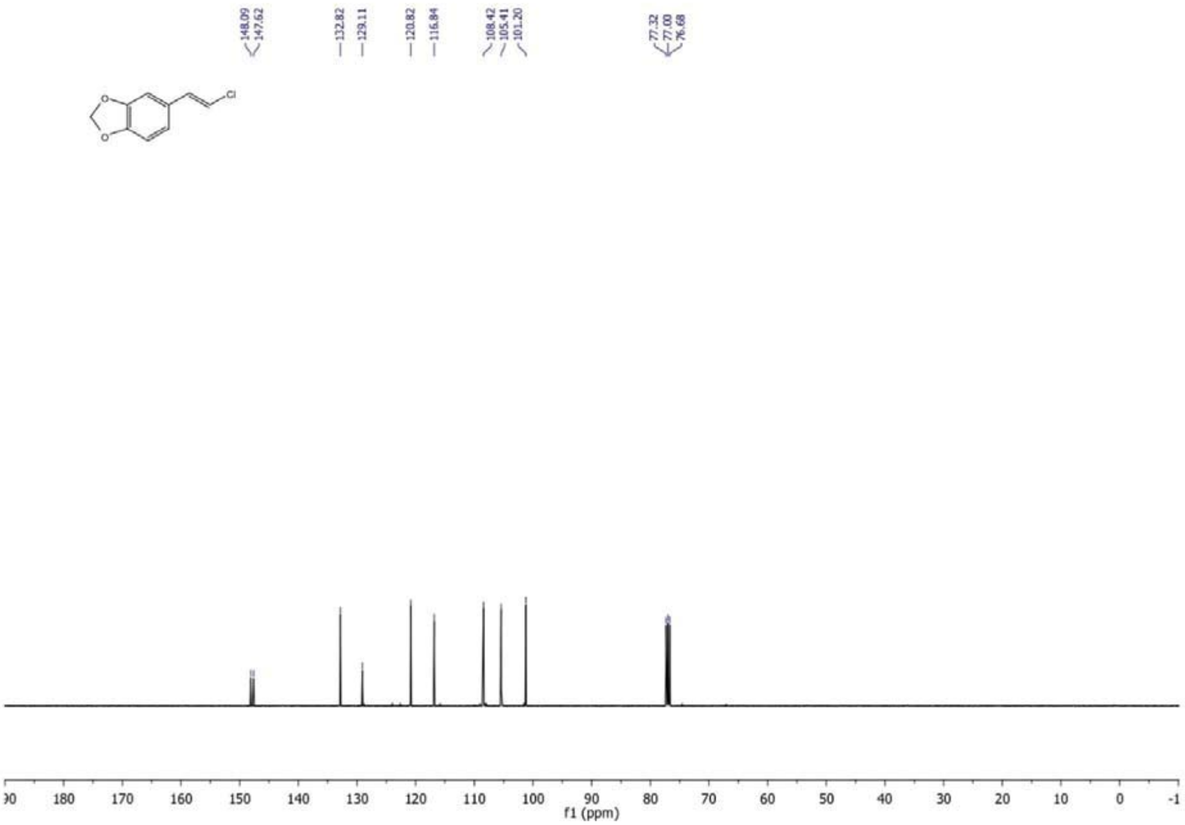

***(E)*-2-(2-chlorovinyl)-1,4-dimethoxybenzene (3q)**

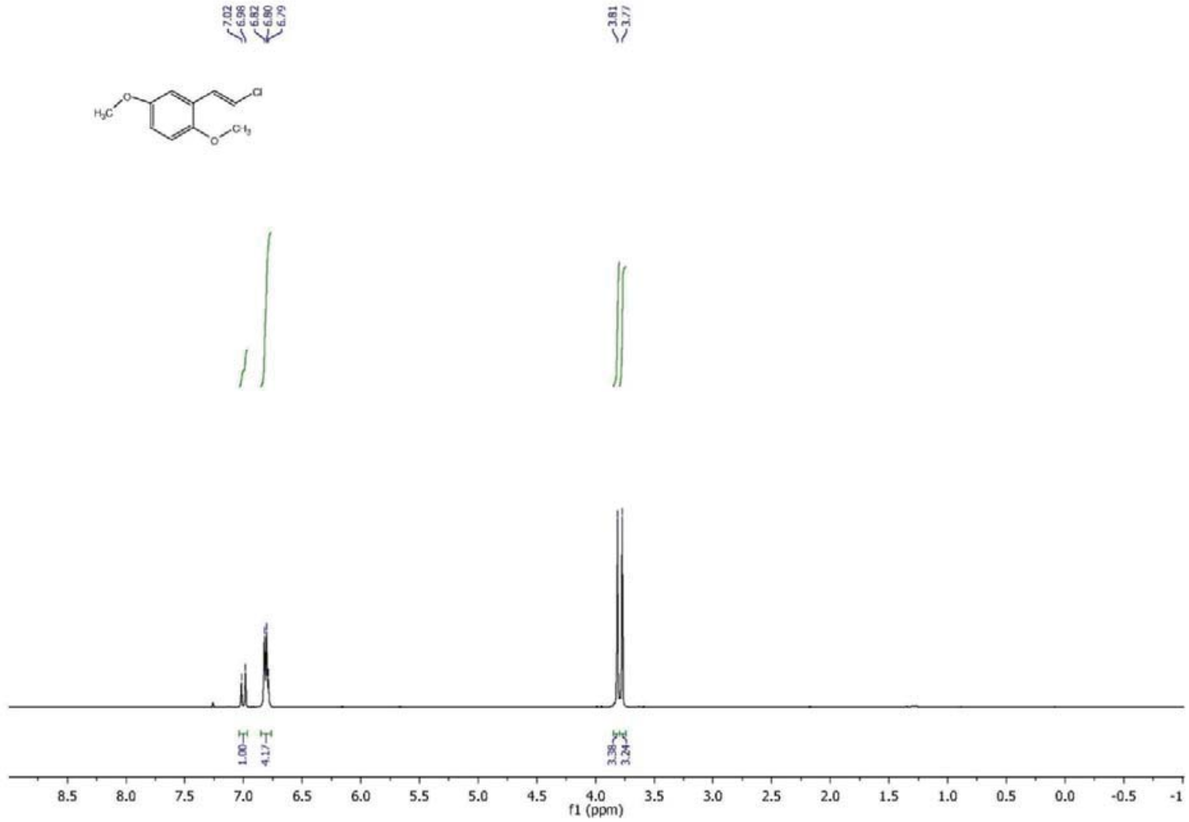

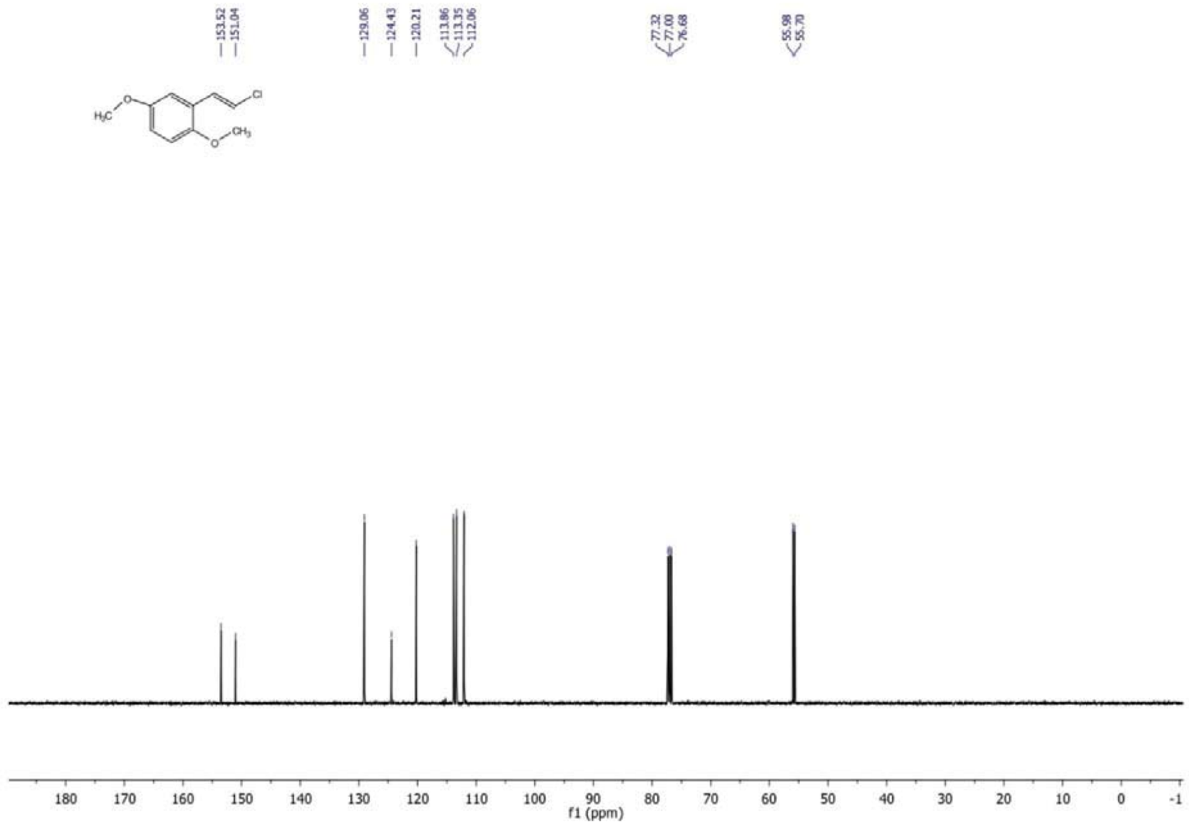

(E)-1-(2-chlorovinyl)-2-fluorobenzene (3r)

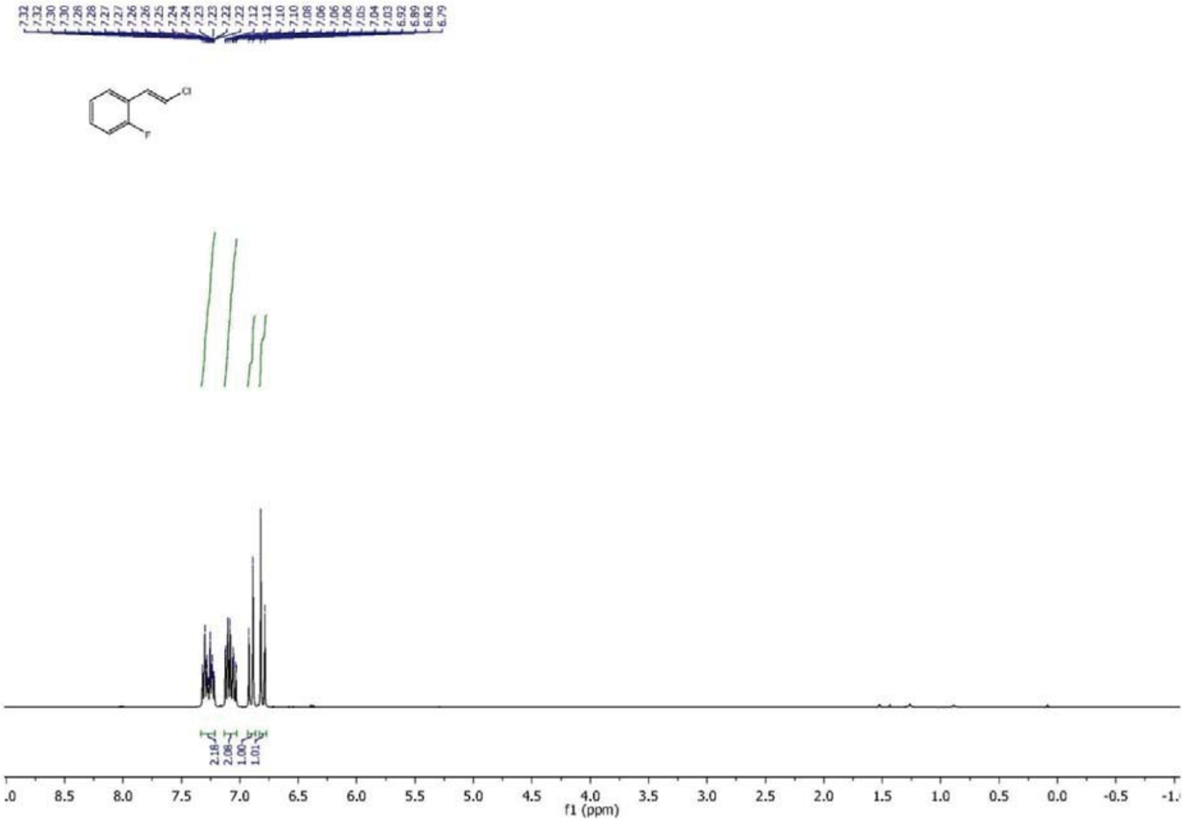

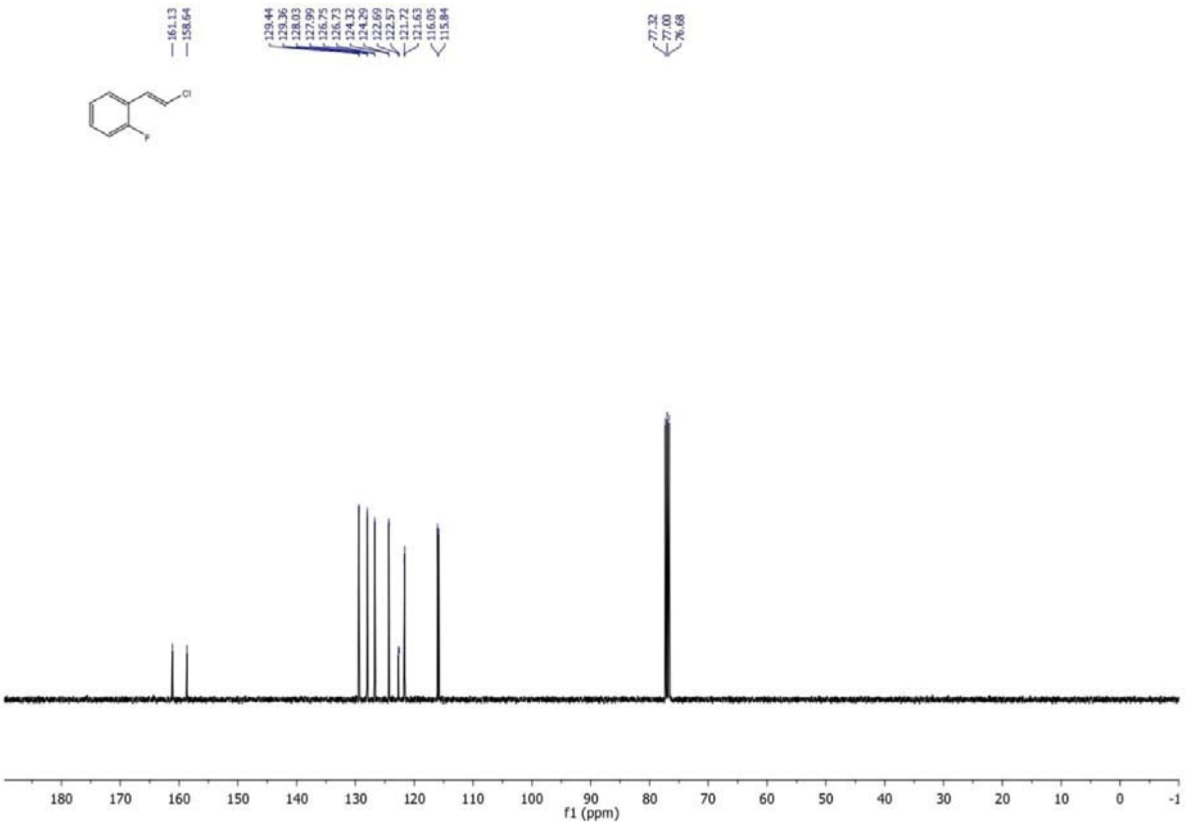

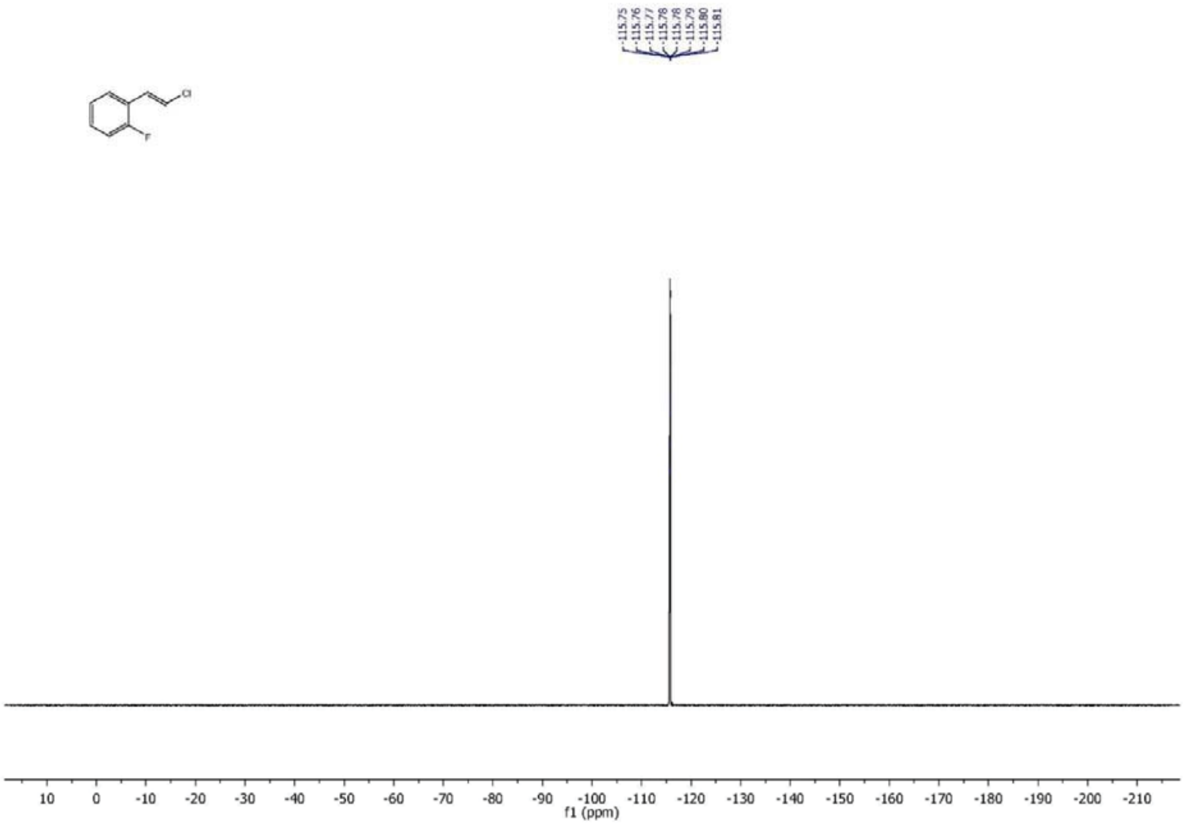

(E)-1-(2-chlorovinyl)-2-methylnaphthalene (3s)

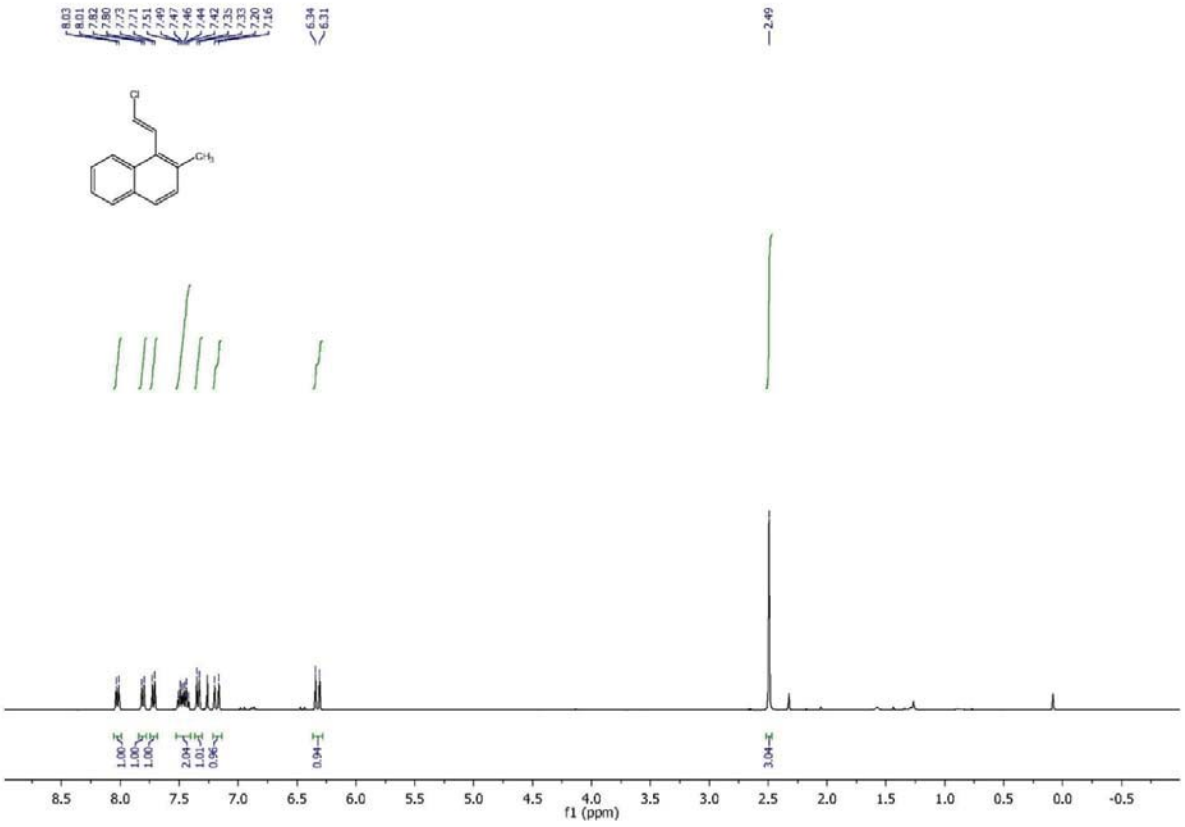

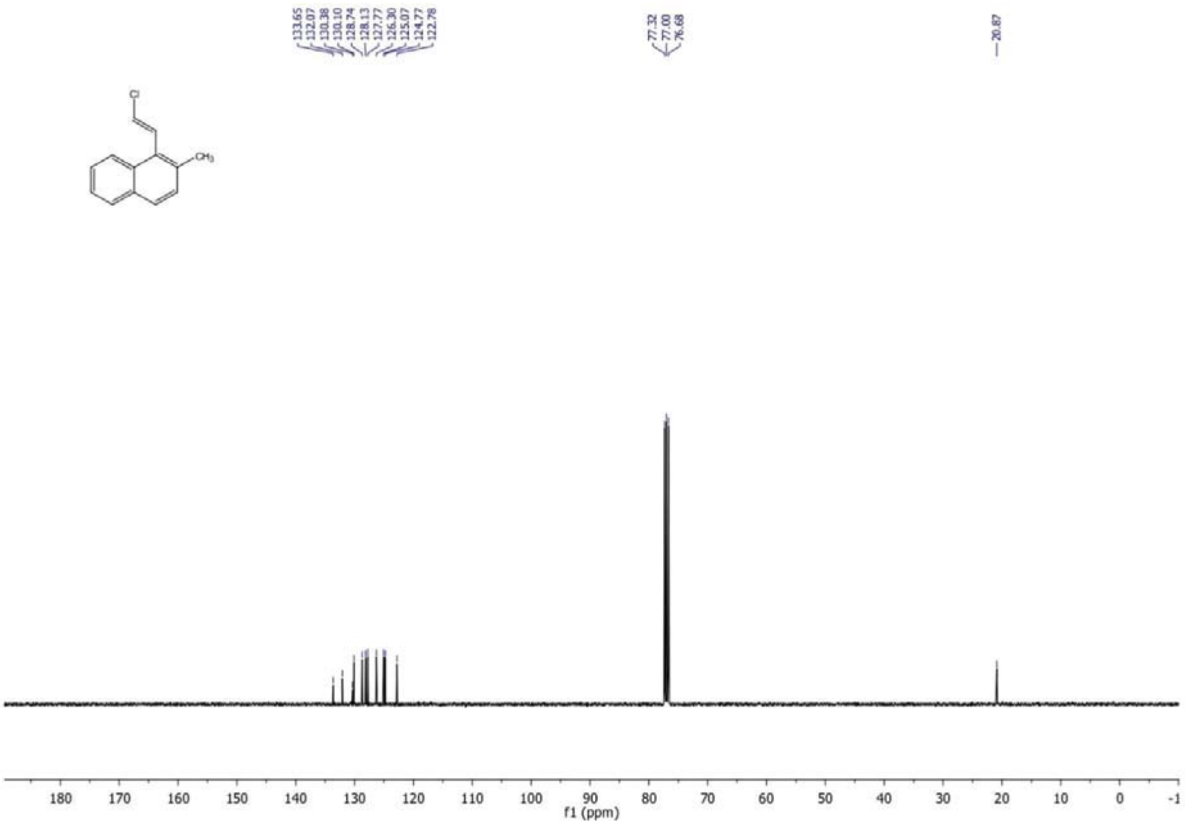

(E)-1-(2-chlorovinyl)naphthalene (3t)

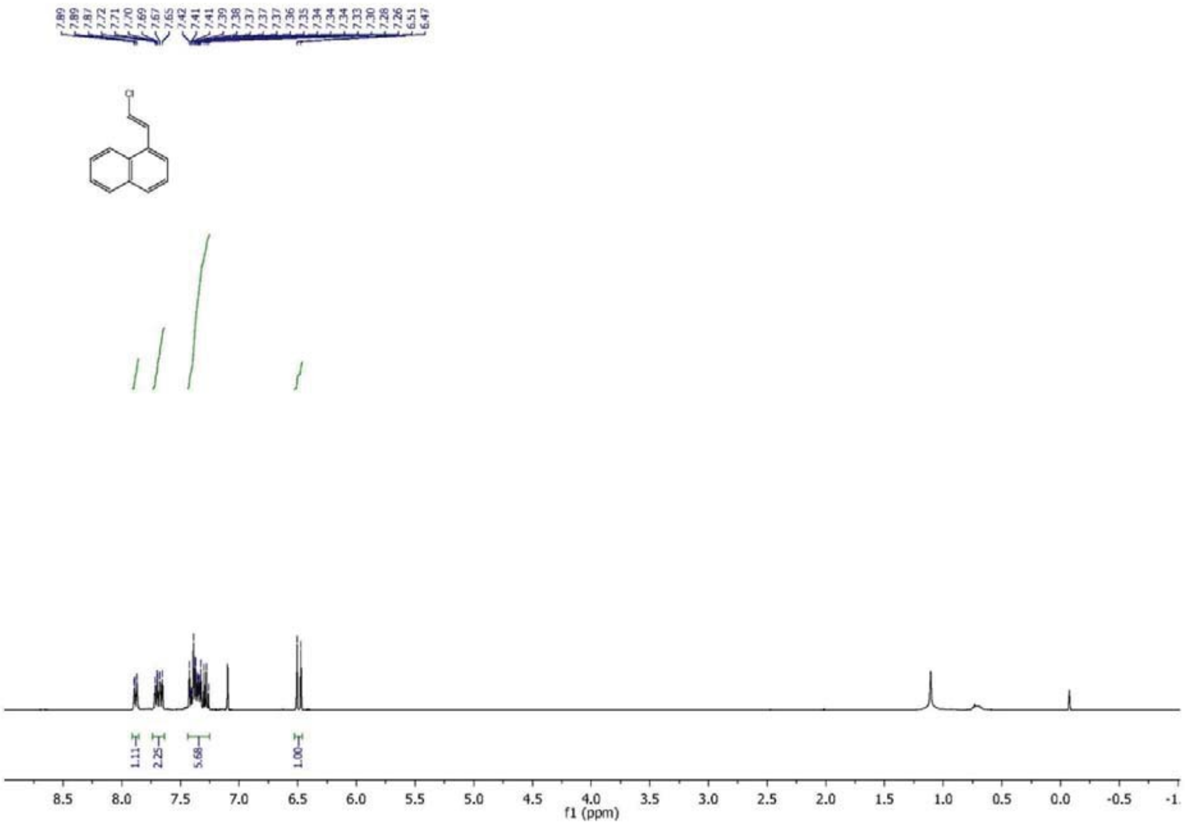

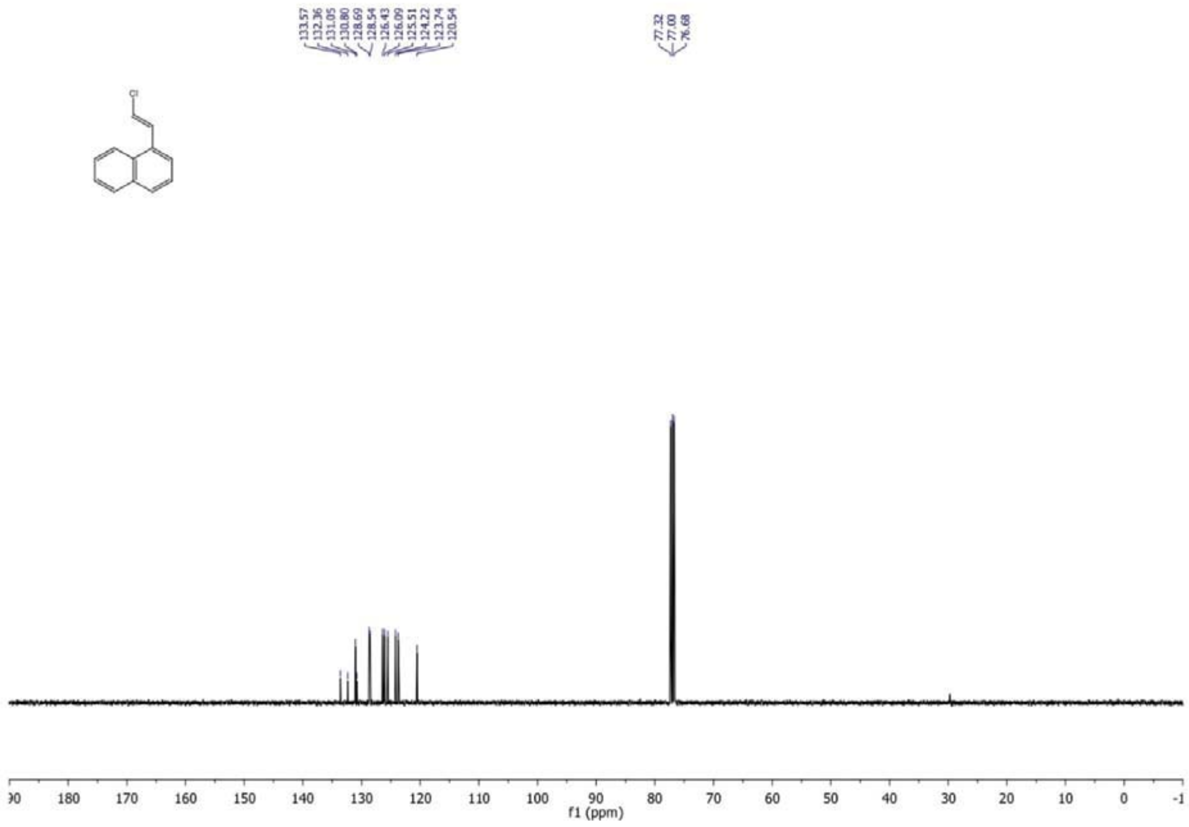

(*E*)-(1-chloroprop-1-en-2-yl)benzene (3u)

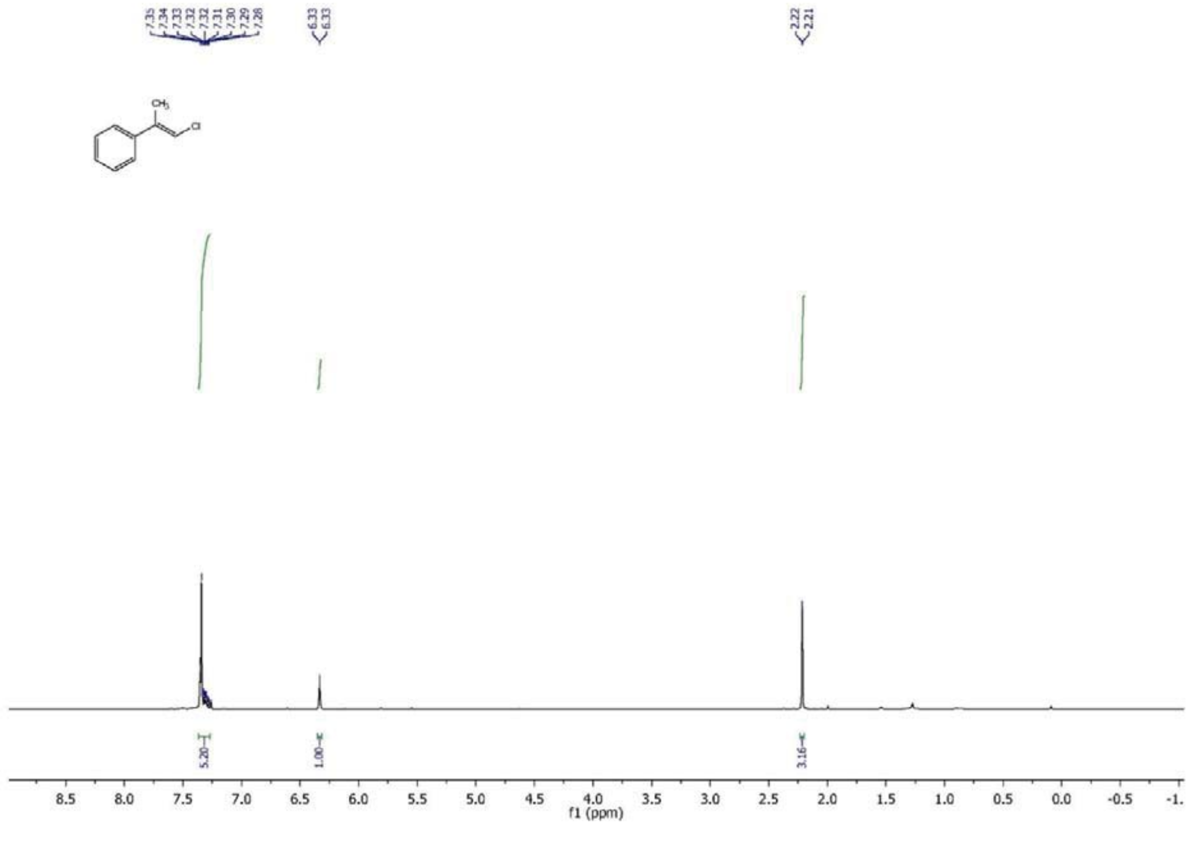

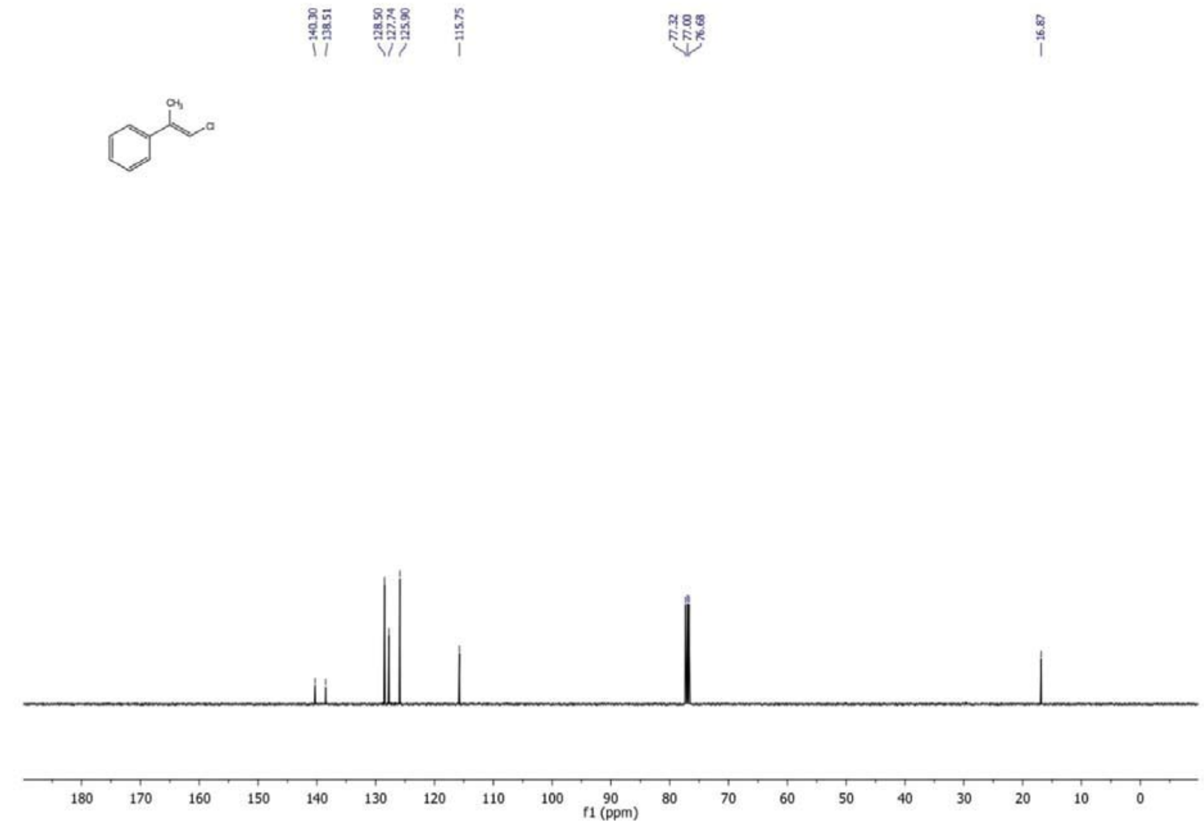

(2-chloroethene-1,1-diyl)dibenzene (3v)

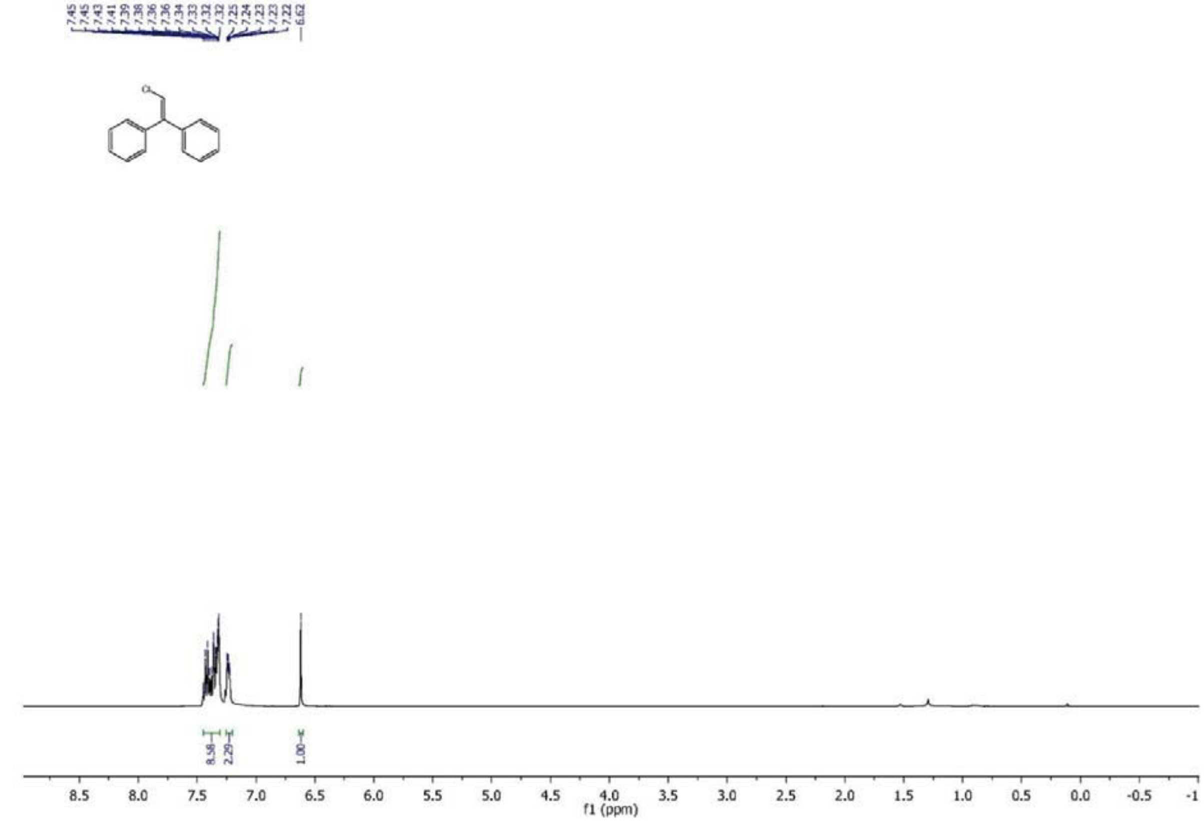

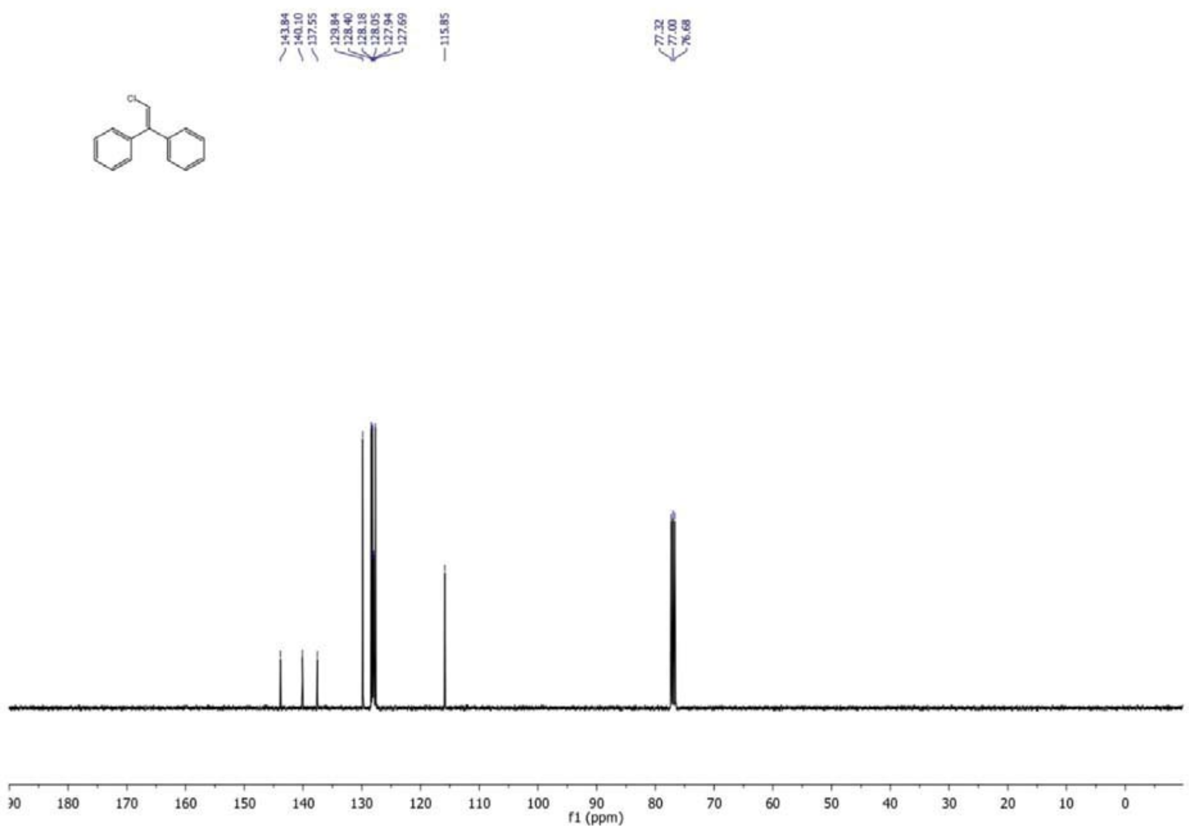

1,4-bis(*(E)*-2-chlorovinyl)benzene (3w)

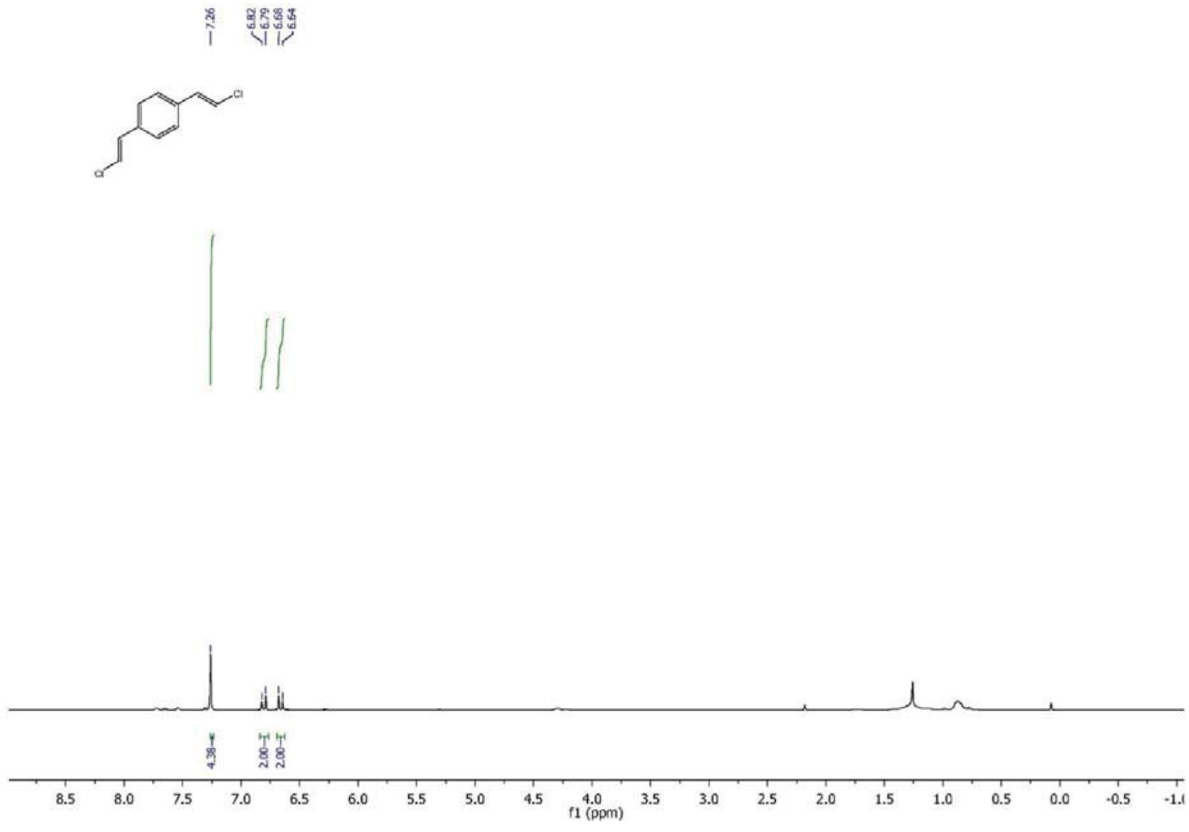

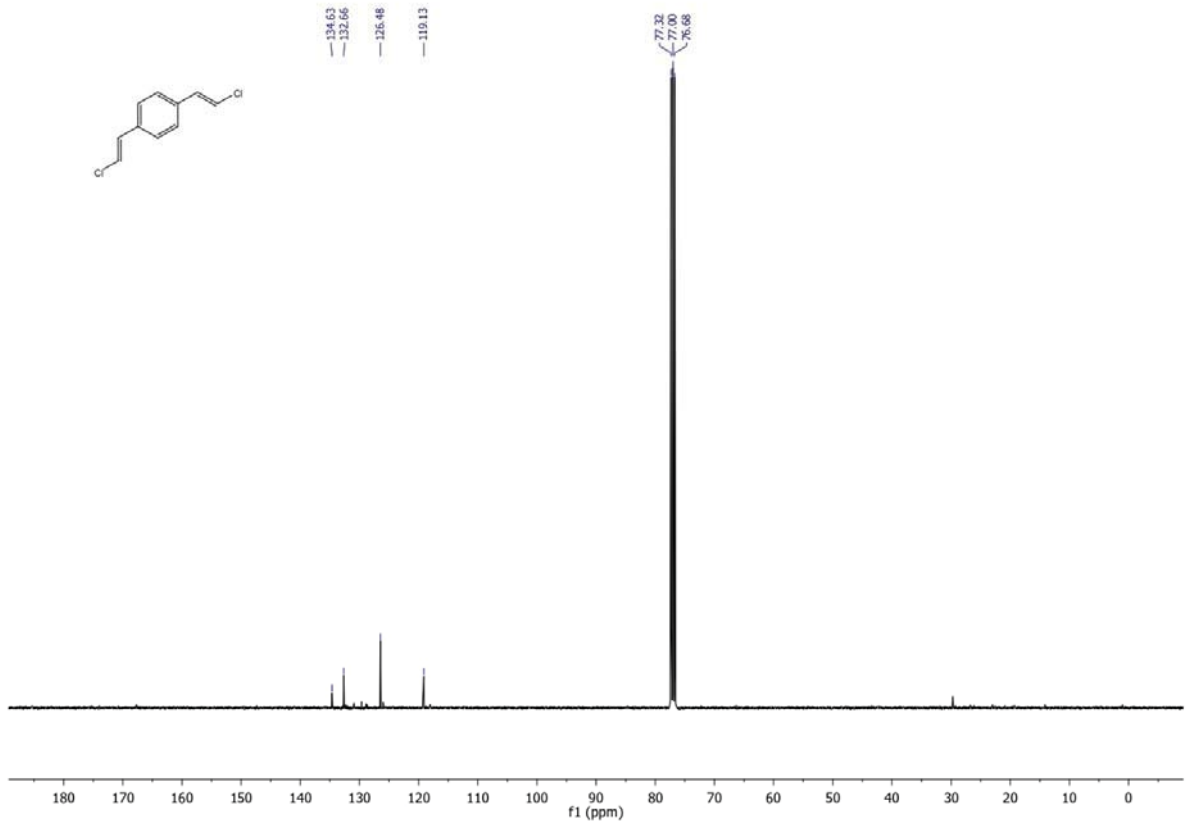

(*Z*)-(2-chlorovinyl)benzene (3x)

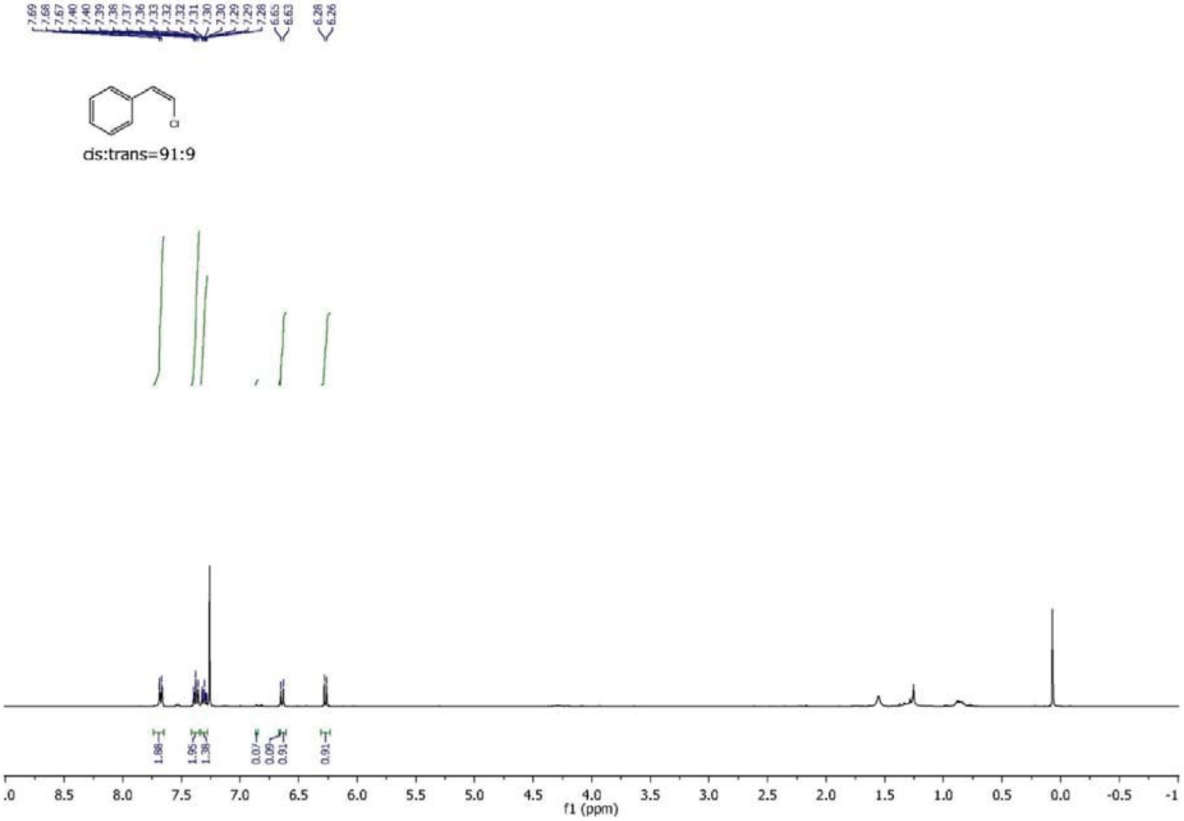

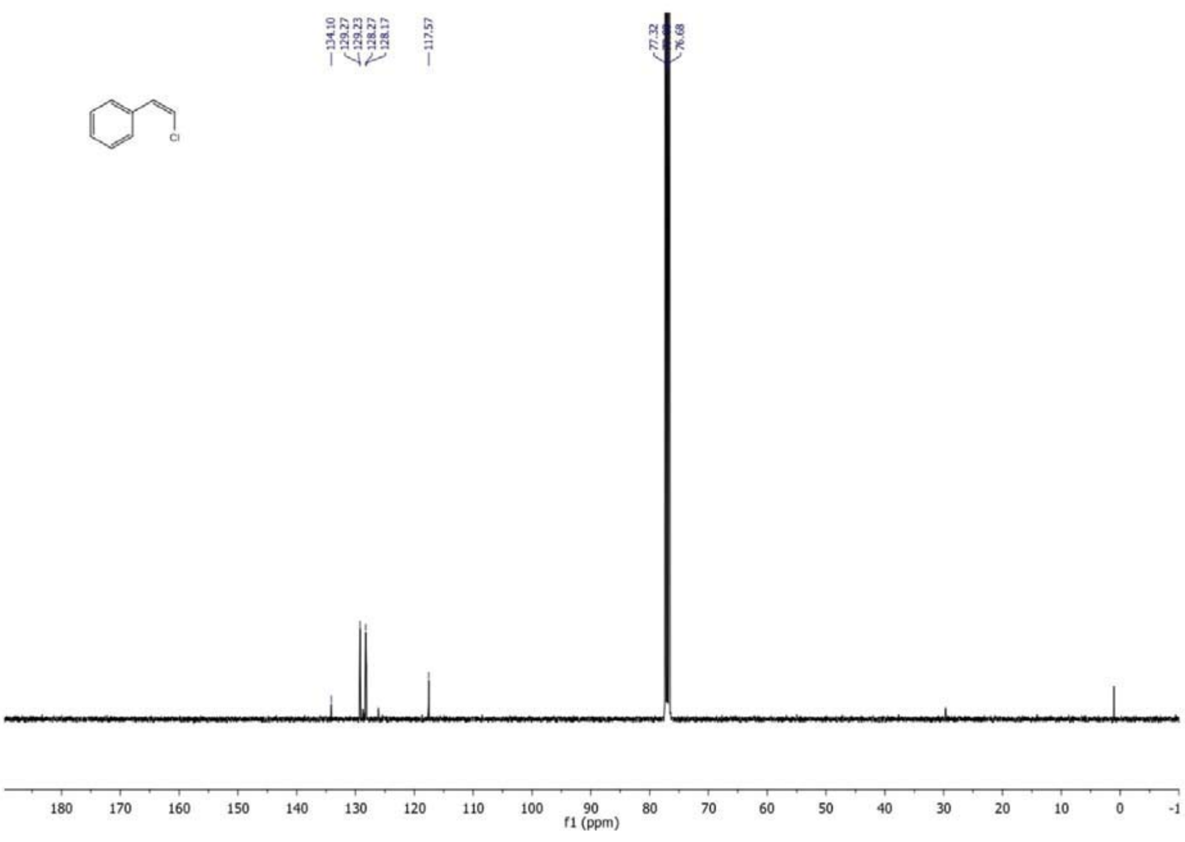
134.10
129.27
129.23
128.27
128.17
117.57
77.32
76.68
Cl
180 170 160 150 140 130 120 110 100 90 80 70 60 50 40 30 20 10 0 -1
f1 (ppm)
